# Supplementary material for: Electroreduction of unactivated alkenes using water as hydrogen source
Source: Nat Commun. 2024 Mar 30;15:2780. doi: 10.1038/s41467-024-47168-w (PMC10981685; doi:10.1038/s41467-024-47168-w)
Supplement: Supplementary file 1 — Supplementary Information [file 41467_2024_47168_MOESM1_ESM.pdf]

Supplementary Information

Electroreduction of Unactivated Alkenes using Water as Hydrogen Source

Yanwei Wang, Qian Wang, Lei Wu, Kangping Jia, Minyan Wang* and Youai Qiu*

State Key Laboratory and Institute of Elemento-Organic Chemistry, Frontiers Science Center for New Organic Matter, College of Chemistry, Nankai University, 94 Weijin Road, Tianjin, 300071, China

***qiuyouai@nankai.edu.cn**

Table of Contents

Table of Contents 1

1. Supplementary methods..2

1.1 General remarks 2

1.2 Preparation of starting materials..3

1.3 Optimization of electroreduction of unactivated alkenes........................7

1.4 General procedure of electroreduction of unactivated alkenes 11

1.5 Procedure of electroreduction of unactivated alkene 10 mmol scale......12

1.6 Characterization data of products 15

2. Supplementary Discussion 42

2.1 Mechanism research 42

2.2 Cyclic voltammetry 66

2.3 Copies of ^{1}H, ^{13}C and ^{19}F NMR spectra for compounds 68

3. Supplementary references..146

1. Supplementary methods

1.1 General remarks

Electroreductive reactions were carried out in undivided electrochemical cells (15 mL) using pre-dried glassware, if not noted otherwise. Iron plate electrodes (0.2 mm × 10.0 mm × 20.0 mm, 99.9%; obtained from Dingsheng scientific research metal materials, Hebei, China), nickel foam electrodes (0.3 mm × 10.0 mm × 20.0 mm, 99.9%; obtained from Guangjiayuan electronic materials Jiangsu, China), were connected using stainless steel adapters. Electroreduction was conducted using an HSPY-36-03 potentiostat in constant current mode. Cyclic Voltammetry studies were performed using a Shanghai Chenhua CHI760E workstation and Nova 2.0 software. Yields refer to isolated compounds, estimated to be >95% purity as determined by ^{1}H-NMR. Flash chromatography was performed using Silica gel (200-300 mesh) purchased from Qingdao Haiyang Chemical Co., China. NMR spectra were recorded on Bruker AVANCE AV 400 in the solvent indicated; chemical shifts (δ) are given in ppm relative to the residual solvent peak. Multiplicities are recorded as: s = singlet, d = doublet, t = triplet, dd = doublet of doublets, m = multiplet. High resolution mass spectrometric data were obtained on an Waters G2-XS QTOf UPLC/MS spectrometer (ESI-TOF) and AB_Triple TOF 5600 UPLC/MS spectrometer (ESI-TOF).

2

1.2 Preparation of starting materials

1.2.1 General procedure A:[1]

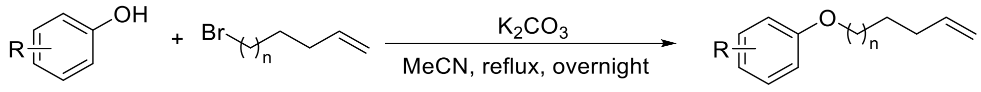

A mixture of phenol derivatives (10.0 mmol, 1.0 equiv.), alkene (13.0 mmol, 1.3 equiv.), and K_2CO_3 (15.0 mmol, 1.5 equiv.) in anhydrous acetonitrile (100 mL) was heated to reflux in an oil bath. After refluxing overnight, the reaction mixture was cooled to room temperature, concentrated under reduced pressure, diluted with saturated NH_4Cl (100 mL) and extracted with ethyl acetate (100 mL × 3). The combined organic layers were dried with Na_2SO_4, filtered, and concentrated under reduced pressure. The residue was purified by silica gel column chromatography to afford the corresponding pure product (Supplementary Figure 1).

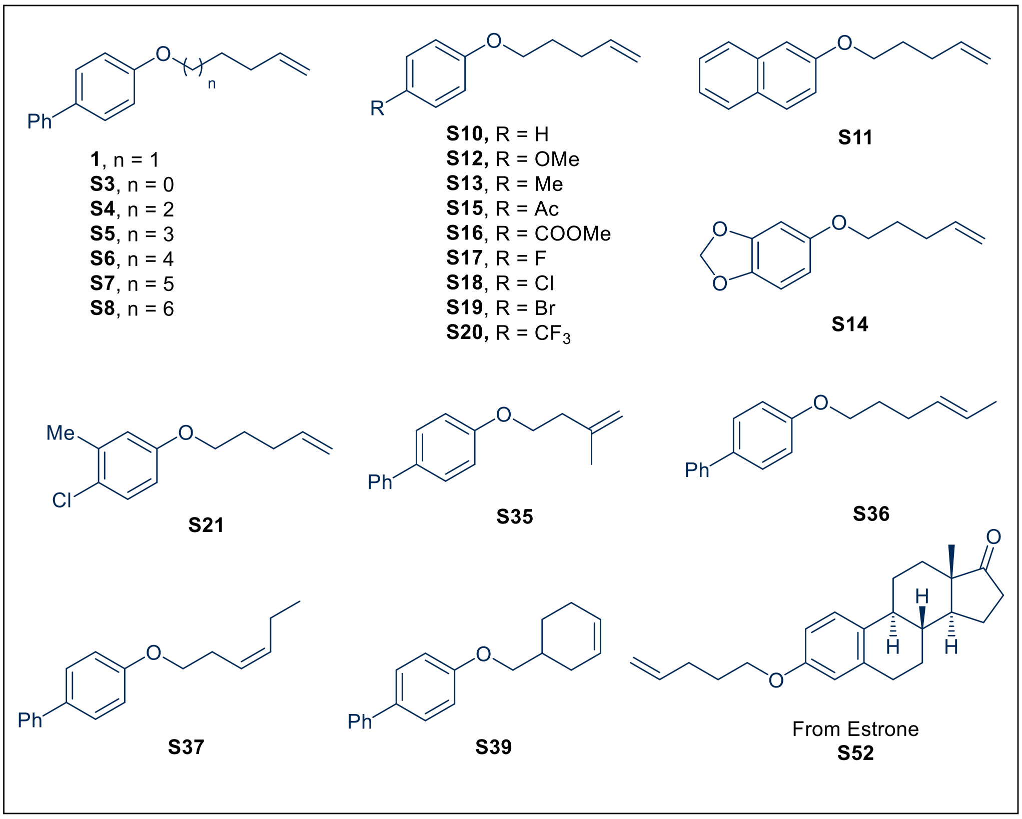

Supplementary Figure 1. Substrates synthesized according to general procedure **A**

1.2.2 General procedure B:[2a]

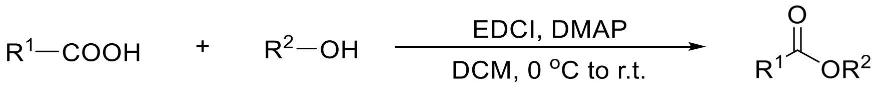

The corresponding acid (10.0 mmol, 1.0 equiv.) was added to a solution of 1-(3-dimethylaminopropyl)-3-ethylcarbodiimide (EDCI, 13.0 mmol, 1.3 equiv.) and DMAP (1.0 mmol 0.1 equiv.) in CH_2Cl_2 (25 mL) at 0 °C. Alcohol (12.0 mmol 1.2 equiv.) was then added. The reaction mixture was allowed to warm to room temperature and stirred overnight. The solution was diluted with CH_2Cl_2 (40 mL) and washed with 1 N HCl (3×20 mL), saturated $NaHCO_3$ (40 mL), brine (40 mL) sequentially. The organic layer was dried over anhydrous Na_2SO_4. After removal of solvent under reduced pressure, the crude product was purified by column chromatography on silica gel (Supplementary Figure 2).

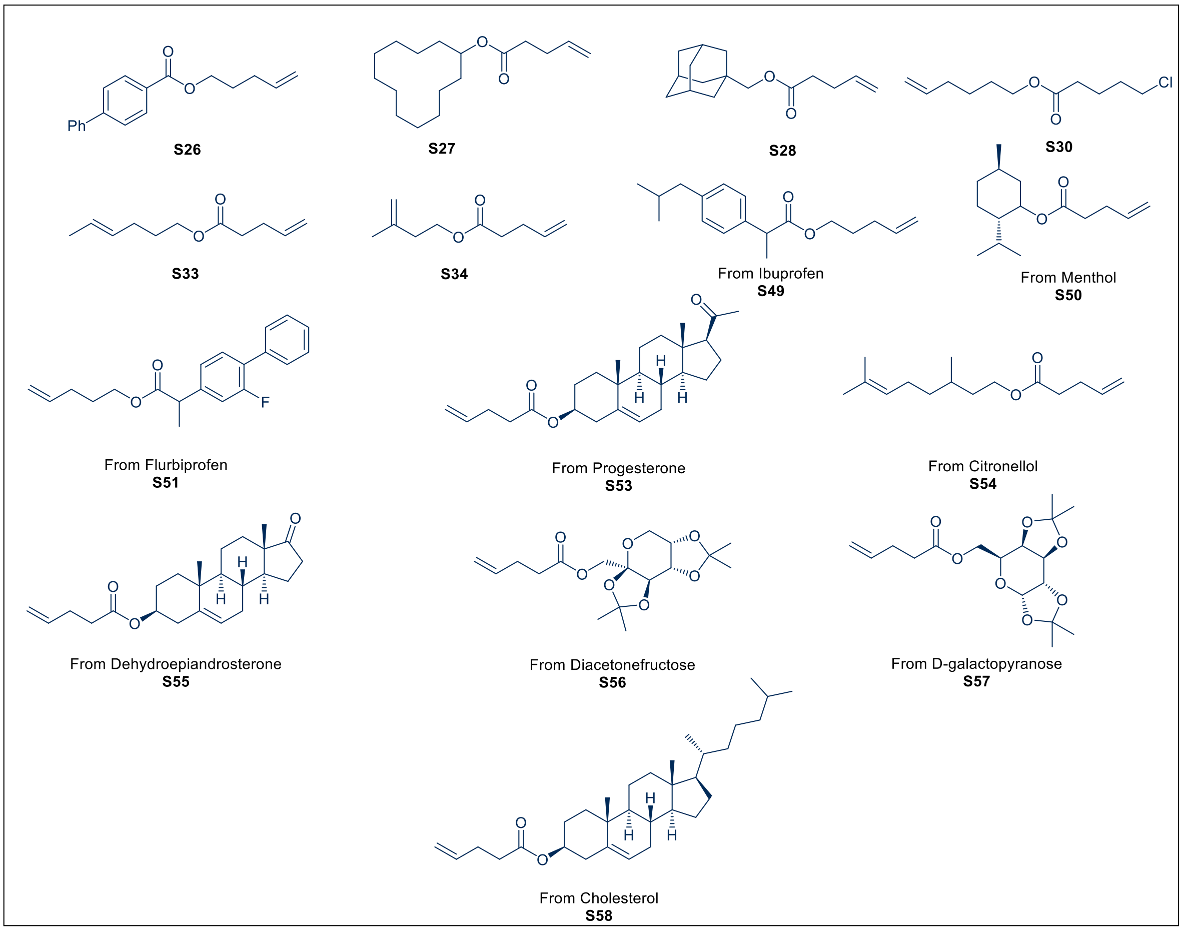

Supplementary Figure 2. Substrates synthesized according to general procedure **B**

1.2.3 General procedure C:[2b]

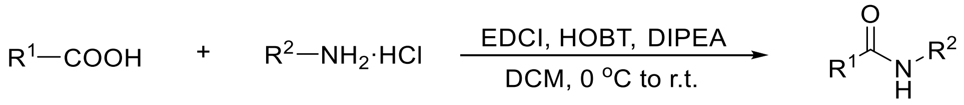

The corresponding acid (10.0 mmol, 1.0 equiv.) and primary amine hydrochloride were added to a solution of 1-(3-dimethylaminopropyl)-3-ethylcarbodiimide (EDCI, 10.0 mmol, 1.0 equiv.) and HOBT (10.0 mmol, 1.0 equiv.) in CH_2Cl_2 (25 mL) at 0 °C. DIPEA (40.0 mmol, 4.0 equiv.) was then added. The reaction mixture was allowed to warm to room temperature and stirred overnight. The solution was diluted with CH_2Cl_2 (40 mL) and washed with 1N HCl (3×20 mL), saturated $NaHCO_3$ (40 mL), brine (40 mL) sequentially. The organic layer was dried over anhydrous Na_2SO_4. After removal of solvent under reduced pressure, the crude product was purified by column chromatography on silica gel (Supplementary Figure 3).

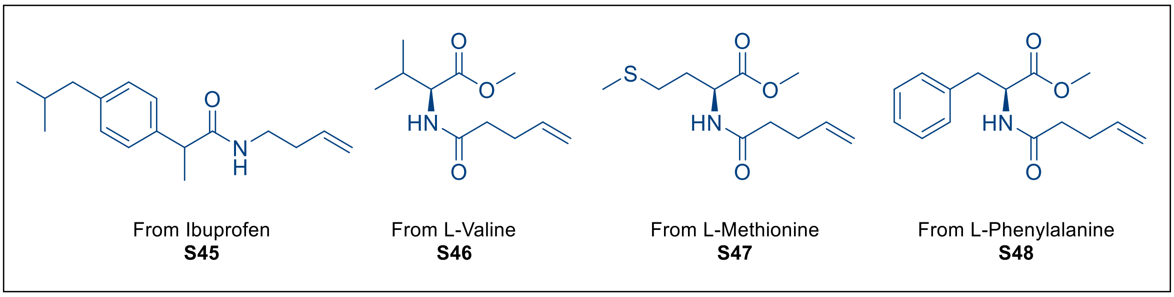

Supplementary Figure 3. Substrates synthesized according to general procedure **C**

1.2.4 General procedure D:[3]

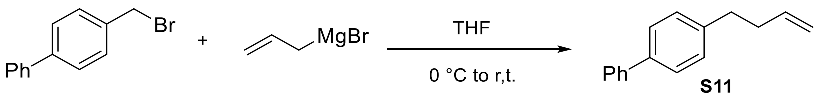

Benzyl bromide (10.0 mmol, 1.0 equiv.) and 20 mL anhydrous THF were added to a flame dried 100 mL round-bottom flash assembled with a constant pressure funnel which had been previously flame dried. Allyl magnesium bromide (20.0 mmol, 2.0 equiv.) in the constant pressure funnel were added dropwise. The reaction was stirred for 4 h at room temperature and then quenched with saturated aqueous NH_4Cl. The aqueous layer was extracted with CH_2Cl_2 and the combined organics were dried over $MgSO_4$, filtered through celite and

5

concentrated under reduced pressure. The crude product was purified by flash chromatography (eluent: hexane), affording the desired product as a clear colorless oil.

6

1.3 Optimization of electroreduction of unactivated alkenes

Supplementary Table 1: The effect of constant current.[a]

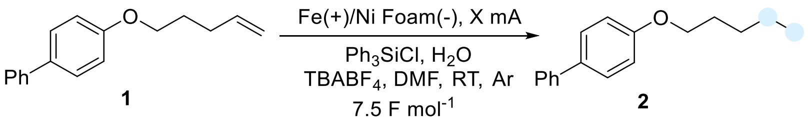

Entry	**Current** (X mA)	Yield (%)[a]
1	5	71
2	***6***	***92***
3	7	91
4	8	85
5	9	83
6	10	86

[a]Reaction conditions: undivided cell, Fe as anode, Ni Foam as cathode, constant current = X mA, **1** (0.3 mmol, 1.0 equiv.), Ph_3SiCl (20 mol%), H_2O (3.0 equiv.), $TBABF_4$ (1.0 equiv.) in DMF (4.0 mL), room temperature, 10 h, under Ar atmosphere. Yields were determined by ^{1}H NMR spectroscopy using 1,3,5-trimethoxybenzene as the internal standard.

Supplementary Table 2: The effect of [Si]-source.[a]

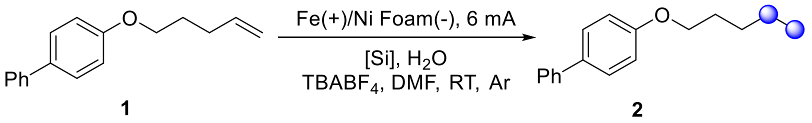

Entry	[Si]-source	Yield (%)
1	TMSCl	89
2	TMSBr	86
3	***Ph_3SiCl***	***92***
4	Ph_3SiF	60
5	$PhMe_2SiCl$	90
6	$(TMS)_2O$	90
7	Ph_3SiH	73
8	Ph_3SiOH	56
9	$Ph_3SiOCH_2CF_3$	80
10	w/o [Si]	trace

[a]Reaction conditions: undivided cell, Fe as anode, Ni Foam as cathode, constant current = 6 mA, **1** (0.3 mmol, 1.0 equiv.), [Si] (20 mol%), H_2O (3.0 equiv.), $TBABF_4$ (1.0 equiv.) in DMF (4.0 mL), room temperature, 10 h, under Ar atmosphere. Yields were determined by ^{1}H NMR spectroscopy using 1,3,5-trimethoxybenzene as the internal standard.

7

Supplementary Table 3: The effect of the Ph_3SiCl loading.[a]

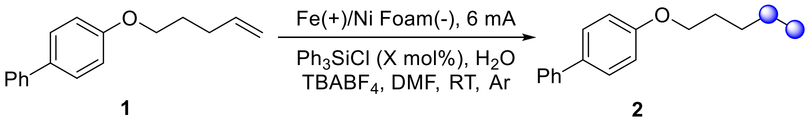

Entry	Ph_3SiCl (X mol%)	Yield (%)
1	0	Trace
2	5	52
3	10	76
4	15	89
5	***20***	***92***
6	30	62
7	50	24

[a]Reaction conditions: undivided cell, Fe as anode, Ni Foam as cathode, constant current = 6 mA, **1** (0.3 mmol, 1.0 equiv.), Ph_3SiCl (X mol%), H_2O (3.0 equiv.), $TBABF_4$ (1.0 equiv.) in DMF (4.0 mL), room temperature, 10 h, under Ar atmosphere. Yields were determined by ^1H NMR spectroscopy using 1,3,5-trimethoxybenzene as the internal standard.

Supplementary Table 4: The effect of electrodes.[a]

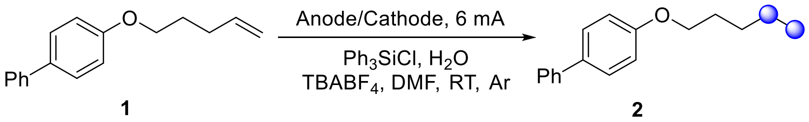

Entry	Anode/Cathode	Yield (%)
1	***Fe(+)/Ni Foam(-)***	***92***
2	Mg(+)/Ni Foam(-)	0
3	Zn(+)/Ni Foam(-)	0
4	Al(+)/Ni Foam(-)	12
5	GF(+)/Ni Foam(-)	0
6	Fe(+)/Fe (-)	90
7	Fe(+)/GF (-)	75
8	Fe(+)/Ni Plate (-)	58

[a]Reaction conditions: undivided cell, Anode/Cathode, constant current = 6 mA, **1** (0.3 mmol, 1.0 equiv.), Ph_3SiCl (20 mol%), H_2O (3.0 equiv.), $TBABF_4$ (1.0 equiv.) in DMF (4.0 mL), room temperature, 10 h, under Ar atmosphere. Yields were determined by ^1H NMR spectroscopy using 1,3,5-trimethoxybenzene as the internal standard.

Supplementary Table 5: The effect of H-source.[a]

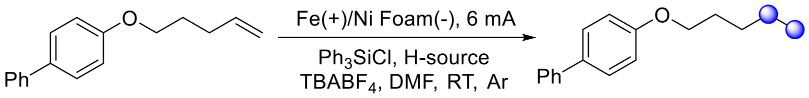

1 2

Entry	H-source	Yield (%)
1	***H_2O***	***92***
2	MeOH	93
3	EtOH	91
4	CF_3CH_2OH	90
5	^{i}PrOH	86
6	AcOH	40

[a]Reaction conditions: undivided cell, Fe as anode, Ni Foam as cathode, constant current = 6 mA, **1** (0.3 mmol, 1.0 equiv.), Ph_3SiCl (20 mol%), H-source (3.0 equiv.), $TBABF_4$ (1.0 equiv.) in DMF (4.0 mL), room temperature, 12 h, under Ar atmosphere. Yields were determined by ^1H NMR spectroscopy using 1,3,5-trimethoxybenzene as the internal standard.

Supplementary Table 6: The effect of the H_2O loading.[a]

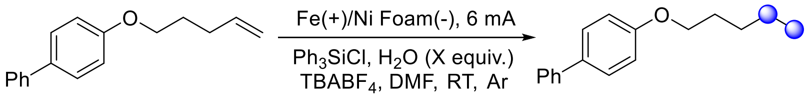

1 2

Entry	H_2O (X equiv.)	Yield (%)
1	0	22
2	1	68
3	2	81
4	***3***	***92***
5	4	68
6	5	72

[a]Reaction conditions: undivided cell, Fe as anode, Ni Foam as cathode, constant current = 6 mA, **1** (0.3 mmol, 1.0 equiv.), Ph_3SiCl (20 mol%), H-source (3.0 equiv.), $TBABF_4$ (1.0 equiv.) in DMF (4.0 mL), room temperature, 10 h, under Ar atmosphere. Yields were determined by ^1H NMR spectroscopy using 1,3,5-trimethoxybenzene as the internal standard.

9

Supplementary Table 7: The effect of solvents.[a]

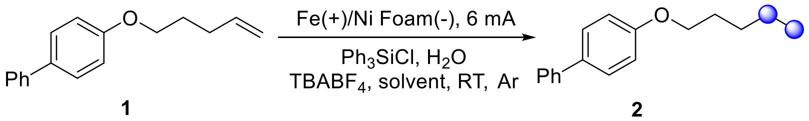

Entry	Solvent	Yield (%)
1	***DMF***	***92***
2	DMA	10
3	DMSO	0
4	NMP	20
5	CH_3CN	0

[a]Reaction conditions: undivided cell, Fe as anode, Ni Foam as cathode, constant current = 6 mA, **1** (0.3 mmol, 1.0 equiv.), Ph_3SiCl (20 mol%), H-source (3.0 equiv.), $TBABF_4$ (1.0 equiv.) in DMF (4.0 mL), room temperature, 10 h, under Ar atmosphere. Yields were determined by ^1H NMR spectroscopy using 1,3,5-trimethoxybenzene as the internal standard.

Supplementary Table 8: Further investigations of other parameters.[a]

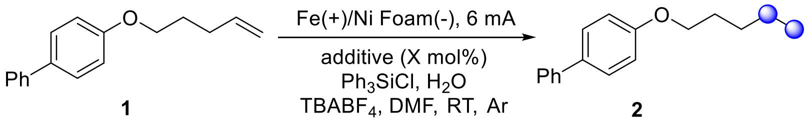

Entry	Additive (X mol%)	Yield (%)
1	$NiBr_2$·dtbpy	95
2	$CoBr_2$·dtbpy	94
3	Fe(phen)Cl_3	91
4	Terephthalonitrile	88
5	Methyl terephthalate	84
6	$NiBr_2$·dtbpy	99[b]

[a]Reaction conditions: undivided cell, Fe as anode, Ni Foam as cathode, constant current = 6 mA, **1** (0.3 mmol, 1.0 equiv.), Ph_3SiCl (20 mol%), H-source (3.0 equiv.), $TBABF_4$ (1.0 equiv.) in DMF (4.0 mL), room temperature, 10 h, under Ar atmosphere. Yields were determined by ^1H NMR spectroscopy using 1,3,5-trimethoxybenzene as the internal standard. [b]5 mA.

10

1.4 General procedure of electroreduction of unactivated alkenes

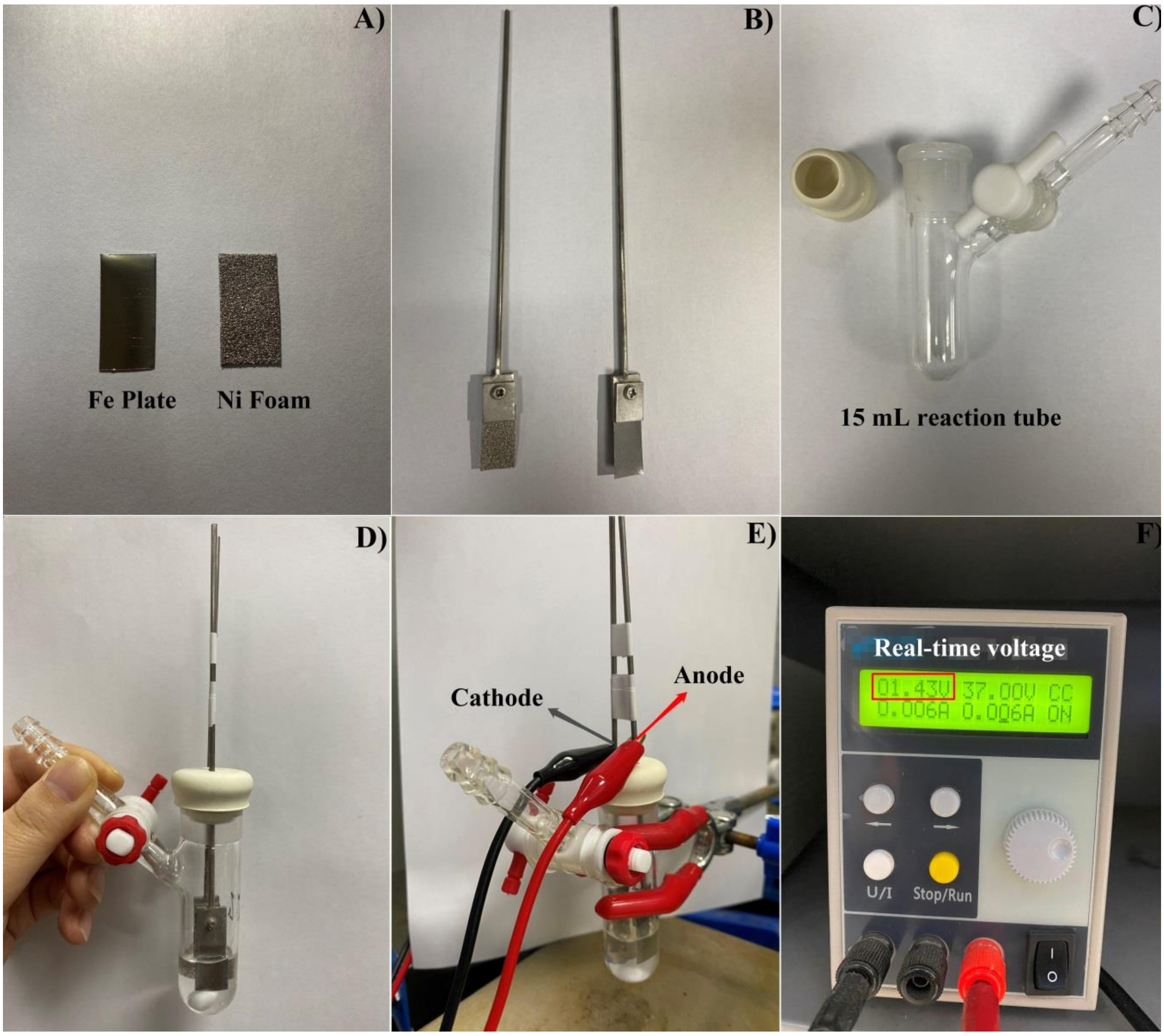

Supplementary Figure 4. Pictures of the reaction setups for electrochemical reduction of unactivated alkenes.

The electrolysis process of 0.3 mmol scale (supplementary Figure 4) was carried out in an undivided cell with a Fe plate anode (10 mm × 20 mm × 0.10 mm) and a Ni Foam cathode (10 mm × 20 mm × 0.30 mm). To a 15 mL oven-dried undivided electrochemical cell equipped with a magnetic bar was added unactivated alkene (0.30 mmol, 1.0 equiv), $NiBr_2$·dtbpy (7.3 mg, 0.015 mmol, 5 mol%), Ph_3SiCl (17.7 mg, 0.06 mmol, 20 mol%), H_2O (16.2 mg, 0.9 mmol, 3.0 equiv.) and $^{n}Bu_4NBF_4$ (98.8 mg, 0.30 mmol, 1.0 equiv.) under Ar atmosphere. The electrolysis process was performed at 5.0 mA of constant current for 10 h at room temperature. After that, the electrodes were washed with EtOAc (3 x 5 mL) in an ultrasonic bath. H_2O (20 mL) was added to the organic system, and the resulting mixture was

11

extracted with EtOAc (3 x 20 mL) and the combined organic phase was washed with brine, dried by anhydrous $MgSO_4$, filtered, and concentrated in vacuo. The crude product was purified by column chromatography to furnish the desired product.

Supplementary Note 1: Chlorosilane is sensitive to moisture, and should be conducted in a glovebox.

Supplementary Note 2: If the product is not sensitive to acid, 5 mL of 1 N HCl aq. could be added after the completion of the reaction to help the removal of the excess scrap iron from the reaction system.

1.5 Procedure of electroreduction of unactivated alkene (1) in 10 mmol scale

Pictures of the reaction setups for the 10 mmol scale:

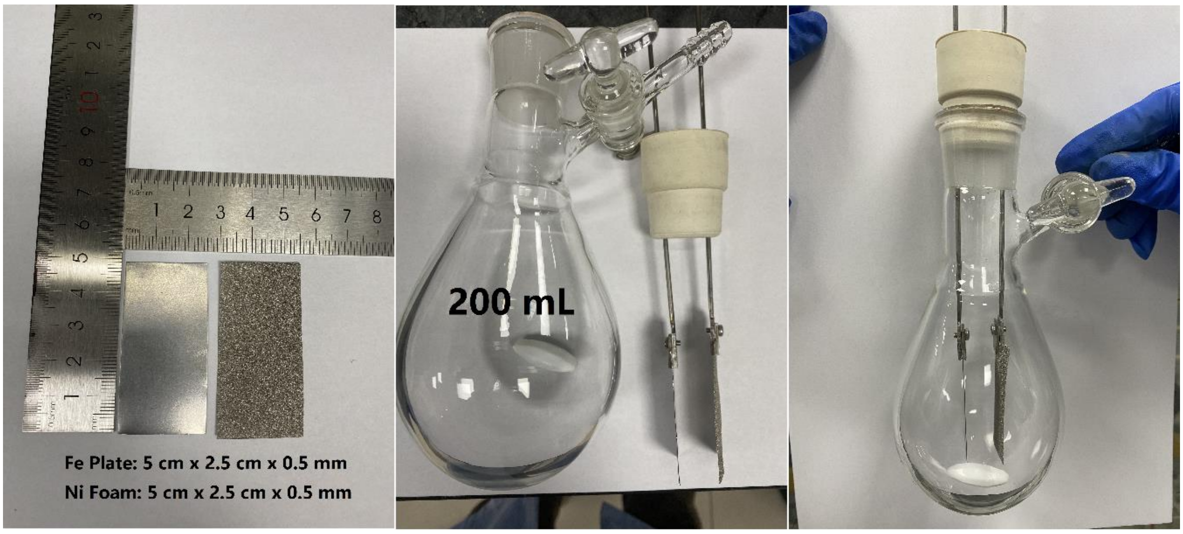

Supplementary Figure 5. Pictures of the reaction setups for the 10 mmol scale.

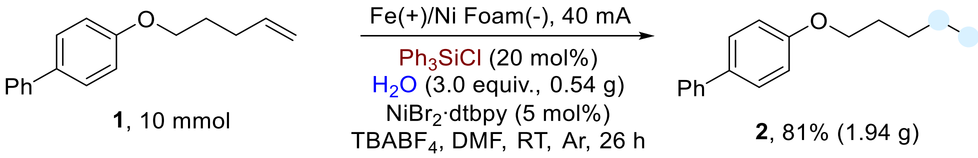

The electrolysis process of 10 mmol scale (supplementary Figure 5) was carried out in an undivided cell with a Fe plate anode (5 cm × 2.5 cm × 0.5 mm) and a Ni Foam cathode (5 cm × 2.5 cm × 0.5 mm). To a 200 mL oven-dried undivided electrochemical cell equipped with a magnetic bar was added unactivated alkene (10 mmol, 1.0 equiv), $NiBr_2$·dtbpy (242 mg, 0.5 mmol, 5 mol%), Ph_3SiCl (600 mg, 2 mmol, 20 mol%), H_2O (540 mg, 30 mmol, 3.0 equiv.) and $^nBu_4NBF_4$ (3.29 g, 10 mmol, 1.0 equiv.) under Ar atmosphere. The electrolysis

12

process was performed at 40 mA of constant current for 26 h at room temperature. After that, the electrodes were washed with EtOAc in an ultrasonic bath. H_2O (20 mL) was added to the organic system, and 20 mL of 1 N HCl aq. was carefully added after the completion of the reaction to help the removal of the excess scrap iron from the reaction system, and the resulting mixture was extracted with EtOAc and the combined organic phase was washed with brine, dried by anhydrous $MgSO_4$, filtered, and concentrated in vacuum. The crude product was purified by column chromatography to furnish the desired product (**2**) in 81% (1.94 g) yield.

Supplementary Table 9: Current efficiencies for this electrochemical hydrogenation process.

Compound	Q (F mol^{-1})	Current efficiencies (%)	Compound	Q (F mol^{-1})	Current efficiencies (%)
**2**	6.2	31.5	**39**	9.9	16.3
**3**	6.2	30.6	**40**	9.9	15.3
**4**	6.2	31.5	**41**	6.2	21.9
**5**	6.2	31.5	**42**	6.2	19.3
**6**	6.2	29.6	**43**	6.2	27.3
**7**	6.2	29.6	**44**	6.2	18.0
**8**	6.2	28.9	**45**	6.2	29.3
**9**	6.2	31.5	**46**	6.2	31.5
**10**	6.2	31.5	**47**	6.2	17.7
**11**	6.2	29.3	**48**	6.2	31.2
**12**	6.2	31.2	**49**	6.2	29.9
**13**	6.2	31.2	**50**	6.2	30.2
**14**	6.2	30.6	**51**	6.2	25.7
**15**	6.2	30.6	**52**	6.2	31.5
**16**	6.2	30.6	**53**	6.2	30.2
**17**	6.2	29.6	**54**	6.2	26.7

**18**	6.2	28.9	**55**	6.2	24.8
**19**	6.2	23.2	**56**	6.2	31.5
**20**	6.2	28.3	**57**	6.2	30.2
**21**	6.2	29.3	**58**	6.2	22.5
**22**	6.2	30.6	**59**	12.4	13.5
**23**	6.2	29.3	**60**	12.4	12.9
**24**	6.2	29.6	**61**	12.4	13.7
**25**	6.2	29.9	**62**	12.4	14.5
**26**	6.2	30.9	**63**	12.4	8.2
**27**	6.2	27.3	**64**	12.4	10.1
**28**	6.2	22.8	**65**	12.4	11.3
**29**	6.2	20.9	**66**	12.4	11.4
**30**	6.2	21.2	**67**	12.4	10.5
**31**	6.2	19.3	**68**	12.4	12.9
**32**	6.2	24.1	**69**	12.4	11.6
**33**	6.2	19.9	**70**	12.4	12.5
**34**	6.2	22.5	**71**	12.4	13.2
**35**	6.2	30.6	**72**	12.4	13.0
**36**	9.9	12.7	**73**	12.4	13.8
**37**	9.9	7.6	**74**	12.4	15.3
**38**	9.9	14.3			

14

1.6 Characterization data of products：

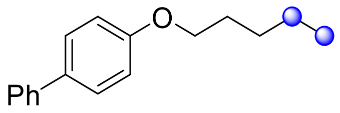

4-(Pentyloxy)-1,1'-biphenyl (2)

The title compound was prepared following the general procedure, purification by column chromatography on silica gel (petroleum ether/EtOAc = 100:1) yielded (70.6 mg, 98%) as a white solid. **^{1}H NMR ($CDCl_3$, 400 MHz)** δ = 7.63 – 7.53 (m, 4H), 7.45 (t, *J* = 7.7 Hz, 2H), 7.34 (t, *J* = 7.4 Hz, 1H), 7.01 (d, *J* = 8.8 Hz, 2H), 4.04 (t, *J* = 6.6 Hz, 2H), 1.86 (p, *J* = 6.6 Hz, 2H), 1.56 – 1.38 (m, 4H), 0.99 (t, *J* = 7.1 Hz, 3H). **^{13}C NMR ($CDCl_3$, 100 MHz)** δ = 158.7, 140.9, 133.5, 128.7, 128.1, 126.7, 126.6, 114.8, 68.1, 29.0, 28.3, 22.5, 14.1. **HRMS (ESI)**: Calcd for $C_{17}H_{21}O^+$ $[M+H]^+$ 241.1587, found 241.1582.

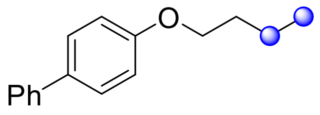

4-Butoxy-1,1'-biphenyl (3)

The title compound was prepared following the general procedure, purification by column chromatography on silica gel (petroleum ether/EtOAc = 100:1) yielded (64.4 mg, 95%) as a white solid. **^{1}H NMR ($CDCl_3$, 400 MHz)** δ = 7.51 (dd, *J* = 15.1, 8.1 Hz, 4H), 7.38 (t, *J* = 7.6 Hz, 2H), 7.27 (t, *J* = 7.3 Hz, 1H), 6.94 (d, *J* = 8.7 Hz, 2H), 3.96 (t, *J* = 6.5 Hz, 2H), 1.77 (dt, *J* = 14.5, 6.6 Hz, 2H), 1.49 (h, *J* = 7.4 Hz, 2H), 0.97 (t, *J* = 7.4 Hz, 3H). **^{13}C NMR ($CDCl_3$, 100 MHz)** δ = 158.8, 140.9, 133.6, 128.8, 128.2, 126.8, 126.7, 114.8, 67.8, 31.5, 19.4, 14.0. Spectroscopic data match those previously reported in the literature.[4]

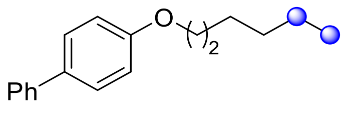

4-(Hexyloxy)-1,1'-biphenyl (4)

The title compound was prepared following the general procedure, purification by column chromatography on silica gel (petroleum ether/EtOAc = 100:1) yielded (74.7 mg, 98%) as a colourless liquid. **^{1}H NMR ($CDCl_3$, 400 MHz)** δ = 7.57 – 7.48 (m, 4H), 7.39 (t, *J* = 7.7 Hz, 2H), 7.28 (t, *J* = 7.4 Hz, 1H), 6.95 (d, *J* = 8.8 Hz, 2H), 3.97 (t, *J* = 6.6 Hz, 2H), 1.83 – 1.74 (m, 2H), 1.47 (p, *J* = 7.2 Hz, 2H), 1.34 (dq, *J* = 7.0, 3.5 Hz, 4H), 0.95 – 0.86 (m, 3H). **^{13}C**

15

NMR ($CDCl_3$, 100 MHz) δ = 158.8, 140.9, 133.6, 128.7, 128.1, 126.7, 126.6, 114.8, 68.1, 31.7, 29.3, 25.8, 22.7, 14.1. Spectroscopic data match those previously reported in the literature.[5]

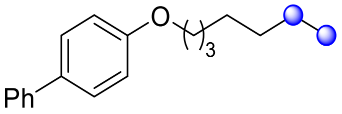

4-(Heptyloxy)-1,1'-biphenyl (5)

The title compound was prepared following the general procedure, purification by column chromatography on silica gel (petroleum ether/EtOAc = 100:1) yielded (78.8 mg, 98%) as a white solid. **^{1}H NMR ($CDCl_3$, 400 MHz)** δ = 7.52 (m, 4H), 7.40 (t, J = 7.6 Hz, 2H), 7.28 (t, J = 7.3 Hz, 1H), 6.96 (d, J = 8.7 Hz, 2H), 3.98 (t, J = 6.6 Hz, 2H), 1.80 (p, J = 6.7 Hz, 2H), 1.51 – 1.41 (m, 2H), 1.41 – 1.27 (m, 6H), 0.90 (t, J = 6.8 Hz, 3H). **^{13}C NMR ($CDCl_3$, 100 MHz)** δ = 158.8, 140.9, 133.5, 128.7, 128.1, 126.7, 126.6, 114.8, 68.1, 31.9, 29.4, 29.1, 26.1, 22.7, 14.2. **HRMS (ESI)**: Calcd for $C_{19}H_{25}O^+$ $[M+H]^+$ 269.1900, found 269.1896.

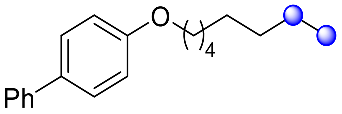

4-(Octyloxy)-1,1'-biphenyl (6)

The title compound was prepared following the general procedure, purification by column chromatography on silica gel (petroleum ether/EtOAc = 100:1) yielded (77.8 mg, 92%) as a white solid. **^{1}H NMR ($CDCl_3$, 400 MHz)** δ = 7.55 (d, J = 8.7 Hz, 4H), 7.45 (t, J = 7.6 Hz, 2H), 7.33 (t, J = 7.3 Hz, 1H), 7.00 (d, J = 8.7 Hz, 2H), 4.02 (t, J = 6.6 Hz, 2H), 1.89 – 1.79 (m, 2H), 1.51 (p, J = 6.9 Hz, 2H), 1.45 – 1.28 (m, 8H), 0.94 (t, J = 6.7 Hz, 3H). **^{13}C NMR ($CDCl_3$, 100 MHz)** δ = 158.9, 141.0, 133.6, 128.8, 128.2, 126.8, 126.7, 114.9, 68.2, 32.0, 29.5, 29.5, 29.4, 26.2, 22.8, 14.3. Spectroscopic data match those previously reported in the literature.[6]

16

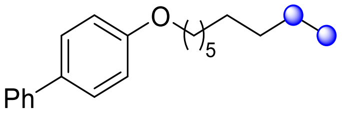

4-(Nonyloxy)-1,1'-biphenyl (7)

The title compound was prepared following the general procedure, purification by column chromatography on silica gel (petroleum ether/EtOAc = 100:1) yielded (81.7 mg, 92%) as a colourless liquid. **^{1}H NMR ($CDCl_3$, 400 MHz)** δ = 7.56 (dd, *J* = 15.0, 7.6 Hz, 4H), 7.43 (t, *J* = 7.6 Hz, 2H), 7.32 (t, *J* = 7.3 Hz, 1H), 6.99 (d, *J* = 8.7 Hz, 2H), 4.01 (t, *J* = 6.6 Hz, 2H), 1.83 (p, *J* = 7.9, 7.2 Hz, 2H), 1.54 – 1.45 (m, 2H), 1.32 (s, 10H), 0.92 (t, *J* = 6.6 Hz, 3H). **^{13}C NMR ($CDCl_3$, 100 MHz)** δ = 158.9, 141.0, 133.6, 128.8, 128.2, 126.8, 126.7, 114.9, 68.2, 32.0, 29.7, 29.6, 29.5, 29.4, 26.2, 22.8, 14.3. **HRMS (ESI)**: Calcd for $C_{21}H_{29}O^+$ $[M+H]^+$ 297.2213, found 297.2213.

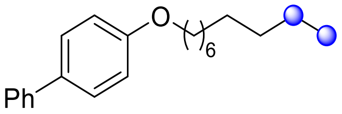

4-(Decyloxy)-1,1'-biphenyl (8)

The title compound was prepared following the general procedure, purification by column chromatography on silica gel (petroleum ether/EtOAc = 100:1) yielded (83.7 mg, 90%) as a white solid. **^{1}H NMR ($CDCl_3$, 400 MHz)** δ = 7.56 (dd, *J* = 14.6, 8.0 Hz, 4H), 7.44 (t, *J* = 7.6 Hz, 2H), 7.32 (t, *J* = 7.3 Hz, 1H), 6.99 (d, *J* = 8.6 Hz, 2H), 4.02 (t, *J* = 6.6 Hz, 2H), 1.83 (p, *J* = 6.7 Hz, 2H), 1.49 (d, *J* = 7.8 Hz, 2H), 1.36 (s, 12H), 0.92 (t, *J* = 6.7 Hz, 3H). **^{13}C NMR ($CDCl_3$, 100 MHz)** δ = 158.7, 141.0, 133.6, 128.8, 128.2, 126.8, 126.7, 114.9, 68.2, 32.1, 29.8, 29.7, 29.6, 29.5, 29.5, 26.2, 22.9, 14.3. Spectroscopic data match those previously reported in the literature.[7]

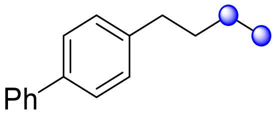

4-Butyl-1,1'-biphenyl (9)

The title compound was prepared following the general procedure, purification by column chromatography on silica gel (petroleum ether/EtOAc = 100:1) yielded (61.7 mg, 98%) as a colorless oil. **^{1}H NMR ($CDCl_3$, 400 MHz)** δ = 7.63 (d, *J* = 7.7 Hz, 2H), 7.55 (d, *J* = 7.7 Hz, 2H), 7.46 (t, *J* = 7.5 Hz, 2H), 7.35 (d, *J* = 7.3 Hz, 1H), 7.29 (d, *J* = 7.7 Hz, 2H), 2.70 (t, *J* = 7.7 Hz, 2H), 1.68 (p, *J* = 7.7 Hz, 2H), 1.44 (h, *J* = 7.3 Hz, 2H), 0.99 (t, *J* = 7.3 Hz, 3H). **^{13}C**

17

NMR ($CDCl_3$, 100 MHz) δ = 142.2, 141.3, 138.7, 129.0, 128.8, 127.1, 127.1, 35.5, 33.8, 22.6, 14.1. Spectroscopic data match those previously reported in the literature.[8]

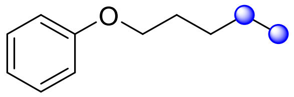

(Pentyloxy)benzene (10)

The title compound was prepared following the general procedure, purification by column chromatography on silica gel (petroleum ether/EtOAc = 100:1) yielded (45.8 mg, 93%) as a colorless oil. **^{1}H NMR ($CDCl_3$, 400 MHz)** δ = 7.34 – 7.28 (m, 2H), 7.01 – 6.89 (m, 3H), 3.99 (t, *J* = 6.6 Hz, 2H), 1.83 (p, *J* = 6.7 Hz, 2H), 1.53 – 1.37 (m, 4H), 0.97 (t, *J* = 7.1 Hz, 3H). **^{13}C NMR ($CDCl_3$, 100 MHz)** δ = 159.3, 129.5, 120.6, 114.6, 68.0, 29.2, 28.4, 22.6, 14.2. Spectroscopic data match those previously reported in the literature.[9]

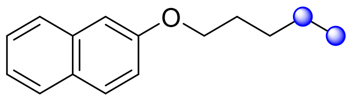

2-(Pentyloxy)naphthalene (11)

The title compound was prepared following the general procedure, purification by column chromatography on silica gel (petroleum ether/EtOAc = 100:1) yielded (58.4 mg, 91%) as a colorless oil. **^{1}H NMR ($CDCl_3$, 400 MHz)** δ = 7.85 – 7.71 (m, 3H), 7.46 (t, *J* = 7.5 Hz, 1H), 7.36 (t, *J* = 7.4 Hz, 1H), 7.23 – 7.12 (m, 2H), 4.10 (t, *J* = 6.6 Hz, 2H), 1.89 (p, *J* = 6.7 Hz, 2H), 1.49 (ddq, *J* = 30.2, 15.9, 8.5, 7.3 Hz, 4H), 1.00 (t, *J* = 7.1 Hz, 3H). **^{13}C NMR ($CDCl_3$, 100 MHz)** δ = 157.2, 134.8, 129.4, 129.0, 127.8, 126.8, 126.4, 123.6, 119.2, 106.7, 68.1, 29.1, 28.4, 22.7, 14.2. Spectroscopic data match those previously reported in the literature.[10]

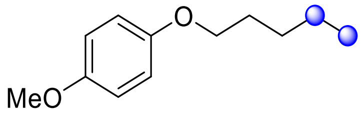

1-Methoxy-4-(pentyloxy)benzene (12)

The title compound was prepared following the general procedure, purification by column chromatography on silica gel (petroleum ether/EtOAc = 80:1) yielded (56.4 mg, 97%) as a colorless oil. **^{1}H NMR ($CDCl_3$, 400 MHz)** δ = 6.85 (m, 4H), 3.91 (t, *J* = 6.6 Hz, 2H), 3.78 (s, 3H), 1.83 – 1.73 (m, 2H), 1.50 – 1.34 (m, 4H), 0.95 (t, *J* = 7.1 Hz, 3H). **^{13}C NMR ($CDCl_3$, 100 MHz)** δ = 153.8, 153.4, 115.5, 114.7, 68.7, 55.8, 29.2, 28.4, 22.6, 14.2. Spectroscopic data match those previously reported in the literature.[11]

18

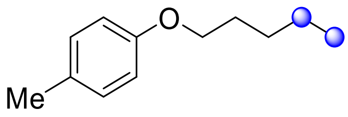

1-Methyl-4-(pentyloxy)benzene (13)

The title compound was prepared following the general procedure, purification by column chromatography on silica gel (petroleum ether/EtOAc = 100:1) yielded (50.2 mg, 94%) as a colorless oil. **^{1}H NMR (CDCl$_3$, 400 MHz)** δ = 7.08 (d, J = 8.4 Hz, 2H), 6.81 (d, J = 8.6 Hz, 2H), 3.93 (t, J = 6.6 Hz, 2H), 2.29 (s, 3H), 1.78 (p, J = 6.7 Hz, 2H), 1.49 – 1.33 (m, 4H), 0.94 (t, J = 7.1 Hz, 3H). **^{13}C NMR (CDCl$_3$, 100 MHz)** δ = 157.2, 130.0, 129.8, 114.5, 68.2, 29.2, 28.4, 22.6, 20.6, 14.2. Spectroscopic data match those previously reported in the literature.[12]

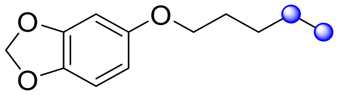

5-(Pentyloxy)benzo[*d*][1,3]dioxole (14)

The title compound was prepared following the general procedure, purification by column chromatography on silica gel (petroleum ether/EtOAc = 50:1) yielded (59.9 mg, 96%) as a colorless oil. **^{1}H NMR (CDCl$_3$, 400 MHz)** δ = 6.70 (d, J = 8.5 Hz, 1H), 6.49 (d, J = 2.5 Hz, 1H), 6.32 (dd, J = 8.5, 2.5 Hz, 1H), 5.90 (s, 2H), 3.87 (t, J = 6.6 Hz, 2H), 1.81 – 1.70 (m, 2H), 1.48 – 1.33 (m, 4H), 0.93 (t, J = 7.1 Hz, 3H). **^{13}C NMR (CDCl$_3$, 100 MHz)** δ = 154.8, 148.3, 141.6, 108.1, 105.8, 101.2, 98.2, 69.1, 29.2, 28.4, 22.6, 14.2. Spectroscopic data match those previously reported in the literature.[13]

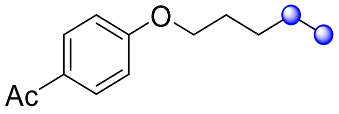

1-(4-(Pentyloxy)phenyl)ethan-1-one (15)

The title compound was prepared following the general procedure, purification by column chromatography on silica gel (petroleum ether/EtOAc = 20:1) yielded (58.7 mg, 95%) as a colorless oil. **^{1}H NMR (CDCl$_3$, 400 MHz)** δ = 7.92 (d, J = 8.9 Hz, 2H), 6.91 (d, J = 8.9 Hz, 2H), 4.01 (t, J = 6.6 Hz, 2H), 1.81 (p, J = 6.6 Hz, 2H), 1.51 – 1.34 (m, 4H), 0.93 (t, J = 7.1 Hz, 3H). **^{13}C NMR (CDCl$_3$, 100 MHz)** δ = 196.9, 163.3, 130.7, 130.2, 114.3, 68.4, 28.9, 28.3, 26.5, 22.6, 14.2. Spectroscopic data match those previously reported in the literature.[14]

19

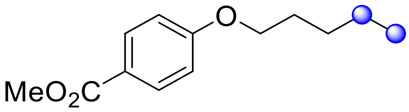

Methyl 4-(pentyloxy)benzoate (16)

The title compound was prepared following the general procedure, purification by column chromatography on silica gel (petroleum ether/EtOAc = 20:1) yielded (63.3 mg, 95%) as a colorless oil. **^{1}H NMR ($CDCl_3$, 400 MHz)** δ = 7.98 (d, J = 8.4 Hz, 2H), 6.90 (d, J = 8.3 Hz, 2H), 4.00 (t, J = 6.4 Hz, 2H), 3.88 (s, 3H), 1.86 – 1.74 (m, 2H), 1.41 (dt, J = 14.2, 7.3 Hz, 4H), 0.94 (t, J = 6.4 Hz, 3H). **^{13}C NMR ($CDCl_3$, 100 MHz)** δ = 167.1, 163.1, 131.7, 122.5, 114.2, 68.3, 52.0, 29.0, 28.3, 22.6, 14.1. Spectroscopic data match those previously reported in the literature.[11]

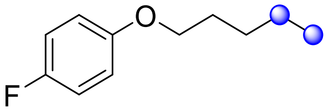

1-Fluoro-4-(pentyloxy)benzene (17)

The title compound was prepared following the general procedure, purification by column chromatography on silica gel (petroleum ether/EtOAc = 100:1) yielded (50.2 mg, 92%) as a colorless oil. **^{1}H NMR ($CDCl_3$, 400 MHz)** δ = 7.00 – 6.91 (m, 2H), 6.86 – 6.77 (m, 2H), 3.91 (t, J = 6.6 Hz, 2H), 1.77 (p, J = 6.6 Hz, 2H), 1.48 – 1.33 (m, 4H), 0.93 (t, J = 7.1 Hz, 3H). **^{13}C NMR ($CDCl_3$, 100 MHz)** δ = 157.1 (d, J = 237.6 Hz), 155.2 (d, J = 2.0 Hz), 115.7 (d, J = 22.9 Hz), 115.4 (d, J = 8.0 Hz), 68.6, 29.0, 28.2, 22.5, 14.0. **^{19}F NMR ($CDCl_3$, 375 MHz)** δ = -124.53. **HRMS (ESI)**: Calcd for $C_{11}H_{16}FO^{+}$ $[M+H]^{+}$ 183.1180, found 183.1175.

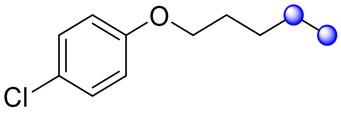

1-Chloro-4-(pentyloxy)benzene (18)

The title compound was prepared following the general procedure, purification by column chromatography on silica gel (petroleum ether/EtOAc = 100:1) yielded (53.5 mg, 90%) as a colorless oil. **^{1}H NMR ($CDCl_3$, 400 MHz)** δ = 7.22 (d, J = 9.0 Hz, 2H), 6.82 (d, J = 9.0 Hz, 2H), 3.92 (t, J = 6.6 Hz, 2H), 1.78 (p, J = 6.6 Hz, 2H), 1.40 (dd, J = 12.0, 6.4 Hz, 4H), 0.94 (t, J = 7.1 Hz, 3H). **^{13}C NMR ($CDCl_3$, 100 MHz)** δ = 157.9, 129.4, 125.4, 115.9, 68.4, 29.1, 28.3, 22.6, 14.2. Spectroscopic data match those previously reported in the literature.[11]

20

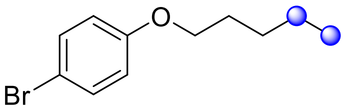

1-Bromo-4-(pentyloxy)benzene (19)

The title compound was prepared following the general procedure, purification by column chromatography on silica gel (petroleum ether/EtOAc = 200:1) yielded (52.3 mg, 72%) as a colorless oil. **^{1}H NMR ($CDCl_3$, 400 MHz)** δ = 7.36 (d, *J* = 9.0 Hz, 2H), 6.77 (d, *J* = 9.0 Hz, 2H), 3.91 (t, *J* = 6.6 Hz, 2H), 1.77 (p, *J* = 6.6 Hz, 2H), 1.48 – 1.31 (m, 4H), 0.93 (t, *J* = 7.1 Hz, 3H). **^{13}C NMR ($CDCl_3$, 100 MHz)** δ = 158.4, 132.3, 116.4, 112.7, 68.4, 29.0, 28.3, 22.6, 14.2. Spectroscopic data match those previously reported in the literature.[11]

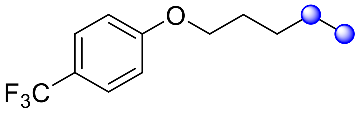

1-(Pentyloxy)-4-(trifluoromethyl)benzene (20)

The title compound was prepared following the general procedure, purification by column chromatography on silica gel (petroleum ether/EtOAc = 100:1) yielded (61.2 mg, 88%) as a colorless oil. **^{1}H NMR ($CDCl_3$, 400 MHz)** δ = 7.53 (d, *J* = 8.5 Hz, 2H), 6.95 (d, *J* = 8.5 Hz, 2H), 3.99 (t, *J* = 6.5 Hz, 2H), 1.81 (p, *J* = 6.6 Hz, 2H), 1.42 (tp, *J* = 15.4, 8.0, 7.2 Hz, 4H), 0.94 (t, *J* = 7.0 Hz, 3H). **^{13}C NMR ($CDCl_3$, 100 MHz)** δ = 161.6, 126.8 (q, *J* = 3.8), 124.5 (7, *J* = 270.8), 122.6 (q, *J* = 32.5), 157.1 (d, *J* = 237.6), 114.4, 68.2, 28.8, 28.1, 22.4, 14.0. **^{19}F NMR ($CDCl_3$, 375 MHz)** δ = -61.4. Spectroscopic data match those previously reported in the literature.[13]

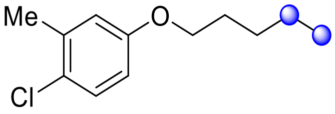

1-Chloro-2-methyl-4-(pentyloxy)benzene (21)

The title compound was prepared following the general procedure, purification by column chromatography on silica gel (petroleum ether/EtOAc = 100:1) yielded (57.9 mg, 91%) as a colorless oil. **^{1}H NMR ($CDCl_3$, 400 MHz)** δ = 7.21 (d, *J* = 8.7 Hz, 1H), 6.77 (s, 1H), 6.67 (d, *J* = 6.0 Hz, 1H), 3.91 (t, *J* = 6.6 Hz, 2H), 2.34 (s, 3H), 1.77 (p, *J* = 6.7 Hz, 2H), 1.41 (tp, *J* = 14.8, 7.6, 6.9 Hz, 4H), 0.94 (t, *J* = 6.9 Hz, 3H). **^{13}C NMR ($CDCl_3$, 100 MHz)** δ = 157.8, 137.0, 129.7, 125.7, 117.2, 113.2, 68.4, 29.1, 28.3, 22.6, 20.5, 14.2. **HRMS (ESI)**: Calcd for $C_{12}H_{18}ClO_5^+$ [M+H]$^+$ 213.1041, found 213.1041.

21

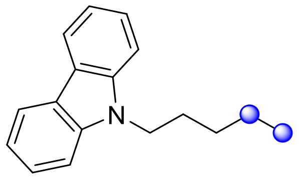

9-Pentyl-9H-carbazole (22)

The title compound was prepared following the general procedure, purification by column chromatography on silica gel (petroleum ether/EtOAc = 100:1) yielded (67.5 mg, 95%) as a white solid. **^{1}H NMR ($CDCl_3$, 400 MHz)** δ = 8.08 (d, J = 7.7 Hz, 2H), 7.48 – 7.41 (m, 2H), 7.37 (d, J = 8.2 Hz, 2H), 7.25 – 7.15 (m, 2H), 4.25 (t, J = 7.3 Hz, 2H), 1.84 (p, J = 7.4 Hz, 2H), 1.34 (dt, J = 7.3, 3.9 Hz, 4H), 0.91 – 0.80 (m, 3H). **^{13}C NMR ($CDCl_3$, 100 MHz)** δ = 140.5, 125.7, 122.9, 120.5, 118.8, 108.8, 43.2, 29.6, 28.8, 22.6, 14.1. Spectroscopic data match those previously reported in the literature.[15]

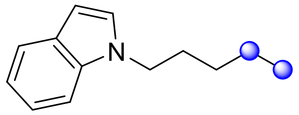

1-Pentyl-1H-indole (23)

The title compound was prepared following the general procedure, purification by column chromatography on silica gel (petroleum ether/EtOAc = 100:1) yielded (50.1 mg, 91%) as a yellow oil. **^{1}H NMR ($CDCl_3$, 400 MHz)** δ = 7.62 (d, J = 7.9 Hz, 1H), 7.33 (d, J = 8.2 Hz, 1H), 7.19 (t, J = 8.1 Hz, 1H), 7.12 – 7.05 (m, 2H), 6.47 (d, J = 3.6 Hz, 1H), 4.08 (t, J = 7.1 Hz, 2H), 1.82 (p, J = 7.3 Hz, 2H), 1.37 – 1.25 (m, 4H), 0.87 (t, J = 7.0 Hz, 3H). **^{13}C NMR ($CDCl_3$, 100 MHz)** δ = 136.0, 128.6, 127.9, 121.3, 121.0, 119.2, 109.4, 100.9, 46.5, 30.0, 29.2, 22.4, 14.0. Spectroscopic data match those previously reported in the literature.[16]

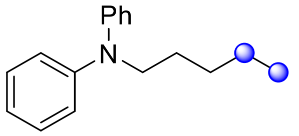

***N*-pentyl-*N*-phenylaniline (24)**

The title compound was prepared following the general procedure, purification by column chromatography on silica gel (petroleum ether/EtOAc = 100:1) yielded (66.0 mg, 92%) as a colorless oil. **^{1}H NMR ($CDCl_3$, 400 MHz)** δ = 7.32 (t, J = 7.9 Hz, 4H), 7.13 – 6.92 (m, 6H), 3.80 – 3.69 (m, 2H), 1.79 – 1.66 (m, 2H), 1.45 – 1.31 (m, 4H), 0.95 (t, J = 6.7 Hz, 3H). **^{13}C NMR ($CDCl_3$, 100 MHz)** δ = 148.1, 129.3, 121.0, 120.9, 52.4, 29.3, 27.2, 22.6, 14.2. **HRMS**

22

(ESI): Calcd for $C_{17}H_{22}N^+$ $[M+H]^+$ 240.1747, found 240.1744.

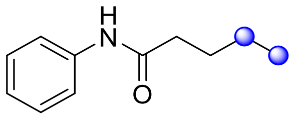

***N*-phenylpentanamide (25)**

The title compound was prepared following the general procedure, purification by column chromatography on silica gel (petroleum ether/EtOAc = 4:1) yielded (52.0 mg, 98%) as a white solid. **^{1}H NMR ($CDCl_3$, 400 MHz)** δ = 7.61 (s, 1H), 7.48 (d, *J* = 7.7 Hz, 2H), 7.24 (t, *J* = 7.6 Hz, 2H), 7.03 (t, *J* = 7.1 Hz, 1H), 2.30 (t, *J* = 7.5 Hz, 2H), 1.65 (p, *J* = 7.5 Hz, 2H), 1.34 (h, *J* = 7.1 Hz, 2H), 0.88 (t, *J* = 7.3 Hz, 3H). **^{13}C NMR ($CDCl_3$, 100 MHz)** δ = 171.8, 138.1, 128.9, 124.1, 120.0, 37.5, 27.8, 22.4, 13.8. Spectroscopic data match those previously reported in the literature.[17

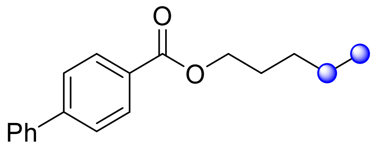

Pentyl [1,1'-biphenyl]-4-carboxylate (26)

The title compound was prepared following the general procedure, purification by column chromatography on silica gel (petroleum ether/EtOAc = 50:1) yielded (77.2 mg, 96%) as a colorless oil. **^{1}H NMR ($CDCl_3$, 400 MHz)** δ = 8.13 (d, *J* = 8.4 Hz, 2H), 7.65 (m, 4H), 7.48 (t, *J* = 7.4 Hz, 2H), 7.40 (t, *J* = 7.3 Hz, 1H), 4.35 (t, *J* = 6.7 Hz, 2H), 1.81 (p, *J* = 6.8 Hz, 2H), 1.53 – 1.37 (m, 4H), 0.96 (t, *J* = 7.1 Hz, 3H). **^{13}C NMR ($CDCl_3$, 100 MHz)** δ = 166.6, 145.5, 140.1, 130.1, 129.3, 128.9, 128.1, 127.3, 127.0, 65.2, 28.5, 28.3, 22.4, 14.1. **HRMS (ESI)**: Calcd for $C_{18}H_{21}O_2^+$ $[M+H]^+$ 269.1536, found 269.1534.

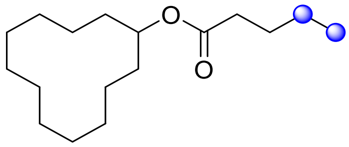

Cyclododecyl pentanoate (27)

The title compound was prepared following the general procedure, purification by column chromatography on silica gel (petroleum ether/EtOAc = 80:1) yielded (68.3 mg, 85%) as a colorless oil. **^{1}H NMR ($CDCl_3$, 400 MHz)** δ = 5.01 (ddd, *J* = 11.9, 7.1, 4.7 Hz, 1H), 2.26 (t, *J* = 7.5 Hz, 2H), 1.69 (dq, *J* = 13.0, 6.4 Hz, 2H), 1.63 – 1.55 (m, 2H), 1.52 – 1.28 (m, 22H),

23

0.91 (t, J = 7.3 Hz, 3H). **^{13}C NMR ($CDCl_3$, 100 MHz)** δ = 173.7, 72.0, 34.6, 29.3, 27.3, 24.2, 24.0, 23.5, 23.3, 22.4, 21.1, 13.9. **HRMS (ESI)**: Calcd for $C_{17}H_{33}O_2^+$ [M+H]$^+$ 269.2475, found 269.2474.

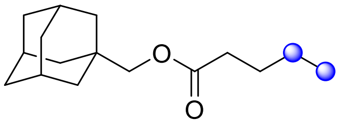

((3r,5r,7r)-Adamantan-1-yl)methyl pentanoate (28)

The title compound was prepared following the general procedure, purification by column chromatography on silica gel (petroleum ether/EtOAc = 80:1) yielded (53.3 mg, 71%) as a colorless oil. **^{1}H NMR ($CDCl_3$, 400 MHz)** δ = 3.65 (s, 2H), 2.30 (t, J = 7.5 Hz, 2H), 1.96 (s, 3H), 1.71 (d, J = 12.2 Hz, 3H), 1.61 (dd, J = 16.0, 8.7 Hz, 5H), 1.52 (s, 6H), 1.34 (h, J = 7.4 Hz, 2H), 0.91 (t, J = 7.3 Hz, 3H). **^{13}C NMR ($CDCl_3$, 100 MHz)** δ = 174.2, 73.9, 39.4, 37.1, 34.2, 33.3, 28.2, 27.3, 22.4, 13.8. **HRMS (ESI)**: Calcd for $C_{16}H_{27}O_2^+$ [M+H]$^+$ 251.2006, found 251.2000.

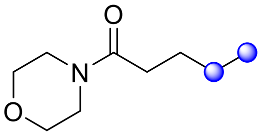

1-Morpholinopentan-1-one (29)

The title compound was prepared following the general procedure, purification by column chromatography on silica gel (petroleum ether/EtOAc = 5:1 to 3:1) yielded (33.3 mg, 65%) as a colorless oil. **^{1}H NMR ($CDCl_3$, 400 MHz)** δ = 3.68 – 3.47 (m, 6H), 3.46 – 3.34 (m, 2H), 2.29 – 2.18 (m, 2H), 1.54 (p, J = 7.6 Hz, 2H), 1.29 (h, J = 7.3 Hz, 2H), 0.85 (t, J = 7.3 Hz, 3H). **^{13}C NMR ($CDCl_3$, 100 MHz)** δ = 171.8, 66.9, 66.7, 46.0, 41.8, 32.8, 27.3, 22.5, 13.8. Spectroscopic data match those previously reported in the literature.[18]

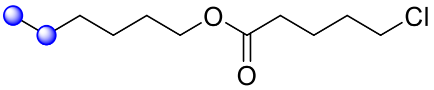

Hexyl 5-chloropentanoate (30)

The title compound was prepared following the general procedure, purification by column chromatography on silica gel (petroleum ether/EtOAc = 100:1) yielded (43.6 mg, 66%) as a colorless oil. **^{1}H NMR ($CDCl_3$, 400 MHz)** δ = 4.05 (t, J = 6.7 Hz, 2H), 3.53 (t, J = 6.1 Hz, 2H), 2.32 (t, J = 6.9 Hz, 2H), 1.84 – 1.72 (m, 4H), 1.60 (dt, J = 14.2, 6.4 Hz, 2H), 1.30 (dd,

24

J = 9.0, 4.6 Hz, 6H), 0.87 (t, J = 6.8 Hz, 3H). **^{13}C NMR ($CDCl_3$, 100 MHz)** δ = 173.2, 64.6, 44.4, 33.4, 31.9, 31.4, 28.6, 25.6, 22.5, 22.3, 14.0. **HRMS (ESI)**: Calcd for $C_{11}H_{22}ClO_2^+$ $[M+H]^+$ 221.1303, found 221.1300.

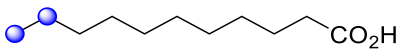

Undecanoic acid (31)

The title compound was prepared following the general procedure, purification by column chromatography on silica gel (petroleum ether/EtOAc = 10:1 to 5:1) yielded (33.5 mg, 60%) as a colorless oil. **^{1}H NMR ($CDCl_3$, 400 MHz)** δ = 2.35 (t, J = 7.5 Hz, 2H), 1.63 (p, J = 8.1, 7.6 Hz, 2H), 1.28 (d, J = 14.9 Hz, 15H), 0.88 (t, J = 6.6 Hz, 3H). **^{13}C NMR ($CDCl_3$, 100 MHz)** δ = 180.5, 34.2, 32.0, 29.7, 29.6, 29.5, 29.4, 29.2, 24.8, 22.8, 14.3. Spectroscopic data match those previously reported in the literature.[19]

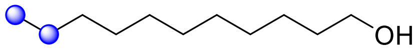

Undecan-1-ol (32)

The title compound was prepared following the general procedure, purification by column chromatography on silica gel (petroleum ether/EtOAc = 100:1 to 50:1) yielded (38.7 mg, 75%) as a colorless oil. **^{1}H NMR ($CDCl_3$, 400 MHz)** δ = 3.62 (t, J = 6.7 Hz, 2H), 1.55 (p, J = 6.7 Hz, 2H), 1.48 (s, 1H), 1.39 – 1.19 (m, 16H), 0.87 (t, J = 6.8 Hz, 3H). **^{13}C NMR ($CDCl_3$, 100 MHz)** δ = 63.2, 32.9, 32.0, 29.8, 29.6, 29.5, 25.9, 22.8, 14.3. Spectroscopic data match those previously reported in the literature.[20]

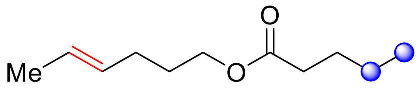

(*E*)-Hex-4-en-1-yl pentanoate (33)

The title compound was prepared following the general procedure, purification by column chromatography on silica gel (petroleum ether/EtOAc = 100:1) yielded (34.2 mg, 62%) as a colorless oil. **^{1}H NMR ($CDCl_3$, 400 MHz)** δ = 5.51 – 5.32 (m, 2H), 4.05 (t, J = 6.7 Hz, 2H), 2.29 (t, J = 7.5 Hz, 2H), 2.04 (q, J = 7.1, 6.5 Hz, 2H), 1.63 (tq, J = 15.1, 7.2 Hz, 7H), 1.34 (h, J = 7.4 Hz, 2H), 0.91 (t, J = 7.3 Hz, 3H). **^{13}C NMR ($CDCl_3$, 100 MHz)** δ = 174.0, 130.0, 125.8, 63.7, 34.1, 28.9, 28.5, 27.1, 22.3, 17.9, 13.7. **HRMS (ESI)**: Calcd for $C_{11}H_{21}O_2^+$ $[M+H]^+$ 185.1536, found 185.1539.

25

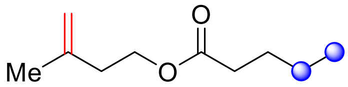

3-Methylbut-3-en-1-yl pentanoate (34)

The title compound was prepared following the general procedure, purification by column chromatography on silica gel (petroleum ether/EtOAc = 100:1) yielded (35.7 mg, 70%) as a colorless oil. **^{1}H NMR (CDCl$_3$, 400 MHz)** δ = 4.74 (d, *J* = 26.6 Hz, 2H), 4.16 (t, *J* = 6.9 Hz, 2H), 2.29 (dt, *J* = 15.1, 7.2 Hz, 4H), 1.73 (s, 3H), 1.58 (p, *J* = 7.5 Hz, 2H), 1.32 (dq, *J* = 14.6, 7.4 Hz, 2H), 0.89 (t, *J* = 7.3 Hz, 3H). **^{13}C NMR (CDCl$_3$, 100 MHz)** δ = 173.8, 141.7, 112.2, 62.4, 36.7, 34.0, 27.0, 22.4, 22.2, 13.7. **HRMS (ESI)**: Calcd for $C_{10}H_{19}O_2^+$ $[M+H]^+$ 171.1380, found 171.1377.

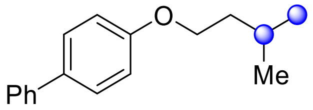

4-(Isopentyloxy)-1,1'-biphenyl (35)

The title compound was prepared following the general procedure, purification by column chromatography on silica gel (petroleum ether/EtOAc = 100:1) yielded (68.4 mg, 95%) as a white solid. **^{1}H NMR (CDCl$_3$, 400 MHz)** δ = 7.61 (m, 4H), 7.48 (t, *J* = 7.7 Hz, 2H), 7.36 (t, *J* = 8.0 Hz, 1H), 7.04 (d, *J* = 8.8 Hz, 2H), 4.09 (t, *J* = 6.7 Hz, 2H), 1.94 (dp, *J* = 13.3, 6.6 Hz, 1H), 1.78 (q, *J* = 6.7 Hz, 2H), 1.05 (d, *J* = 6.6 Hz, 6H). **^{13}C NMR (CDCl$_3$, 100 MHz)** δ = 158.8, 140.9, 133.6, 128.8, 128.2, 126.8, 126.6, 114.8, 66.5, 38.1, 25.1, 22.7. **HRMS (ESI)**: Calcd for $C_{17}H_{21}O^+$ $[M+H]^+$ 241.1587, found 241.1586.

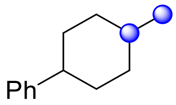

dr = 2 : 1

(4-Methylcyclohexyl)benzene (38)

The title compound was prepared following the general procedure, purification by column chromatography on silica gel (petroleum ether) yielded (37.1 mg, 71%) as a colorless oil. **^{1}H NMR (CDCl$_3$, 400 MHz)** δ = 7.39 – 7.19 (m, 5H), 2.66 – 2.44 (m, 1H), 2.06 – 1.65 (m, 6H), 1.65 – 1.47 (m, 3H), 1.15 (td, *J* = 12.4, 12.0, 3.2 Hz, 1H), 1.04 (m, 3H). Spectroscopic data match those previously reported in the literature.[21]

26

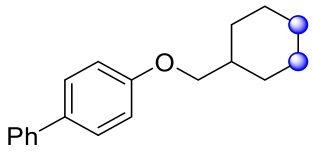

4-(Cyclohexylmethoxy)-1,1'-biphenyl (39)

The title compound was prepared following the general procedure, purification by column chromatography on silica gel (petroleum ether/EtOAc = 100:1) yielded (64.6 mg, 81%) as a white solid. **^{1}H NMR ($CDCl_3$, 400 MHz)** δ = 7.56 (dd, *J* = 16.0, 8.6 Hz, 4H), 7.44 (t, *J* = 7.7 Hz, 2H), 7.33 (t, *J* = 7.3 Hz, 1H), 6.99 (d, *J* = 8.7 Hz, 2H), 3.82 (d, *J* = 6.3 Hz, 2H), 1.92 (d, *J* = 13.2 Hz, 2H), 1.88 – 1.68 (m, 4H), 1.41 – 1.20 (m, 3H), 1.11 (td, *J* = 12.0, 2.8 Hz, 2H). **^{13}C NMR ($CDCl_3$, 100 MHz)** δ = 158.9, 141.0, 133.5, 128.7, 128.1, 126.8, 126.6, 114.8, 73.6, 37.8, 30.0, 26.6, 25.9. Spectroscopic data match those previously reported in the literature.[22]

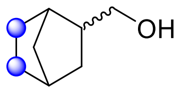

dr = 3 : 1
from **S40**, ratio = 1.2 : 1

Bicyclo[2.2.1]heptan-2-ylmethanol (40)

The title compound was prepared following the general procedure, purification by column chromatography on silica gel (petroleum ether/EtOAc = 100:1 to 50:1) yielded (28.7 mg, 76%) as a colorless oil. **^{1}H NMR ($CDCl_3$, 400 MHz)** δ = 3.67 – 3.29 (m, 2H), 2.33 – 2.00 (m, 3H), 1.76 – 1.45 (m, 4H), 1.43 – 0.94 (m, 5H), 0.62 (ddd, *J* = 12.2, 5.2, 2.2 Hz, 1H). Spectroscopic data match those previously reported in the literature.[23]

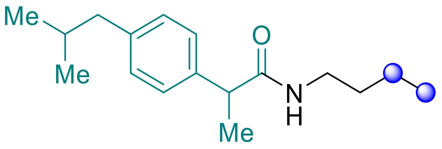

***N*-Butyl-2-(4-isobutylphenyl)propanamide (45)**

The title compound was prepared following the general procedure, purification by column chromatography on silica gel (petroleum ether/EtOAc = 4:1) yielded (71.3 mg, 91%) as a white solid. **^{1}H NMR ($CDCl_3$, 400 MHz)** δ = 7.18 (d, *J* = 8.0 Hz, 2H), 7.11 (d, *J* = 8.0 Hz, 2H), 5.34 (s, 1H), 3.51 (q, *J* = 7.2 Hz, 1H), 3.17 (q, *J* = 6.9 Hz, 2H), 2.45 (d, *J* = 7.2 Hz, 2H), 1.84 (dp, *J* = 13.6, 6.8 Hz, 1H), 1.50 (d, *J* = 7.2 Hz, 3H), 1.37 (p, *J* = 7.1 Hz, 2H), 1.21 (dq,

27

J = 14.4, 7.2 Hz, 2H), 0.92 – 0.80 (m, 9H). **^{13}C NMR (CDCl$_3$, 100 MHz)** δ = 174.4, 140.7, 138.7, 129.6, 127.4, 46.8, 45.0, 39.3, 31.6, 30.2, 22.7, 19.9, 18.5, 13.7. **HRMS (ESI)**: Calcd for $C_{17}H_{28}NO^+$ $[M+H]^+$ 262.2165, found 262.2160.

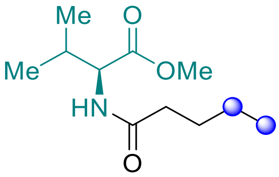

Methyl pentanoyl-(L)-valinate (46)

The title compound was prepared following the general procedure, purification by column chromatography on silica gel (petroleum ether/EtOAc = 10:1 to 3:1) yielded (63.2 mg, 98%) as a colorless oil. **^{1}H NMR (CDCl$_3$, 400 MHz)** δ = 6.03 (d, J = 6.8 Hz, 1H), 4.61 – 4.47 (m, 1H), 3.70 (s, 3H), 2.21 (t, J = 7.4 Hz, 2H), 2.12 (dt, J = 12.2, 6.1 Hz, 1H), 1.59 (p, J = 7.3 Hz, 2H), 1.32 (h, J = 8.3, 7.7 Hz, 2H), 0.88 (dt, J = 9.8, 5.7 Hz, 9H). **^{13}C NMR (CDCl$_3$, 100 MHz)** δ = 173.1, 172.8, 56.8, 52.1, 36.4, 31.3, 27.8, 22.3, 18.9, 17.8, 13.7. **HRMS (ESI)**: Calcd for $C_{11}H_{22}NO_3^+$ $[M+H]^+$ 216.1594, found 216.1590.

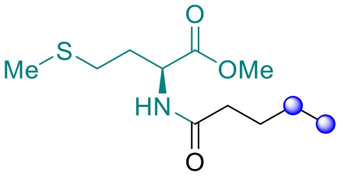

Methyl pentanoyl-(L)-methioninate (47)

The title compound was prepared following the general procedure, purification by column chromatography on silica gel (petroleum ether/EtOAc = 10:1 to 3:1) yielded (40.8 mg, 55%) as a colorless oil. **^{1}H NMR (CDCl$_3$, 400 MHz)** δ = 6.25 (d, J = 7.5 Hz, 1H), 4.72 (td, J = 7.5, 5.2 Hz, 1H), 3.75 (s, 3H), 2.51 (td, J = 8.0, 2.7 Hz, 2H), 2.27 – 2.20 (m, 2H), 2.20 – 2.11 (m, 1H), 2.09 (s, 3H), 1.96 (dt, J = 14.1, 7.0 Hz, 1H), 1.61 (q, J = 7.6 Hz, 2H), 1.35 (dq, J = 14.7, 7.3 Hz, 2H), 0.91 (t, J = 7.3 Hz, 3H). **^{13}C NMR (CDCl$_3$, 100 MHz)** δ = 173.0, 172.6, 52.5, 51.4, 36.3, 31.7, 30.0, 27.6, 22.3, 15.5, 13.8. **HRMS (ESI)**: Calcd for $C_{11}H_{22}NO_3S^+$ $[M+H]^+$ 248.1315, found 248.1312.

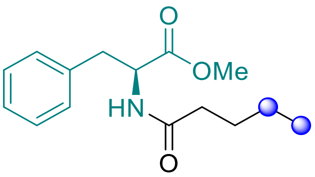

28

Methyl pentanoyl-(L)-phenylalaninate (48)

The title compound was prepared following the general procedure, purification by column chromatography on silica gel (petroleum ether/EtOAc = 10:1 to 3:1) yielded (76.5 mg, 97%) as a colorless oil. **^{1}H NMR ($CDCl_3$, 400 MHz)** δ = 7.32 – 7.22 (m, 3H), 7.09 (d, J = 6.7 Hz, 2H), 5.87 (d, J = 7.0 Hz, 1H), 4.97 – 4.84 (m, 1H), 3.73 (s, 3H), 3.12 (qd, J = 13.9, 5.8 Hz, 2H), 2.17 (t, J = 7.6 Hz, 2H), 1.57 (p, J = 7.5 Hz, 2H), 1.30 (dq, J = 14.8, 7.4 Hz, 2H), 0.89 (t, J = 7.3 Hz, 3H). **^{13}C NMR ($CDCl_3$, 100 MHz)** δ = 172.6, 172.2, 135.9, 129.3, 128.6, 127.1, 52.9, 52.3, 37.9, 36.3, 27.6, 22.3, 13.8. **HRMS (ESI)**: Calcd for $C_{15}H_{22}NO_3^+$ [M+H]$^+$ 264.1594, found 264.1578.

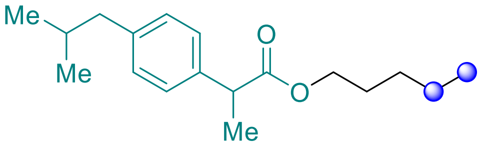

Pentyl 2-(4-isobutylphenyl)propanoate (49)

The title compound was prepared following the general procedure, purification by column chromatography on silica gel (petroleum ether/EtOAc = 100:1 to 50:1) yielded (77.0 mg, 93%) as a colorless oil. **^{1}H NMR ($CDCl_3$, 400 MHz)** δ = 7.20 (d, J = 8.0 Hz, 2H), 7.08 (d, J = 8.1 Hz, 2H), 4.05 (t, J = 6.6 Hz, 2H), 3.68 (q, J = 7.1 Hz, 1H), 2.44 (d, J = 7.2 Hz, 2H), 1.84 (dp, J = 13.6, 6.8 Hz, 1H), 1.56 (dt, J = 13.8, 6.8 Hz, 2H), 1.48 (d, J = 7.2 Hz, 3H), 1.24 (ddt, J = 13.8, 8.0, 5.0 Hz, 4H), 0.93 – 0.80 (m, 9H). **^{13}C NMR ($CDCl_3$, 100 MHz)** δ = 174.8, 140.4, 137.9, 129.3, 127.2, 64.8, 45.2, 45.1, 30.2, 28.2, 27.9, 22.4, 22.2, 18.5, 13.9. **HRMS (ESI)**: Calcd for $C_{18}H_{29}O_2^+$ [M+H]$^+$ 277.2162, found 277.2168.

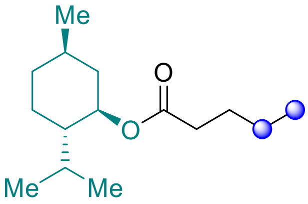

(1R,2S,5R)-2-Isopropyl-5-methylcyclohexyl pentanoate (50)

The title compound was prepared following the general procedure, purification by column chromatography on silica gel (petroleum ether/EtOAc = 100:1) yielded (67.7 mg, 94%) as a colorless oil. **^{1}H NMR ($CDCl_3$, 400 MHz)** δ = 4.68 (td, J = 10.9, 4.4 Hz, 1H), 2.28 (t, J = 7.5 Hz, 2H), 1.98 (d, J = 11.8 Hz, 1H), 1.87 (dd, J = 9.6, 7.0 Hz, 1H), 1.73 – 1.57 (m, 4H), 1.55 – 1.43 (m, 1H), 1.35 (dq, J = 14.7, 7.7, 7.2 Hz, 3H), 1.12 – 0.80 (m, 12H), 0.76 (d, J =

29

7.0 Hz, 3H). **^{13}C NMR ($CDCl_3$, 100 MHz)** δ = 173.4, 73.9, 47.0, 41.0, 34.5, 34.3, 31.4, 27.2, 26.3, 23.4, 22.3, 22.0, 20.8, 16.3, 13.7. **HRMS (ESI)**: Calcd for $C_{15}H_{29}O_2^+$ $[M+H]^+$ 241.2162, found 241.2157.

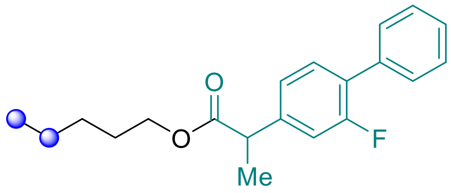

Pentyl 2-(2-fluoro-[1,1'-biphenyl]-4-yl)propanoate (51)

The title compound was prepared following the general procedure, purification by column chromatography on silica gel (petroleum ether/EtOAc = 100:1) yielded (75.4 mg, 80%) as a white solid. **^{1}H NMR ($CDCl_3$, 400 MHz)** δ = 7.55 (d, *J* = 8.0 Hz, 2H), 7.48 – 7.34 (m, 4H), 7.20 – 7.11 (m, 2H), 4.11 (t, *J* = 6.7 Hz, 2H), 3.76 (q, *J* = 7.2 Hz, 1H), 1.62 (p, *J* = 6.8 Hz, 2H), 1.55 (d, *J* = 7.2 Hz, 3H), 1.30 (tp, *J* = 7.4, 4.4, 3.6 Hz, 4H), 0.88 (t, *J* = 6.8 Hz, 3H). **^{13}C NMR ($CDCl_3$, 100 MHz)** δ = 174.1, 159.7 (d, *J* = 248.1 Hz), 142.0 (d, *J* = 8.1 Hz), 135.5, 130.7 (d, *J* = 4.2 Hz), 128.9 (d, *J* = 3.1 Hz), 128.5, 127.7 (d, *J* = 13.7 Hz), 127.6, 123.5 (d, *J* = 3.4 Hz), 115.2 (d, *J* = 23.6 Hz), 65.1, 45.1, 28.2, 28.0, 22.3, 18.3, 14.0. **^{19}F NMR ($CDCl_3$, 375 MHz)** δ = -117.76. **HRMS (ESI)**: Calcd for $C_{20}H_{24}FO_2^+$ $[M+H]^+$ 315.1755, found 315.1758.

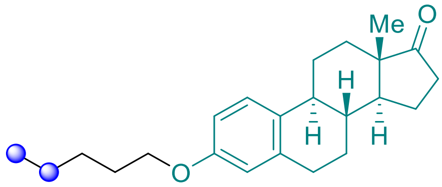

(8R,9S,13S,14S)-13-Methyl-3-(pentyloxy)-6,7,8,9,11,12,13,14,15,16-decahydro-17H-cyclopenta[a]phenanthren-17-one (52)

The title compound was prepared following the general procedure, purification by column chromatography on silica gel (petroleum ether/EtOAc = 100:1 to 50:1) yielded (100.0 mg, 98%) as a white solid. **^{1}H NMR ($CDCl_3$, 400 MHz)** δ = 7.19 (d, *J* = 8.5 Hz, 1H), 6.75 – 6.68 (m, 1H), 6.64 (s, 1H), 3.93 (t, *J* = 6.5 Hz, 2H), 2.89 (d, *J* = 8.0 Hz, 2H), 2.50 (dd, *J* = 18.8, 8.5 Hz, 1H), 2.40 (d, *J* = 9.9 Hz, 1H), 2.26 (d, *J* = 10.4 Hz, 1H), 2.06 (ddt, *J* = 52.7, 21.9, 10.0 Hz, 4H), 1.82 – 1.72 (m, 2H), 1.64 – 1.36 (m, 10H), 0.92 (d, *J* = 7.4 Hz, 6H). **^{13}C NMR ($CDCl_3$, 100 MHz)** δ = 220.9, 157.2, 137.7, 131.8, 126.3, 114.6, 112.1, 67.9, 50.4, 48.0, 44.0,

30

38.4, 35.9, 31.6, 29.7, 29.1, 28.2, 26.6, 25.9, 22.5, 21.6, 14.0, 13.9. **HRMS (ESI)**: Calcd for $C_{23}H_{33}O_2^+$ $[M+H]^+$ 341.2475, found 341.2467.

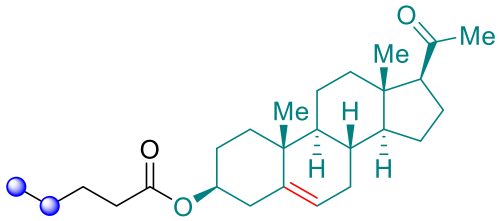

(3S,8S,9S,10R,13S,14S,17S)-17-Acetyl-10,13-dimethyl-2,3,4,7,8,9,10,11,12,13,14,15,16,17-tetradecahydro-1H-cyclopenta[a]phenanthren-3-yl pentanoate (53)

The title compound was prepared following the general procedure, purification by column chromatography on silica gel (petroleum ether/EtOAc = 100:1 to 50:1) yielded (112.8 mg, 94%) as a white solid. **^{1}H NMR ($CDCl_3$, 400 MHz)** δ = 5.37 (d, *J* = 4.1 Hz, 1H), 4.68 – 4.54 (m, 1H), 2.53 (t, *J* = 8.8 Hz, 1H), 2.28 (dt, *J* = 15.1, 7.1 Hz, 4H), 2.12 (s, 4H), 2.00 (dd, *J* = 30.5, 7.2 Hz, 2H), 1.86 (d, *J* = 9.9 Hz, 2H), 1.74 – 1.42 (m, 10H), 1.34 (h, *J* = 7.4 Hz, 2H), 1.26 – 1.10 (m, 3H), 1.01 (s, 4H), 0.91 (t, *J* = 7.3 Hz, 3H), 0.62 (s, 3H). **^{13}C NMR ($CDCl_3$, 100 MHz)** δ = 209.5, 173.3, 139.7, 122.3, 73.5, 63.7, 56.8, 49.9, 44.0, 38.8, 38.1, 37.0, 36.6, 34.4, 31.8, 31.6, 27.8, 27.1, 24.5, 22.8, 22.3, 21.0, 19.3, 13.7, 13.2. **HRMS (ESI)**: Calcd for $C_{26}H_{41}O_3^+$$[M+H]^+$ 401.3050, found 401.3051.

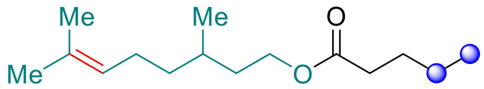

3,7-Dimethyloct-6-en-1-yl pentanoate (54)

The title compound was prepared following the general procedure, purification by column chromatography on silica gel (petroleum ether/EtOAc = 100:1) yielded (59.8 mg, 83%) as a colorless oil. **^{1}H NMR ($CDCl_3$, 400 MHz)** δ = 5.02 (t, *J* = 8.3 Hz, 1H), 4.11 – 3.96 (m, 2H), 2.22 (t, *J* = 7.5 Hz, 2H), 1.91 (tt, *J* = 14.9, 7.7 Hz, 2H), 1.63 – 1.43 (m, 10H), 1.40 – 1.23 (m, 4H), 1.16 – 1.06 (m, 1H), 0.89 – 0.81 (m, 6H). **^{13}C NMR ($CDCl_3$, 100 MHz)** δ = 174.0, 131.3, 124.6, 62.8, 37.0, 35.5, 34.2, 29.5, 27.1, 25.7, 25.4, 22.3, 19.4, 17.6, 13.7. **HRMS (ESI)**: Calcd for $C_{15}H_{29}O_2^+$ $[M+H]^+$ 241.2162, found 241.2163.

31

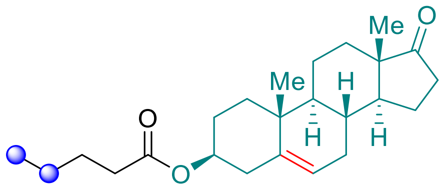

(3S,8R,9S,10R,13S,14S)-10,13-Dimethyl-17-oxo-2,3,4,7,8,9,10,11,12,13,14,15,16,17-tetradecahydro-1H-cyclopenta[a]phenanthren-3-yl pentanoate (55)

The title compound was prepared following the general procedure, purification by column chromatography on silica gel (petroleum ether/EtOAc = 100:1 to 50:1) yielded (85.9 mg, 77%) as a white solid. **^{1}H NMR ($CDCl_3$, 400 MHz)** δ = 5.41 (d, J = 4.9 Hz, 1H), 4.62 (tt, J = 10.5, 4.9 Hz, 1H), 2.46 (dd, J = 19.2, 8.8 Hz, 1H), 2.39 – 2.24 (m, 4H), 2.09 (dt, J = 18.9, 9.0 Hz, 2H), 2.00 – 1.91 (m, 1H), 1.91 – 1.80 (m, 3H), 1.56 (dddd, J = 49.1, 17.3, 13.6, 8.0 Hz, 8H), 1.40 – 1.24 (m, 4H), 1.21 – 1.10 (m, 1H), 1.05 (s, 4H), 0.96 – 0.83 (m, 6H). **^{13}C NMR ($CDCl_3$, 100 MHz)** δ = 173.3, 140.0, 121.8, 73.4, 51.7, 50.1, 47.5, 38.1, 37.0, 36.7, 35.9, 34.4, 31.5, 31.4, 30.8, 27.7, 27.1, 22.3, 21.9, 20.3, 19.4, 13.8, 13.6. **HRMS (ESI)**: Calcd for $C_{24}H_{37}O_3^+$ $[M+H]^+$ 373.2737, found 373.2738.

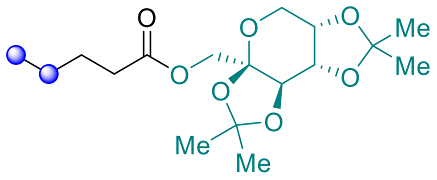

((3aR,5aS,8aS,8bR)-2,2,7,7-Tetramethyltetrahydro-3aH-bis([1,3]dioxolo)[4,5-b:4',5'-d]pyran-3a-yl)methyl pentanoate (56)

The title compound was prepared following the general procedure, purification by column chromatography on silica gel (petroleum ether/EtOAc = 100:1 to 50:1) yielded (101.1 mg, 98%) as a colorless oil. **^{1}H NMR ($CDCl_3$, 400 MHz)** δ = 5.54 (d, J = 4.9 Hz, 1H), 4.62 (dd, J = 7.9, 2.3 Hz, 1H), 4.32 (dt, J = 7.8, 4.3 Hz, 2H), 4.24 (dd, J = 7.9, 1.6 Hz, 1H), 4.16 (dd, J = 11.5, 7.8 Hz, 1H), 4.08 – 3.97 (m, 1H), 2.35 (t, J = 7.5 Hz, 2H), 1.61 (p, J = 7.5 Hz, 2H), 1.51 (s, 3H), 1.45 (s, 3H), 1.35 (dd, J = 14.5, 5.2 Hz, 8H), 0.91 (t, J = 7.3 Hz, 3H). **^{13}C NMR ($CDCl_3$, 100 MHz)** δ = 173.8, 109.6, 108.7, 96.3, 71.1, 70.7, 70.4, 66.0, 63.2, 33.9, 27.0, 26.0, 25.0, 24.5, 22.2, 13.7. **HRMS (ESI)**: Calcd for $C_{17}H_{29}O_7^+$ $[M+H]^+$ 345.1908, found 345.1901.

32

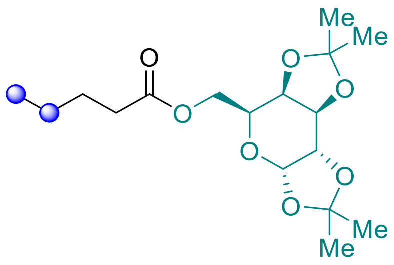

((3aS,5S,5aR,8aR,8bS)-2,2,7,7-Tetramethyltetrahydro-5H-bis([1,3]dioxolo)[4,5-b:4',5'-d]pyran-5-yl)methyl pentanoate (57)

The title compound was prepared following the general procedure, purification by column chromatography on silica gel (petroleum ether/EtOAc = 20:1 to 10:1) yielded (97.0 mg, 94%) as a colorless oil. **^{1}H NMR ($CDCl_3$, 400 MHz)** δ = 4.61 (dd, J = 7.9, 2.6 Hz, 1H), 4.40 (d, J = 11.7 Hz, 1H), 4.31 (d, J = 2.6 Hz, 1H), 4.27 – 4.22 (m, 1H), 4.04 (d, J = 11.7 Hz, 1H), 3.91 (dd, J = 13.0, 1.8 Hz, 1H), 3.77 (d, J = 13.0 Hz, 1H), 2.36 (t, J = 7.6 Hz, 2H), 1.63 (p, J = 7.6 Hz, 2H), 1.55 (s, 3H), 1.49 (s, 3H), 1.41 (s, 3H), 1.35 (d, J = 6.8 Hz, 5H), 0.91 (t, J = 7.3 Hz, 3H). **^{13}C NMR ($CDCl_3$, 100 MHz)** δ = 173.1, 109.1, 108.7, 101.6, 70.8, 70.5, 70.1, 65.1, 61.2, 33.8, 26.8, 26.5, 25.9, 25.2, 24.1, 22.2, 13.7. **HRMS (ESI)**: Calcd for $C_{17}H_{29}O_7^+$ $[M+H]^+$ 345.1908, found 345.1904.

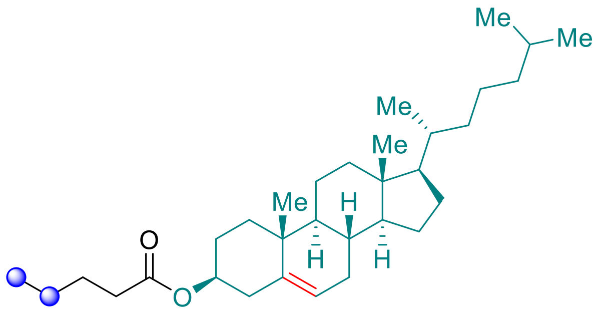

(3S,8S,9S,10R,13R,14S,17R)-10,13-Dimethyl-17-((R)-6-methylheptan-2-yl)-2,3,4,7,8,9,10,11,12,13,14,15,16,17-tetradecahydro-1H-cyclopenta[a]phenanthren-3-yl pentanoate (58)

The title compound was prepared following the general procedure, purification by column chromatography on silica gel (petroleum ether/EtOAc = 100:1 to 50:1) yielded (98.7 mg, 70%) as a white solid. **^{1}H NMR ($CDCl_3$, 400 MHz)** δ = 5.37 (d, J = 4.5 Hz, 1H), 4.61 (ddt, J = 11.2, 8.3, 4.2 Hz, 1H), 2.36 – 2.20 (m, 4H), 2.06 – 1.92 (m, 2H), 1.83 (ddt, J = 19.1, 9.5, 4.7 Hz, 3H), 1.52 (dtd, J = 42.9, 16.5, 15.8, 6.1 Hz, 9H), 1.40 – 1.30 (m, 5H), 1.29 – 0.94 (m, 14H), 0.94 – 0.83 (m, 12H), 0.68 (s, 3H). **^{13}C NMR ($CDCl_3$, 100 MHz)** δ = 173.3, 139.7,

33

122.6, 73.7, 56.7, 56.1, 50.0, 42.3, 39.7, 39.5, 38.2, 37.0, 36.6, 36.2, 35.8, 34.5, 31.9, 28.2, 28.0, 27.8, 27.2, 24.3, 23.8, 22.8, 22.6, 22.3, 21.0, 19.3, 18.7, 13.8, 11.9. **HRMS (ESI)**: Calcd for $C_{32}H_{55}O_2^+$ $[M+H]^+$ 471.4197, found 471.4196.

General procedure of electroreductive deuteration of unactivated alkenes

The electrolysis process was carried out in an undivided cell with a Fe plate anode (10 mm × 20 mm × 0.30 mm) and a Cu plate cathode (10 mm × 20 mm × 0.30 mm). To a 15 mL oven-dried undivided electrochemical cell equipped with a magnetic bar was added unactivated alkene (0.30 mmol, 1.0 equiv), $NiBr_2{\cdot}dtbpy$ (7.3 mg, 0.015 mmol, 5 mol%), Ph_3SiCl (17.7 mg, 0.06 mmol, 20 mol%), D_2O (120 mg, 6 mmol, 20 equiv.) and $^{n}Bu_4NBF_4$ (98.8 mg, 0.30 mmol, 1.0 equiv.) under Ar atmosphere. The electrolysis process was performed at 5.0 mA of constant current for 10 h at room temperature. After that, the electrodes were washed with EtOAc (3 x 5 mL) in an ultrasonic bath. H_2O (20 mL) was added to the organic system, and the resulting mixture was extracted with EtOAc (3 x 20 mL) and the combined organic phase was washed with brine, dried by anhydrous $MgSO_4$, filtered, and concentrated in vacuo. The crude product was purified by column chromatography to furnish the desired product.

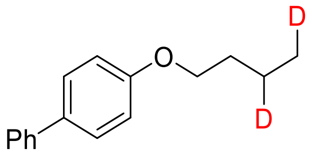

4-(Butoxy-3,4-*d*$_2$)-1,1'-biphenyl (59)

The title compound was prepared following the general procedure, purification by column chromatography on silica gel (petroleum ether/EtOAc = 100:1) yielded (57.5 mg, 84%) as a white solid. **^{1}H NMR ($CDCl_3$, 400 MHz)** δ = 7.58 (m, 4H), 7.45 (t, J = 7.5 Hz, 2H), 7.34 (t, J = 7.3 Hz, 1H), 7.01 (d, J = 8.6 Hz, 2H), 4.04 (t, J = 6.5 Hz, 2H), 1.84 (p, J = 6.7 Hz, 2H), 1.55 (p, J = 7.5 Hz, 1H), 1.03 (q, J = 7.1 Hz, 2H). **^{13}C NMR ($CDCl_3$, 100 MHz)** δ = 158.8, 141.0, 133.7, 128.8, 128.2, 126.8, 126.7, 114.9, 67.9, 31.4 (m), 19.2 (m), 13.8 (m). **HRMS (ESI)**: Calcd for $C_{16}H_{17}D_2O^+$ $[M+H]^+$ 229.1556, found 229.1542.

34

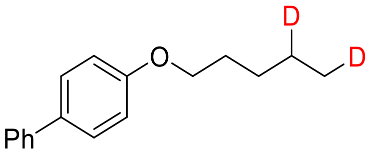

4-((Pentyl-4,5-d_2)oxy)-1,1'-biphenyl (60)

The title compound was prepared following the general procedure, purification by column chromatography on silica gel (petroleum ether/EtOAc = 100:1) yielded (58.1 mg, 80%) as a white solid. **^{1}H NMR ($CDCl_3$, 400 MHz)** δ = 7.59 (m, 4H), 7.46 (t, J = 7.6 Hz, 2H), 7.35 (t, J = 7.4 Hz, 1H), 7.02 (d, J = 8.7 Hz, 2H), 4.04 (t, J = 6.6 Hz, 2H), 1.86 (dt, J = 14.6, 6.7 Hz, 2H), 1.48 (dq, J = 21.4, 7.6 Hz, 3H), 0.99 (t, J = 7.1 Hz, 2H). **^{13}C NMR ($CDCl_3$, 100 MHz)** δ = 158.8, 141.0, 133.6, 128.8, 128.2, 126.8, 126.6, 114.8, 68.1, 29.1, 28.2 (m), 22.1 (m), 13.7 (m). **HRMS (ESI)**: Calcd for $C_{17}H_{19}D_2O^+$ [M+H]$^+$ 243.1712, found 243.1704.

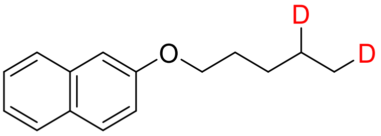

2-((Pentyl-4,5-d_2)oxy)naphthalene (61)

The title compound was prepared following the general procedure, purification by column chromatography on silica gel (petroleum ether/EtOAc = 100:1) yielded (55.1 mg, 85%) as a white solid. **^{1}H NMR ($CDCl_3$, 400 MHz)** δ = 7.89 – 7.74 (m, 3H), 7.55 – 7.45 (m, 1H), 7.39 (t, J = 7.5 Hz, 1H), 7.27 – 7.16 (m, 2H), 4.13 (t, J = 6.6 Hz, 2H), 1.92 (p, J = 6.7 Hz, 2H), 1.52 (dh, J = 21.4, 6.9 Hz, 3H), 1.02 (q, J = 7.2 Hz, 2H). **^{13}C NMR ($CDCl_3$, 100 MHz)** δ = 157.2, 134.7, 129.3, 128.9, 127.7, 126.7, 126.3, 123.5, 119.1, 106.6, 68.0, 29.0, 28.2 (m), 22.3 (m), 14.0 (m). **HRMS (ESI)**: Calcd for $C_{15}H_{17}D_2O^+$ [M+H]$^+$ 217.1556, found 217.1550.

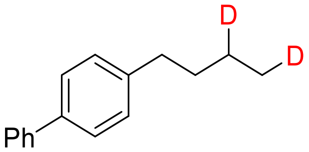

4-(Butyl-3,4-d_2)-1,1'-biphenyl (62)

The title compound was prepared following the general procedure, purification by column chromatography on silica gel (petroleum ether/EtOAc = 100:1) yielded (57.2 mg, 90%) as a colorless oil. **^{1}H NMR ($CDCl_3$, 400 MHz)** δ = 7.64 (d, J = 7.6 Hz, 2H), 7.57 (d, J = 8.1 Hz, 2H), 7.48 (t, J = 7.6 Hz, 2H), 7.38 (t, J = 7.3 Hz, 1H), 7.31 (d, J = 8.1 Hz, 2H), 2.76 – 2.65 (m, 2H), 1.70 (p, J = 7.4 Hz, 2H), 1.44 (m, 1H), 1.00 (m, 2H). **^{13}C NMR ($CDCl_3$, 100 MHz)**

35

δ = 142.1, 141.3, 138.6, 128.9, 128.8, 127.1, 127.0, 35.4, 33.6 (m), 22.0 (m), 14.0 (m). **HRMS (ESI)**: Calcd for $C_{16}H_{17}D_2^+$ $[M+H]^+$ 213.1607, found 213.1611.

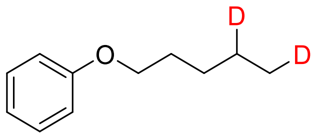

((Pentyl-4,5-*d*$_2$)oxy)benzene (63)

The title compound was prepared following the general procedure, purification by column chromatography on silica gel (petroleum ether/EtOAc = 100:1) yielded (25.4 mg, 51%) as a colorless oil. **^{1}H NMR ($CDCl_3$, 400 MHz)** δ = 7.38 – 7.26 (m, 2H), 7.03 – 6.87 (m, 3H), 3.99 (t, J = 6.6 Hz, 2H), 1.82 (p, J = 6.7 Hz, 2H), 1.44 (m, 3H), 0.96 (m, 2H). **^{13}C NMR ($CDCl_3$, 100 MHz)** δ = 159.1, 129.4, 120.4, 114.5, 67.9, 29. 0, 28.1 (m), 21.9 (m), 13.9 (m). **HRMS (ESI)**: Calcd for $C_{11}H_{15}D_2O^+$ $[M+H]^+$ 167.1399, found 167.1396.

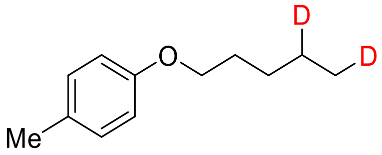

1-Methyl-4-((pentyl-4,5-*d*$_2$)oxy)benzene (64)

The title compound was prepared following the general procedure, purification by column chromatography on silica gel (petroleum ether/EtOAc = 100:1) yielded (30.0 mg, 63%) as a colorless oil. **^{1}H NMR ($CDCl_3$, 400 MHz)** δ = 7.09 (d, J = 8.2 Hz, 2H), 6.82 (d, J = 8.5 Hz, 2H), 3.94 (t, J = 6.6 Hz, 2H), 2.30 (s, 3H), 1.79 (p, J = 6.9 Hz, 2H), 1.52 – 1.32 (m, 3H), 0.94 (m, 2H). **^{13}C NMR ($CDCl_3$, 100 MHz)** δ = 157.0, 129.9, 129.6, 114.4, 68.1, 29.1, 28.2 (m), 22.3 (m), 20.5, 14.0 (m). **HRMS (ESI)**: Calcd for $C_{12}H_{17}D_2O^+$ $[M+H]^+$ 181.1556, found 181.1557.

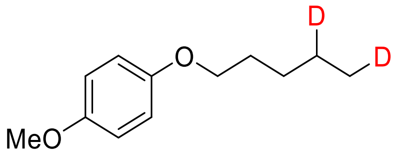

1-Methoxy-4-((pentyl-4,5-d2)oxy)benzene (65)

The title compound was prepared following the general procedure, purification by column chromatography on silica gel (petroleum ether/EtOAc = 100:1) yielded (41.2 mg, 70%) as a colorless oil. **^{1}H NMR ($CDCl_3$, 400 MHz)** δ = 6.84 (m, 4H), 3.91 (t, J = 6.6 Hz, 2H), 3.77 (s, 3H), 1.77 (p, J = 6.7 Hz, 2H), 1.42 (m, 3H), 0.93 (m, 2H). **^{13}C NMR ($CDCl_3$, 100 MHz)**

36

δ = 153.8, 153.4, 115.5, 114.7, 68.8, 55.8, 29.2, 28.2 (m), 22.4 (m), 14.2 (m). **HRMS (ESI)**: Calcd for $C_{16}H_{17}D_2O_2^+$ $[M+H]^+$ 197.1505, found 197.1504.

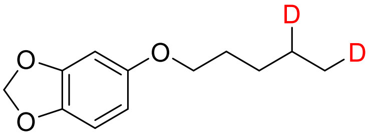

5-((Pentyl-4,5-*d*$_2$)oxy)benzo[*d*][1,3]dioxole (66)

The title compound was prepared following the general procedure, purification by column chromatography on silica gel (petroleum ether/EtOAc = 80:1) yielded (44.7 mg, 71%) as a colorless oil. **^{1}H NMR ($CDCl_3$, 400 MHz)** δ = 6.70 (d, J = 8.5 Hz, 1H), 6.49 (d, J = 2.4 Hz, 1H), 6.32 (dd, J = 8.5, 2.5 Hz, 1H), 5.90 (s, 2H), 3.87 (t, J = 6.6 Hz, 2H), 1.75 (p, J = 6.7 Hz, 2H), 1.39 (m, 3H), 0.92 (m, 2H). **^{13}C NMR ($CDCl_3$, 100 MHz)** δ = 154.7, 148.2, 141.4, 107.9, 105.6, 101.1, 98.0, 69.0, 29.0, 28.1 (m), 22.4 (m), 13.9 (m). **HRMS (ESI)**: Calcd for $C_{12}H_{15}D_2O_3^+$ $[M+H]^+$ 211.1298, found 211.1293.

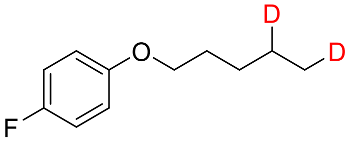

1-Fluoro-4-((pentyl-4,5-*d*$_2$)oxy)benzene (67)

The title compound was prepared following the general procedure, purification by column chromatography on silica gel (petroleum ether/EtOAc = 100:1) yielded (35.9 mg, 65%) as a colorless oil. **^{1}H NMR ($CDCl_3$, 400 MHz)** δ = 7.02 – 6.90 (m, 2H), 6.88 – 6.77 (m, 2H), 3.91 (t, J = 6.6 Hz, 2H), 1.77 (p, J = 6.7 Hz, 2H), 1.41 (m, 3H), 0.93 (m, 2H). **^{13}C NMR ($CDCl_3$, 100 MHz)** δ = 157.1 (d, J = 237.6 Hz), 155.2, 115.7 (d, J = 22.9 Hz), 115.4 (d, J = 8.0 Hz), 68.6, 29.0, 28.1 (m), 22.2 (m), 13.9 (m). **^{19}F NMR ($CDCl_3$, 375 MHz)** δ = -124.54. **HRMS (ESI)**: Calcd for $C_{11}H_{14}D_2FO^+$ $[M+H]^+$ 185.1305, found 185.1302.

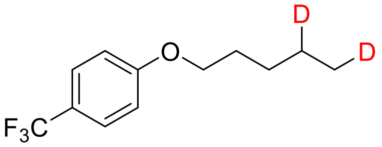

1-((Pentyl-4,5-*d*$_2$)oxy)-4-(trifluoromethyl)benzene (68)

The title compound was prepared following the general procedure, purification by column chromatography on silica gel (petroleum ether/EtOAc = 100:1) yielded (56.2 mg, 80%) as a colorless oil. **^{1}H NMR ($CDCl_3$, 400 MHz)** δ = 7.54 (d, J = 8.5 Hz, 2H), 6.95 (d, J = 8.5 Hz,

37

2H), 3.99 (t, J = 6.6 Hz, 2H), 1.81 (dt, J = 14.6, 6.7 Hz, 2H), 1.43 (m, 3H), 0.94 (m, 2H). **^{13}C NMR ($CDCl_3$, 100 MHz)** δ = 161.6, 126.8 (q, J = 3.7 Hz), 124.5 (q, J = 270.9 Hz), 122.5 (q, J = 32.6 Hz), 114.4, 68.2, 28.8, 28.0 (m), 22.4 (m), 13.9 (m). **^{19}F NMR ($CDCl_3$, 375 MHz)** δ = 19F NMR (CDCl3, 375 MHz) δ = -61.43. **HRMS (ESI)**: Calcd for $C_{12}H_{14}D_2F_3O^+$ $[M+H]^+$ 235.1273, found 235.1280.

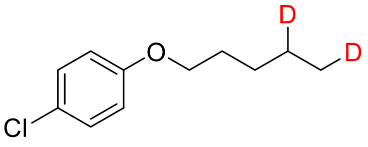

1-Chloro-4-((pentyl-4,5-d_2)oxy)benzene (69)

The title compound was prepared following the general procedure, purification by column chromatography on silica gel (petroleum ether/EtOAc = 100:1) yielded (43.2 mg, 72%) as a colorless oil. **^{1}H NMR ($CDCl_3$, 400 MHz)** δ = 7.26 (d, J = 8.8 Hz, 2H), 6.85 (d, J = 8.8 Hz, 2H), 3.95 (t, J = 6.6 Hz, 2H), 1.81 (p, J = 6.7 Hz, 2H), 1.45 (m, 3H), 0.96 (m, 2H). **^{13}C NMR ($CDCl_3$, 100 MHz)** δ = 157.8 129.3, 125.3, 115.7, 68.3, 28.9, 28.2 (m), 22.5 (m), 13.9 (m). **HRMS (ESI)**: Calcd for $C_{11}H_{14}D_2ClO^+$ $[M+H]^+$ 201.1010, found 201.1012.

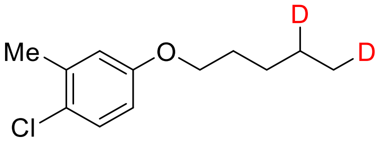

1-Chloro-2-methyl-4-((pentyl-4,5-d_2)oxy)benzene (70)

The title compound was prepared following the general procedure, purification by column chromatography on silica gel (petroleum ether/EtOAc = 100:1) yielded (50.1 mg, 78%) as a colorless oil. **^{1}H NMR ($CDCl_3$, 400 MHz)** δ = 7.21 (d, J = 8.7 Hz, 1H), 6.78 (d, J = 2.9 Hz, 1H), 6.67 (dd, J = 8.7, 3.0 Hz, 1H), 3.91 (t, J = 6.6 Hz, 2H), 2.35 (s, 3H), 1.78 (p, J = 6.7 Hz, 2H), 1.41 (m, 3H), 0.94 (m, 2H). **^{13}C NMR ($CDCl_3$, 100 MHz)** δ = 157.7, 136.9, 129.5, 125.5, 117.1, 113.0, 68.2, 28.9, 28.1 (m), 22.0 (m), 20.3, 13.9 (m). **HRMS (ESI)**: Calcd for $C_{12}H_{16}D_2ClO^+$ $[M+H]^+$ 215.1166, found 215.1159.

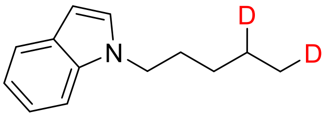

1-(Pentyl-4,5-d_2)-1H-indole (71)

The title compound was prepared following the general procedure, purification by column

38

chromatography on silica gel (petroleum ether/EtOAc = 100:1) yielded (46.5 mg, 82%) as a yellow oil. **^{1}H NMR ($CDCl_3$, 400 MHz)** δ = 7.64 (d, *J* = 7.9 Hz, 1H), 7.36 (d, *J* = 8.2 Hz, 1H), 7.21 (t, *J* = 7.6 Hz, 1H), 7.15 – 7.06 (m, 2H), 6.49 (d, *J* = 3.1 Hz, 1H), 4.12 (t, *J* = 7.2 Hz, 2H), 1.85 (p, *J* = 7.2 Hz, 2H), 1.32 (m, 3H), 0.89 (m, 2H). **^{13}C NMR ($CDCl_3$, 100 MHz)** δ = 136.0, 128.6, 127.8, 121.3, 120.9, 119.2, 109.4, 100.8, 46.4, 30.0, 29.1 (m), 22.1 (m), 13.9 (m). **HRMS (ESI)**: Calcd for $C_{13}H_{16}D_2N^+$ $[M+H]^+$ 190.1559, found 190.1558.

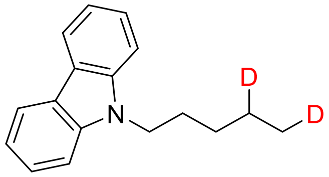

9-(Pentyl-4,5-*d*$_2$)-9H-carbazole (72)

The title compound was prepared following the general procedure, purification by column chromatography on silica gel (petroleum ether/EtOAc = 100:1) yielded (58.1 mg, 81%) as a white solid. **^{1}H NMR ($CDCl_3$, 400 MHz)** δ = 8.08 (d, *J* = 7.8 Hz, 2H), 7.51 – 7.33 (m, 4H), 7.21 (t, *J* = 7.4 Hz, 2H), 4.24 (t, *J* = 7.3 Hz, 2H), 1.83 (p, *J* = 7.3 Hz, 2H), 1.39 – 1.22 (m, 3H), 0.84 (t, *J* = 6.6 Hz, 2H). **^{13}C NMR ($CDCl_3$, 100 MHz)** δ = 140.5, 125.6, 122.9, 120.4, 118.8, 108.7, 43.1, 29.4 (m), 28.7, 22.3 (m), 13.8 (m). **HRMS (ESI)**: Calcd for $C_{17}H_{18}D_2N^+$ $[M+H]^+$ 240.1716, found 240.1717.

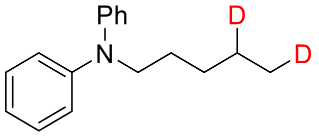

***N*-(Pentyl-4,5-*d*$_2$)-N-phenylaniline (73)**

The title compound was prepared following the general procedure, purification by column chromatography on silica gel (petroleum ether/EtOAc = 100:1) yielded (62.2 mg, 86%) as a colorless oil. **^{1}H NMR ($CDCl_3$, 400 MHz)** δ = 7.28 (t, *J* = 7.9 Hz, 4H), 7.05 – 6.91 (m, 6H), 3.76 – 3.65 (m, 2H), 1.69 (p, *J* = 7.7 Hz, 2H), 1.33 (m, 3H), 0.89 (m, 2H). **^{13}C NMR ($CDCl_3$, 100 MHz)** δ = 140.5, 125.6, 122.9, 120.4, 118.7, 108.7, 43.1, 29.4 (m), 28.7, 22.5 (m), 13.9 (m). **HRMS (ESI)**: Calcd for $C_{17}H_{20}D_2N^+$ $[M+H]^+$ 242.1872, found 242.1873.

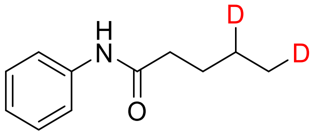

39

N-Phenyl-(pentyl-4,5-d_2)amide (74)

The title compound was prepared following the general procedure, purification by column chromatography on silica gel (petroleum ether/EtOAc = 4:1) yielded (51.0 mg, 95%) as a white solid. **^{1}H NMR ($CDCl_3$, 400 MHz)** δ = 7.96 (s, 1H), 7.56 (d, J = 8.1 Hz, 2H), 7.30 (t, J = 7.4 Hz, 2H), 7.10 (t, J = 7.3 Hz, 1H), 2.37 (t, J = 7.6 Hz, 2H), 1.70 (q, J = 7.5 Hz, 2H), 1.39 (m, 1H), 0.93 (m, 2H). **^{13}C NMR ($CDCl_3$, 100 MHz)** δ = 172.0, 138.2, 128.9, 124.1, 120.1, 37.4, 27.7 (m), 22.4 (m), 13.7 (m). **HRMS (ESI)**: Calcd for $C_{11}H_{14}D_2NO^+$ $[M+H]^+$ 180.1352, found 180.1348.

Eectroreduction of styrenes under standard conditons

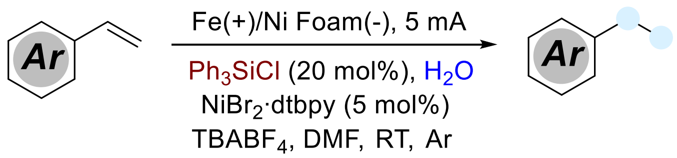

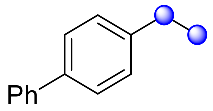

4-Ethyl-1,1'-biphenyl (79)

The title compound was prepared following the general procedure, purification by column chromatography on silica gel (petroleum ether) yielded (51.8 mg, 95%) as a white solid. **^{1}H NMR ($CDCl_3$, 400 MHz)** δ = 7.58 (d, J = 7.2 Hz, 2H), 7.52 (d, J = 8.2 Hz, 2H), 7.42 (t, J = 7.4 Hz, 2H), 7.35 – 7.24 (m, 3H), 2.70 (q, J = 7.6 Hz, 2H), 1.28 (t, J = 7.6 Hz, 3H). **^{13}C NMR ($CDCl_3$, 100 MHz)** δ = 143.5, 141.3, 138.7, 128.8, 128.4, 127.2, 127.1, 28.7, 15.7.

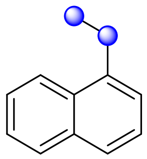

1-Ethylnaphthalene (80)

The title compound was prepared following the general procedure, purification by column chromatography on silica gel (petroleum ether) yielded (37.9 mg, 81%) as a colorless oil. **^{1}H NMR ($CDCl_3$, 400 MHz)** δ = 8.11 (d, J = 8.2 Hz, 1H), 7.91 (d, J = 7.8 Hz, 1H), 7.76 (d, J = 8.0 Hz, 1H), 7.61 – 7.50 (m, 2H), 7.49 – 7.43 (m, 1H), 7.39 (d, J = 6.9 Hz, 1H), 3.17 (q, J = 7.5 Hz, 2H), 1.44 (t, J = 7.6 Hz, 3H). **^{13}C NMR ($CDCl_3$, 100 MHz)** δ = 140.4, 133.9, 131.9, 128.9, 126.5, 125.8, 125.5, 125.0, 123.9, 26.0, 15.2.

40

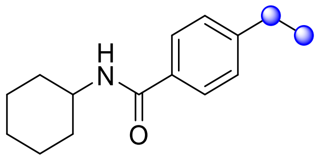

***N*-Cyclohexyl-4-ethylbenzamide (81)**

The title compound was prepared following the general procedure, purification by column chromatography on silica gel (petroleum ether/EtOAc = 3:1) yielded (62.4 mg, 90%) as a white solid. **^{1}H NMR ($CDCl_3$, 400 MHz)** δ = 7.67 (d, *J* = 8.1 Hz, 2H), 7.22 (d, *J* = 7.9 Hz, 2H), 6.06 (s, 1H), 4.03 – 3.89 (m, 1H), 2.67 (q, *J* = 7.6 Hz, 2H), 2.01 (d, *J* = 9.5 Hz, 2H), 1.73 (m, 2H), 1.63 (d, *J* = 10.3 Hz, 1H), 1.40 (q, *J* = 12.6 Hz, 2H), 1.23 (m, 6H). **^{13}C NMR ($CDCl_3$, 100 MHz)** δ = 166.7, 147.9, 132.6, 128.0, 127.0, 48.7, 33.4, 28.9, 25.7, 25.1, 15.5.

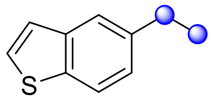

5-Ethylbenzo[*b*]thiophene (82)

The title compound was prepared following the general procedure, purification by column chromatography on silica gel (petroleum ether) yielded (26.7 mg, 55%) as a white solid. **^{1}H NMR ($CDCl_3$, 400 MHz)** δ = 7.80 (d, *J* = 8.3 Hz, 1H), 7.66 (s, 1H), 7.42 (d, *J* = 5.4 Hz, 1H), 7.29 (d, *J* = 5.4 Hz, 1H), 7.22 (d, *J* = 8.2 Hz, 1H), 2.79 (q, *J* = 7.6 Hz, 2H), 1.32 (t, *J* = 7.6 Hz, 3H). **^{13}C NMR ($CDCl_3$, 100 MHz)** δ = 140.6, 140.1, 137.3, 126.5, 125.1, 123.8, 122.4, 122.3, 29.0, 16.2.

Ectroreduction of conjugated diene under standard conditons

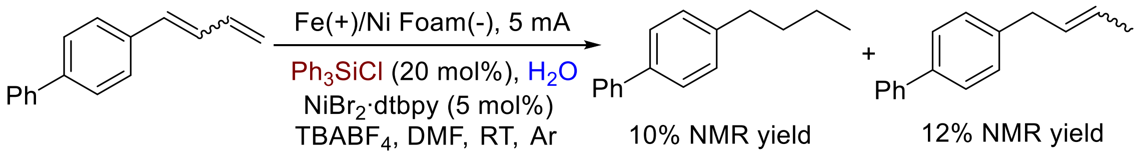

41

2. Supplementary Discussion

2.1 Mechanism research

2.1.1. Investigation on the role of chlorosilane

Intermediate capture experiment

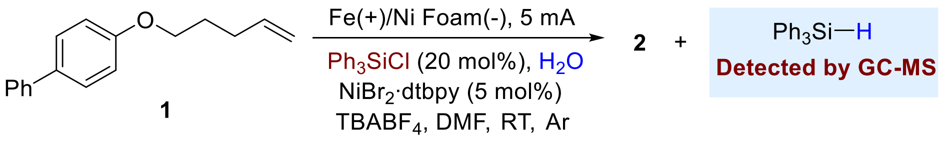

The electrolysis process was carried out under the standard conditions. Afterwards, the reaction mixture was analyzed by GC-MS, and Ph_3SiH (m/z 260.10) was detected at 14.78 minute.

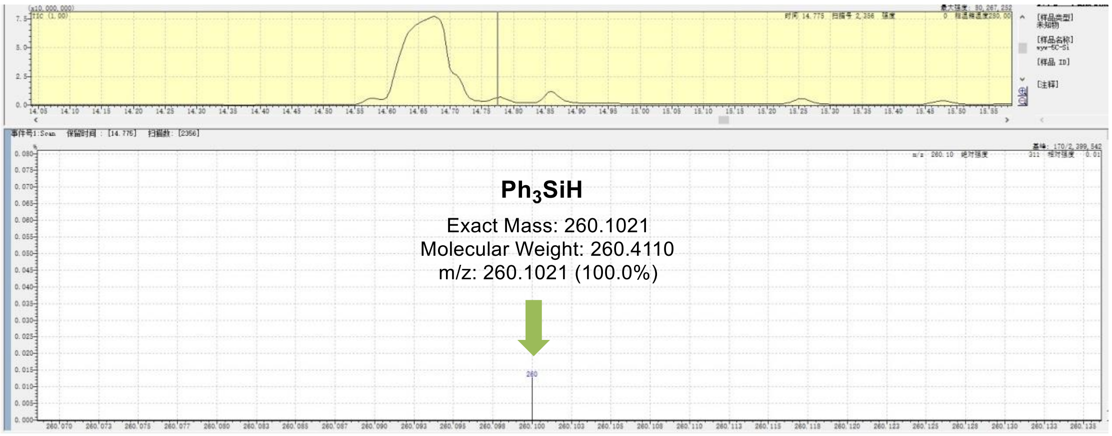

Control experiment: using Ph_3SiH in replacement of Ph_3SiCl as catalyst

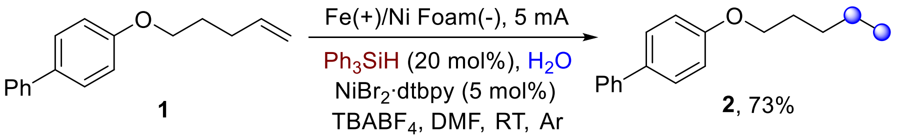

The electrolysis process was carried out in an undivided cell with a Fe plate anode (10 mm × 20 mm × 0.10 mm) and a Ni Foam cathode (10 mm × 20 mm × 0.30 mm). To a 15 mL oven-dried undivided electrochemical cell equipped with a magnetic bar was added unactivated alkene (0.30 mmol, 1.0 equiv), $NiBr_2$·dtbpy (7.3 mg, 0.015 mmol, 5 mol%), Ph_3SiH (15.6 mg, 0.06 mmol, 20 mol%), H_2O (16.2 mg. 3.0 equiv.) and $^nBu_4NBF_4$ (98.8 mg, 0.30 mmol, 1.0 equiv.) under Ar atmosphere. The electrolysis process was performed at 5.0 mA of constant current for 10 h at room temperature.

42

After that, the electrodes were washed with EtOAc (3 x 5 mL) in an ultrasonic bath. H_2O (20 mL) was added to the organic system, and the resulting mixture was extracted with EtOAc (3 x 20 mL) and the combined organic phase was washed with brine, dried by anhydrous $MgSO_4$, filtered, and concentrated in vacuo. The crude product was dealed with a short column chromatography, and the ^{1}H NMR yields was reported in 73% yield (using 1,3,5-trimethoxybenzene as the internal standard).

Control experiment on C–Si bond cleavage

Preparation of trimethyl(4-phenylbutyl)silane (**75**):

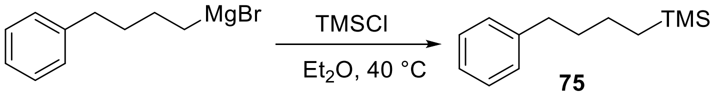

The title compound was prepared following a modified literature procedure.[24] Firstly, Grignard reagent was prepared from (4-bromobutyl)benzene according to literature reports.[25] The Grignard reagent (10 mmol, 1.0 equiv.) was added dropwise to a solution of TMSCl (15 mmol, 1.5 equiv.) in 20.0 mL anhydrous Et_2O. The reaction mixture was then heated in a 40 °C oil bath overnight. After cooling to room temperature, the cloudy suspension was quenched by 10 wt% NaOH aqueous. The layers were separated, and the aqueous layer extracted with Et_2O (3 x 20 mL). The organic phase was washed with brine, dried over anhydrous $MgSO_4$, filtered and concentrated under reduced pressure. Purification by reduced pressure distillation to give the product as a colorless liquid. **^{1}H NMR ($CDCl_3$, 400 MHz)** δ 7.36 – 7.29 (m, 2H), 7.23 (m, 3H), 2.71 – 2.61 (m, 2H), 1.69 (p, J = 7.3 Hz, 2H), 1.47 – 1.34 (m, 2H), 0.65 – 0.50 (m, 2H), 0.03 (s, 9H). **^{13}C NMR ($CDCl_3$, 100 MHz)** δ 144.6, 130.0, 129.8, 127.1, 37.3, 37.1, 25.3, 18.1, 0.0. Spectroscopic data match those previously reported in the literature.[26]

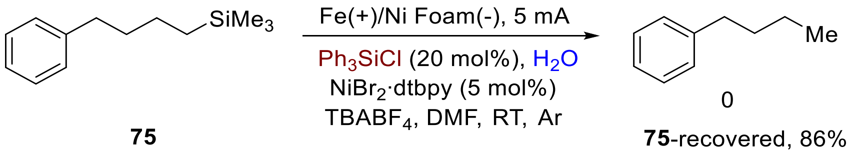

The electrolysis process was carried out in an undivided cell with a Fe plate anode (10 mm × 20 mm × 0.10 mm) and a Ni Foam cathode (10 mm × 20 mm × 0.30 mm). To a 15 mL oven-dried undivided electrochemical cell equipped with a magnetic bar was added **75** (0.30 mmol, 1.0 equiv.), $NiBr_2$·dtbpy (7.3 mg, 0.015 mmol, 5 mol%), Ph_3SiCl

43

(17.7 mg, 0.06 mmol, 20 mol%), H_2O (16.2 mg, 0.9 mmol, 3.0 equiv.) and ^{n}Bu$_4$NBF$_4$ (98.8 mg, 0.30 mmol, 1.0 equiv.) under Ar atmosphere. The electrolysis process was performed at 5.0 mA of constant current for 10 h at room temperature. After that, the electrodes were washed with EtOAc (3 x 5 mL) in an ultrasonic bath. H_2O (20 mL) was added to the organic system, and the resulting mixture was extracted with EtOAc (3 x 20 mL) and the combined organic phase was washed with brine, dried by anhydrous $MgSO_4$, filtered, and concentrated in vacuo. The crude product was dealed with a short column chromatography, no C–Si bond hydrogenation product was detected and **75** was recovered in 86% yield (using 1,3,5-trimethoxybenzene as the internal standard).

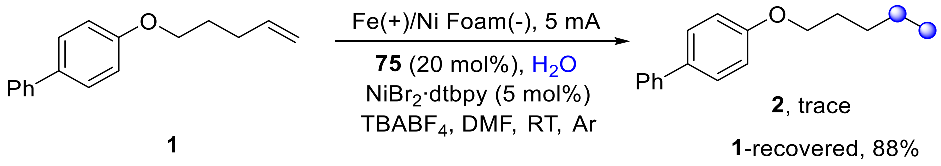

The electrolysis process was carried out in an undivided cell with a Fe plate anode (10 mm × 20 mm × 0.10 mm) and a Ni Foam cathode (10 mm × 20 mm × 0.30 mm). To a 15 mL oven-dried undivided electrochemical cell equipped with a magnetic bar was added substrate **1** (0.30 mmol, 1.0 equiv.), $NiBr_2$·dtbpy (7.3 mg, 0.015 mmol, 5 mol%), **75** (12.4 mg, 0.06 mmol, 20 mol%), H_2O (16.2 mg, 0.9 mmol, 3.0 equiv.) and ^{n}Bu$_4$NBF$_4$ (98.8 mg, 0.30 mmol, 1.0 equiv.) under Ar atmosphere. The electrolysis process was performed at 5.0 mA of constant current for 10 h at room temperature. After that, the electrodes were washed with EtOAc (3 x 5 mL) in an ultrasonic bath. H_2O (20 mL) was added to the organic system, and the resulting mixture was extracted with EtOAc (3 x 20 mL) and the combined organic phase was washed with brine, dried by anhydrous $MgSO_4$, filtered, and concentrated in vacuo. The crude product was dealed with a short column chromatography, trace amount of product **2** was detected and substrate **1** was recovered in 88% yield (using 1,3,5-trimethoxybenzene as the internal standard).

The above results indicated that the activation of alkenes through the formation of C–Si bond in the transformation could be ruled out.

44

2.1.2. Deuteration experiments

Preparation of dimethyl(phenyl)silane-*d* ($PhMe_2SiD$):

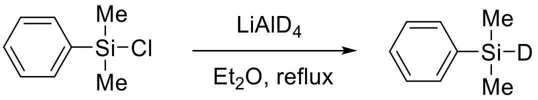

The title compound was prepared following a modified literature procedure.[27] To a stirring suspension of $LiAlD_4$ (210 mg, 5 mmol) in dry Et_2O (12 mL) was added $PhMe_2SiCl$ (2.55g, 15 mmol) dropwise at ambient temperature under Ar. The reaction mixture was refluxed at 40 °C for 12 h. The reaction was cooled to room temperature. Then, the reaction was quenched by adding aqueous solution of sodium hydroxide (15 mL, 10 wt%) into the crude reaction mixture, which was subsequently extracted by diethyl ether for three times. The combined organic layers were dried over Na_2SO_4, evaporated under reduced pressure, and purified by column chromatography on silica gel to give $PhMe_2SiD$ as a colorless oil in 90% yield (1.85 g, 99% D). **^{1}H NMR ($CDCl_3$, 400 MHz)** δ 7.61 – 7.53 (m, 2H), 7.39 (m, 3H), 4.41 – 4.45 (m, 0 H), 0.37 (s, 6 H).

^{1}H NMR of $PhMe_2SiD$

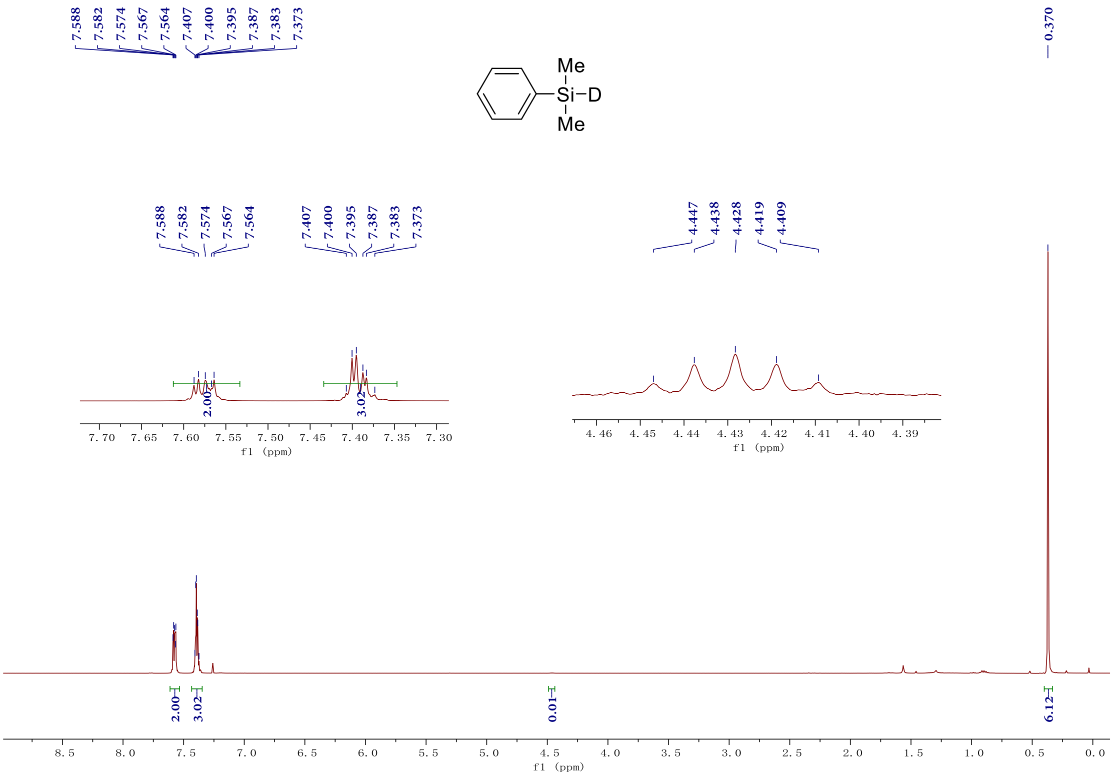

45

Deuteration experiments using $PhMe_2SiD$

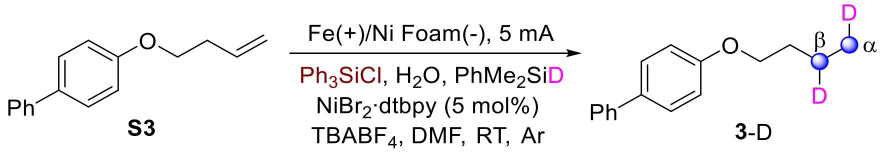

The electrolysis process was carried out in an undivided cell with a Fe plate anode (10 mm × 20 mm × 0.10 mm) and a Ni Foam cathode (10 mm × 20 mm × 0.30 mm). To a 15 mL oven-dried undivided electrochemical cell equipped with a magnetic bar was added substrate **S3** (0.30 mmol, 1.0 equiv), $NiBr_2$·dtbpy (7.3 mg, 0.015 mmol, 5 mol%) (or without $NiBr_2$·dtbpy), Ph_3SiCl (17.7 mg, 0.06 mmol, 20 mol%), H_2O (16.2 mg, 3.0 equiv.), $PhMe_2SiD$ (205.5 mg, 1.5 mmol 5.0 equiv.) and $^{n}Bu_4NBF_4$ (98.8 mg, 0.30 mmol, 1.0 equiv.) under Ar atmosphere. The electrolysis process was performed at 5.0 mA of constant current for 10 h at room temperature. After that, the electrodes were washed with EtOAc (3 x 5 mL) in an ultrasonic bath. H_2O (20 mL) was added to the organic system, and the resulting mixture was extracted with EtOAc (3 x 20 mL) and the combined organic phase was washed with brine, dried by anhydrous $MgSO_4$, filtered, and concentrated in vacuo. The crude product was purified by column chromatography to furnish **3**-D in 90% and 88% yields, respectively, with D-incorporation (α/β: 85%/79% and 56%/85%), which demonstrated that the hydrogen came from silane.

46

^{1}H NMR of 3-D performed with Ni-complex ($CDCl_3$)

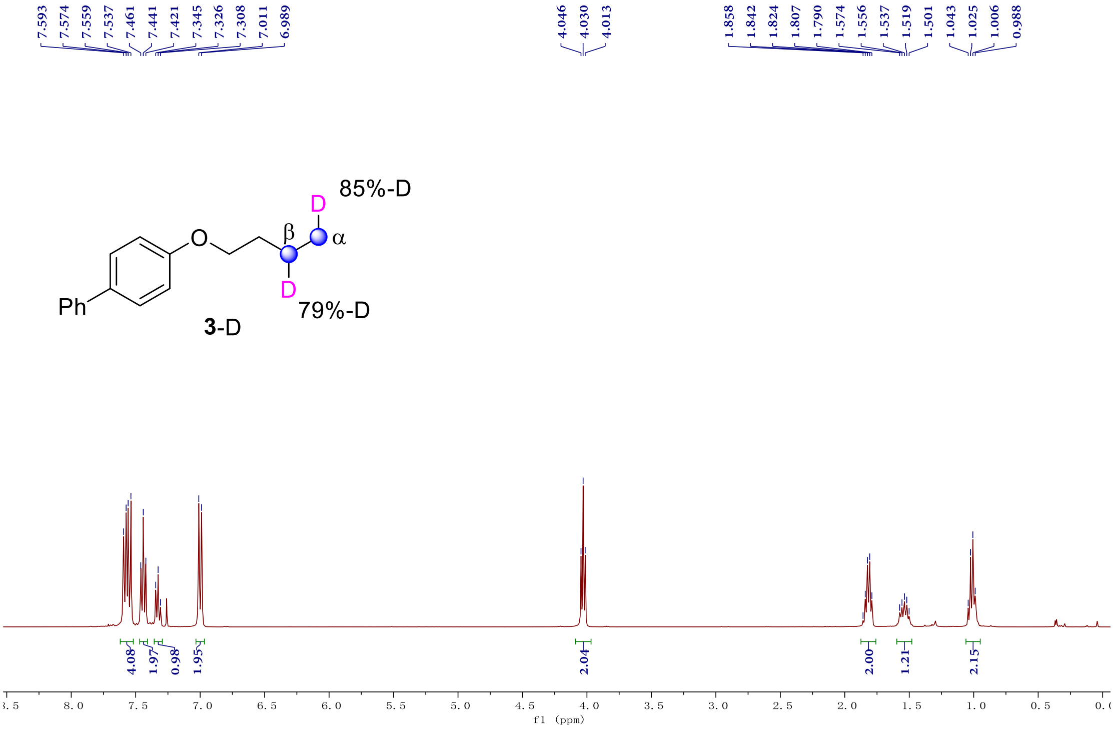

^{1}H NMR of 3-D performed without Ni-complex ($CDCl_3$)

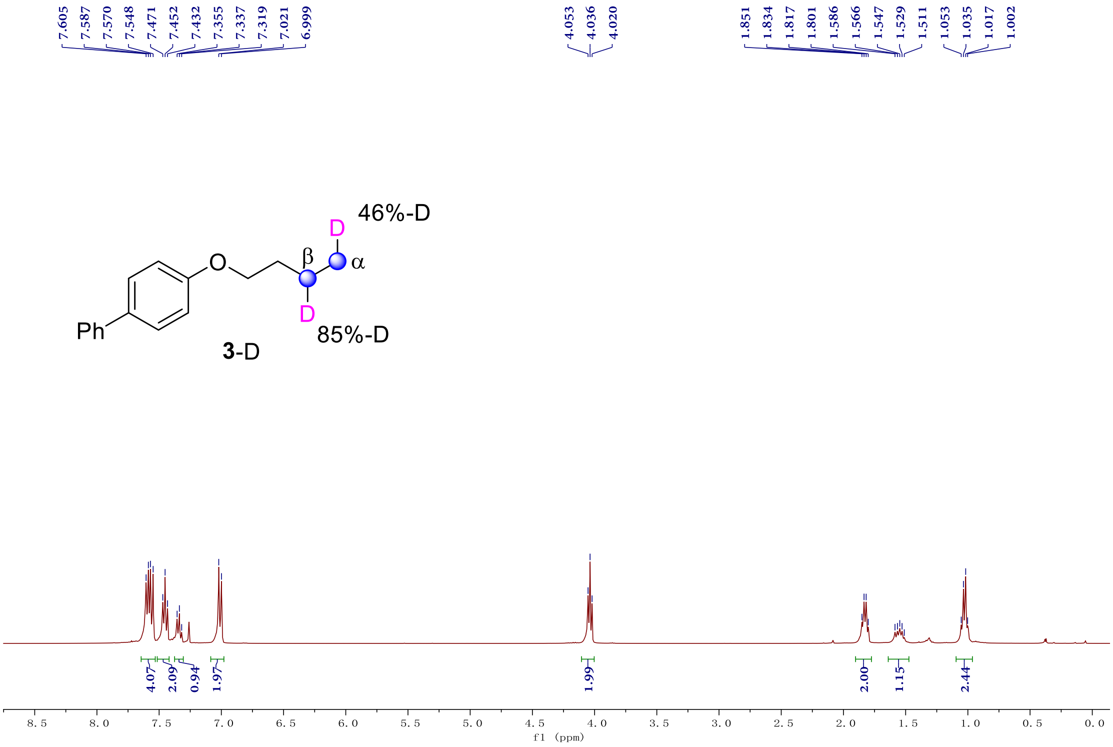

Deuteration experiments of internal alkenes using D_2O

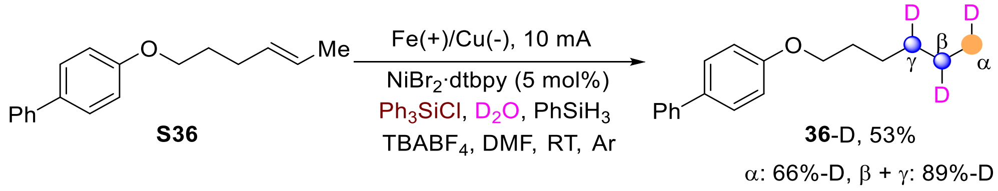

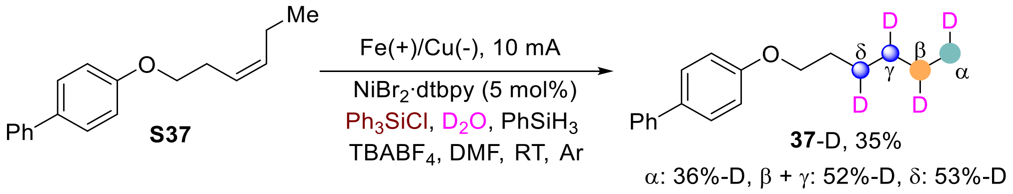

The electrolysis process was carried out in an undivided cell with a Fe plate anode (10 mm × 20 mm × 0.30 mm) and a Cu plate cathode (10 mm × 20 mm × 0.30 mm). To a 15 mL oven-dried undivided electrochemical cell equipped with a magnetic bar was added substrates **S36** or **S37** (0.30 mmol, 1.0 equiv), $NiBr_2$·dtbpy (7.3 mg, 0.015 mmol, 5 mol%), Ph_3SiCl (8.9 mg, 0.03 mmol, 10 mol%), $PhSiH_3$ (9.7 mg, 0.09 mmol, 30 mol%), D_2O (120 mg, 20 equiv.) and $^nBu_4NBF_4$ (98.8 mg, 0.30 mmol, 1.0 equiv.) under Ar atmosphere. The electrolysis process was performed at 5.0 mA of constant current for 10 h at room temperature. After that, the electrodes were washed with EtOAc (3 x 5 mL) in an ultrasonic bath. H_2O (20 mL) was added to the organic system, and the resulting mixture was extracted with EtOAc (3 x 20 mL) and the combined organic phase was washed with brine, dried by anhydrous $MgSO_4$, filtered, and concentrated in vacuo. The crude product was purified by column chromatography to furnish α/β/γ- and α/β/γ/δ-positions deuteration products **36**-D and **37**-D in 53% and 35% yields, respectively. However, no product could be detected in the absence of Ni-complex in this transformation, which indicated that Ni-complex could promote the chain-walking process.

48

^{1}H NMR of **36-**D ($CDCl_3$)

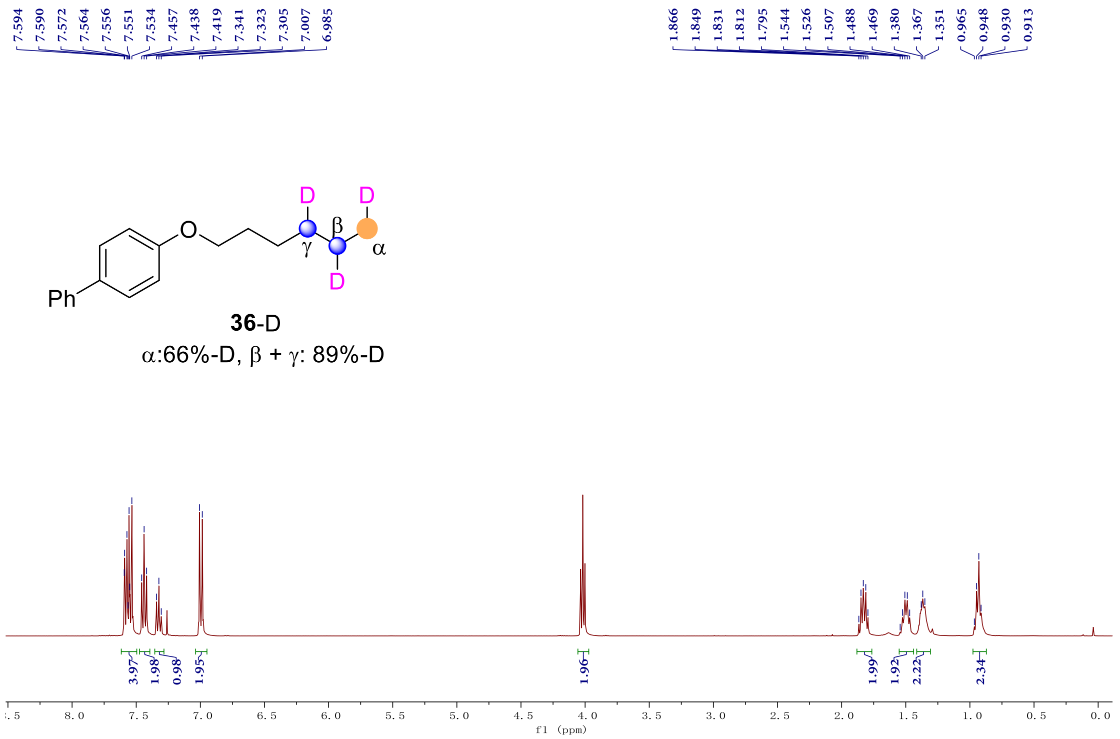

^{1}H NMR of **37-**D ($CDCl_3$)

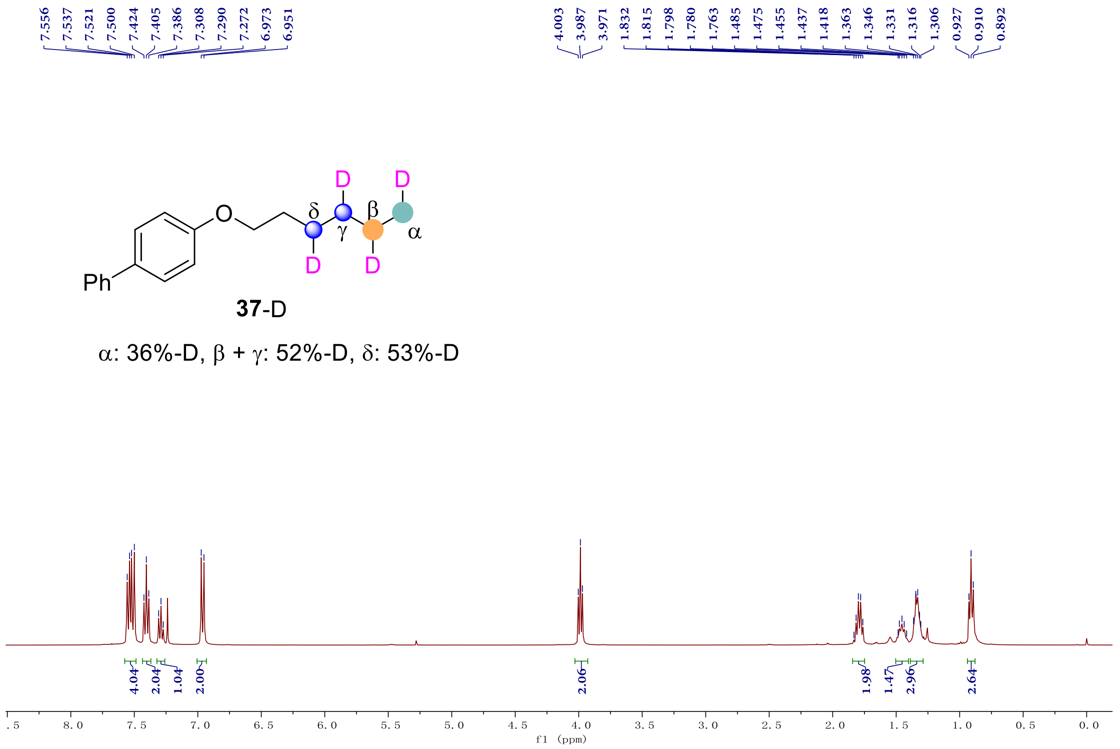

49

2.1.3. Ring-opening studies

Preparation of ((1*R*,2*S*)-2-vinylcyclopropyl)benzene:[28]

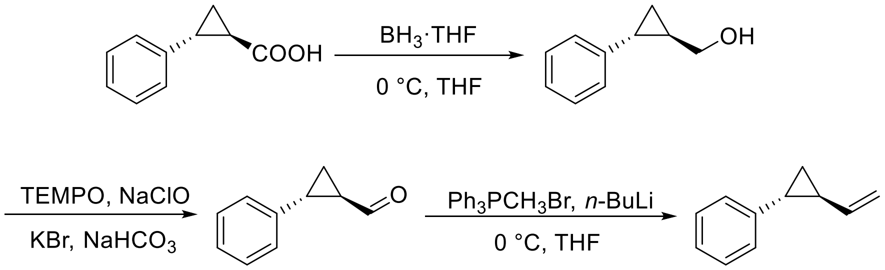

The title compound was prepared following a modified three-step literature procedure. In a 100 mL dried Schlenk tube with a stir bar under argon trans-2-phenylcyclopropane-1- carboxylic acid (1.62 g, 10.0 mmol, 1.0 equiv.) was dissolved in THF (20 mL). The solution was cooled to 0 °C and BH_3·THF (13.0 mL of a 1.0 M solution in THF, 13.0 mmol, 1.3 equiv.) was added dropwise. The reaction mixture was stirred for 1 h at 0 °C and then 1 h at rt. As TLC suggested that starting material remained, BH_3·THF (5.0 mL, 5.0 mmol, 0. 5 equiv.) was added and the reaction mixture was stirred for another 1.5 h. The reaction mixture was then quenched by dropwise addition of MeOH at 0 °C and water was added until the formation of two layers could be observed. The layers were separated with Et_2O and brine and the aqueous layer extracted with Et_2O (3 x 25 mL). The combined organic layers were washed with brine (2 x 20 mL), dried over MgSO4, filtered and concentrated to afford trans-(3-phenylcyclopropyl)methanol (1.48 g, 10.0 mmol, quant.) as a colorless liquid.

The latter was transferred to a 250 mL round bottom flask with a stir bar and dissolved in CH_2Cl_2 (25 mL). After cooling to 0 °C, TEMPO (16.0 mg, 0.1 mmol, 1.0 mol%) and an aqueous solution of KBr (1.30 g, 11.0 mmol, 1.1 equiv.) and $NaHCO_3$ (2.77 g, 33.0 mmol, 3.3 eq.) in H_2O (56 mL) were added and the reaction mixture stirred. Next, sodium hypochlorite (14%, 14 mL) was added and, after stirring for 5 min, another 5 mL sodium hypochlorite solution was added. After the reaction mixture was stirred at 0 °C for 1 h, the reaction mixture was quenched with sat. aq. $Na_2S_2O_3$. The layers were separated, and the aqueous layer extracted with CH_2Cl_2 (3 x 25 mL). The combined organic layers were dried over $MgSO_4$, filtered, concentrated and trans-2-phenylcyclopropanecarbaldehyde (1.36 g, 9.3 mmol, 93%) was obtained.

50

A dried Schlenk tube equipped with a stir bar under argon was loaded with methyltriphenylphosphonium bromide (3.57 g, 10.0 mmol, 1.08 equiv.) dissolved in THF (50 mL). The solution was cooled to 0 °C and stirring initiated. *n*-BuLi (6.32 mL, 1.6 M in hexane, 10.0 mmol, 1.08 equiv.) was added dropwise and the solution stirred for 30 min at rt. Then, a solution of trans-2- phenylcyclopropanecarbaldehyde (1.36 g, 9.3 mmol, 1.0 equiv.) in THF (9 mL) was added and the reaction mixture stirred for 20 h. The reaction mixture was quenched with sat. NH_4Cl, the aqueous layer washed with CH_2Cl_2 (3 x 50 mL) and the combined organic layers dried over $MgSO_4$, filtered and concentrated. After purification by column chromatography on silica gel (pentane), the title compound (446 mg, 3.4 mmol, 34% over three steps) was obtained as a colorless liquid. The analytical data is in accordance with the reported data. **^{1}H NMR ($CDCl_3$, 400 MHz)** δ 7.35 – 7.28 (m, 2H), 7.20 (t, *J* = 7.4 Hz, 1H), 7.15 – 7.08 (m, 2H), 5.59 (m, 1H), 5.16 (dd, *J* = 17.0, 1.1 Hz, 1H), 4.99 (dd, *J* = 10.3, 1.5 Hz, 1H), 1.97 (m, 1H), 1.75 (m, 1H), 1.25 (m, 1H), 1.15 (m, 1H). **^{13}C NMR ($CDCl_3$, 100 MHz)** δ 142.4, 140.7, 128.4, 125.7, 125.7, 112.6, 27.5, 25.3, 16.8.

Ring-opening experiment

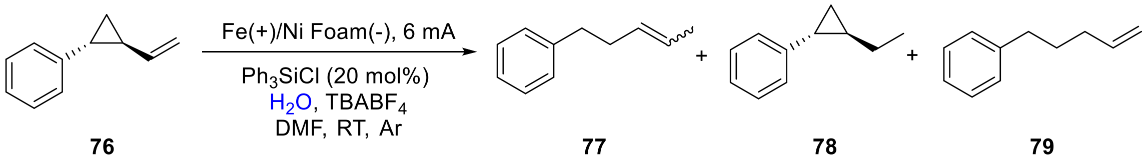

76 **77** **78** **79**

The electrolysis process was carried out in an undivided cell with a Fe plate anode (10 mm × 20 mm × 0.10 mm) and a Ni Foam cathode (10 mm × 20 mm × 0.30 mm). To a 15 mL oven-dried undivided electrochemical cell equipped with a magnetic bar was added unactivated alkene (**76**) (0.30 mmol, 1.0 equiv.), Ph_3SiCl (17.7 mg, 20 mol%), H_2O (16.2 mg. 3.0 equiv.) and $^nBu_4NBF_4$ (98.8 mg, 0.30 mmol, 1.0 equiv.) under Ar atmosphere. The electrolysis process was performed at 5.0 mA of constant current for 12 h at room temperature. After that, the electrodes were washed with EtOAc (3 x 5 mL) in an ultrasonic bath. H_2O (20 mL) was added to the organic system, and the resulting mixture was extracted with EtOAc (3 x 20 mL) and the combined organic phase was washed with brine, dried by anhydrous $MgSO_4$, filtered, and concentrated in vacuo. Then, the reaction system was analyzed by ^{1}H NMR Spectrum and giving

51

hydrogenation product **77** (33%), ring-opening products **78** (20%) and **79** (12%) (Supplementary Figure 6).

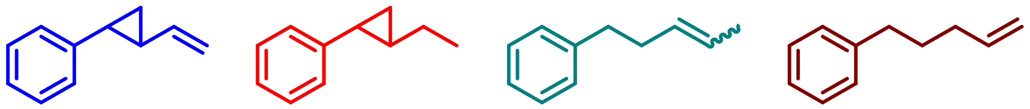

76 **77** **78** **79**

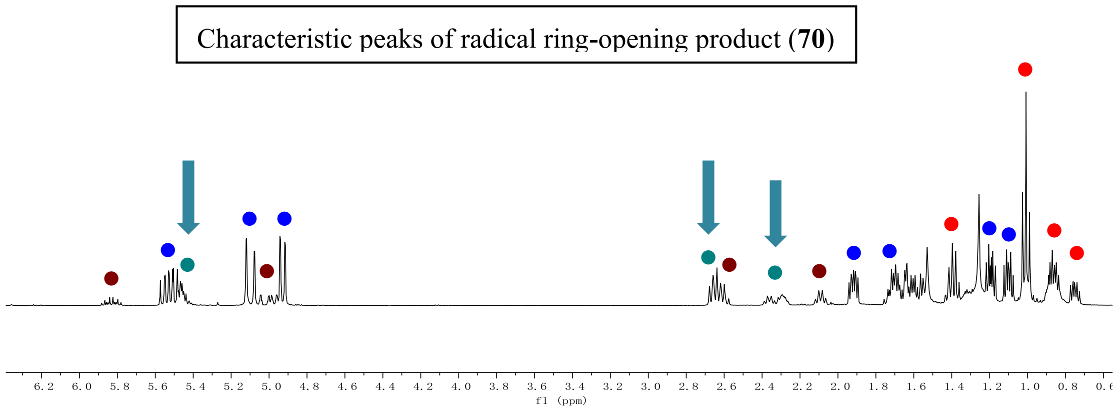

Supplementary Figure 6.^{1}H NMR spectrum of ring-opening experiment

Note: Products **77** and **78** were obtained from two different pathways (path a and b). Firstly, [Fe]-hydride species underwent migration insertion process with unactivated alkene **76** to provide intermediates **I** and **I'**. The ring-opening product **78** was formed through *β*-carbon elimination of intermediate **I**, which was followed by protonation or further reductive elimination to give product **77** (path a). And intermediate **I'** could then transform into hydrogenation to give product **78** (path b).

For ring-opening product **79**, according to the literature reports,[29] we speculated that a direct electrolysis ring-opening process was involved in this transformation (path c).

2.1.4. ^{19}F NMR spectroscopic evidence for the formation of fluorosilane

To test the stability of TMSCl and TMSBr in this system, we systemically investigated the behaviors of TMSCl and TMSBr *via* NMR tests. We found most of TMSCl/TMSBr could be transformed into TMSF in very short time without electricity. The characteristic ^{19}F NMR signal of TMSF could be detected in 3 miniutes in the system (13% for TMSCl, 14% for TMSBr *vs.* 20%, 4-fluoroanisole as internal standard), and

52

there was no significant decline after stirred for 12 h (14% for TMSCl, 14% for TMSBr). Experimental details for NMR tests: All the experiments were conducted using 5 mL reaction tube on the 0.3 mmol scale at room temperature in 1.0 mL DMF (substrate **1** (0.3 mmol), TMSCl/TMSBr (20 mol%), $NiBr_2$·dtbpy (5 mol%), H_2O (3.0 equiv.), $TBABF_4$ (1.0 equiv.)). Then $CDCl_3$ and 4-fluoroanisole (0.3 mmol) were added to the system, and took ^{19}F NMR test (Supplementary Figure 7).

These results indicated highly active chlorosilane/bromosilane could exist in the system in the form of fluorosilane. And fluorosilane could efficiently promote the transformation as well (Table S-2, entry 4).

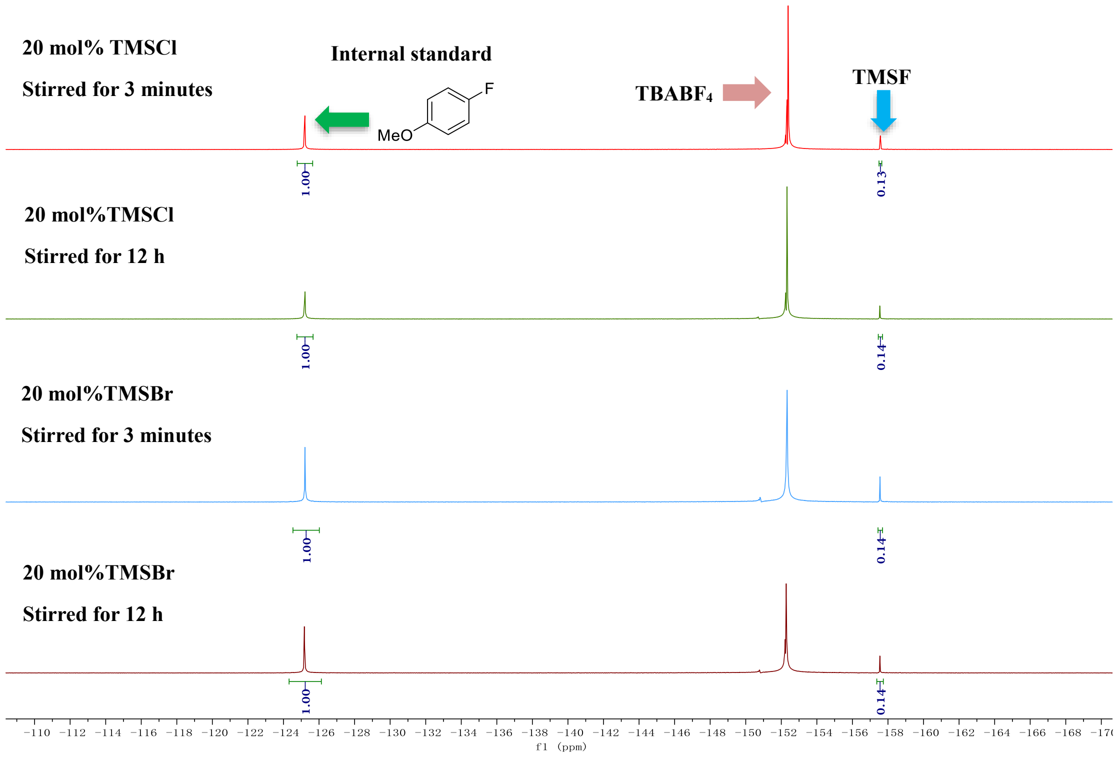

Supplementary Figure 7. ^{19}F NMR evidence of TMSF

53

2.1.5. DFT calculations

All density functional theory (DFT) calculations were carried out using the Gaussian 09 software package[30]. Geometry optimizations for all the intermediates and transition structures were calculated with B3LYP-D3[31-33] level of theory with a basis set of def2SVP[34] in gas phase. Vibrational frequency calculations were carried out for all stationary points at the same level to confirm if each optimized structure is a local minimum (no imaginary frequency) or a transition state (only one imaginary frequency and vibration mode of the negative frequency corresponds to the bond breaking and bond forming process that is anticipated). The thermal corrections for the free energies were also obtained at 298.15 K and 1.0 atm. A correction factor which consists on using an entropic term that is half (0.5) of the entropy in vacuum was applied in solvent.[35-39] In order to obtain more accurate energies, solvation single-point energy were calculated at higher level of the M062X[40]/def2TZVP in *N,N*-dimethylformamide solvent with SMD solvation model[41]. The calculated 3D optimized structures are displayed utilizing CYLview visualization program.[42]

The 18-electron nickel(0) species ^{3}**Ni-0** undergoes oxidative addition with silane through transition state ^{3}**Ni-TS1A** with an energy barrier of 16.6 kcal·mol^{-1}, leading to the formation of intermediate ^{1}**Ni-INT1A**, which then undergoes disproportionation with ^{3}**Ni-0** to generate Ni(I)-H species ^{2}**Ni-INT2A** and Ni(I)-Si species ^{2}**Ni-INT2B**.[43-45]

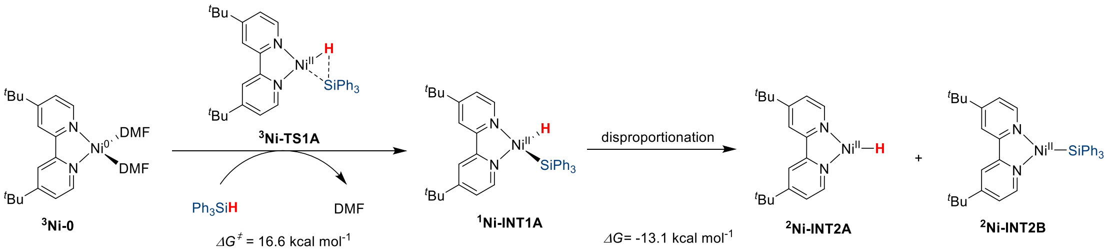

Supplementary Figure 8. The generation of Ni-H species ^{2}Ni-INT2A.

The calculation results indicate that the nickel hydride species ^{2}**Ni-INT2A** may undergo two types of migratory insertion processes with the alkene **1**. The energy barrier of the transition state ^{2}**Ni-TS3A** is 4.1 kcal mol^{-1} lower than that of ^{2}**Ni-TS3B**,

54

suggesting that the branched intermediate ^{2}**Ni-INT3A** is kinetically favorable in the nickel catalytic cycle. Subsequently, the favored intermediate ^{2}**Ni-INT3A** undergoes a protonation reaction with water through the transition state ^{2}**Ni-TS4A** with an energy barrier of 16.6 kcal mol^{-1}, forming the hydroxy nickel species ^{2}**Ni-INT4A**. Followed by σ-bond metathesis with silane, silanol is generated, and ^{2}**Ni-INT2A** is regenerated, thereby completing the catalytic cycle.

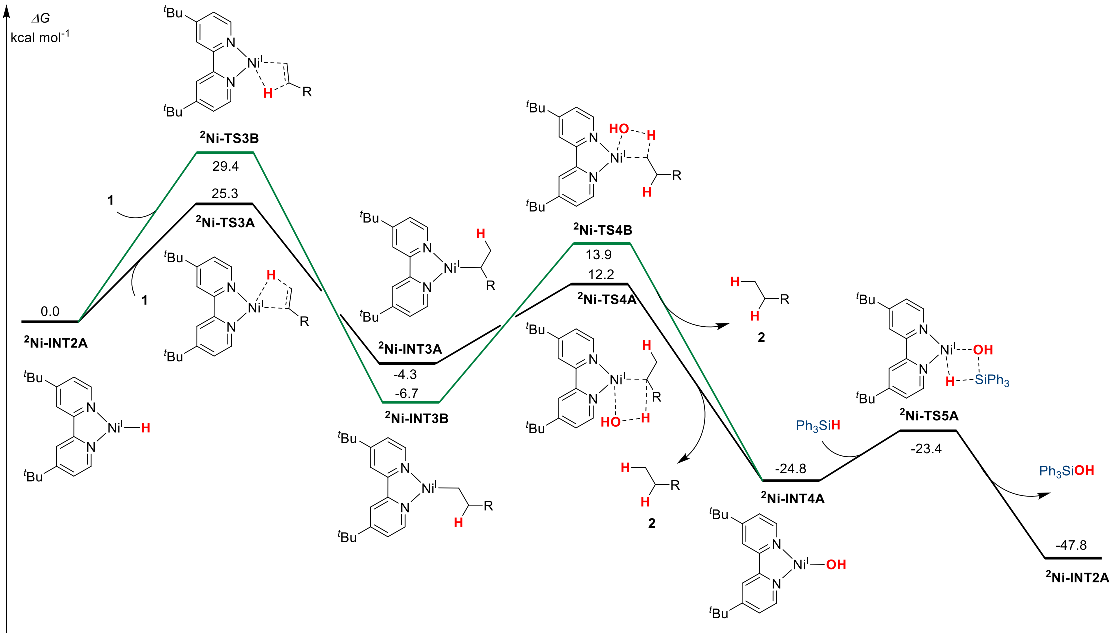

Supplementary Figure 9. The calculated energy profile for the reduction of unactivated alkenes involving ^{2}Ni-INT2A.

The intermediate ^{5}**Fe-INT2A** undergoes a migration insertion process with alkene **1**, resulting in the formation of the branched intermediate ^{5}**Fe-INT3B**. This process occurs via the transition state ^{5}**Fe-TS3B**, which has a lower activation energy compared to the competitive linear transition state ^{5}**Fe-TS3A** (20.9 vs 23.7 kcal mol^{-1}). Subsequently, the preferred ^{5}**Fe-INT3B** undergoes protonation with water via the transition state ^{5}**Fe-TS4B** with an energy barrier of 9.3 kcal mol^{-1}, leading to the formation of the hydroxy iron species ^{5}**Fe-INT4A**. Further, a σ-bond metathesis reaction takes place, resulting in the generation of silanol **D**. This reaction proceeds via the transition state ^{5}**Fe-TS5A** with an energy barrier of 7.7 kcal mol^{-1}.

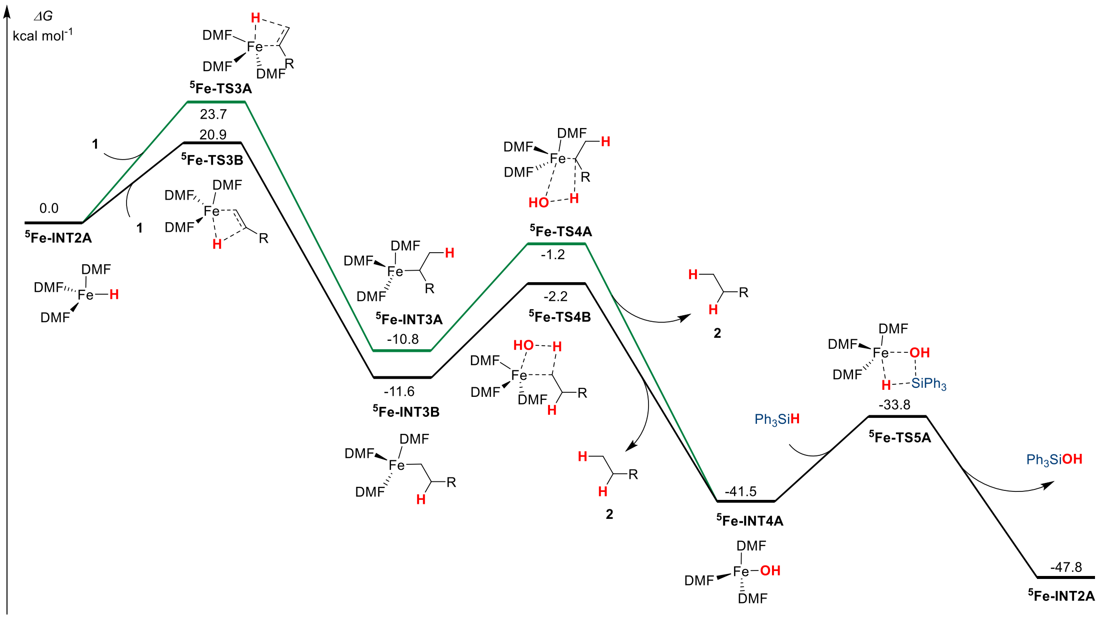

Supplementary Figure 10. The calculated energy profile for the reduction of unactivated alkenes involving ^{5}Fe-INT2A.

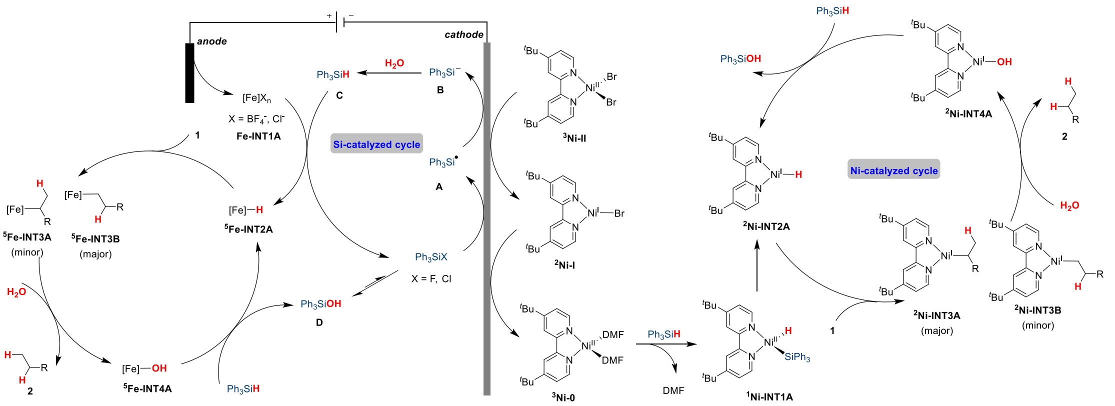

Supplementary Figure 11. The proposed catalytic cycle for the electroreduction of unactivated alkenes using H_2O as hydrogen source.

Supplementary Table 10. The calculated energies of stationary points (in Hartree/Particle).

Structure	E_{ele}	H_{corr}	G_{corr}	-0.5TS	G_{sol}
**1**	-733.814488	0.321515	0.258214	-0.031651	-733.524624
**2**	-735.042128	0.345539	0.281161	-0.032189	-734.728778
DMF	-248.505244	0.107981	0.073143	-0.017419	-248.414682
**C**	-985.015784	0.299239	0.233714	-0.032763	-984.749307

56

H_2O	-76.434111	0.025006	0.002908	-0.011049	-76.420154
**[3]Ni-II**	-7466.711713	0.411902	0.328360	-0.041771	-7466.341582
**[1]Ni-II**	-7466.674638	0.413160	0.329819	-0.041671	-7466.303149
**[2]Ni-I**	-4892.438426	0.408676	0.328070	-0.040303	-4892.070053
**[3]Ni-0**	-2815.182526	0.626219	0.515846	-0.055187	-2814.611494
**[1]Ni-0**	-2815.132787	0.626087	0.520605	-0.052741	-2814.559441
**[3]Ni-TS1A**	-3303.150036	0.701999	0.587953	-0.057023	-3302.505060
**[1]Ni-TS1A**	-3303.112665	0.702864	0.588418	-0.057223	-3302.467024
**[3]Ni-INT1A**	-3303.153224	0.704829	0.589332	-0.057749	-3302.506144
**[1]Ni-INT1A**	-3303.155201	0.706804	0.591710	-0.057547	-3302.505944
**[2]Ni-INT2A**	-2318.733693	0.411498	0.336954	-0.037272	-2318.359467
**[2]Ni-INT2B**	-3302.587500	0.697767	0.578159	-0.059804	-3301.949537
**[2]Ni-TS3A**	-3052.525540	0.735955	0.627697	-0.054129	-3051.843714
**[2]Ni-INT3A**	-3052.574502	0.739964	0.627088	-0.056438	-3051.890976
**[2]Ni-TS3B**	-3052.516676	0.735805	0.623143	-0.056331	-3051.837202
**[2]Ni-INT3B**	-3052.573199	0.739632	0.617320	-0.061156	-3051.894723
**[2]Ni-TS4A**	-3128.985936	0.761852	0.640564	-0.060644	-3128.284728
**[2]Ni-TS4B**	-3128.984857	0.761836	0.643783	-0.059027	-3128.282047
**[2]Ni-INT4A**	-2393.996824	0.420309	0.343404	-0.038453	-2393.614967
**[2]Ni-TS5A**	-3379.024096	0.720868	0.602643	-0.0591125	-3378.362341
**[5]Fe-INT2A**	-2009.737199	0.342611	0.254809	-0.043901	-2009.438489
**[3]Fe-INT2A**	-2009.688656	0.344716	0.262099	-0.041309	-2009.385248
**[1]Fe-INT2A**	-2009.639679	0.344342	0.262583	-0.04088	-2009.336217
**[5]Fe-TS3A**	-2743.530532	0.665713	0.544611	-0.060551	-2742.925370
**[3]Fe-TS3A**	-2743.498415	0.666813	0.548199	-0.059307	-2742.890909
**[5]Fe-INT3A**	-2743.589909	0.670940	0.548374	-0.061283	-2742.980252
**[3]Fe-INT3A**	-2743.541685	0.672090	0.551375	-0.060358	-2742.929952
**[1]Fe-INT3A**	-2743.488785	0.673698	0.554244	-0.059727	-2742.874814
**[5]Fe-TS3B**	-2743.532785	0.665389	0.540544	-0.062423	-2742.929818

**^{3}Fe-TS3B**	-2743.499001	0.666756	0.544395	-0.061181	-2742.893425
**^{1}Fe-TS3B**	-2743.469113	0.668045	0.551098	-0.058474	-2742.859542
**^{5}Fe-INT3B**	-2743.586939	0.671179	0.539625	-0.065777	-2742.981537
**^{3}Fe-INT3B**	-2743.532667	0.672097	0.543890	-0.064104	-2742.924673
**^{1}Fe-INT3B**	-2743.494754	0.673832	0.554956	-0.059438	-2742.880360
**^{5}Fe-TS4A**	-2820.017320	0.694059	0.570354	-0.061853	-2819.385113
**^{5}Fe-TS4B**	-2820.016778	0.693886	0.566041	-0.063923	-2819.386814
**^{5}Fe-INT4A**	-2085.029047	0.351806	0.265171	-0.043318	-2084.720558
**^{5}Fe-TS5A**	-3070.046920	0.651718	0.526695	-0.062512	-3069.457714
**Si-OH**	-1060.314127	0.306616	0.238759	-0.033929	-1060.041439

Note: E_{ele} (electronic energies in solvent), H_{corr} (the thermal correction to enthalpy in gas), G_{corr} (the thermal correction to Gibbs free energy in gas), -0.5TS (half the gas phase entropy), and G_{sol} (sum of electronic and thermal free energies in solvent).

58

2.1.6. Investigation on the heterogeneous hydrogenation

We conducted a series of parallel experiments using substrate **1** under the standard conditions, and no obvious deposits was observed in most cases (see the pictures below: **Cathode-1**) after electrolysis, and some black deposits was obtained in a few cases (**Cathode-2**). Although the morphologies of the cathode surface were not identical after the electrolysis, the reaction efficiency were stable in all of these experiments (hydrogenation products in 96~99% yields).

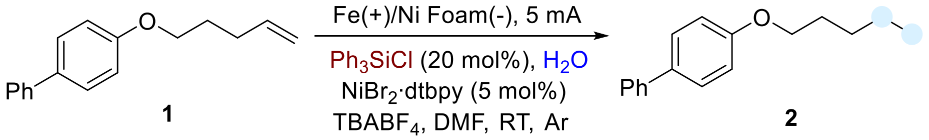

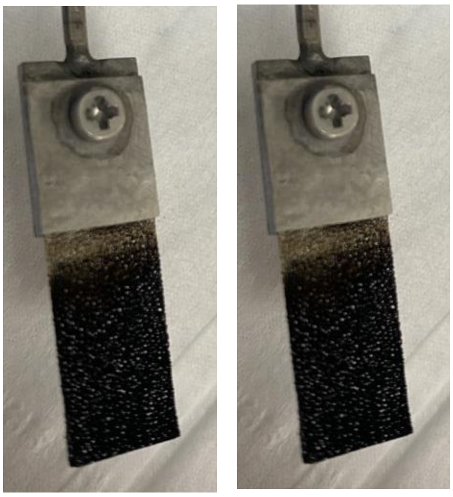

Cathode-1

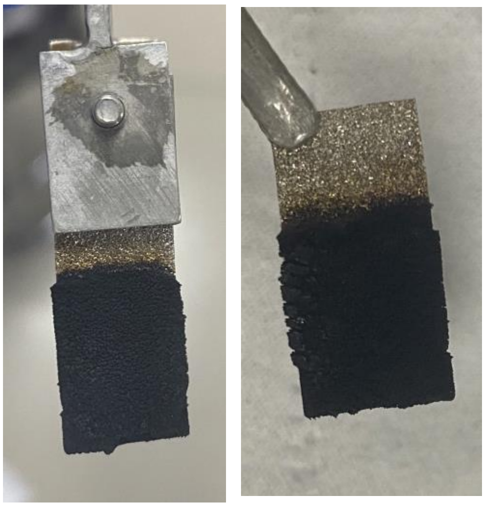

Cathode-2

Then, we carried out a series control experiments using **Cathode-1**/**Cathode-2** as cathode, Zn/Mg as anode, as showing in the following table. Firstly, when using Zn(+)/Ni Foam(-) as electrodes under the standard conditions, only 17% hydrogenation product was obtained (Supplementary Table 11, entry 2); and using Zn(+)/**Cathode-1**(-) as electrodes under standard conditions, trace amount of desired product was detected (entry 3); employing Zn(+)/**Cathode-2**(-) as electrodes, the hydrogenation product was provided in 83% yield (entry 4). Then, control experiments using Mg as anode and **Cathode-1**/**Cathode-2** as cathode were conducted under the standard conditions, the hydrogenation products were obtained in 0% and 37% yields (entries 6 and 7), respectively.

59

Supplementary Table 11. Control experiments using the coated cathodes.

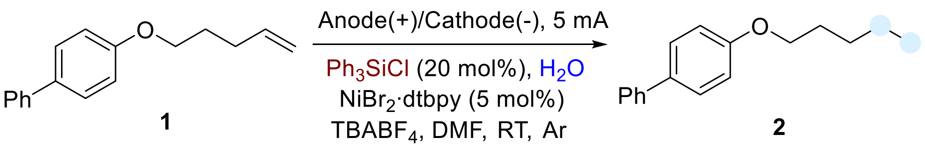

Entry	Anode(+)/Cathode(-)	Yield (%)[a]
1	Fe(+)/Ni Foam(-)	99
2	Zn(+)/Ni Foam(-)	17
3	Zn(+)/**Cathode-1**(-)	trace
4	Zn(+)/**Cathode-2**(-)	83
5	Mg(+)/Ni Foam(-)	0
6	Mg(+)/**Cathode-1**(-)	0
7	Mg(+)/**Cathode-2**(-)	37

[a]Reaction conditions: undivided cell, anode(+)/cathode(-), constant current = 5.0 mA, **1** (0.3 mmol, 1.0 equiv.), Ph_3SiCl (20 mol%), $NiBr_2$·dtbpy (5.0 mol%), H_2O (3.0 equiv.), $TBABF_4$ (1.0 equiv.) in DMF (4.0 mL), room temperature, 10 h, under Ar atmosphere. Yields were determined by ^1H NMR spectroscopy using 1,3,5-trimethoxybenzene as the internal standard.

Moreover, we conducted the electroreductive hydrogenation under conditions in the absence of $NiBr_2$·dtbpy (for details, please see entry 1 in Table 1 in the manuscript), and no obvious deposits was observed (see the pictures below, **Cathode-3**, which was similar with **Cathode-1**). And we employed Zn(+)/**Cathode-3** as electrodes under conditions in the absence of $NiBr_2$·dtbpy, no desired product was obtained.

60

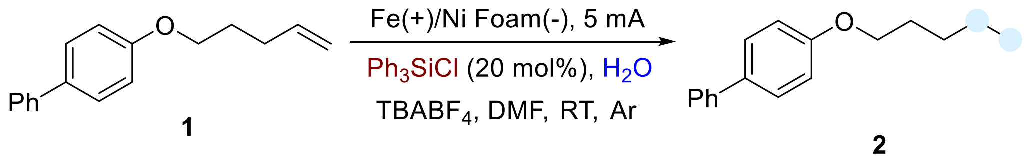

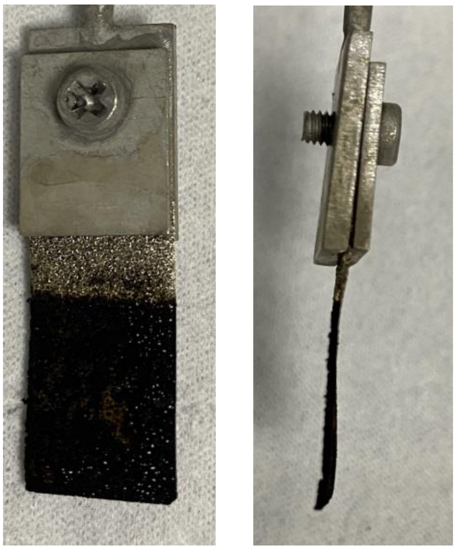

Cathode-3

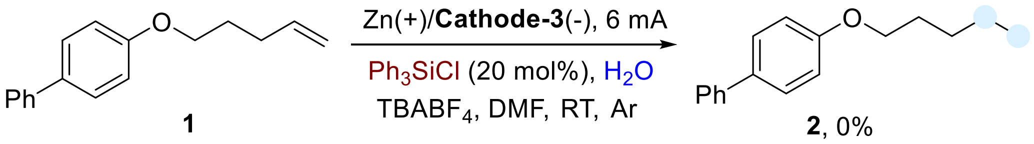

In conclusion, according to the above results, we speculated the homogeneous electroreductive hydrogenation process was the main path of this reaction. However, because the **cathode-2** could work in high efficiency in the control experiments as well, it is impossible to fully rule out a heterogeneous process proceeded on the coated Ni Foam cathode.

Moreover, we conducted PXRD, XPS and SEM-EDS mapping to investigate the Fe particles deposited on the **cathode-2**.

(1) We conducted the PXRD tests of Fe particles deposited on Ni Foam (Supplementary Figure 8), but no obvious new peak was observed, which indicating these electrolytic deposits were amorphous.

61

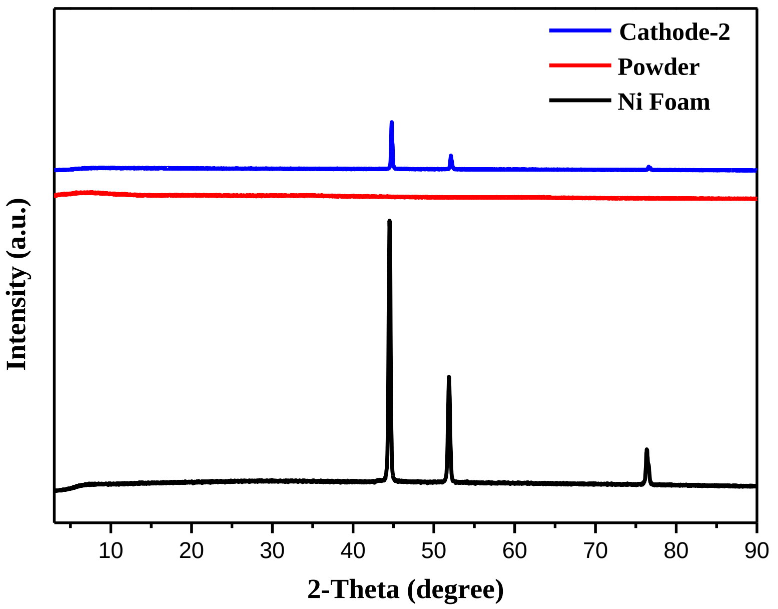

Supplementary Figure 12. PXRD patterns of Fe particles deposited on Ni Foam. a) Cathode after electrolysis (**Cathode-2**, blue line); b) Powder scraped from the surface of cathode after electrolysis (red line); c) New Ni Foam electrode (black line).

(2) XPS technique was further used to analyze the valance state of Fe particles on the cathode (Ni Foam) after electrolysis. The high resolution of spectra was shown in Supplementary Figure 13. The fitted peaks of 706.40 and 719.50 eV indicated the existence of Fe(0), and the peaks at 709.10 and 723.20 eV corresponded to Fe(II). The peaks at 711.10 and 728.20 eV corresponded to Fe(III).

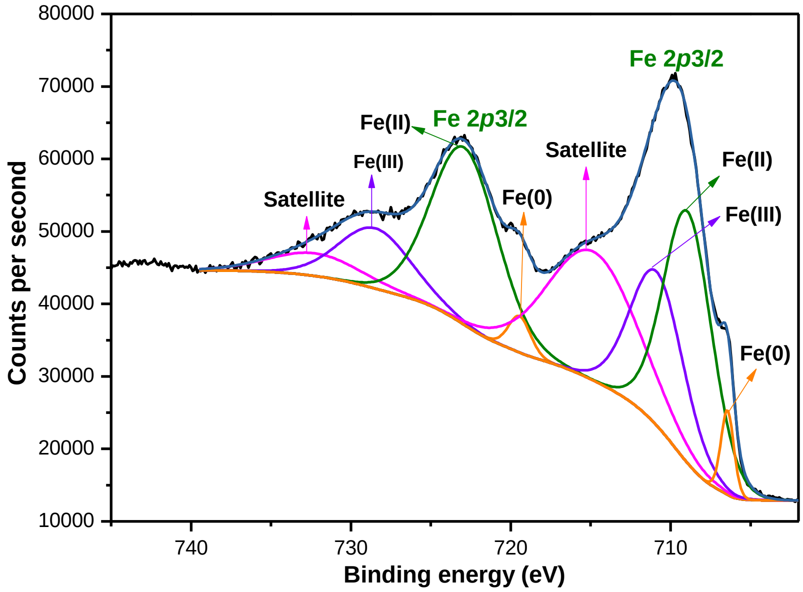

Supplementary Figure 13. XPS spectra of deposits on the surface of Ni Foam

62

(3) The SEM images indicated the morphology of deposits on the surface of the cathode was amorphous (Supplementary Figure 14).

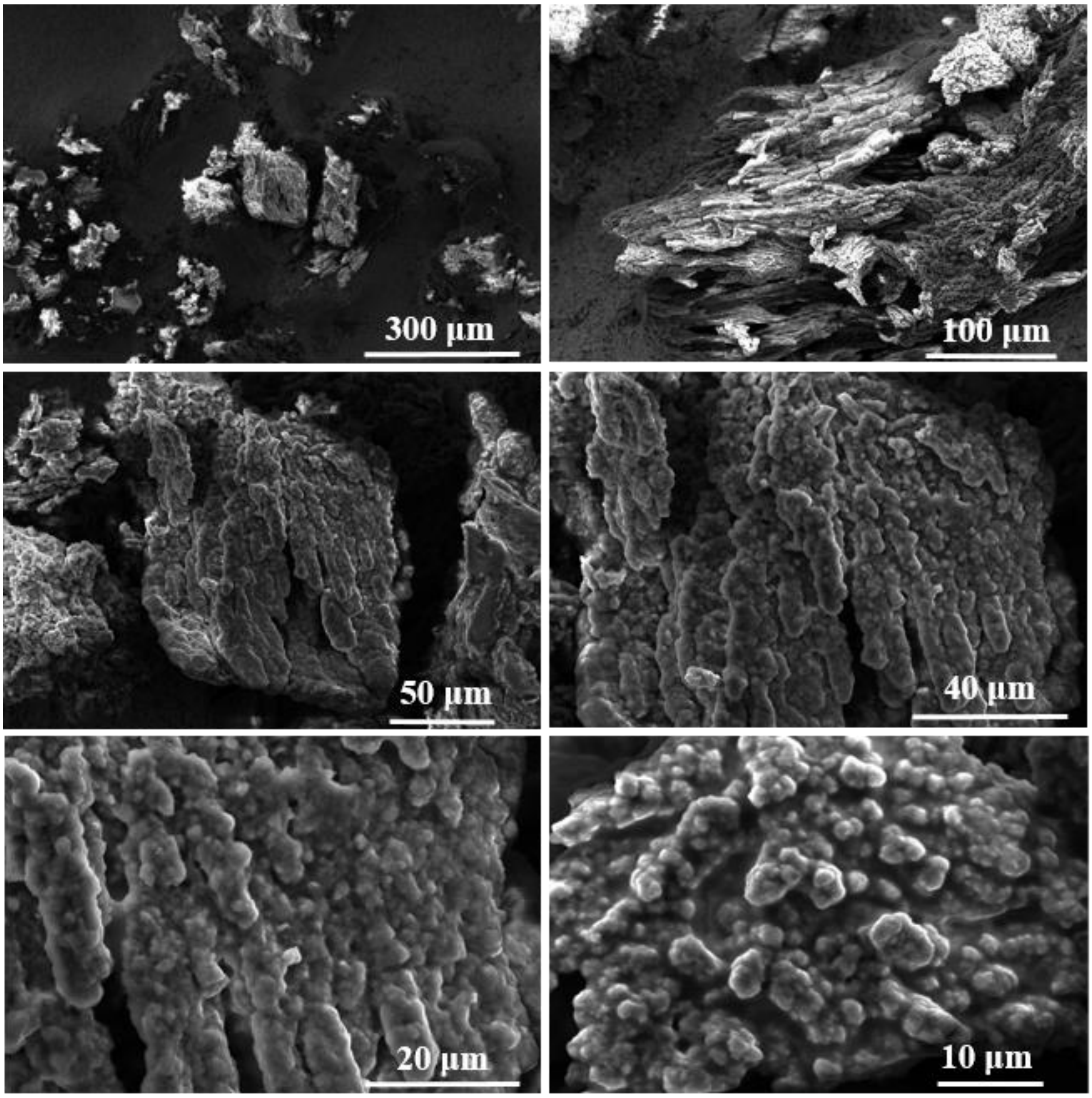

Supplementary Figure 14. SEM images of deposits on the surface of Ni Foam

And SEM EDS mapping revealed C, O and Fe signal localized in the deposits on the surface of Ni Foam (Supplementary Figure 15).

63

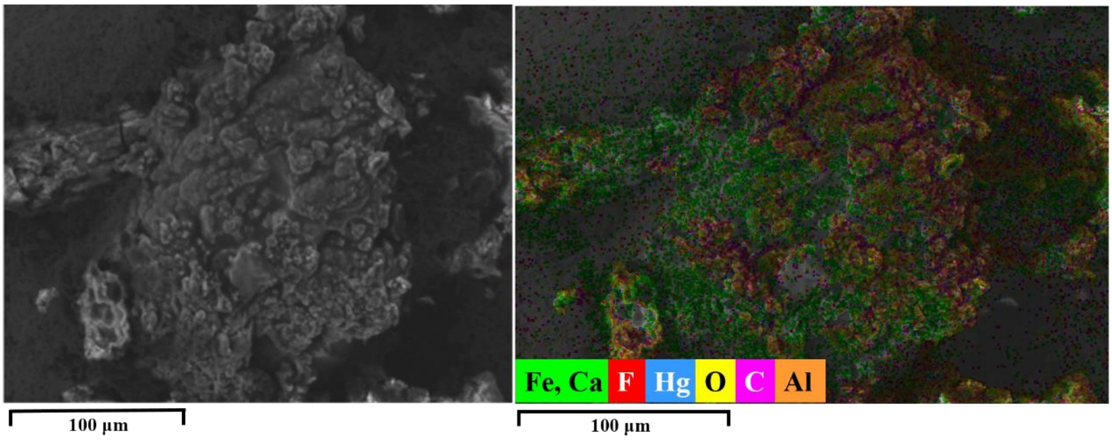
Fe, Ca
F
Hg
O
C
Al
100 µm
100 µm

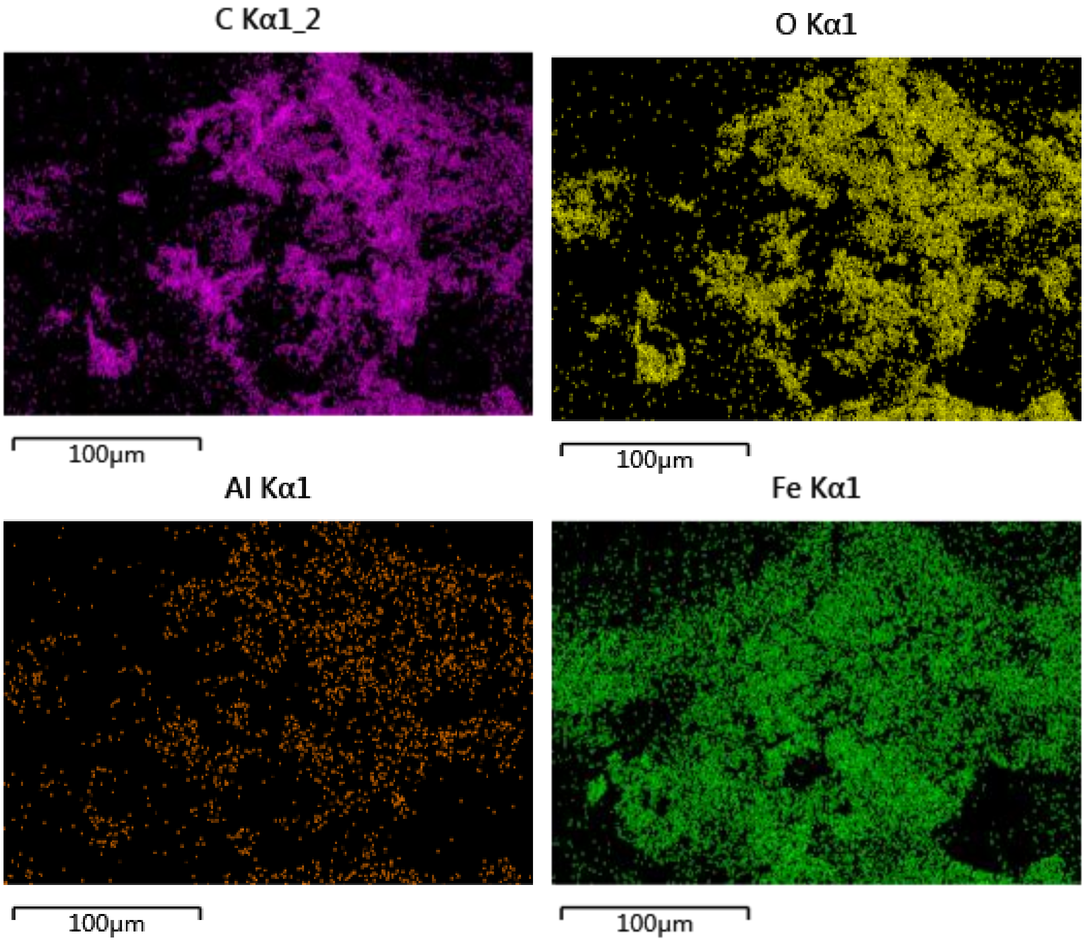
C Kα1_2
O Kα1
100µm
100µm
Al Kα1
Fe Kα1
100µm
100µm

64

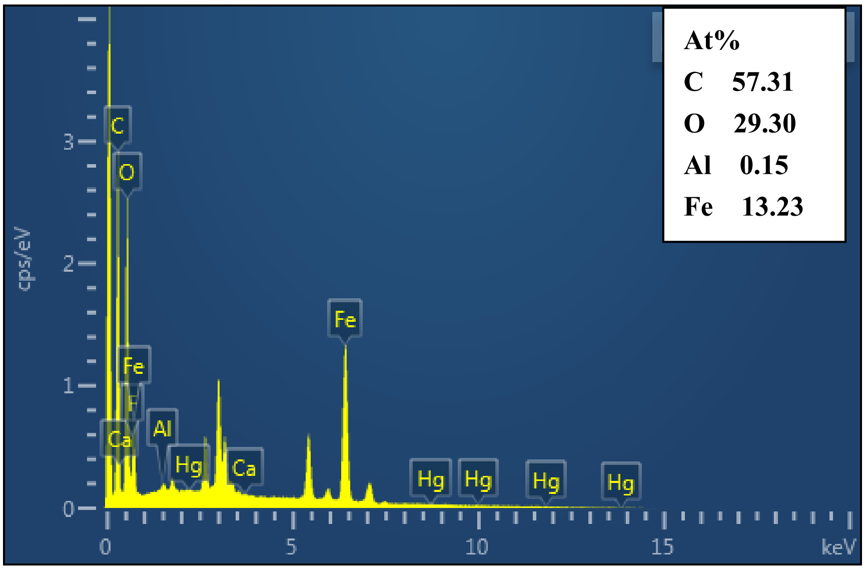

Supplementary Figure 15. SEM EDS mapping showing the C, O and Fe signal localized in the deposits on the surface of Ni Foam

In conclusion, the deposits on the surface of cathode after electrolysis were amorphous, and C, O and Fe signal localized in the deposits. The valence of Fe particles were including Fe(0), Fe(II) and Fe(III).

65

2.2 Cyclic voltammetry

The cyclic voltammetry was carried out with a Shanghai Chenhua CHI760E workstation. A glassy-carbon electrode (3mm-diameter, disc-electrode) was used as the working electrode, a Pt plate was used as the auxiliary electrode and an Ag/Ag^{+} electrode was used as a reference electrode. All of the samples should be bubbled with Ar for 5 min before testing. The measurements were carried out at a scan rate of 100 mV s^{-1} in solvent/$^{n}Bu_4NBF_4$ (0.1 M).

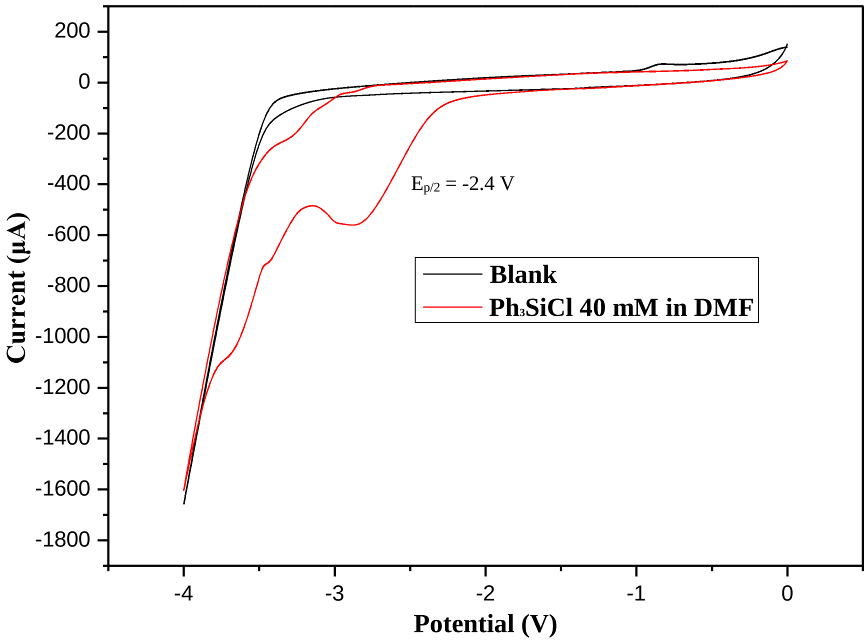

Supplementary Figure 16. Cyclic voltammograms of Ph_3SiCl (40 mM) in DMF/$^{n}Bu_4NBF_4$ (0.1 M) under Ar atmosphere.

66

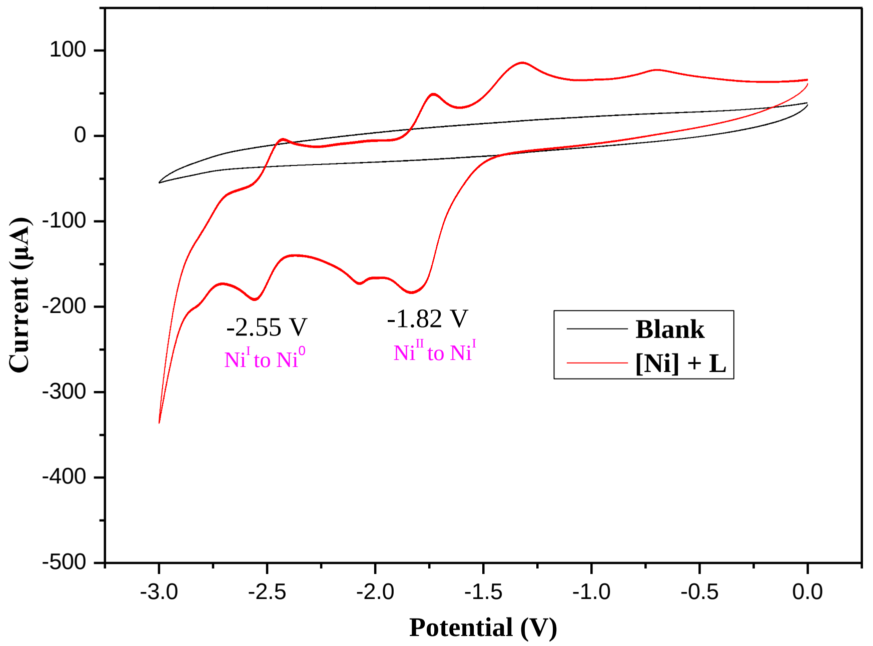

Supplementary Figure 17. Cyclic voltammograms of $NiBr_2$·dme and 4,4'-Di-tert-butyl-2,2'-bipyridine (**L**) in DMF/^{n}Bu_4NBF_4 (0.1 M) under Ar atmosphere.

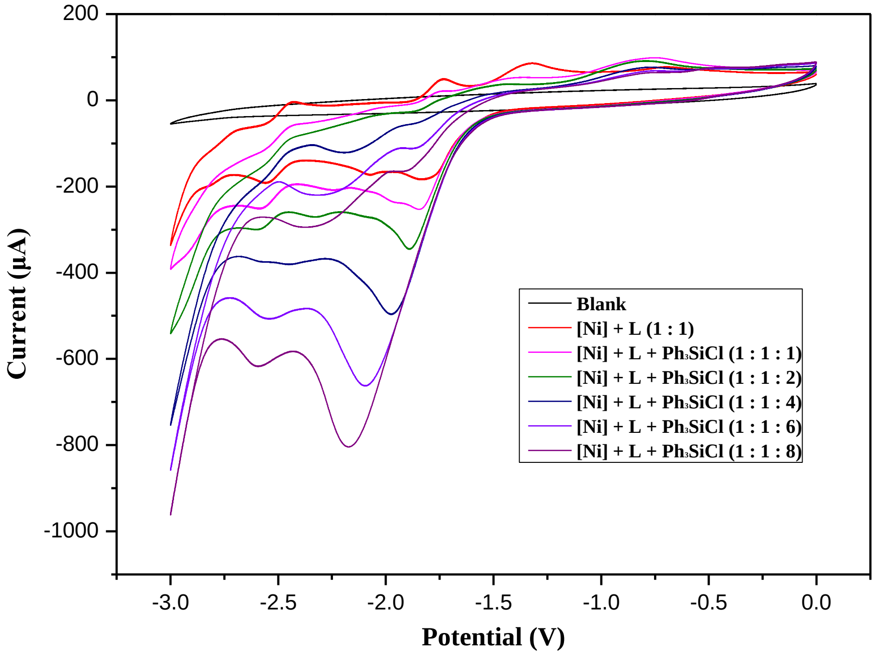

Supplementary Figure 18. Cyclic voltammograms of $NiBr_2$·dme and 4,4'-Di-tert-butyl-2,2'-bipyridine (**L**) performed in the presence of increasing equivalents of Ph_3SiCl.

67

2.3 Copies of ^{1}H, ^{13}C and ^{19}F NMR spectra for compounds

^{1}H NMR spectrum of **2** (**$CDCl_3$**)

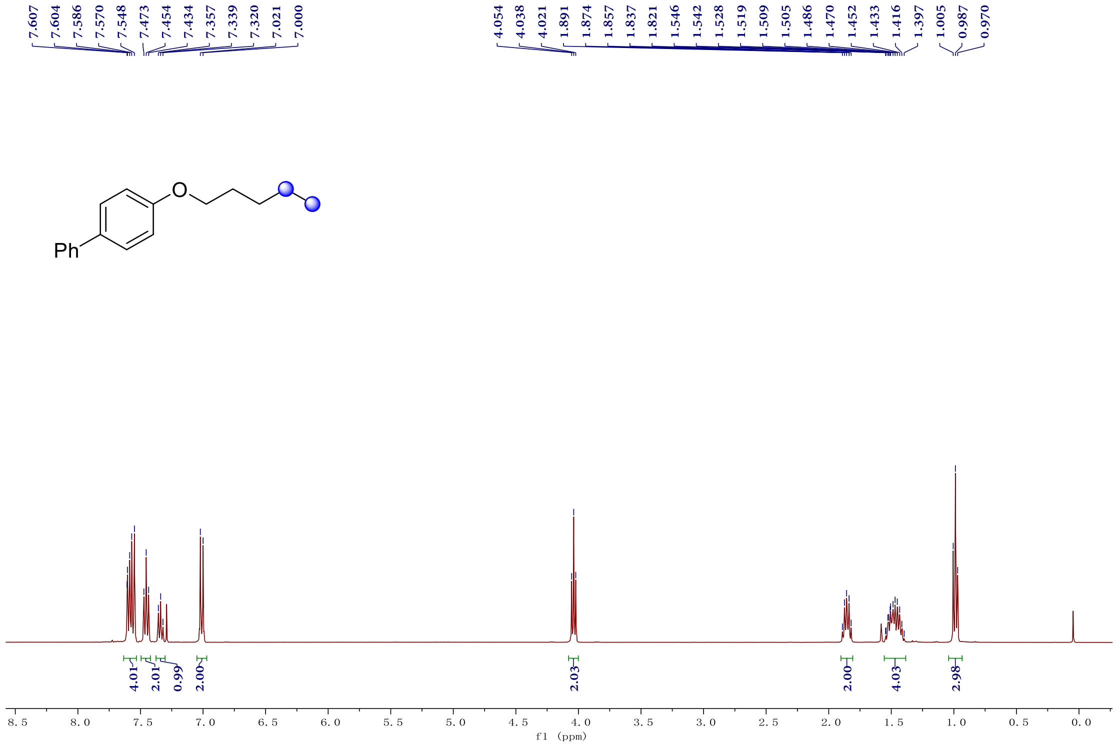

^{13}C NMR spectrum of **2** (**$CDCl_3$**)

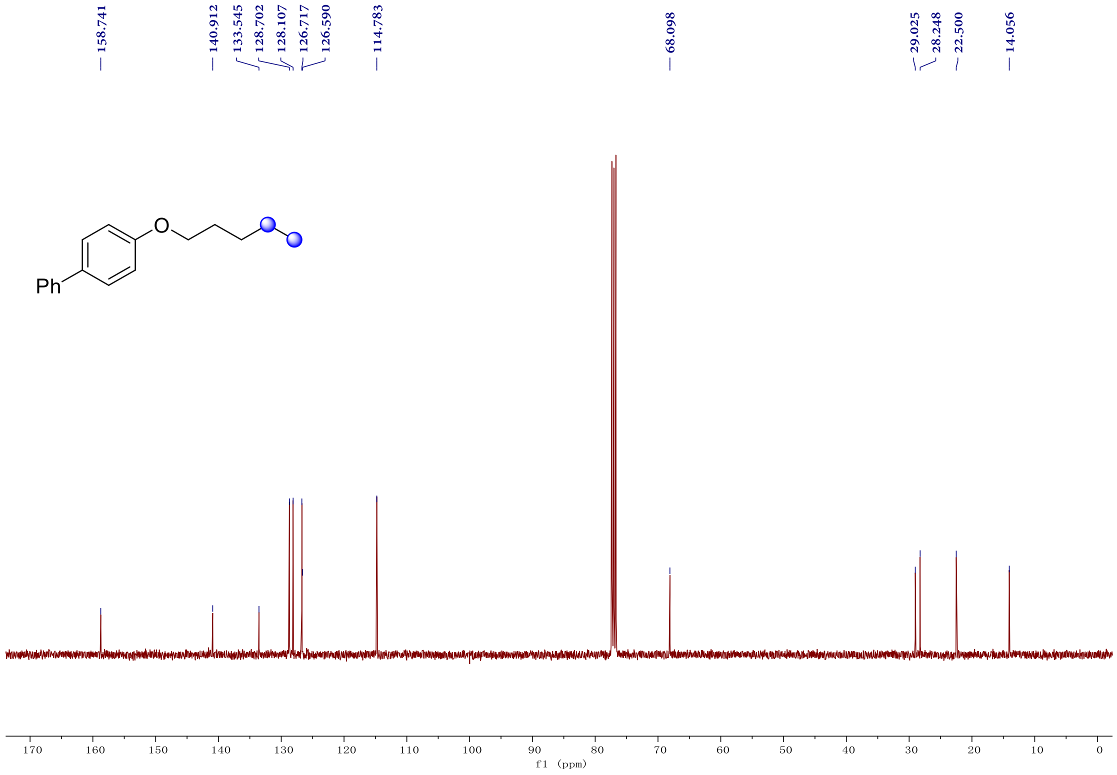

68

^1H NMR spectrum of **3** (**$CDCl_3$**)

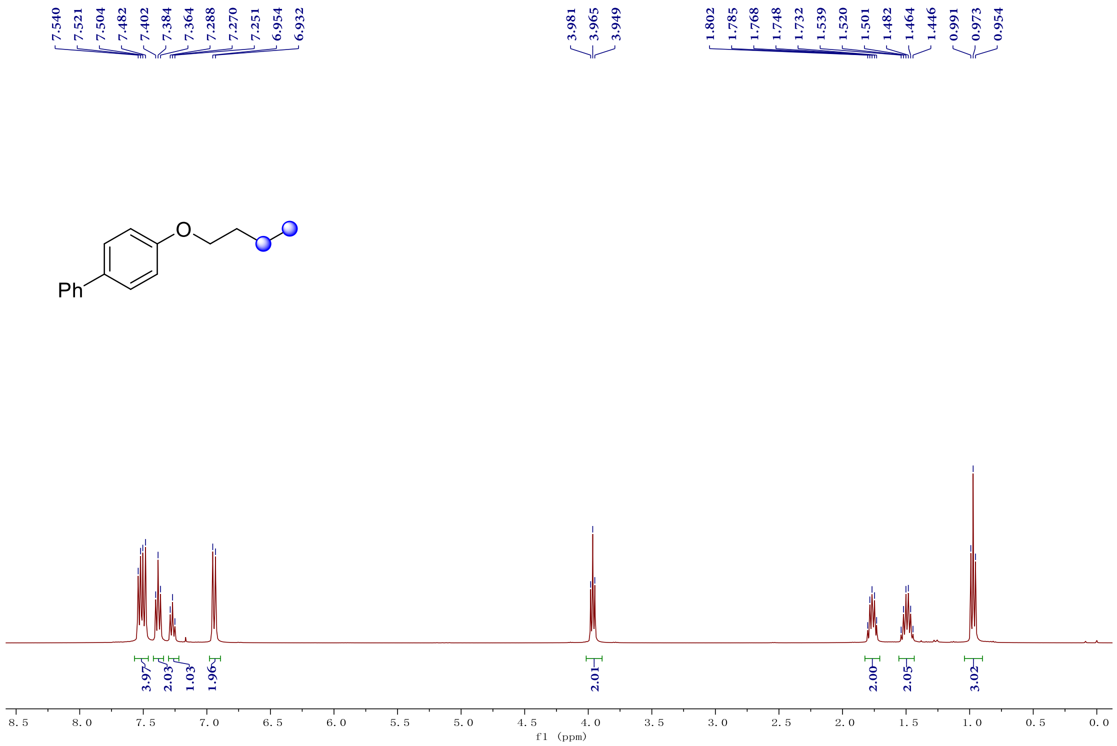

^{13}C NMR spectrum of **3** (**$CDCl_3$**)

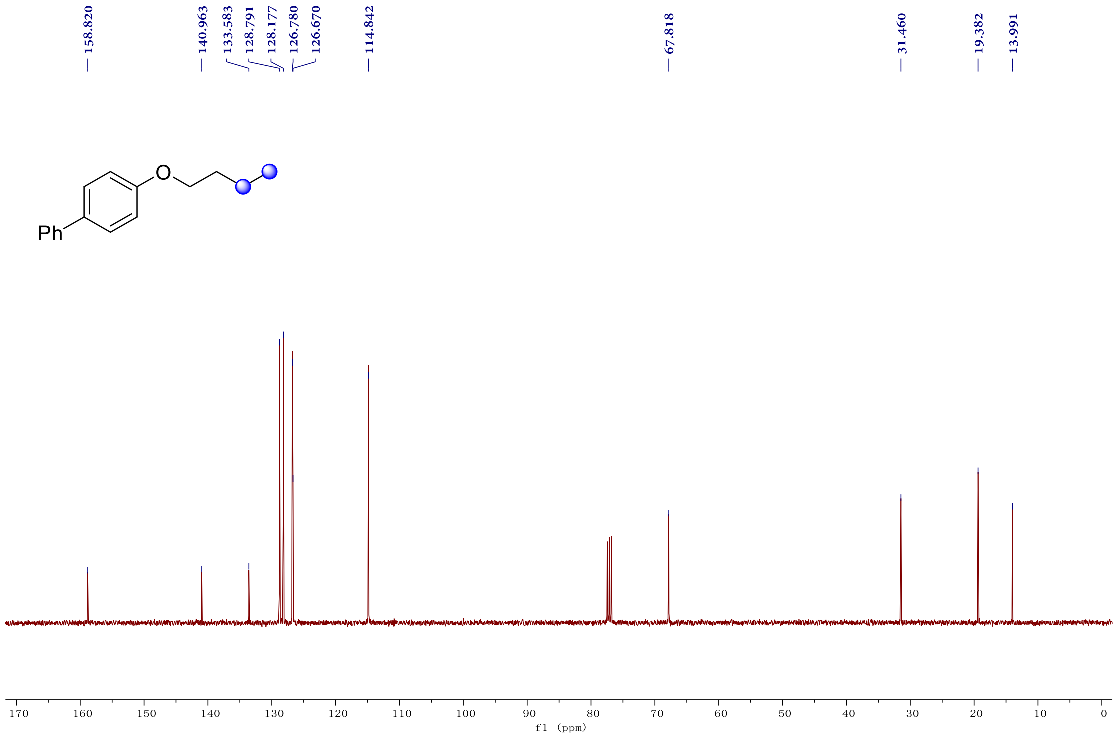

^{1}H NMR spectrum of **4** (**$CDCl_3$**)

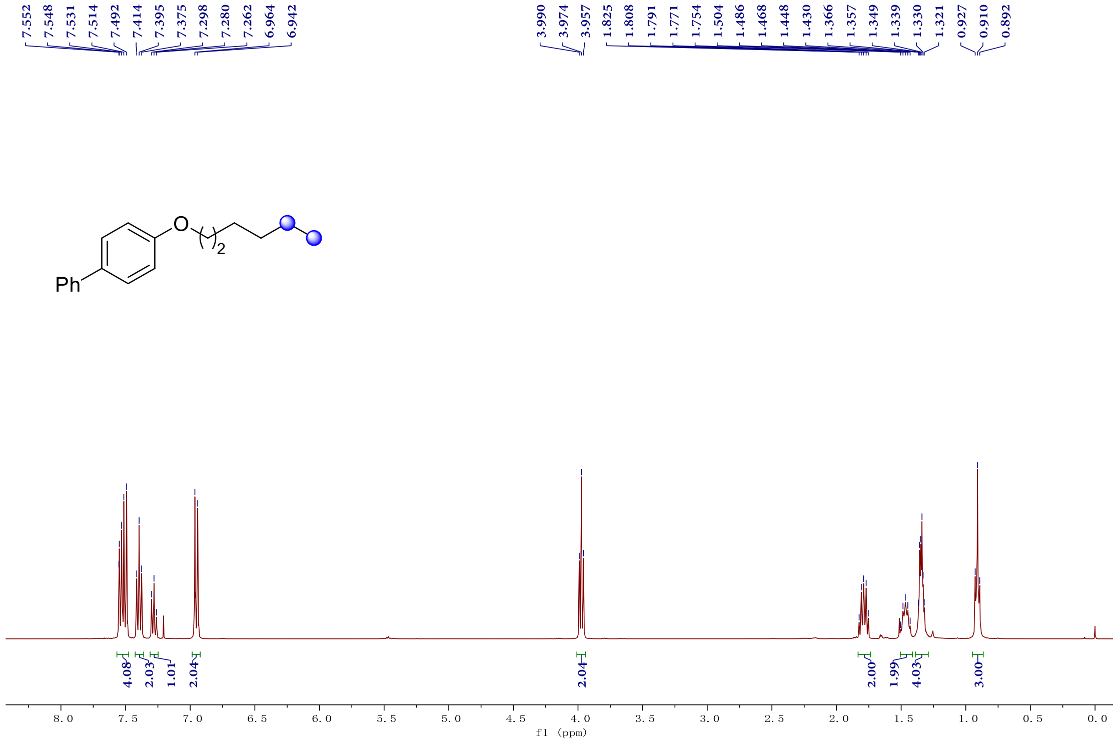

^{13}C NMR spectrum of **4** (**$CDCl_3$**)

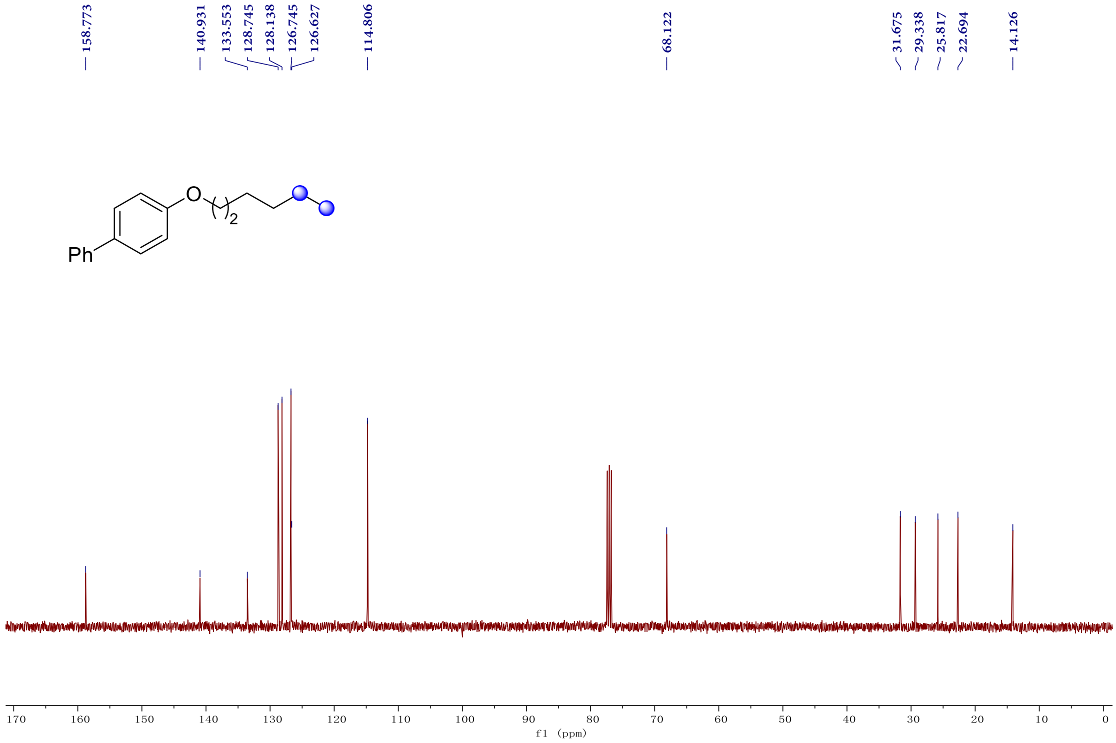

70

^{1}H NMR spectrum of **5** (**CDCl$_3$**)

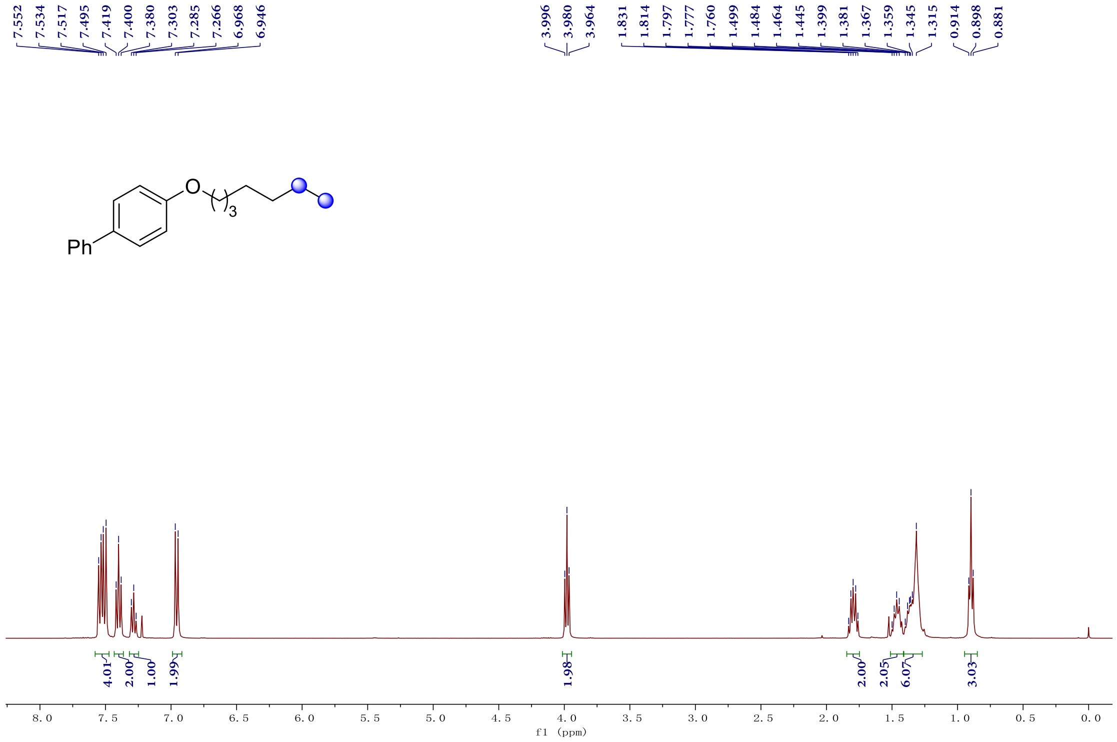

^{13}C NMR spectrum of **5** (**CDCl$_3$**)

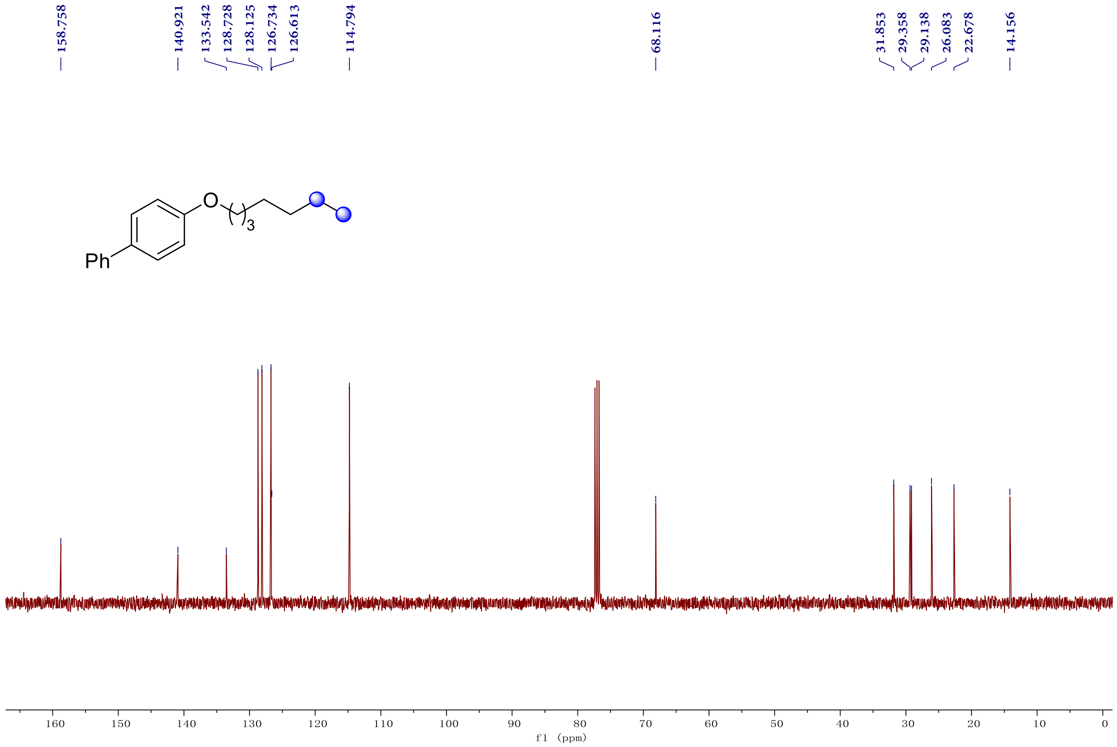

71

^{1}H NMR spectrum of **6** (**$CDCl_3$**)

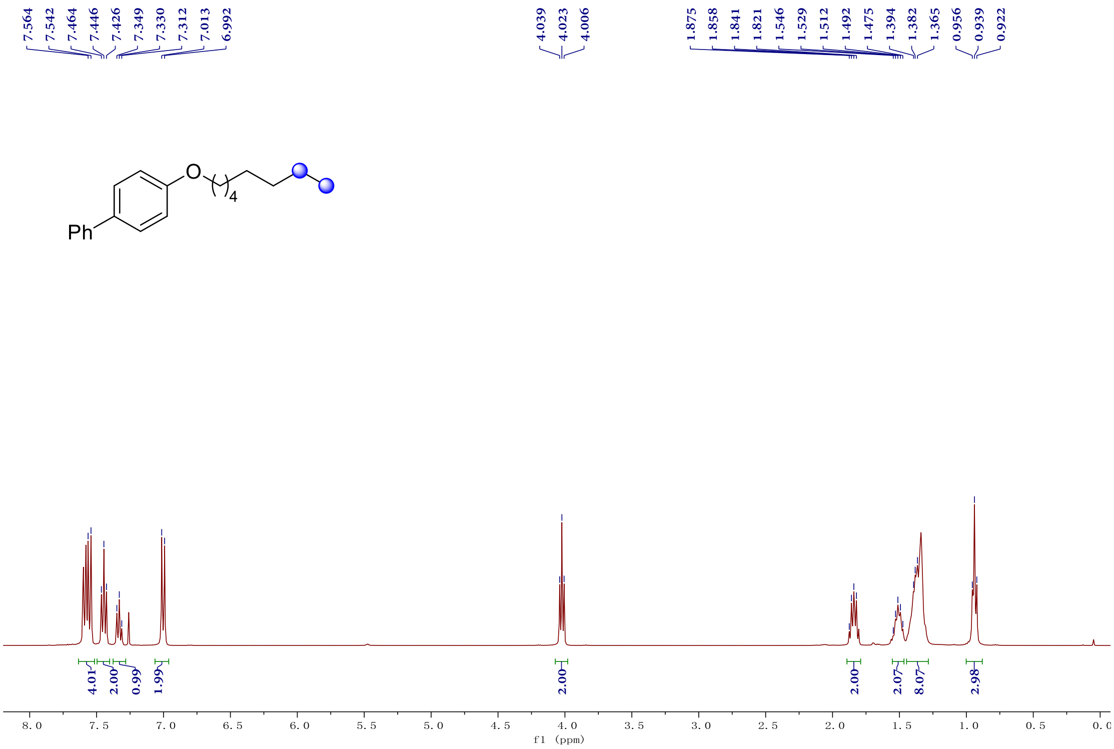

^{13}C NMR spectrum of **6** (**$CDCl_3$**)

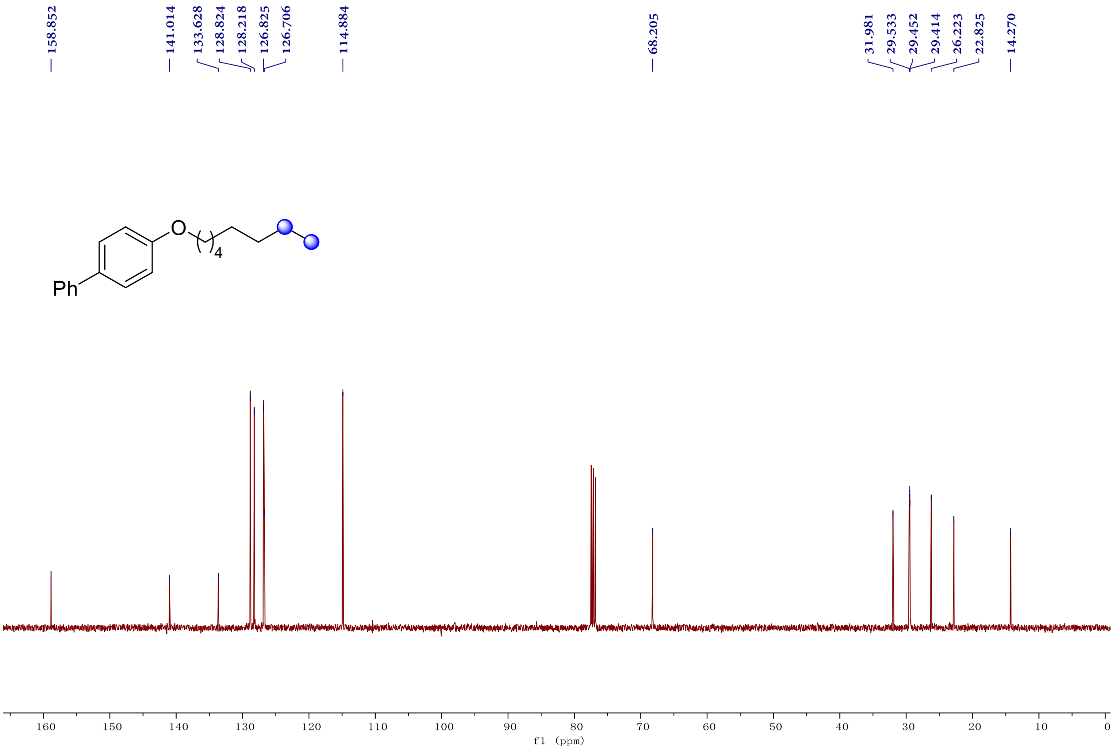

72

^{1}H NMR spectrum of **7** (**CDCl$_3$**)

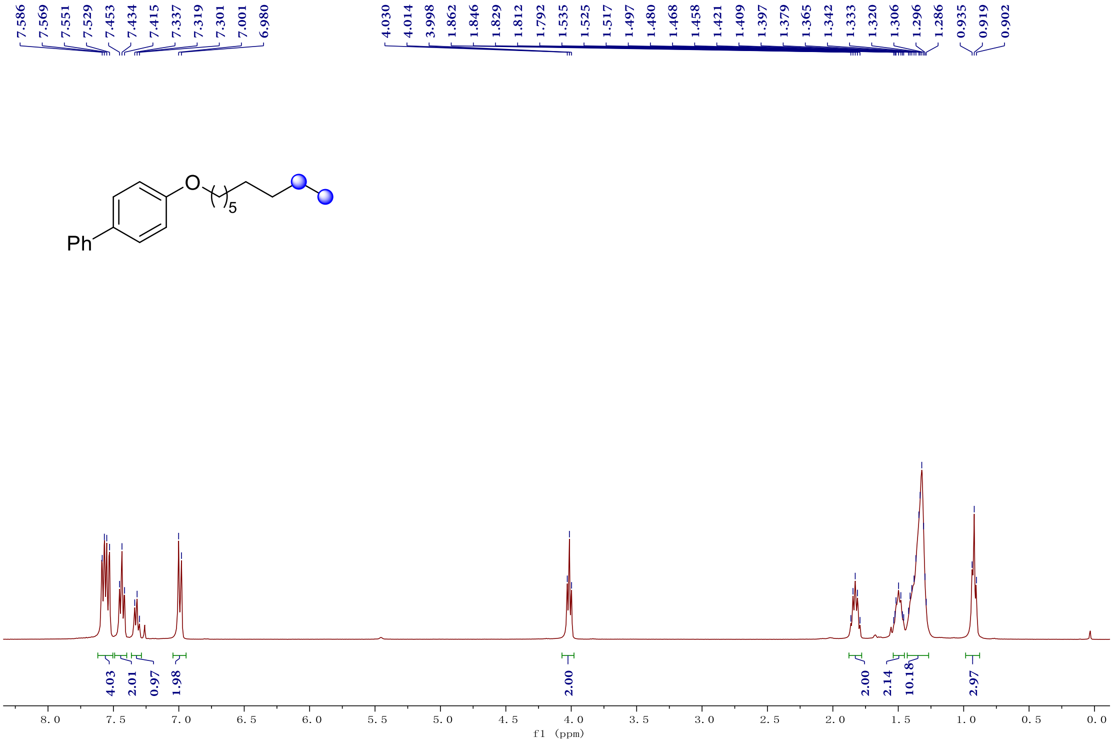

^{13}C NMR spectrum of **7** (**CDCl$_3$**)

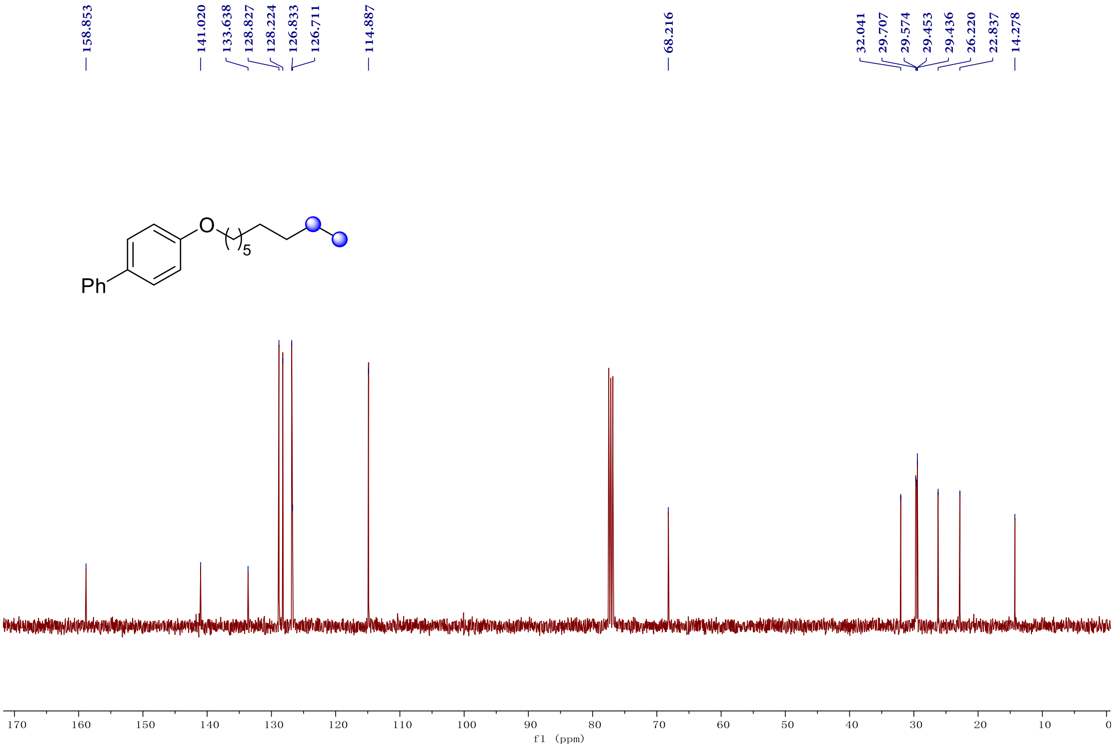

^{1}H NMR spectrum of **8** (**$CDCl_3$**)

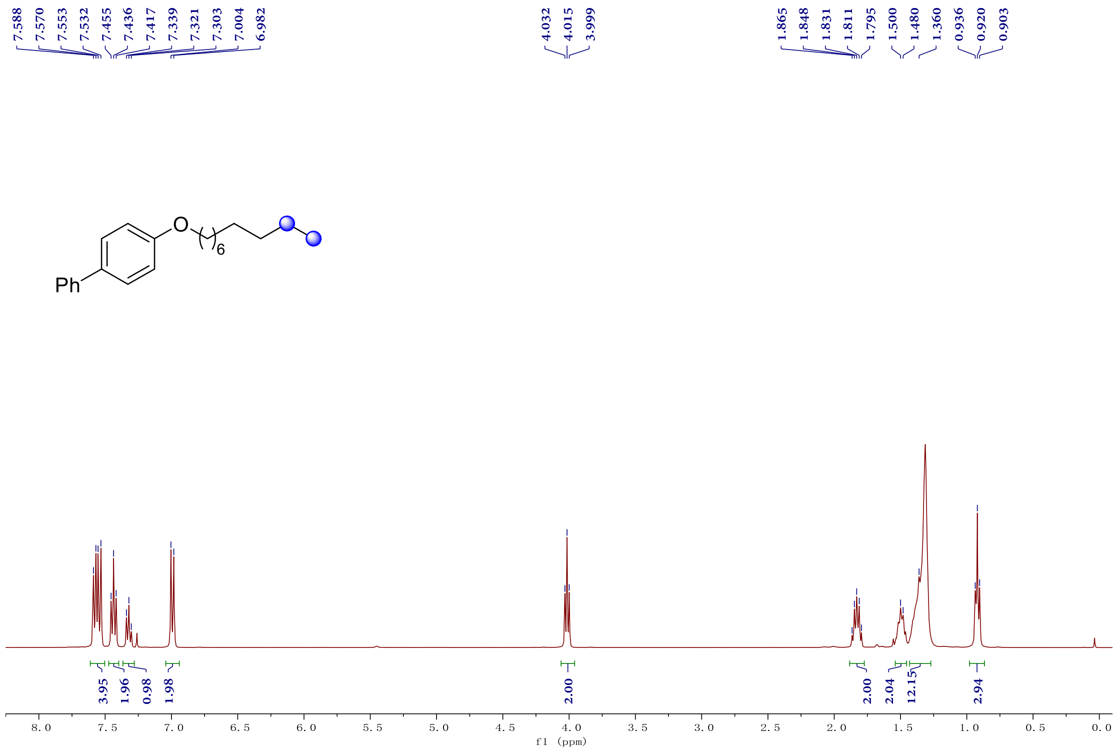

^{13}C NMR spectrum of **8** (**$CDCl_3$**)

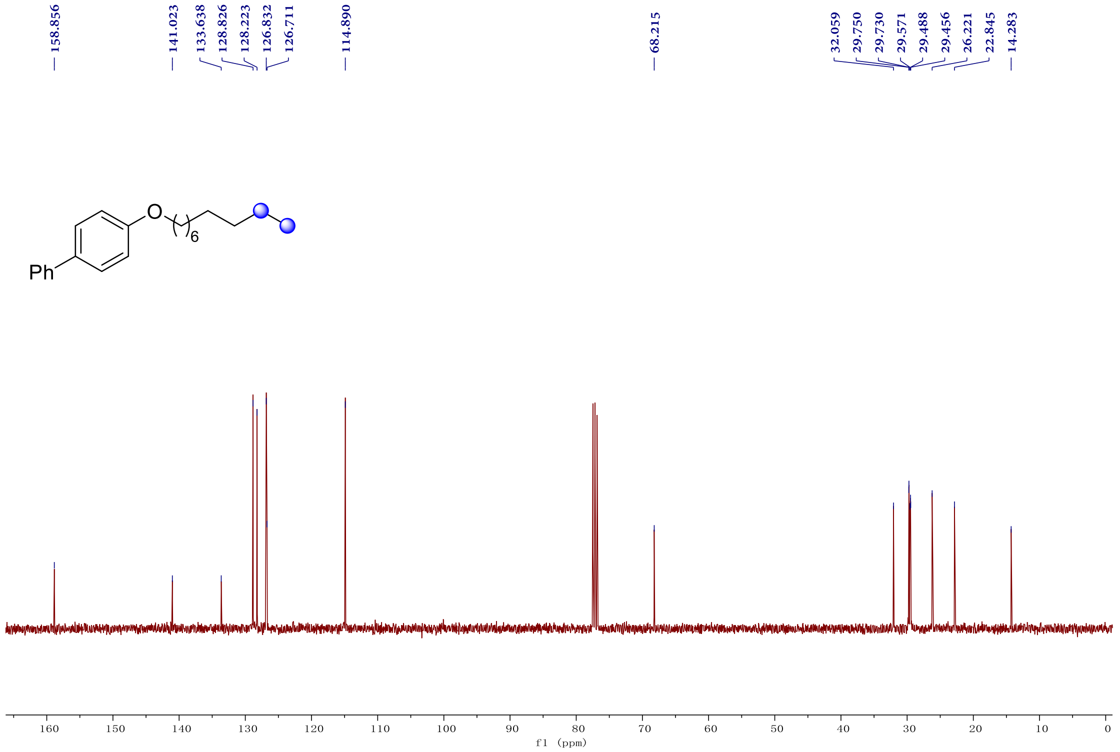

74

^{1}H NMR spectrum of **9** (**$CDCl_3$**)

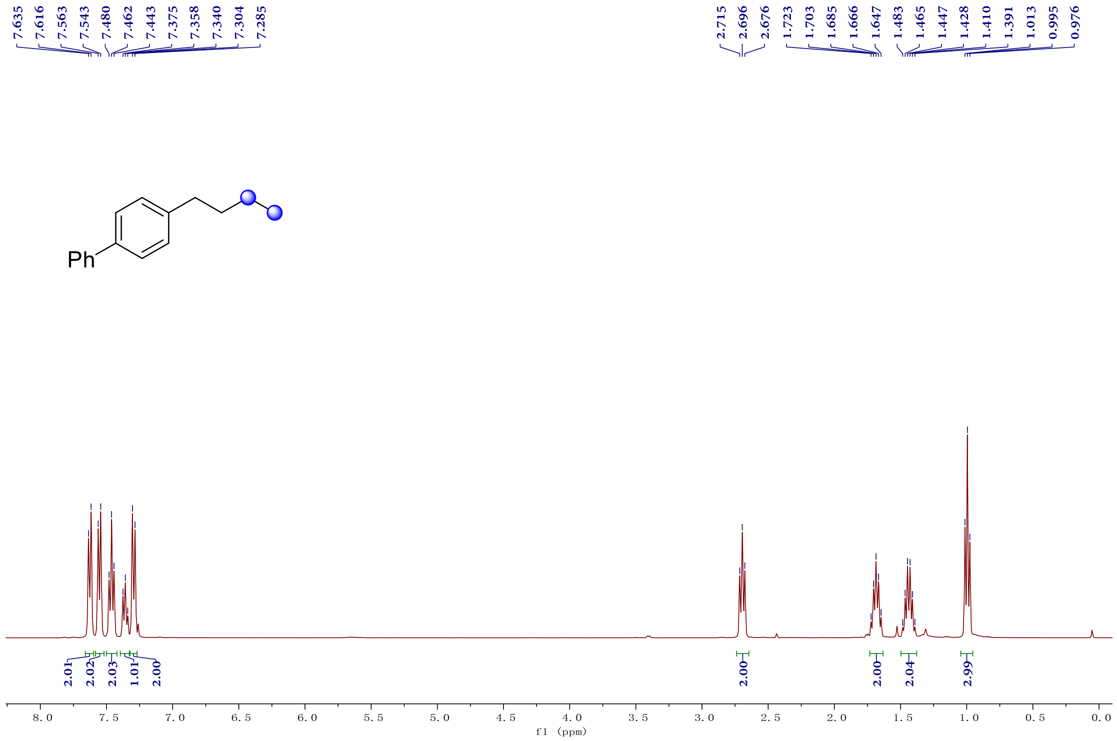

^{13}C NMR spectrum of **9** (**$CDCl_3$**)

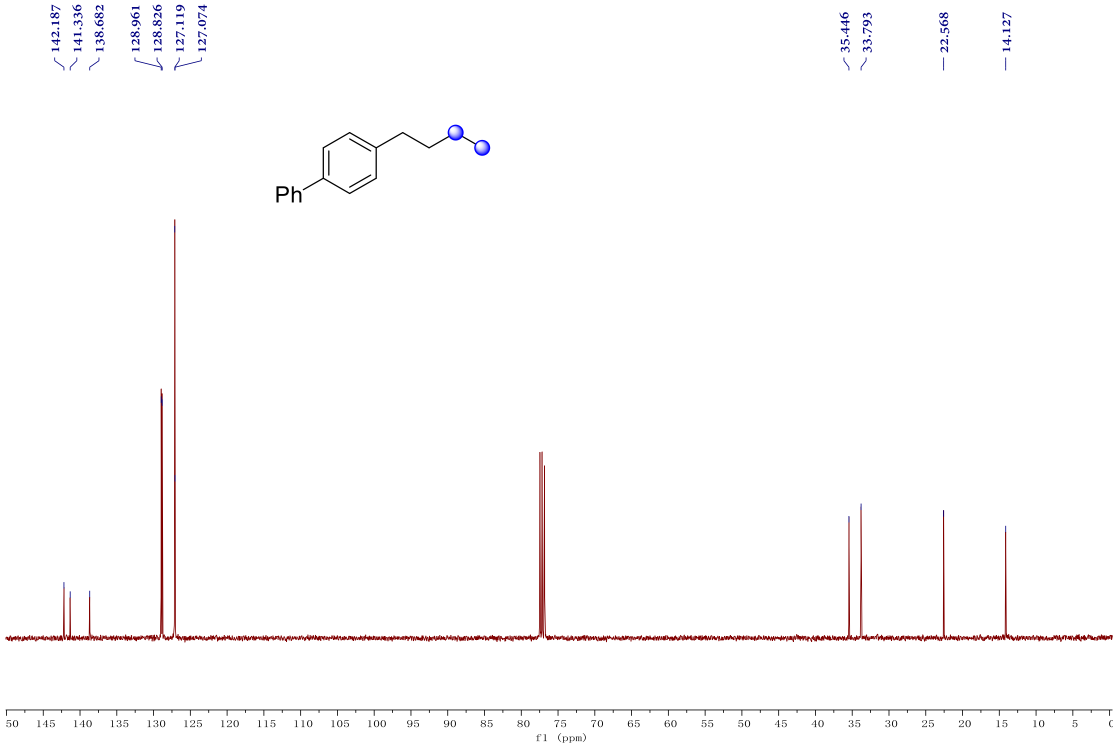

75

^{1}H NMR spectrum of **10** (**$CDCl_3$**)

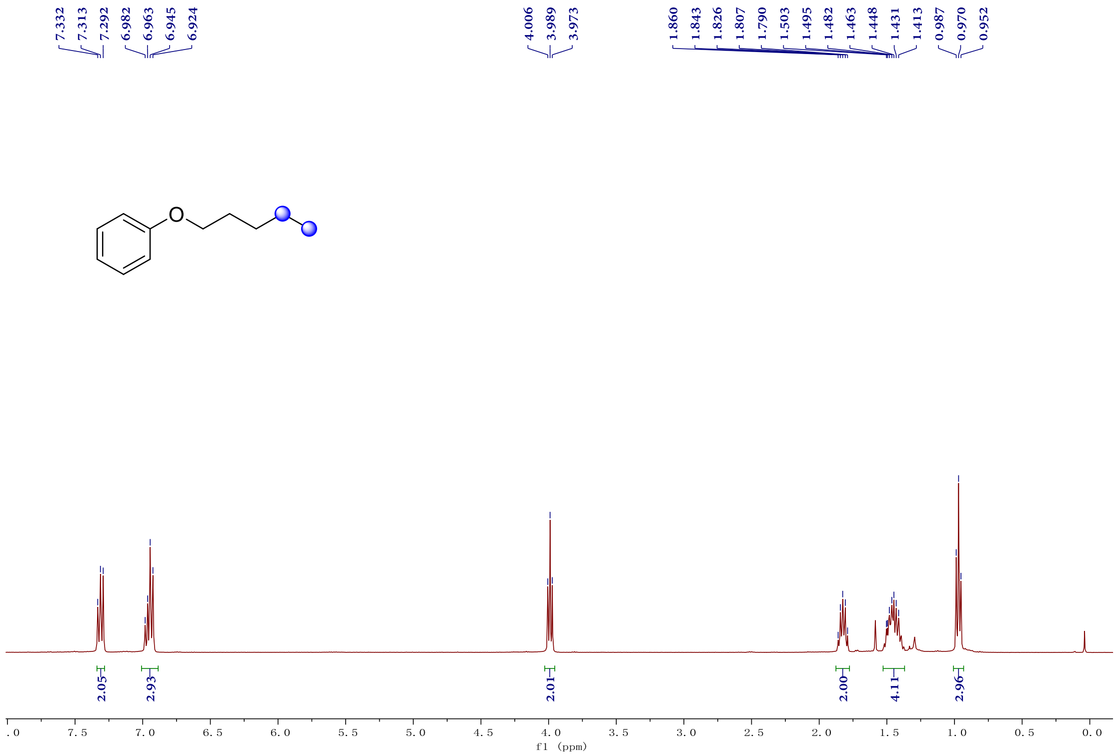

^{13}C NMR spectrum of **10** (**$CDCl_3$**)

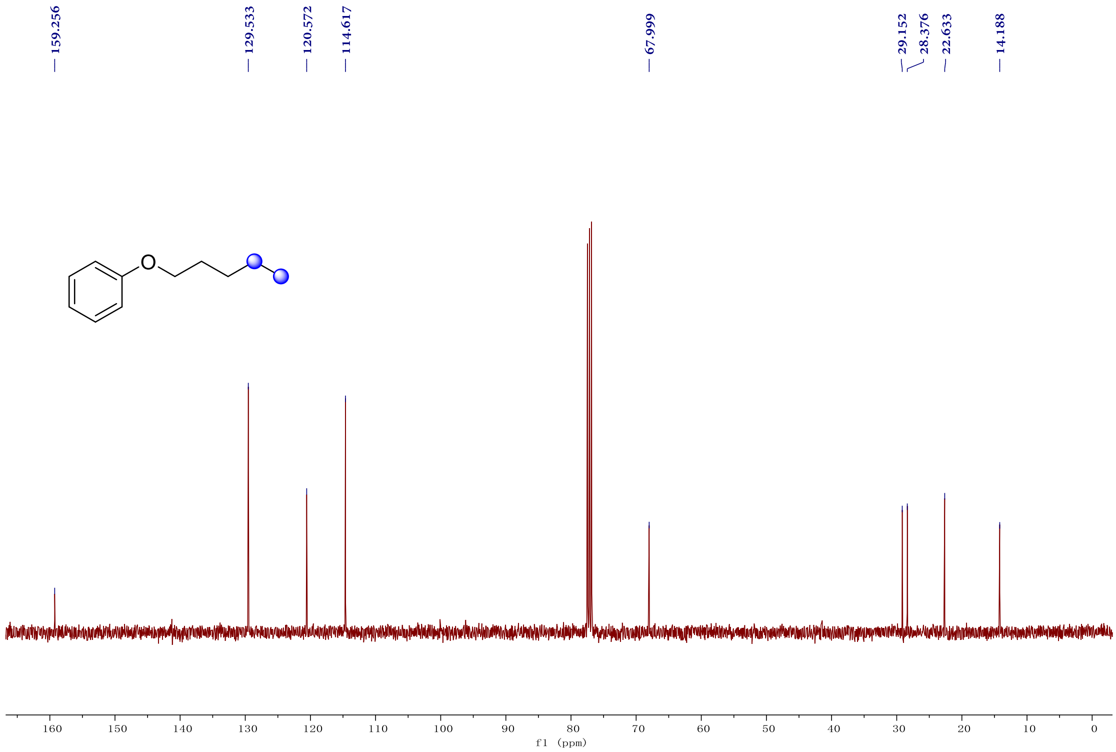

76

^{1}H NMR spectrum of **11** (**$CDCl_3$**)

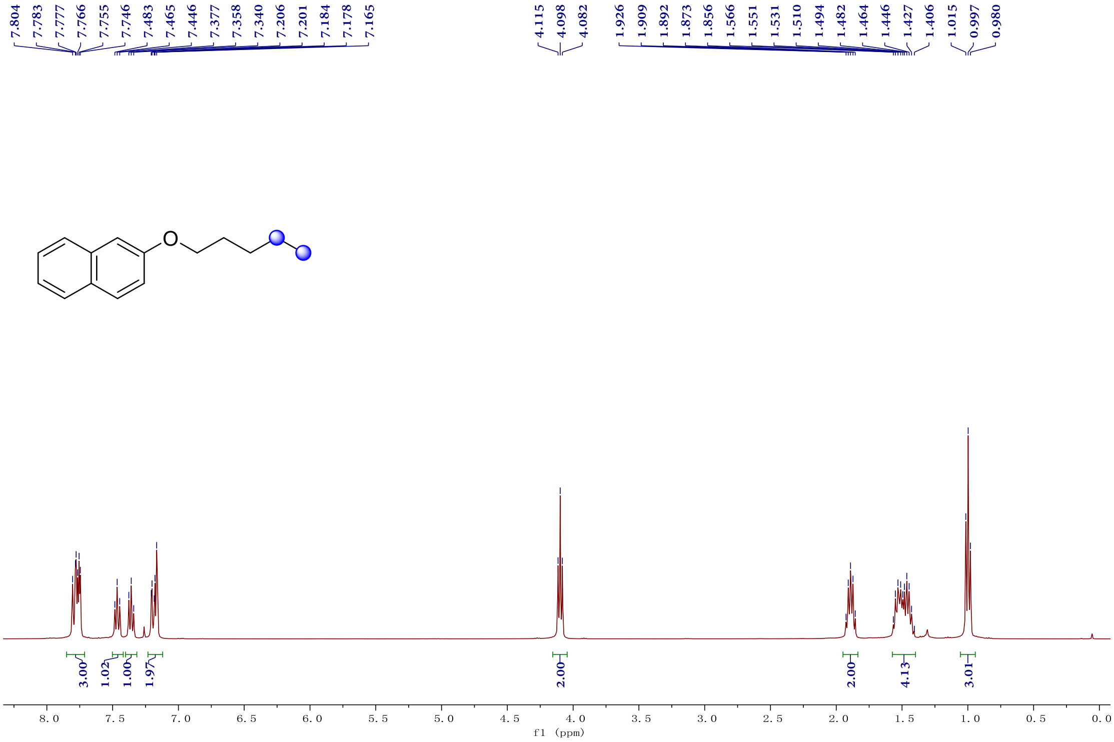

^{13}C NMR spectrum of **11** (**$CDCl_3$**)

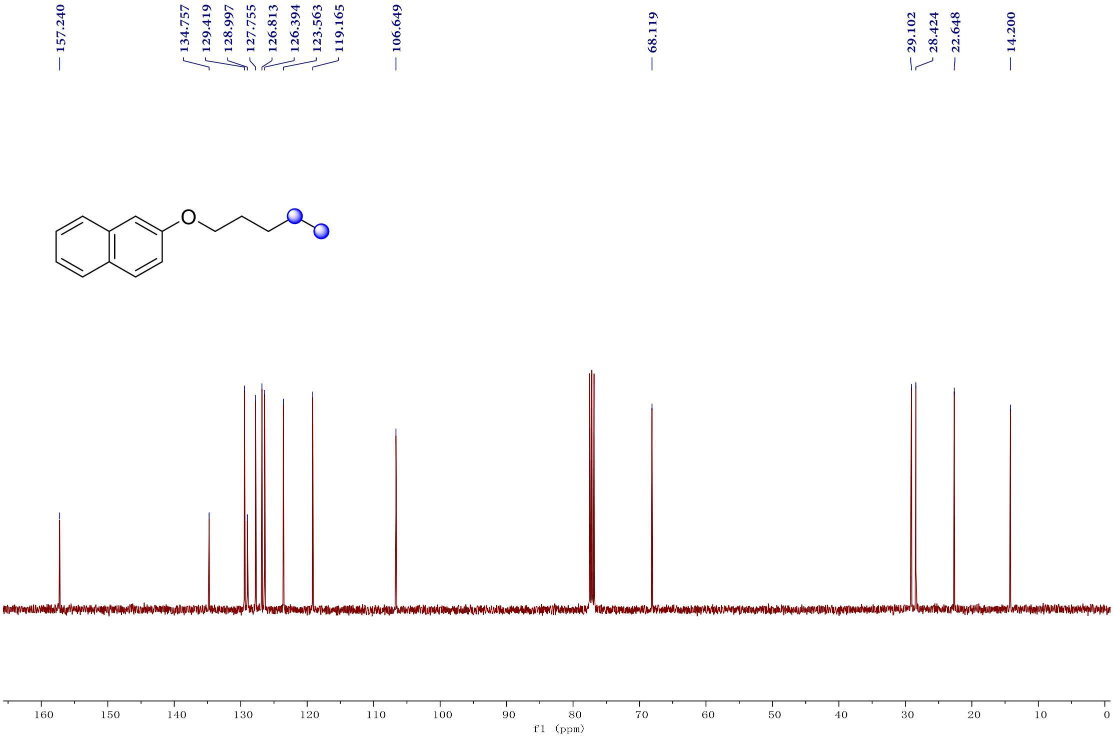

77

^{1}H NMR spectrum of **12** (**CDCl$_3$**)

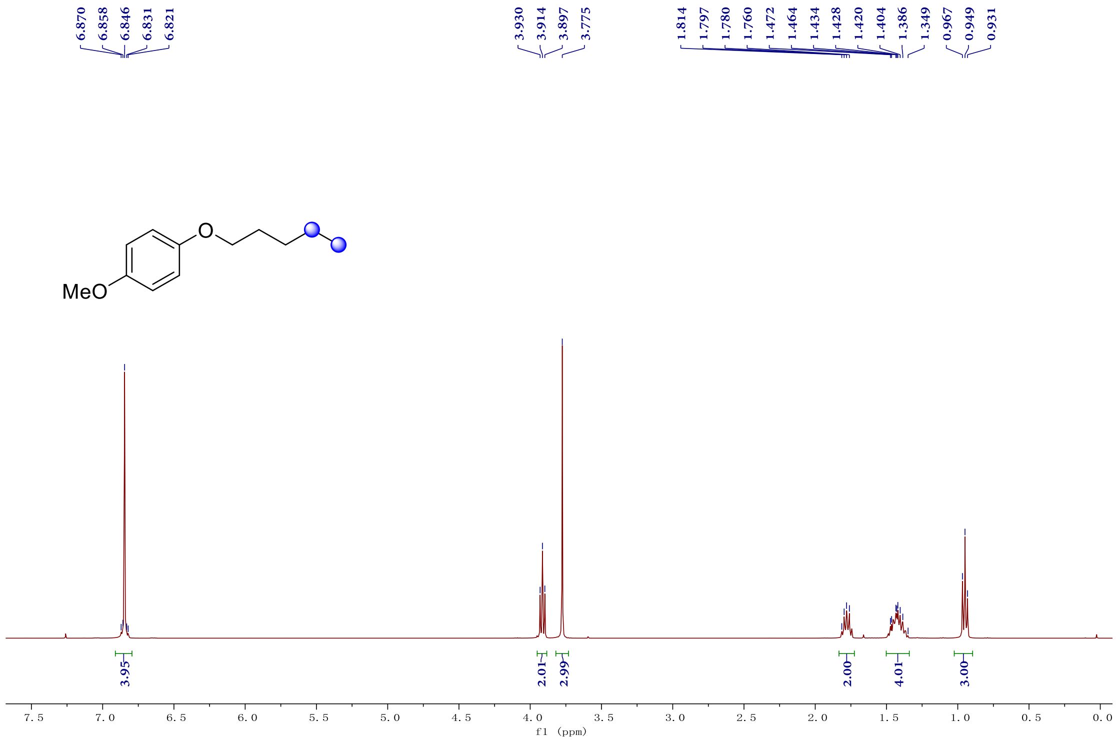

^{13}C NMR spectrum of **12** (**CDCl$_3$**)

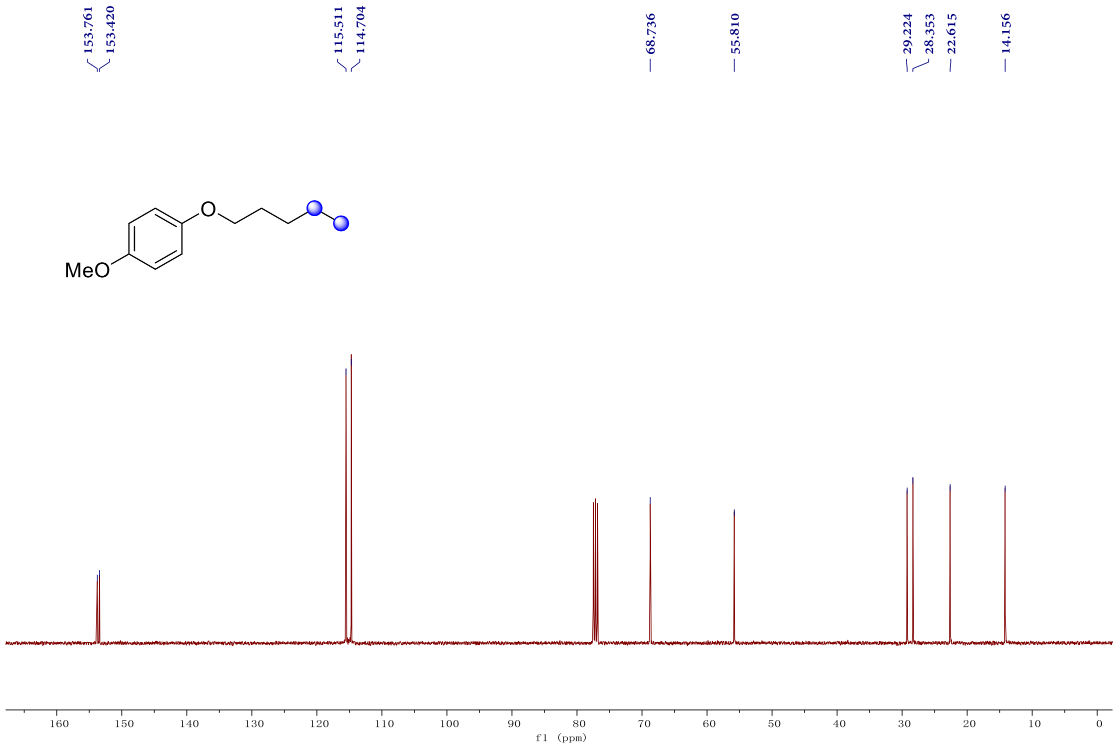

78

^{1}H NMR spectrum of **13** (**$CDCl_3$**)

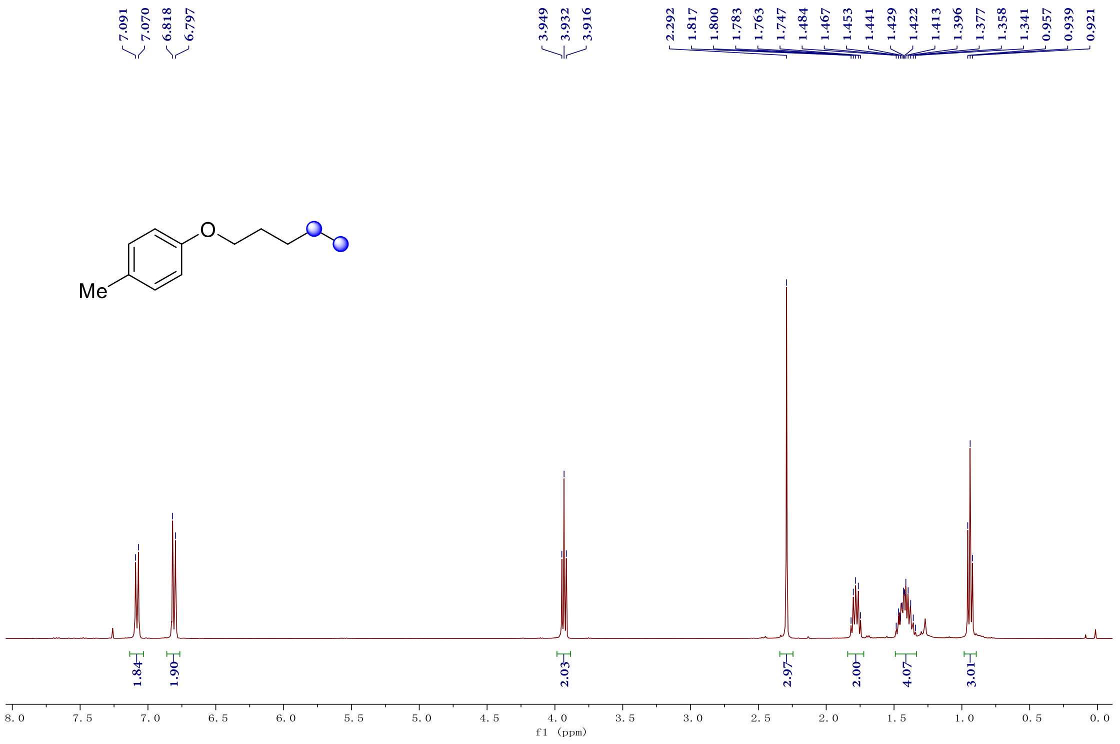

^{13}C NMR spectrum of **13** (**$CDCl_3$**)

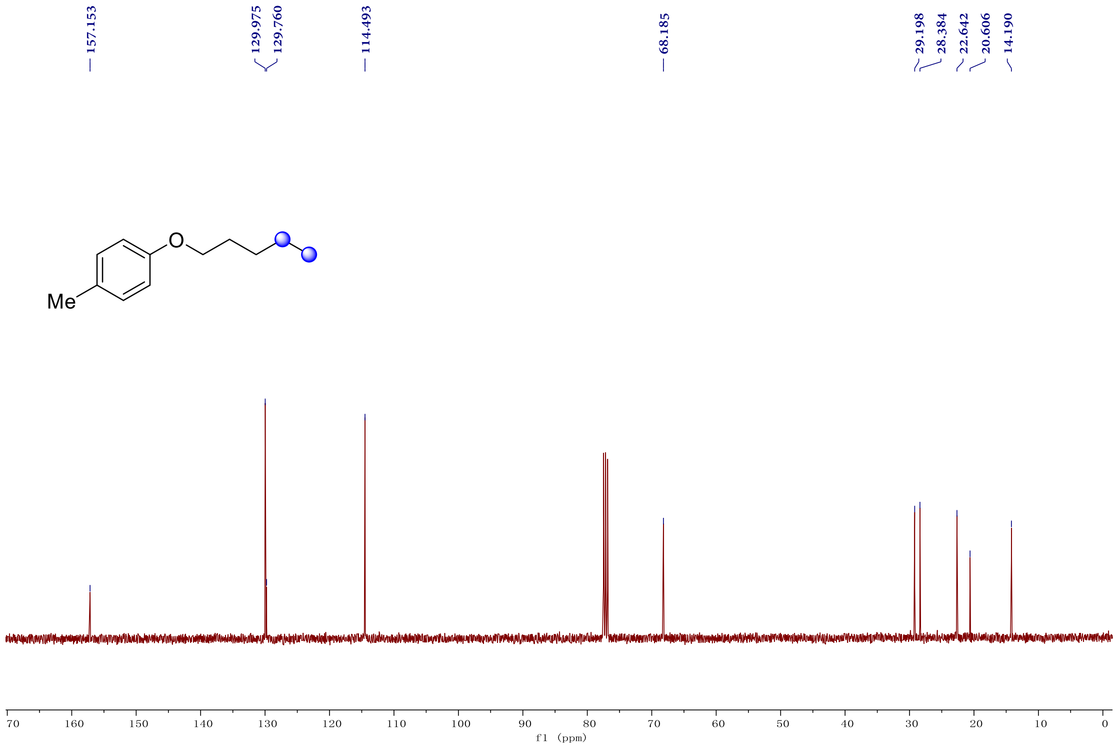

79

^{1}H NMR spectrum of **14** (**$CDCl_3$**)

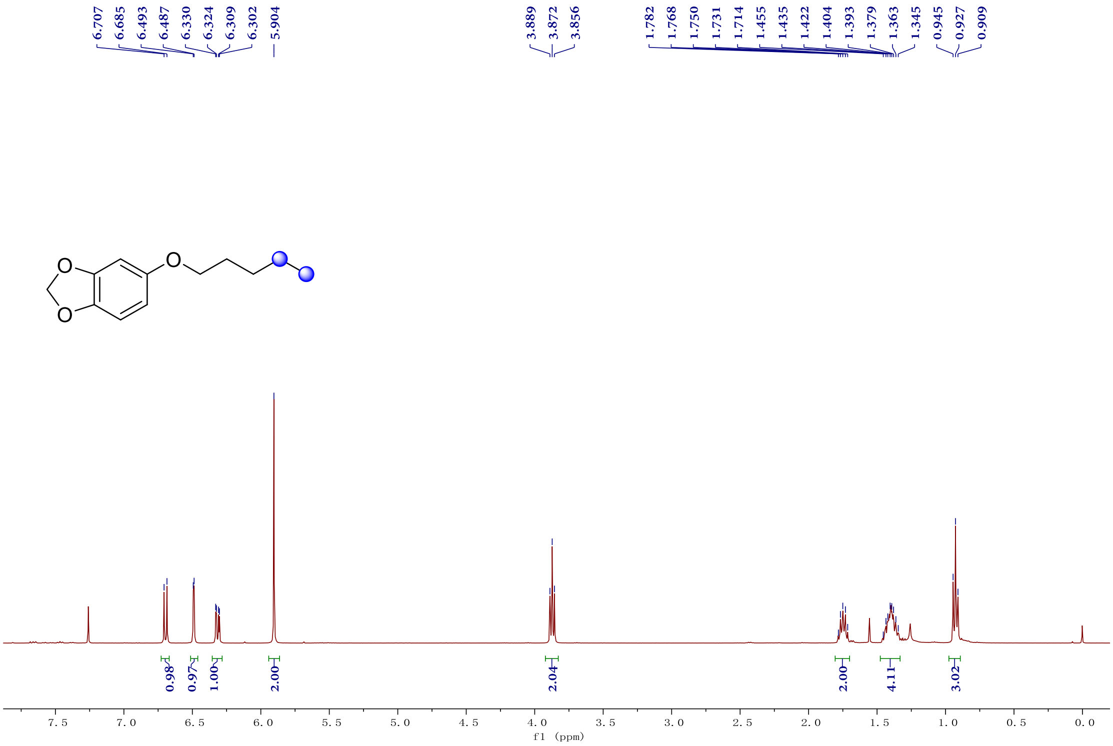

^{13}C NMR spectrum of **14** (**$CDCl_3$**)

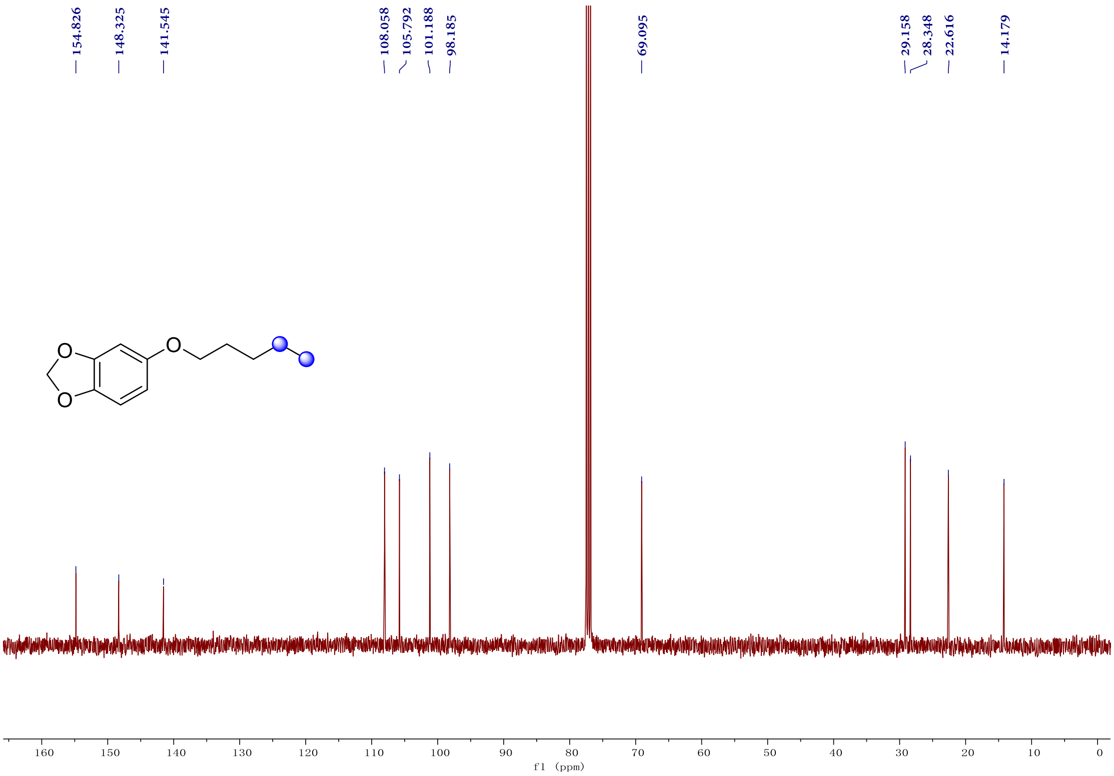

80

^{1}H NMR spectrum of **15** (**CDCl$_3$**)

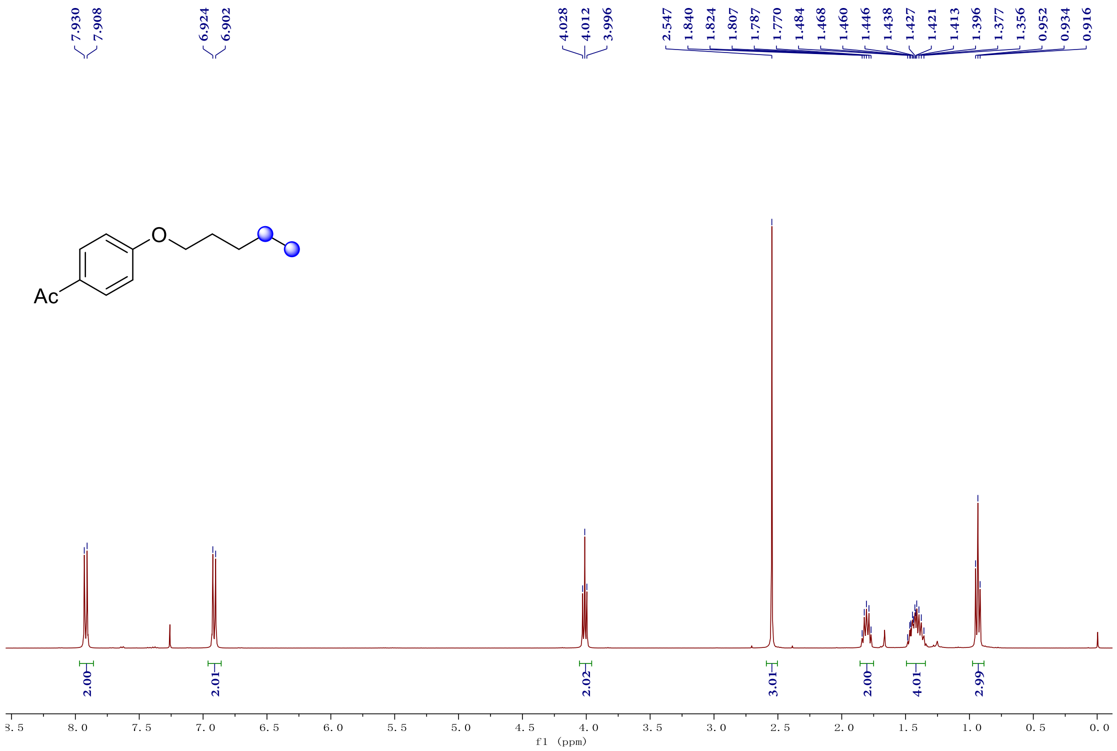

^{13}C NMR spectrum of **15** (**CDCl$_3$**)

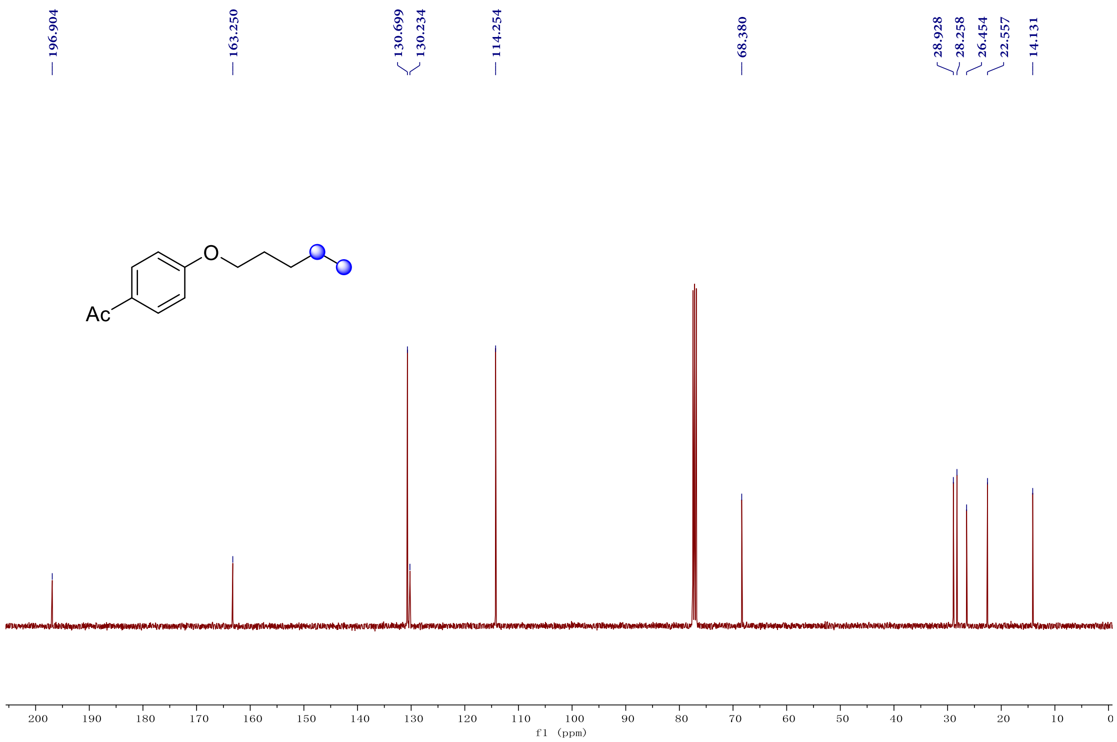

81

^{1}H NMR spectrum of **16** (**$CDCl_3$**)

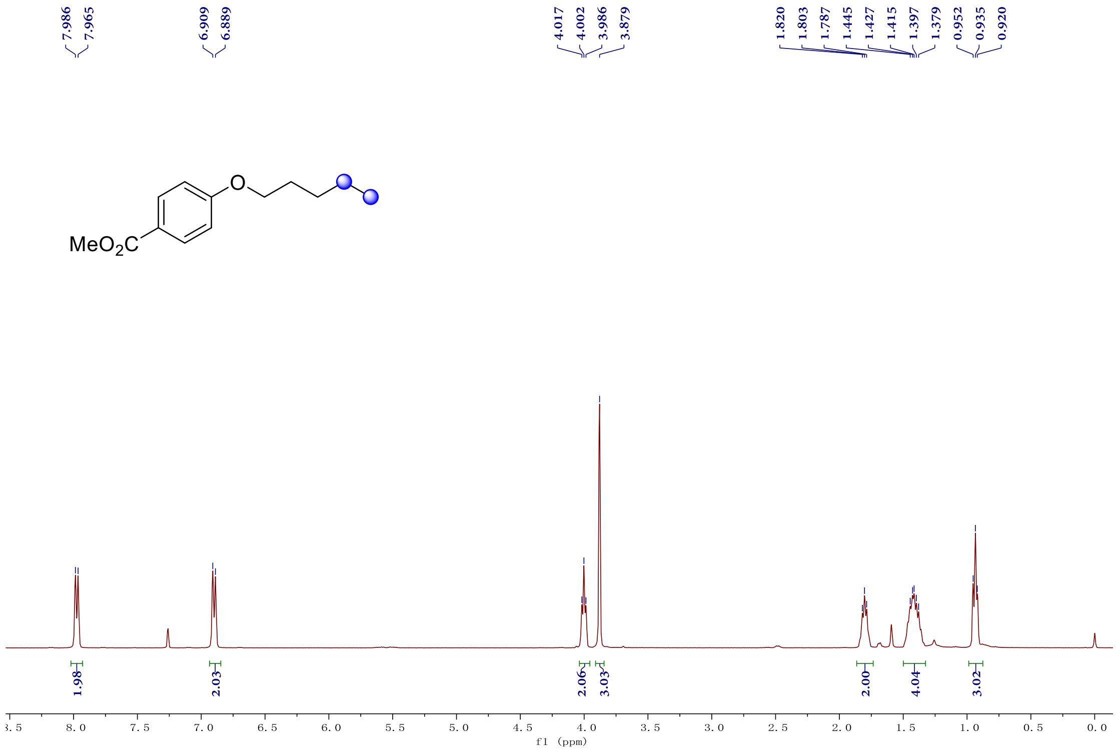

^{13}C NMR spectrum of **16** (**$CDCl_3$**)

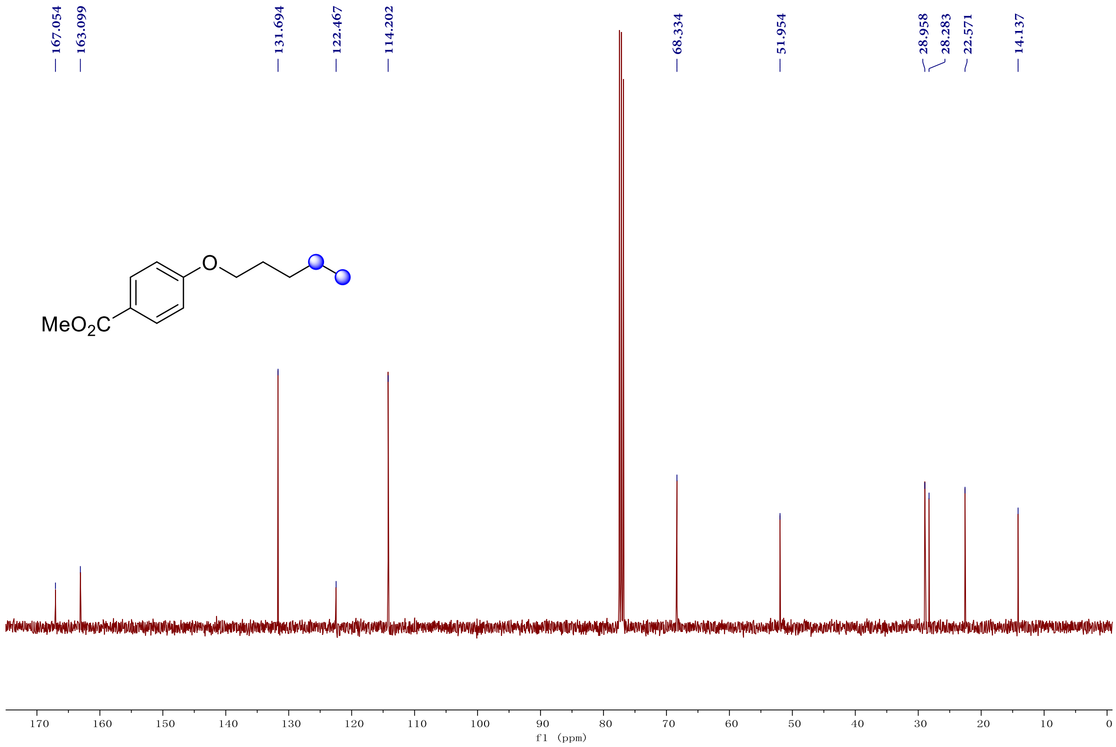

82

^{1}H NMR spectrum of **17** (**$CDCl_3$**)

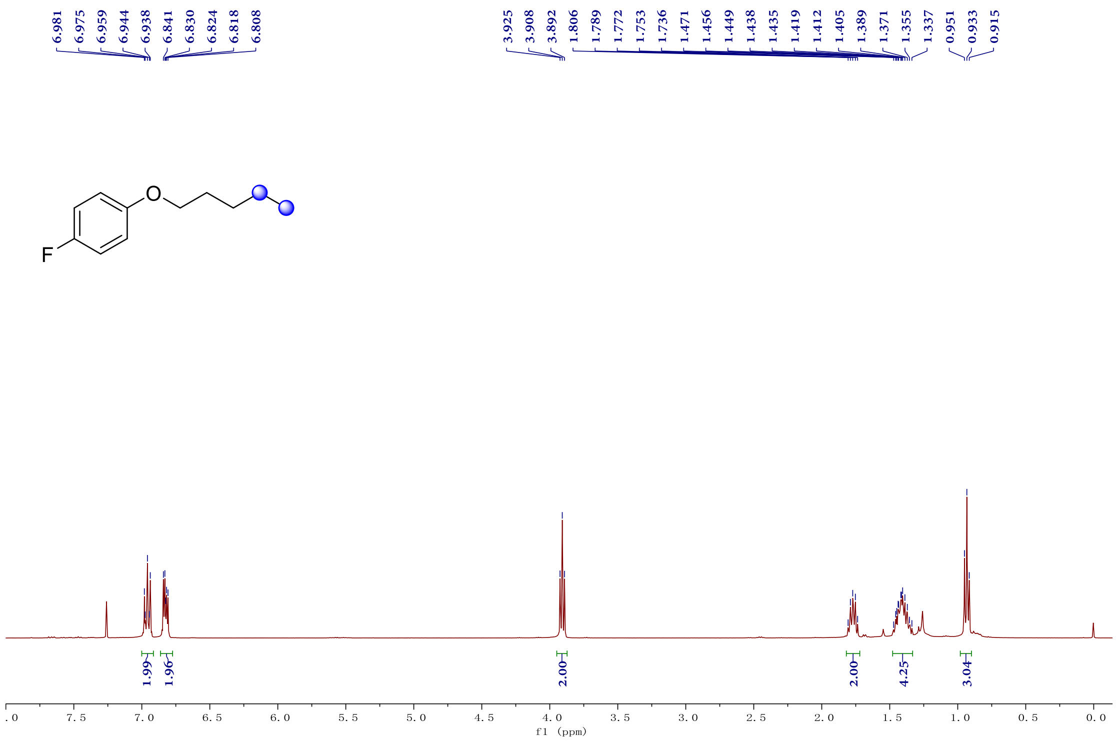

^{13}C NMR spectrum of **17** (**$CDCl_3$**)

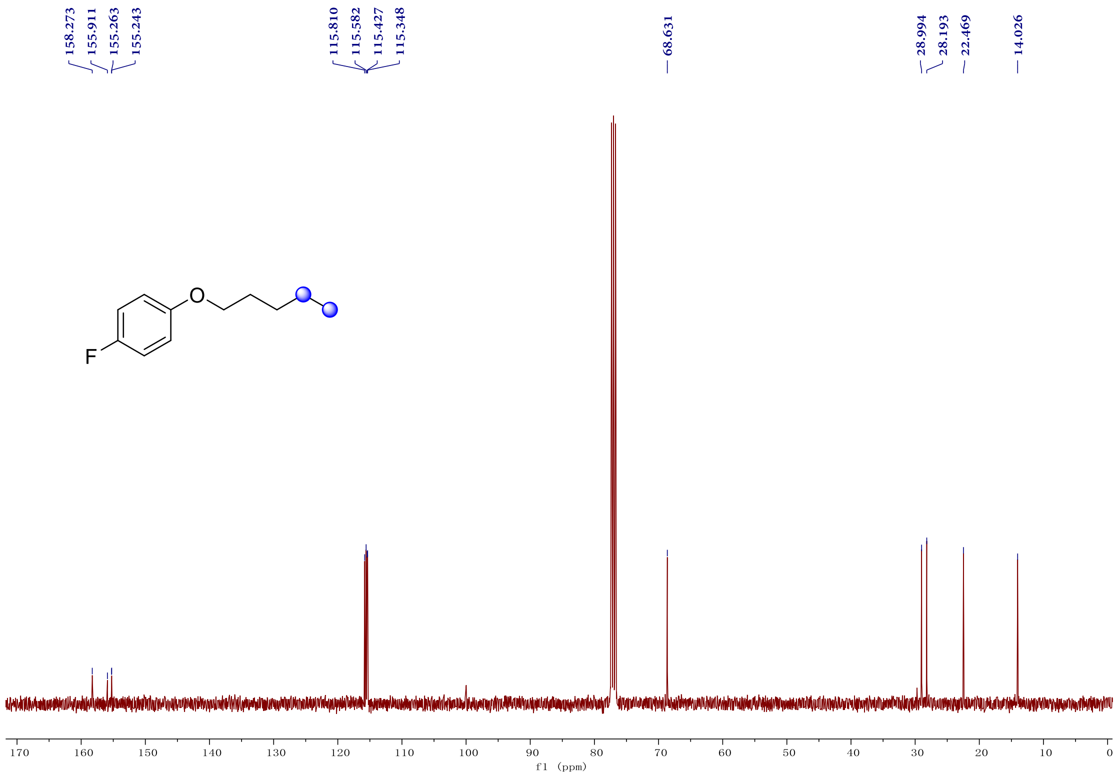

83

^{19}F NMR spectrum of **17** (**CDCl$_3$**)

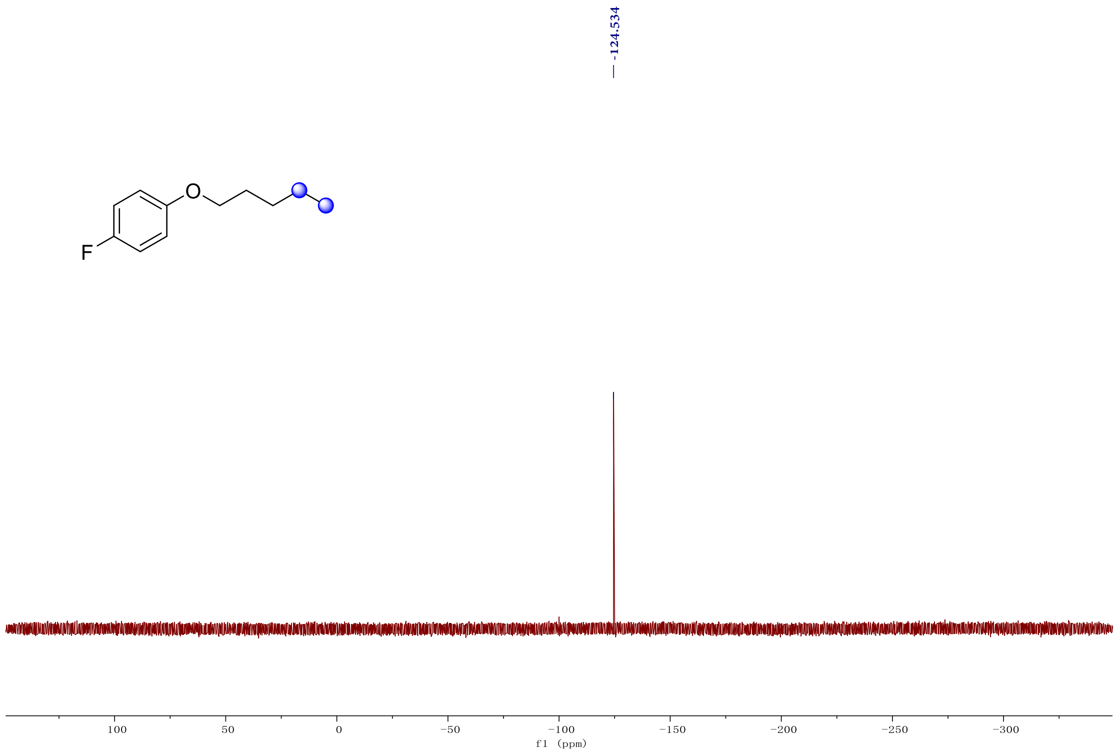

84

^{1}H NMR spectrum of **18** (**CDCl$_3$**)

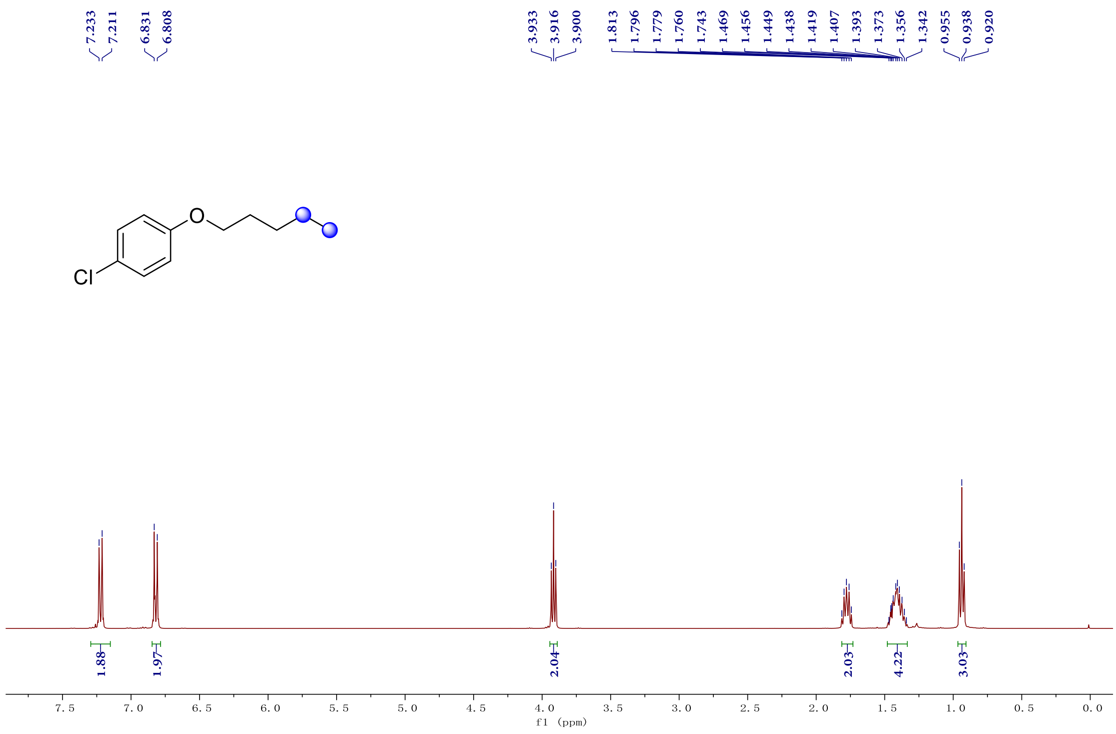

^{13}C NMR spectrum of **18** (**CDCl$_3$**)

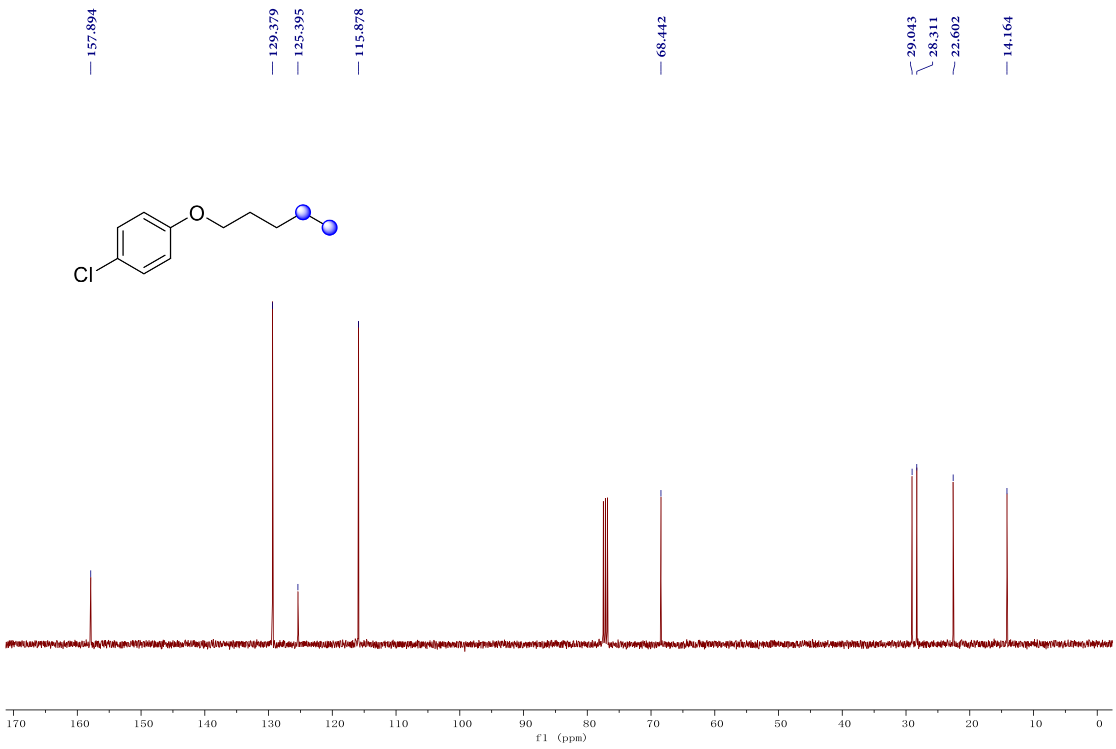

85

^{1}H NMR spectrum of **19** (**$CDCl_3$**)

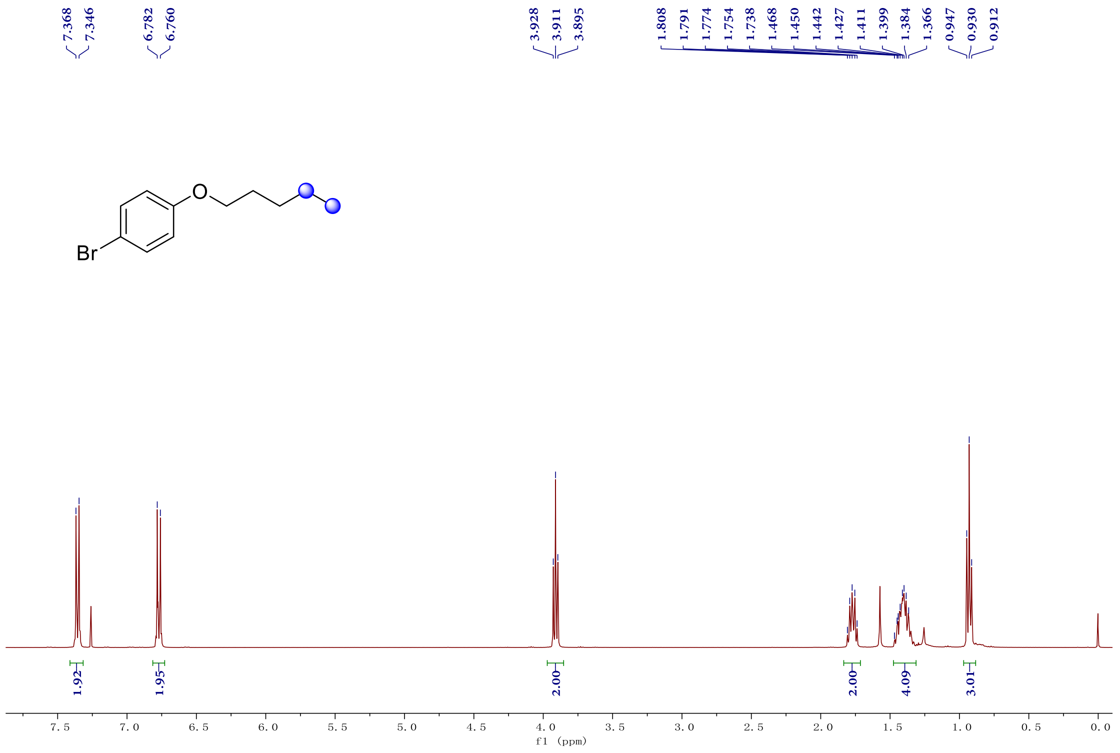

^{13}C NMR spectrum of **19** (**$CDCl_3$**)

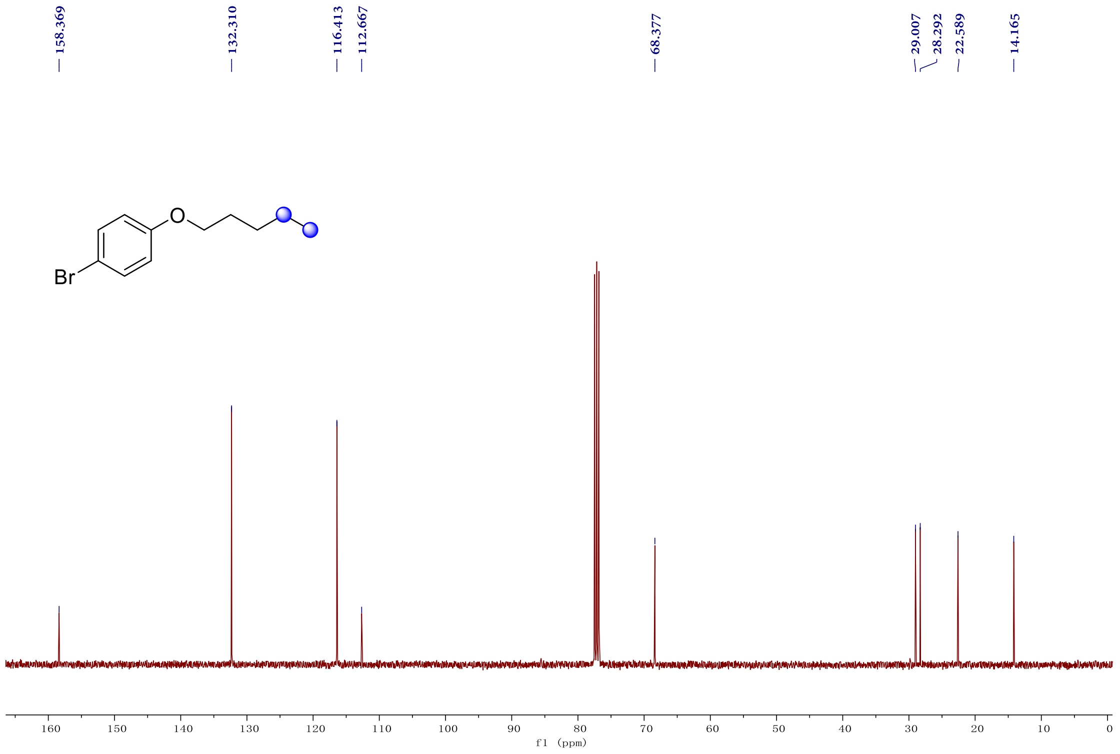

86

^{1}H NMR spectrum of **20** (**CDCl$_3$**)

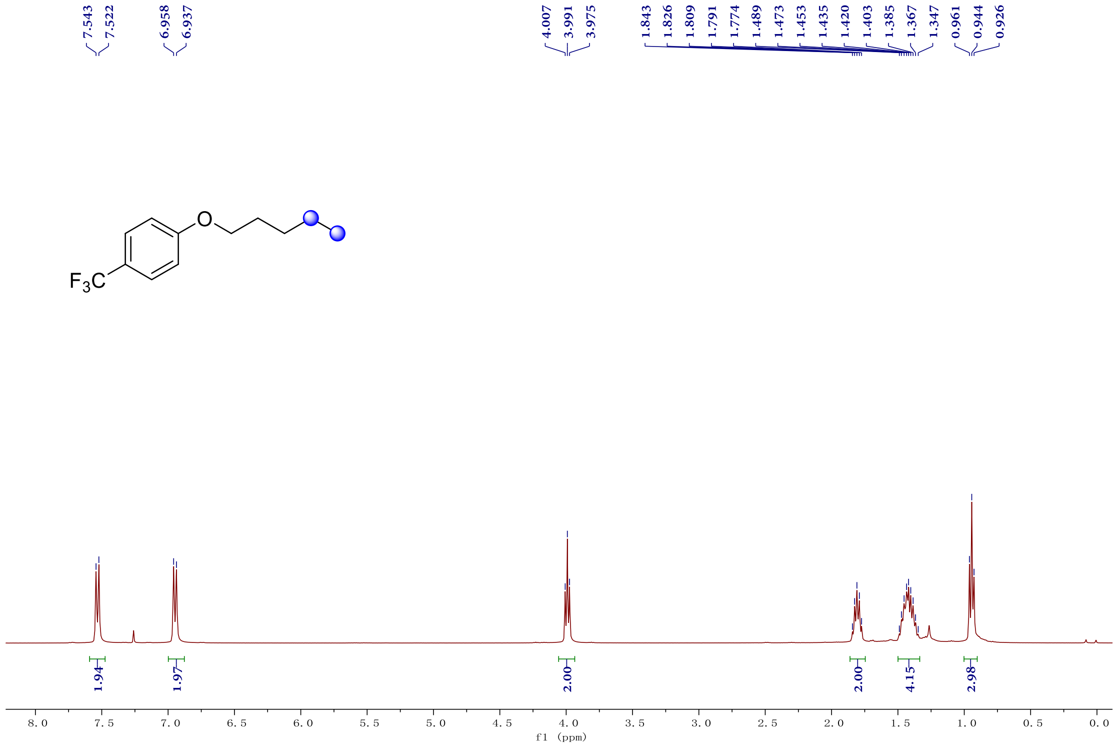

^{13}C NMR spectrum of **20** (**CDCl$_3$**)

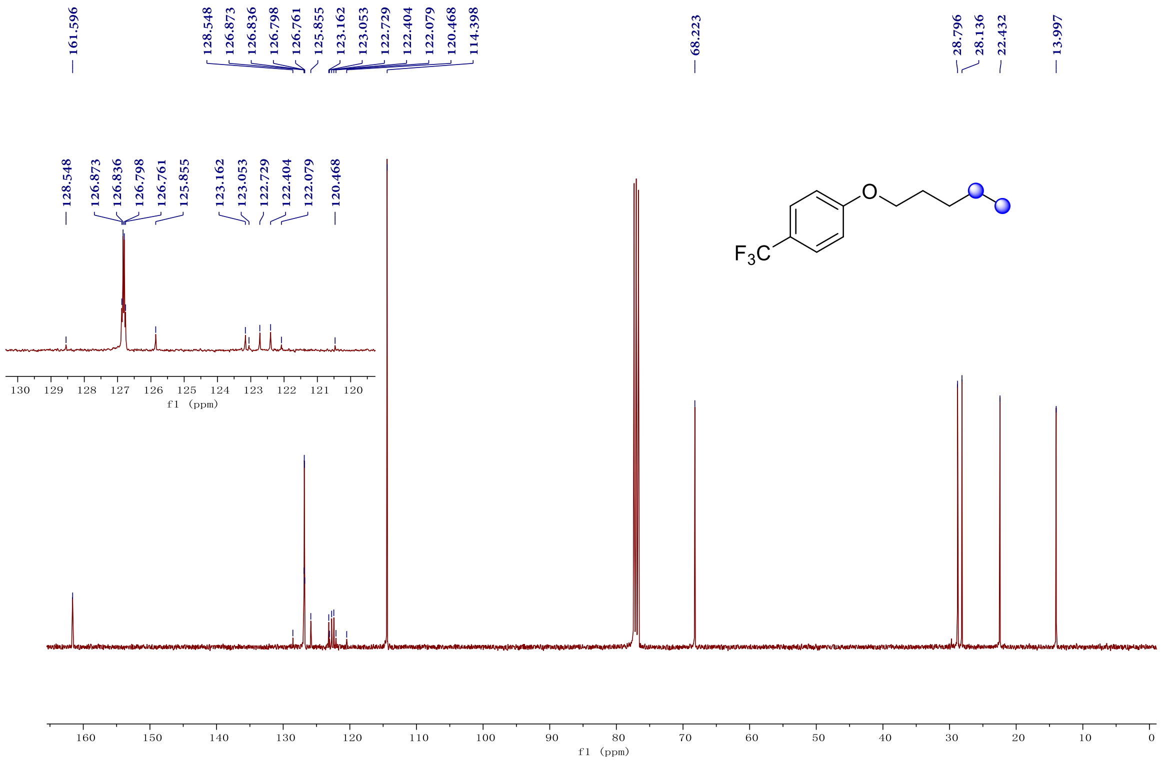

87

^{19}F NMR spectrum of **20** (**$CDCl_3$**)

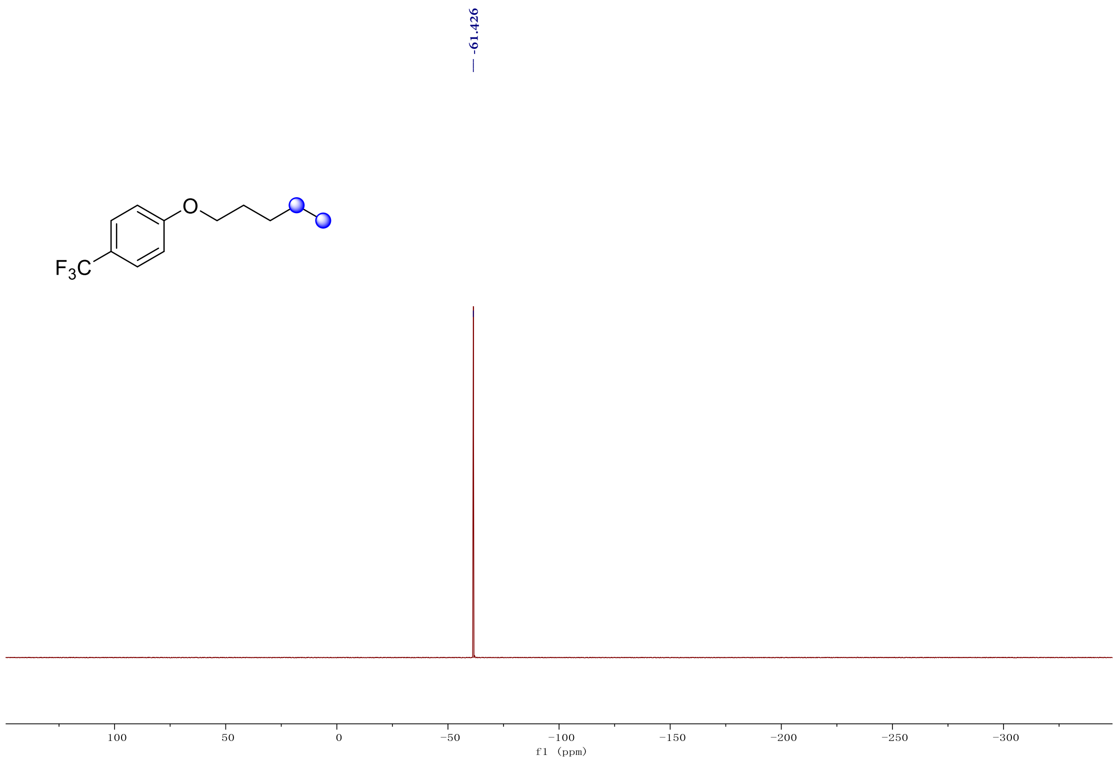

88

^{1}H NMR spectrum of **21** (**$CDCl_3$**)

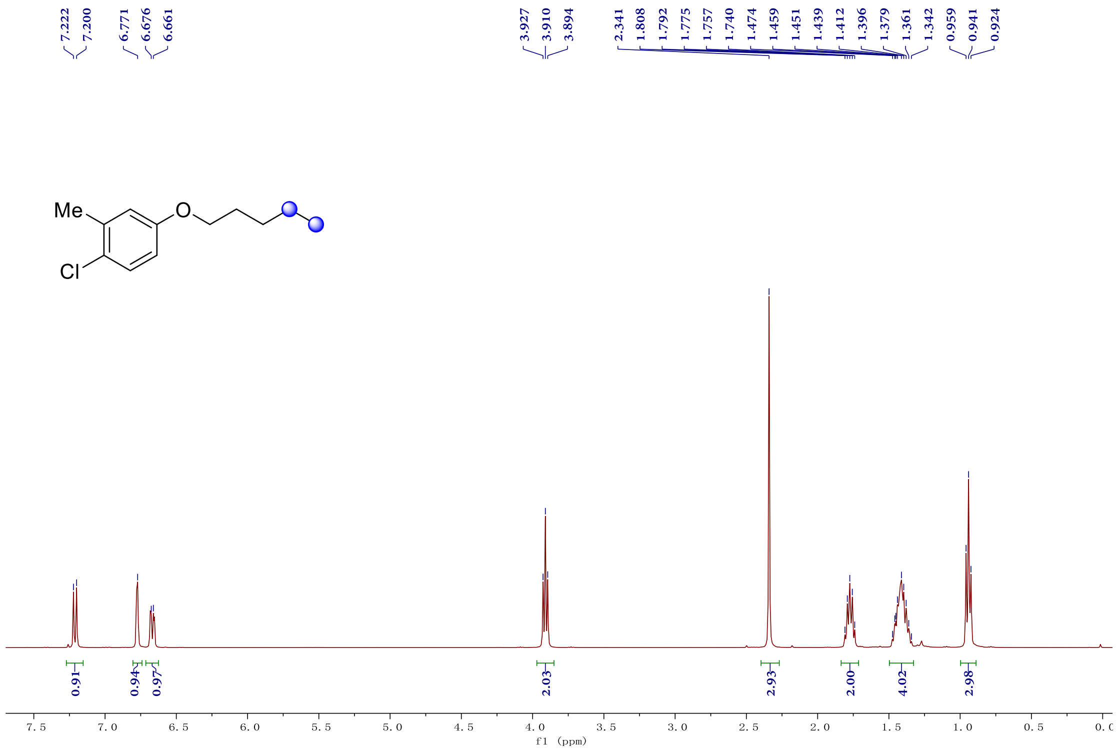

^{13}C NMR spectrum of **21** (**$CDCl_3$**)

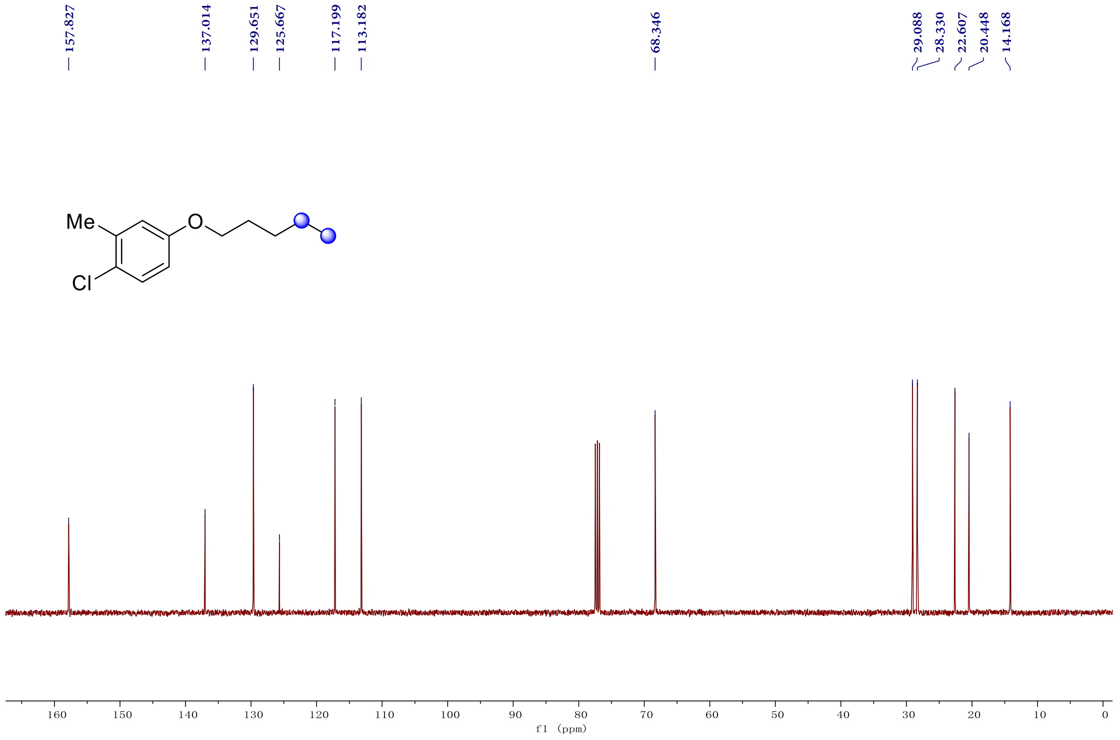

89

^{1}H NMR spectrum of **22** (**$CDCl_3$**)

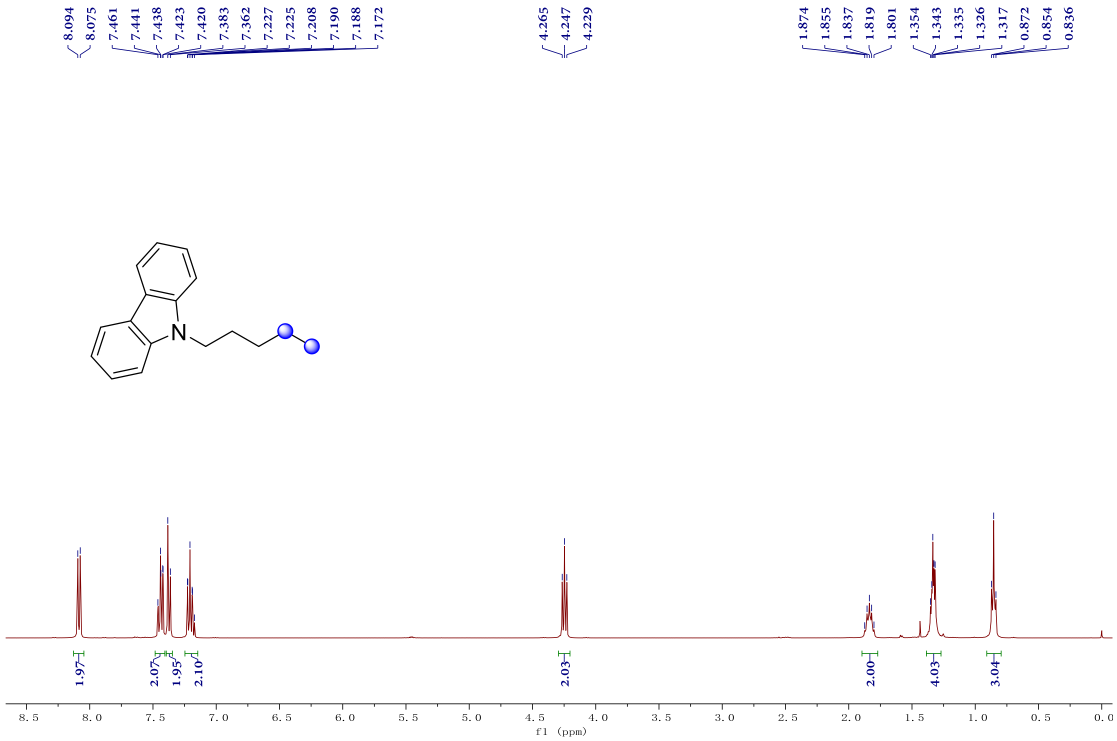

^{13}C NMR spectrum of **22** (**$CDCl_3$**)

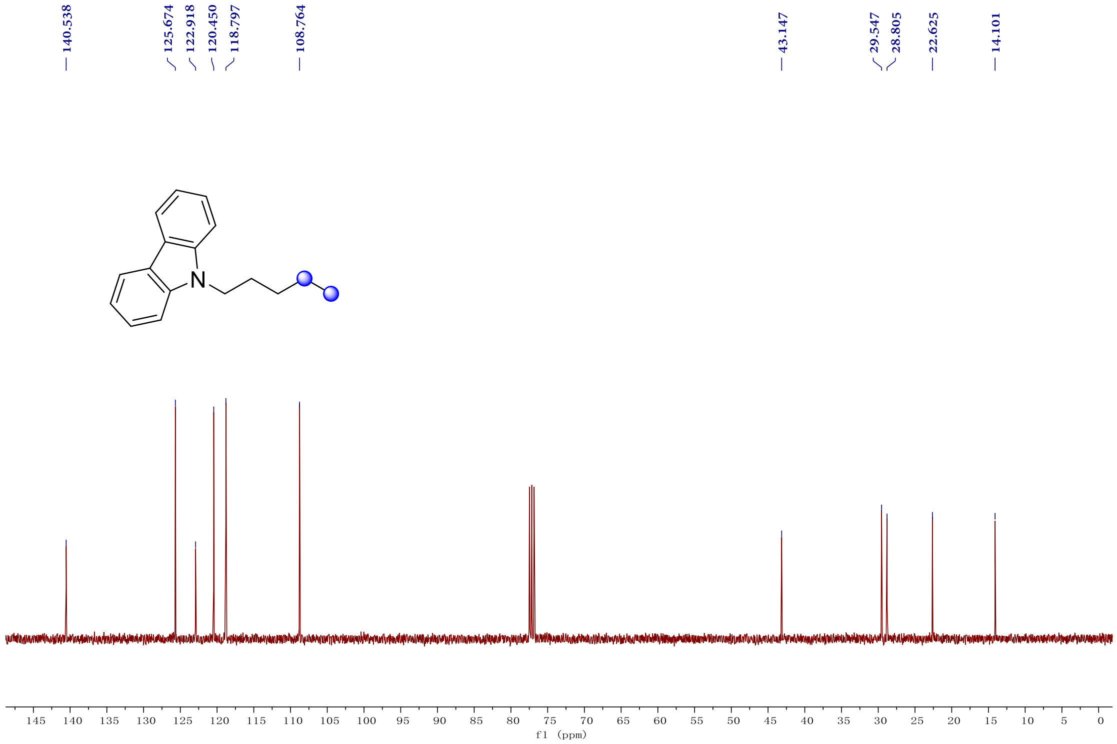

90

^{1}H NMR spectrum of **23** (**$CDCl_3$**)

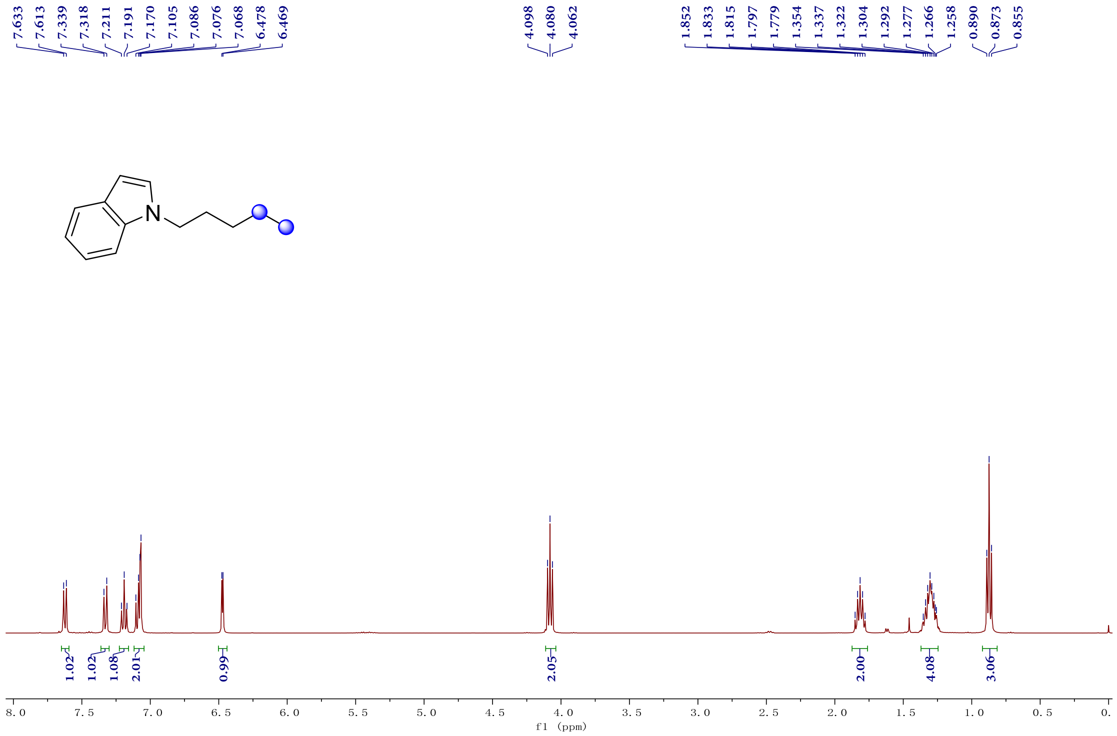

^{13}C NMR spectrum of **23** (**$CDCl_3$**)

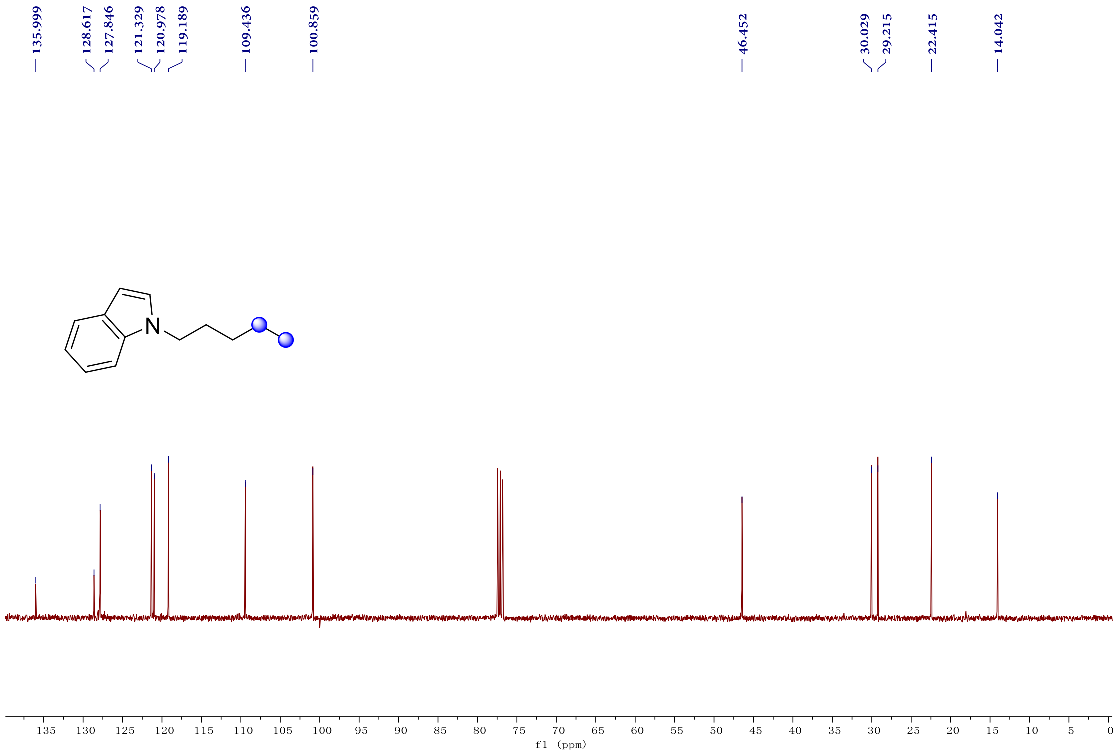

91

^{1}H NMR spectrum of **24** (**$CDCl_3$**)

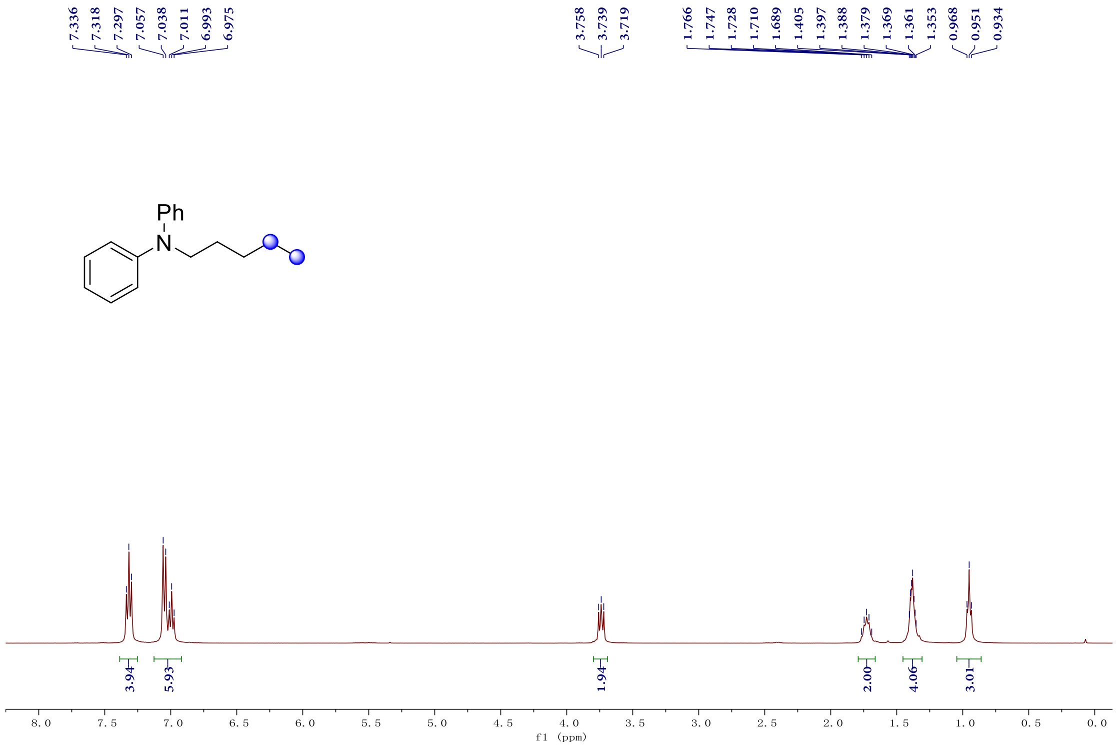

^{13}C NMR spectrum of **24** (**$CDCl_3$**)

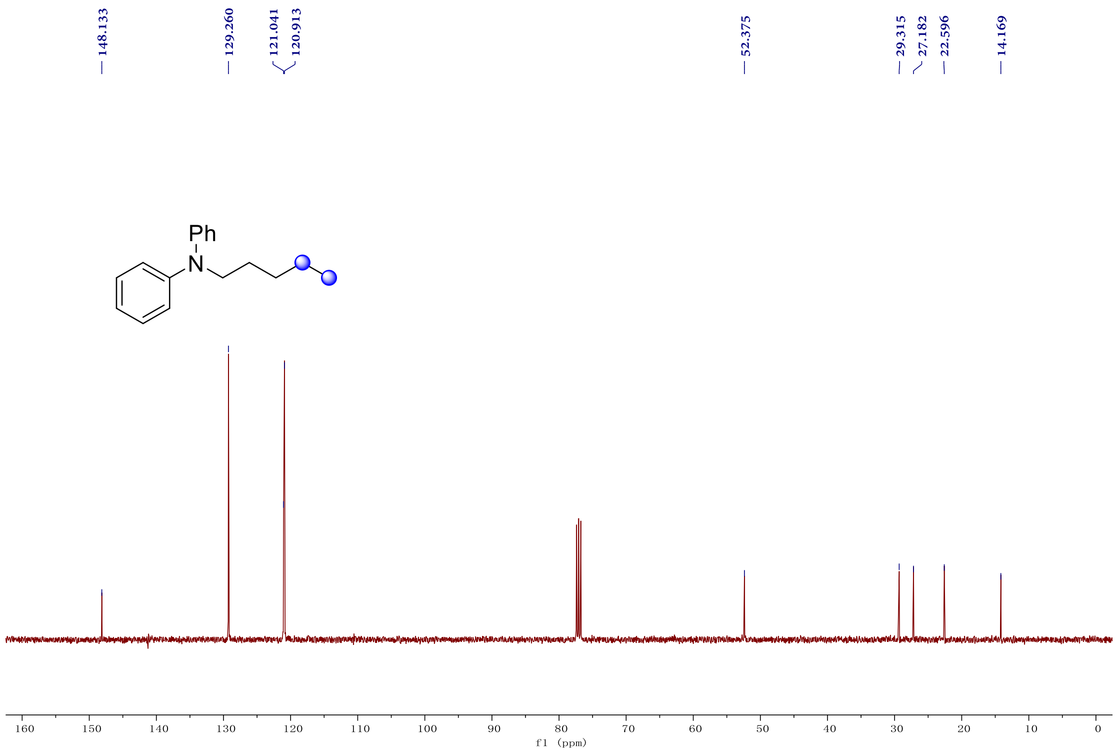

92

^{1}H NMR spectrum of **25** (**CDCl$_3$**)

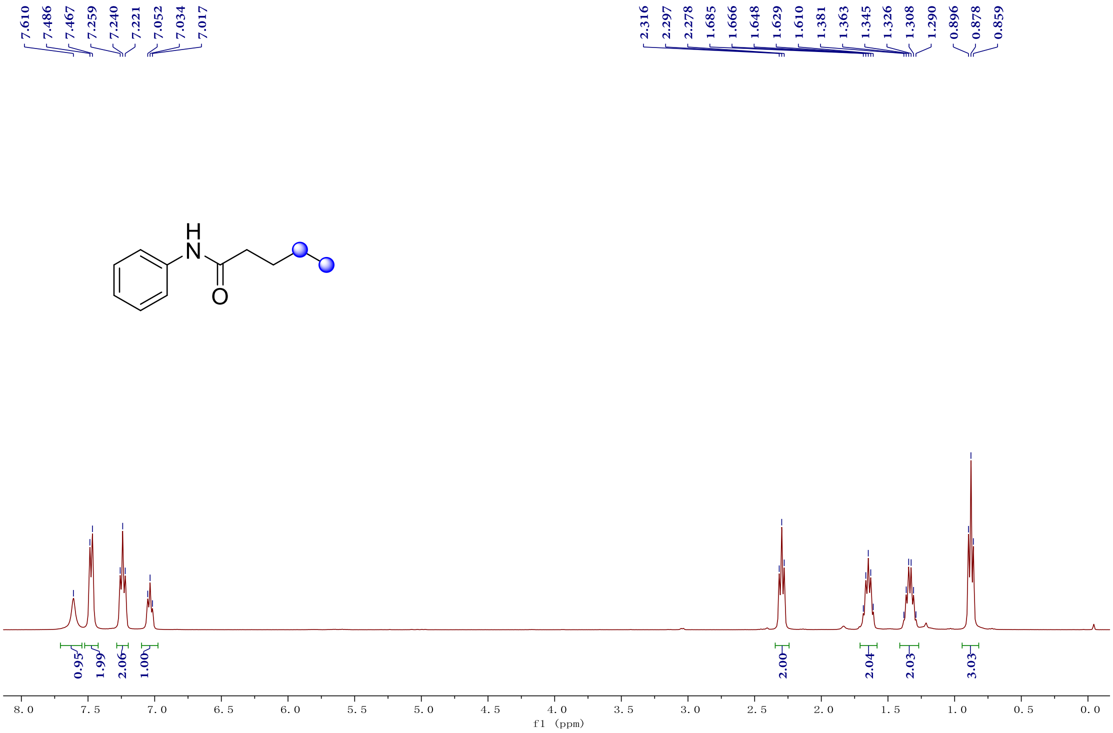

^{13}C NMR spectrum of **25** (**CDCl$_3$**)

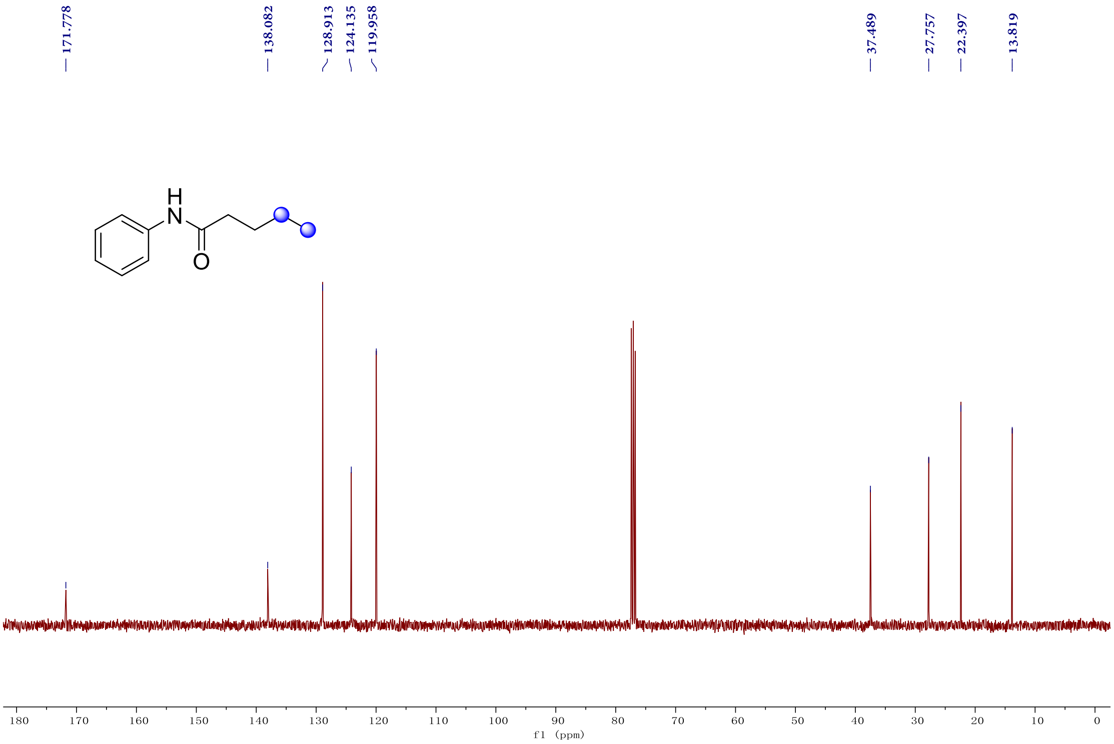

^{1}H NMR spectrum of **26** (**$CDCl_3$**)

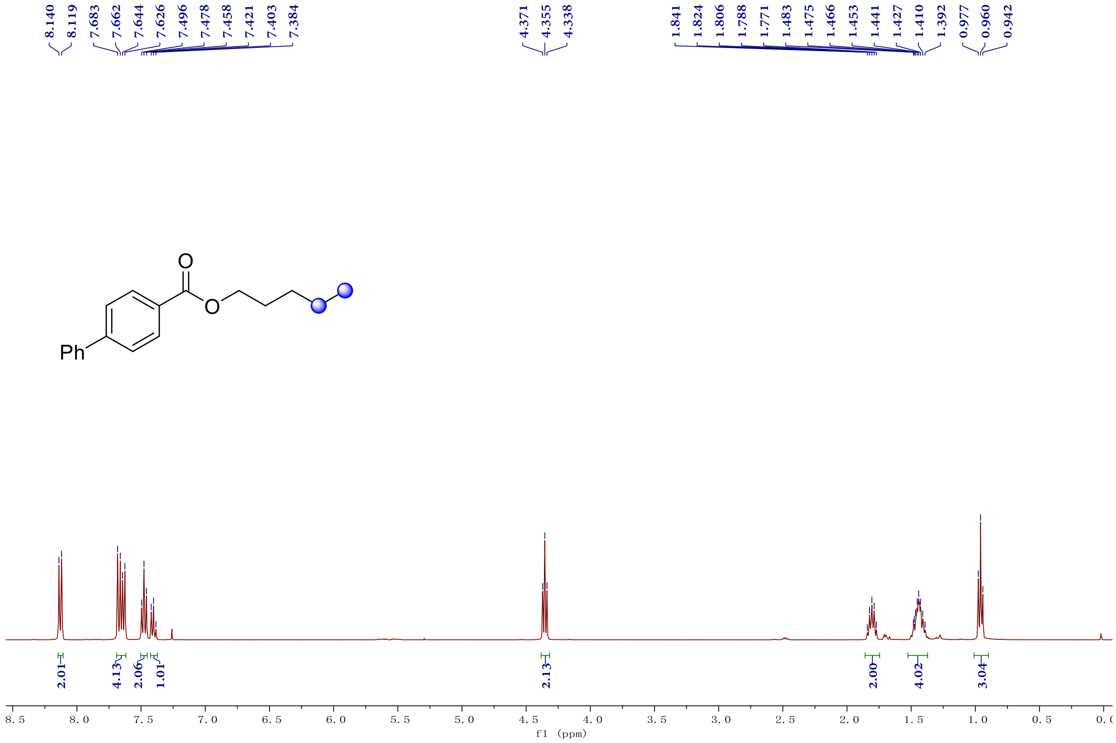

^{13}C NMR spectrum of **26** (**$CDCl_3$**)

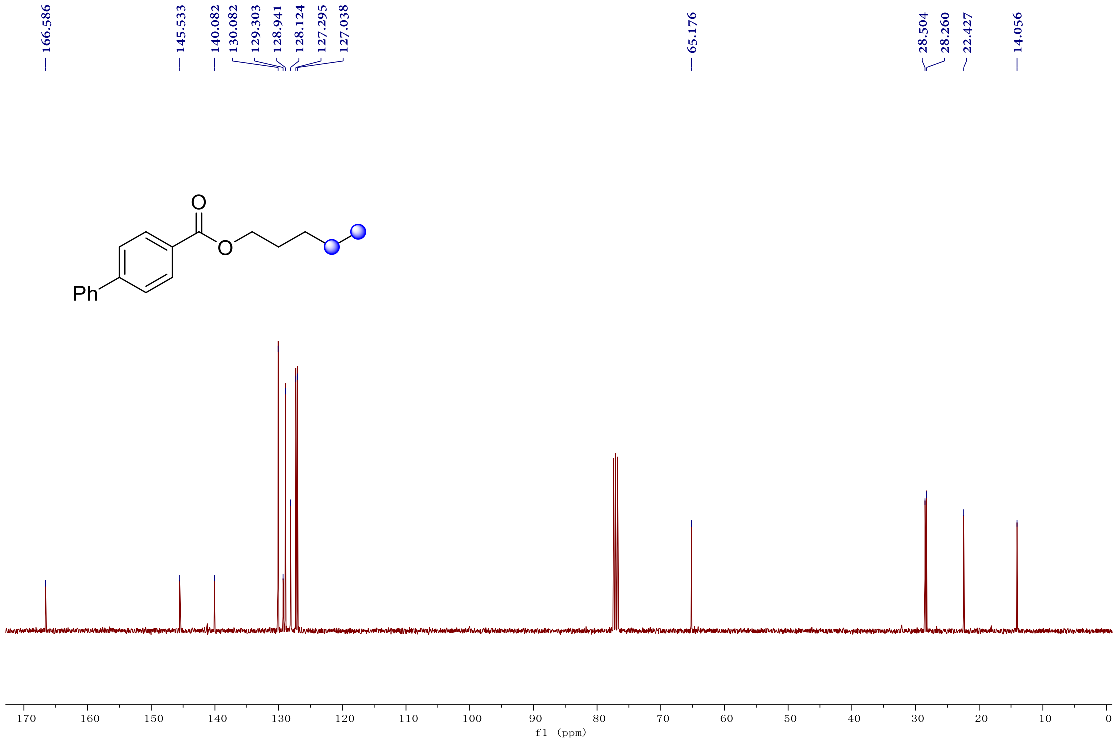

94

^{1}H NMR spectrum of **27** (**$CDCl_3$**)

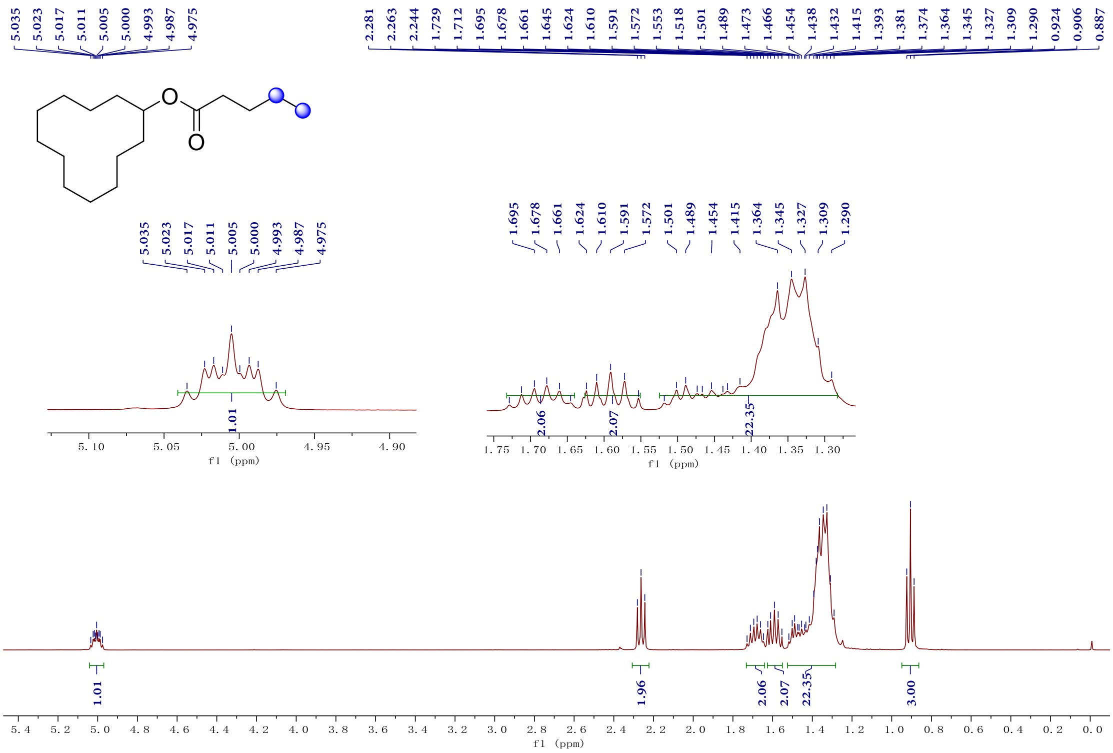

^{13}C NMR spectrum of **27** (**$CDCl_3$**)

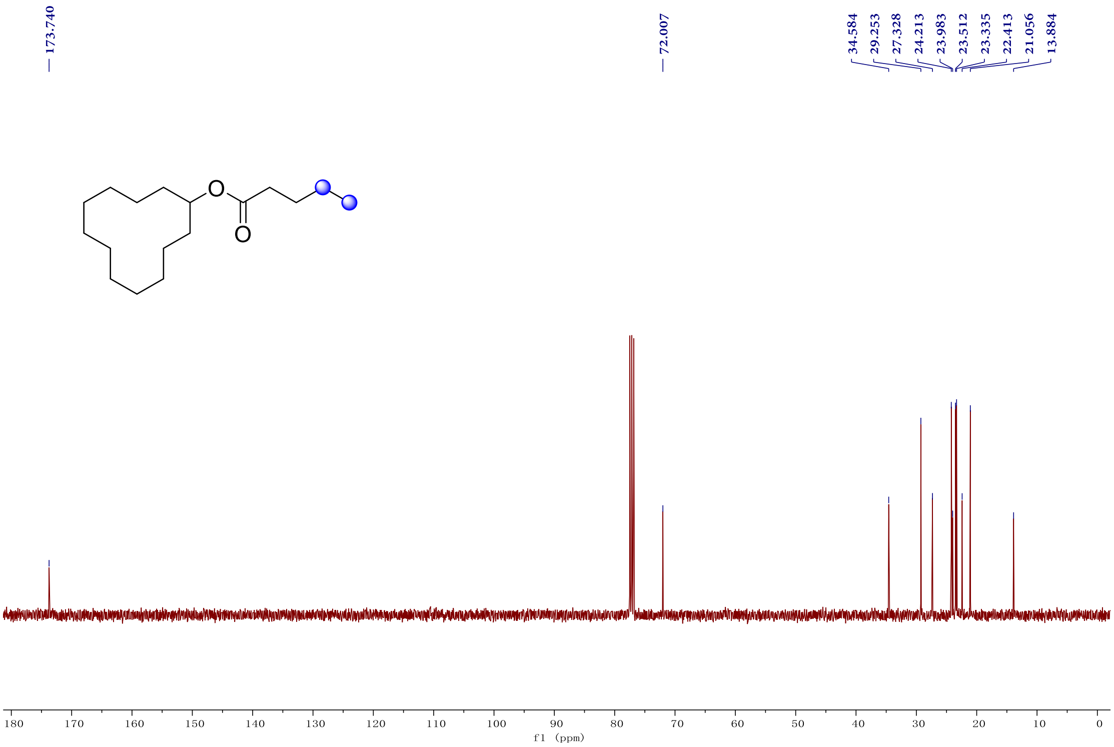

95

^{1}H NMR spectrum of **28** (**$CDCl_3$**)

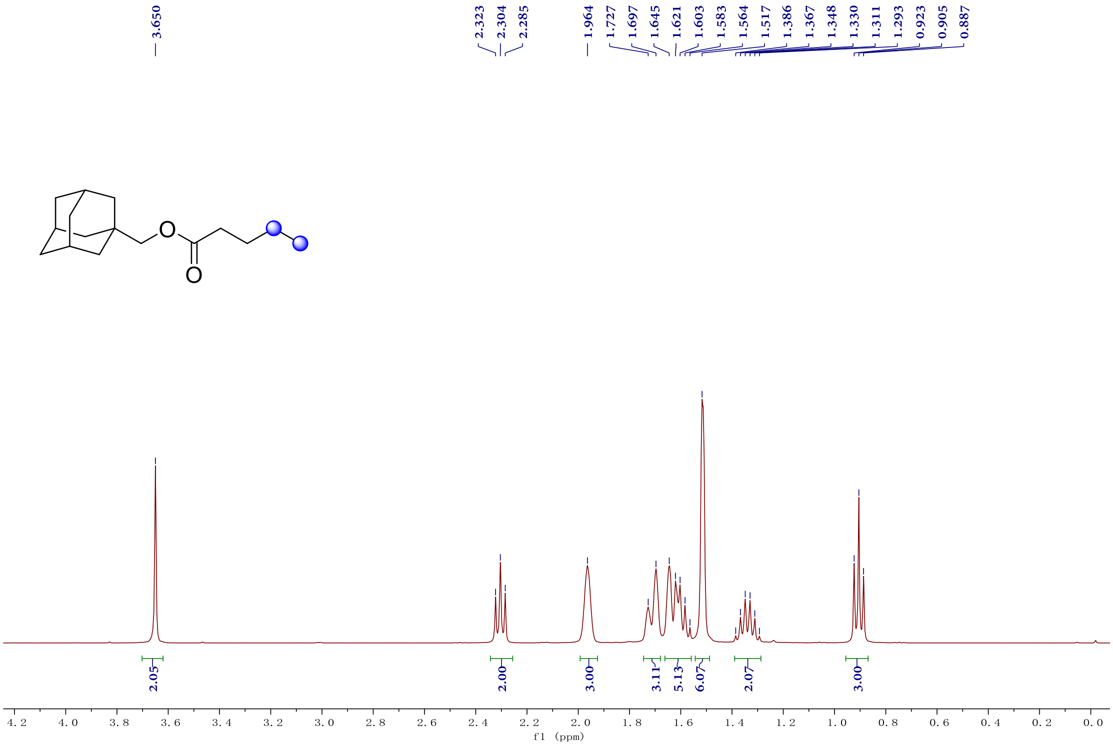

^{13}C NMR spectrum of **28** (**$CDCl_3$**)

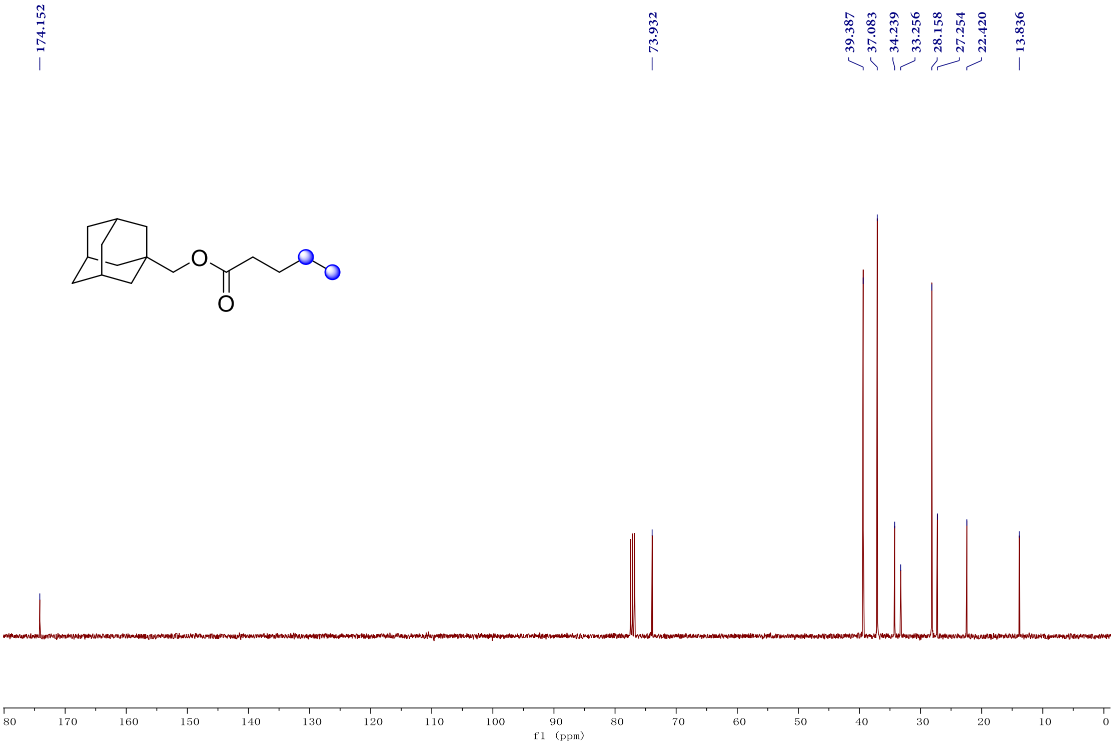

96

^{1}H NMR spectrum of **29** (**$CDCl_3$**)

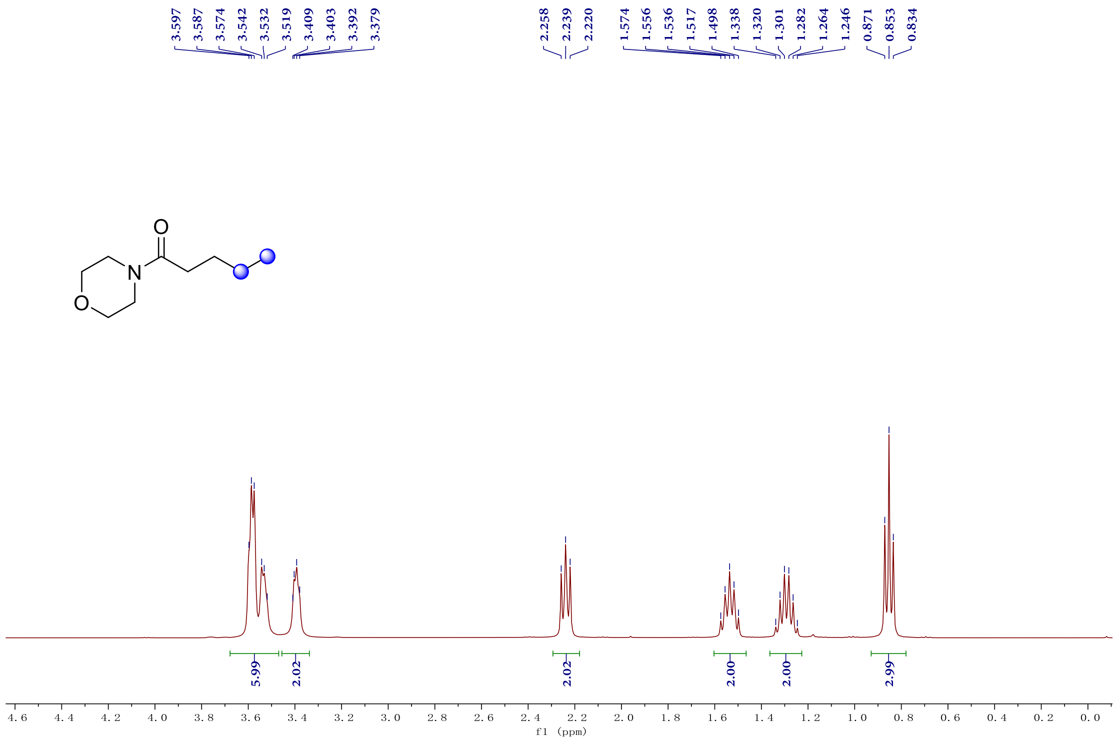

^{13}C NMR spectrum of **29** (**$CDCl_3$**)

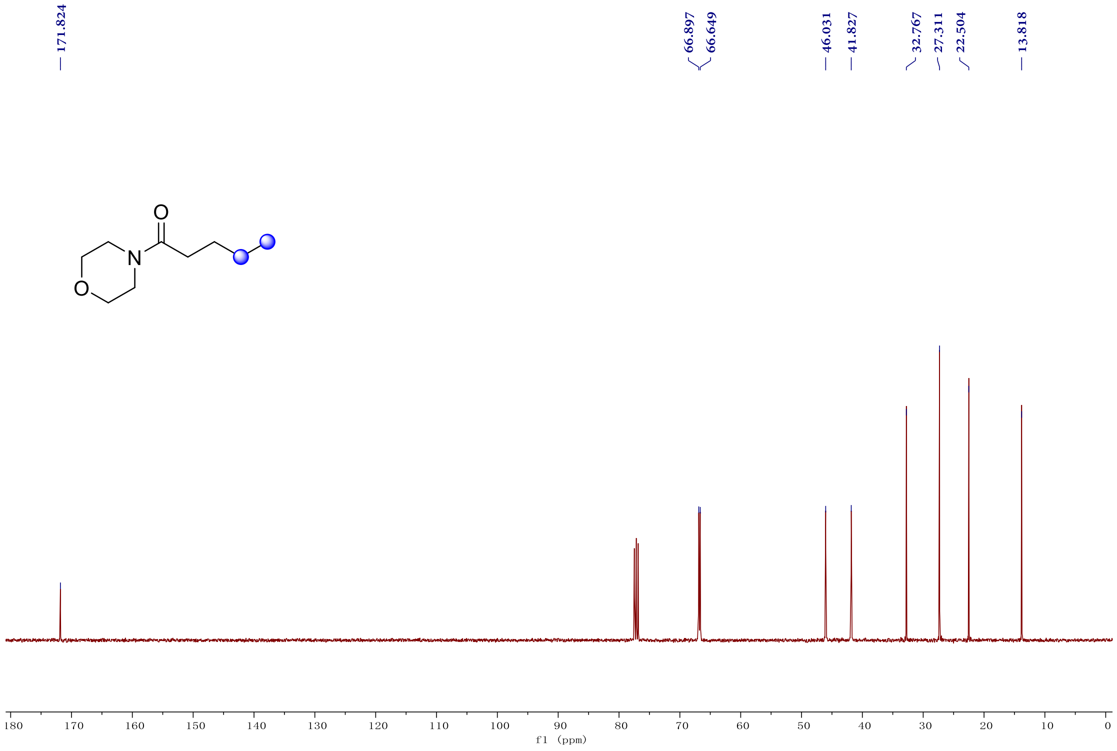

97

^{1}H NMR spectrum of **30** (**$CDCl_3$**)

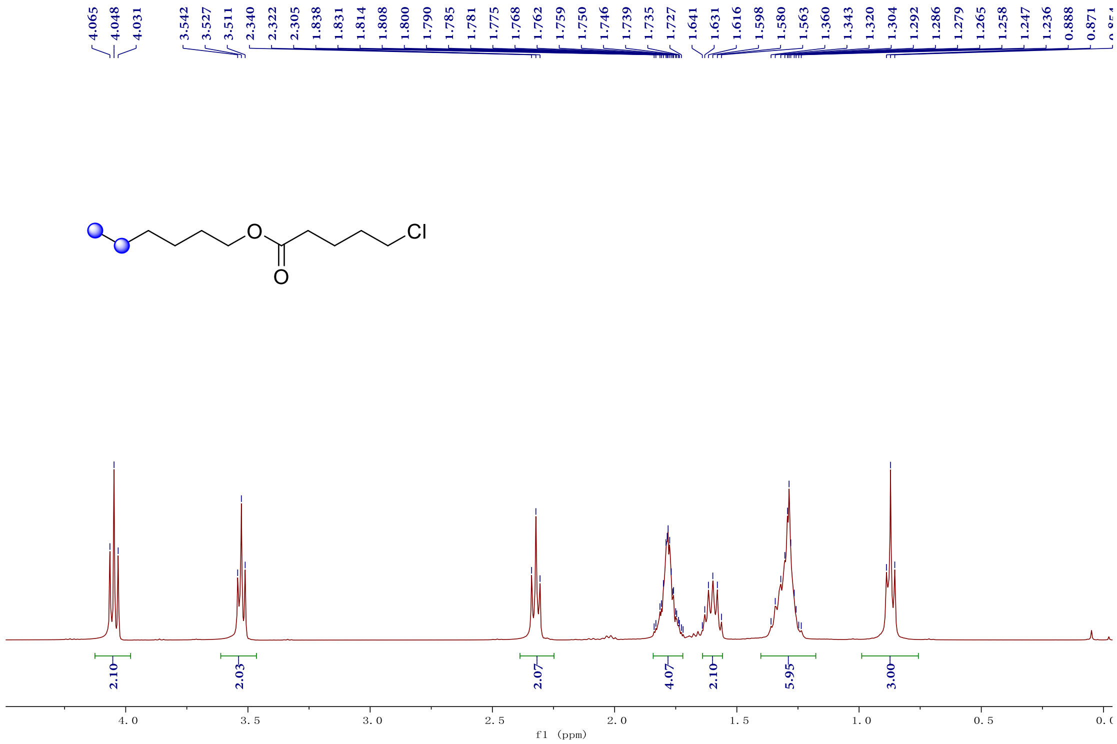

^{13}C NMR spectrum of **30** (**$CDCl_3$**)

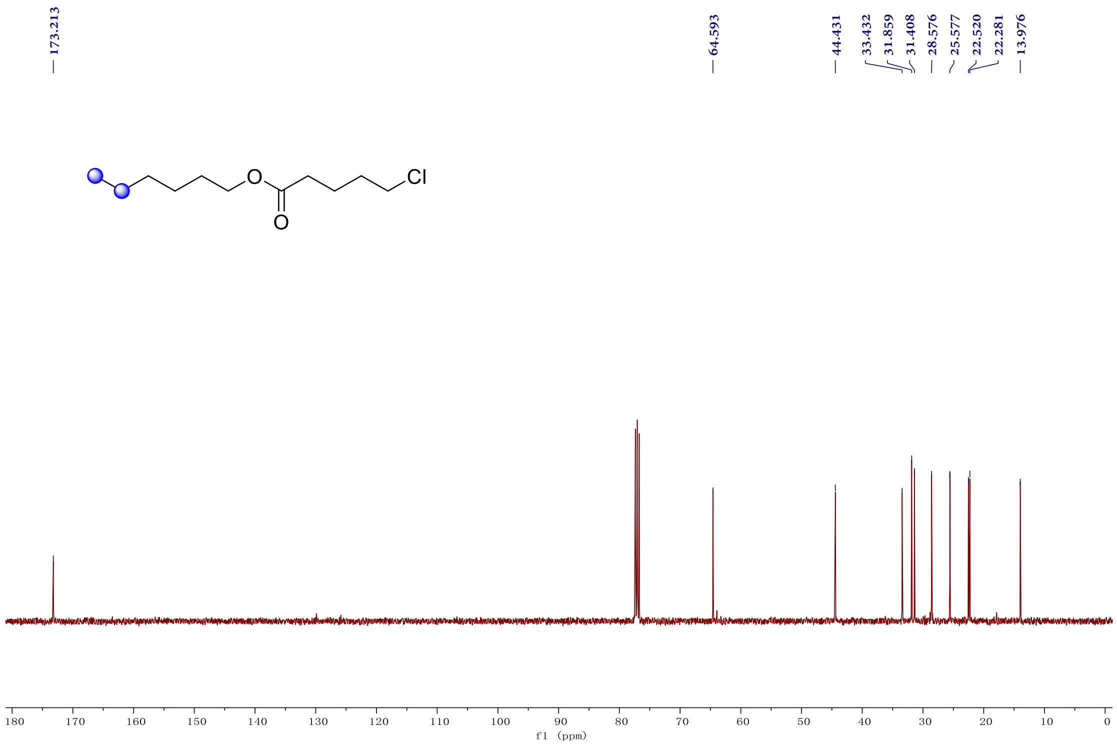

98

^{1}H NMR spectrum of **31** (**$CDCl_3$**)

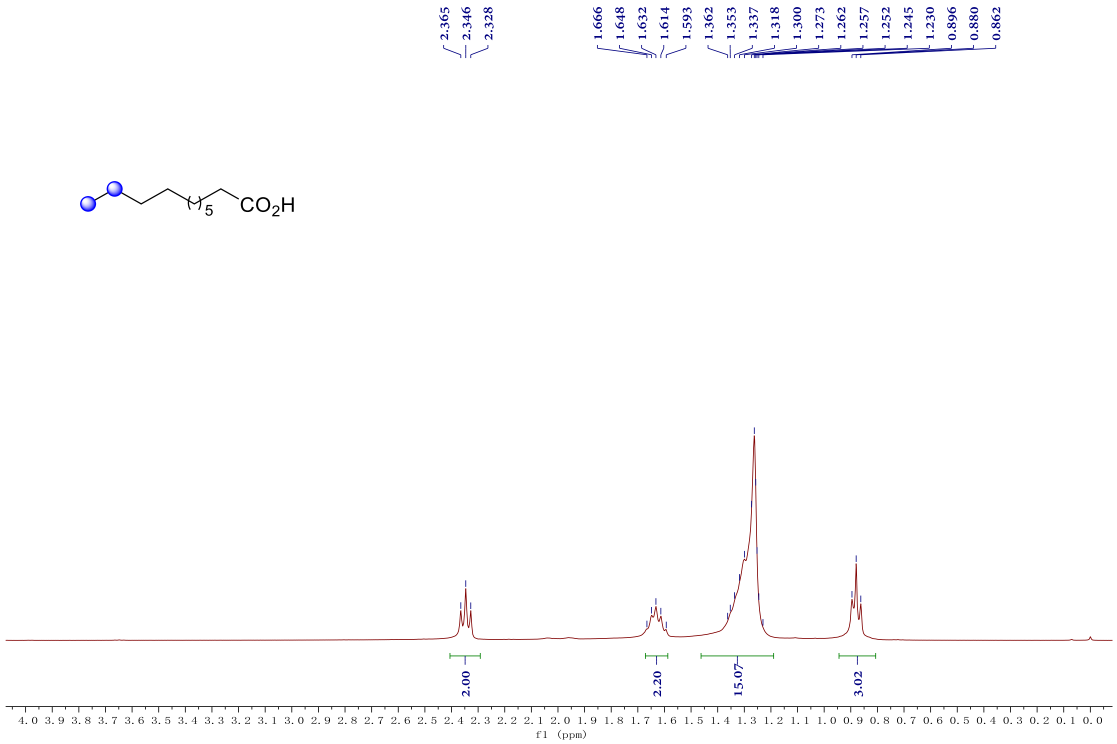

^{13}C NMR spectrum of **31** (**$CDCl_3$**)

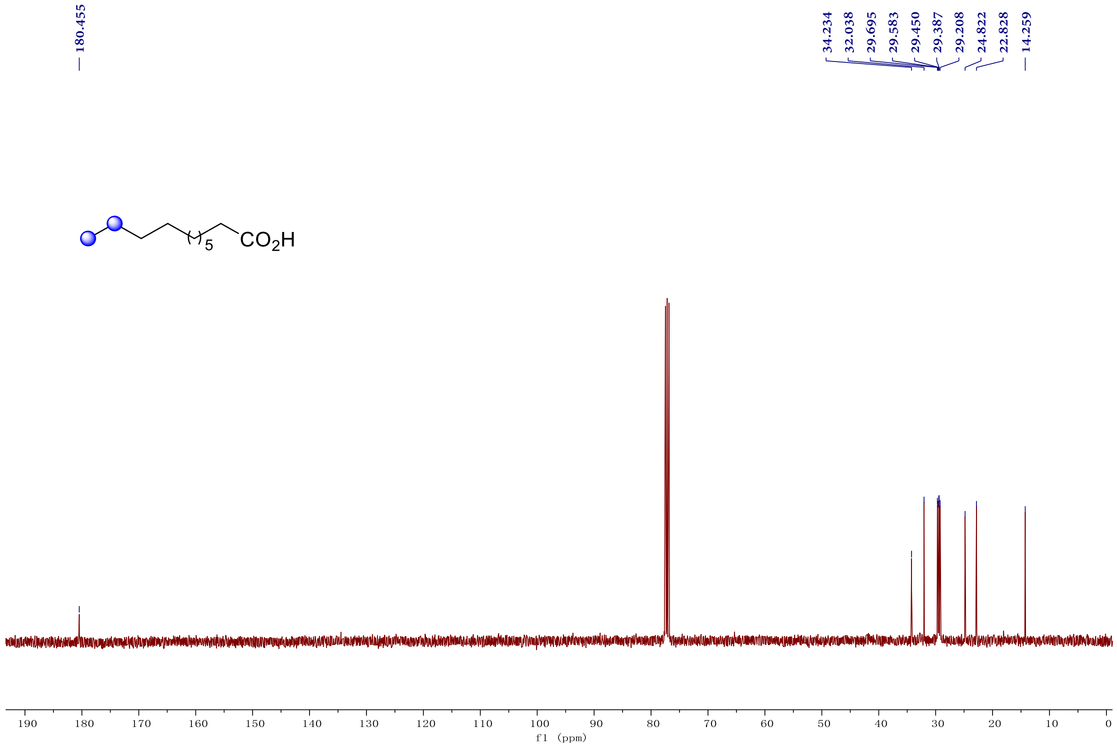

99

^{1}H NMR spectrum of **32** (**$CDCl_3$**)

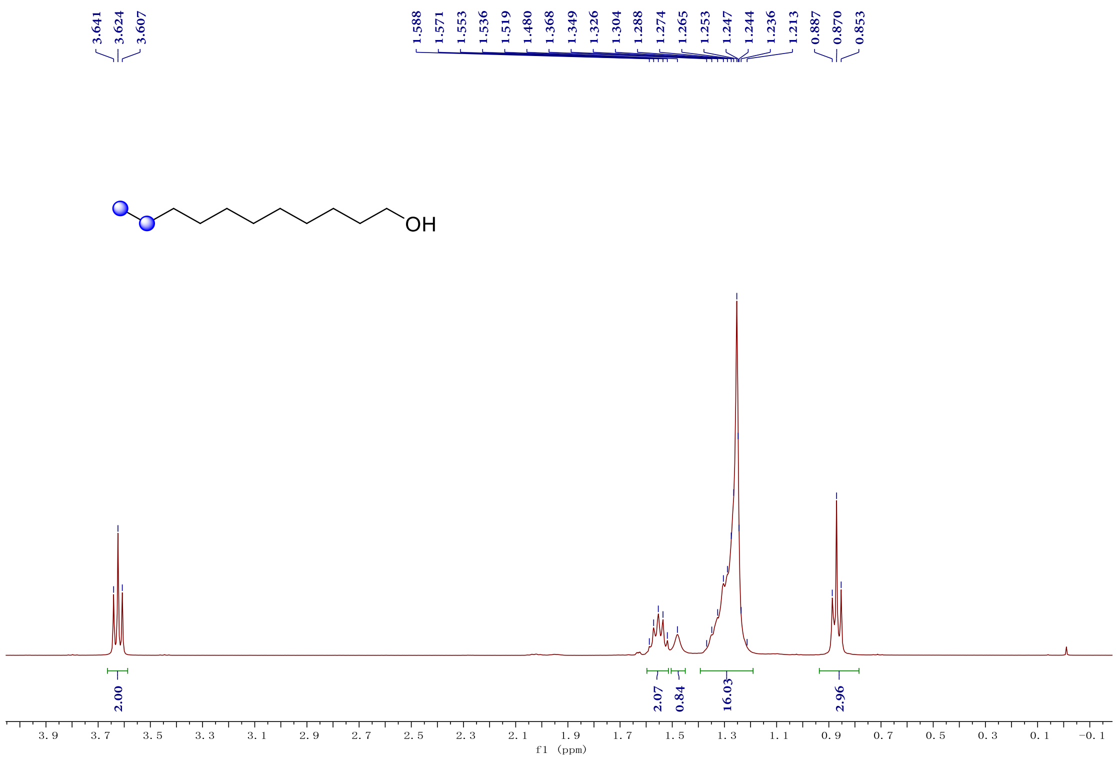

^{13}C NMR spectrum of **32** (**$CDCl_3$**)

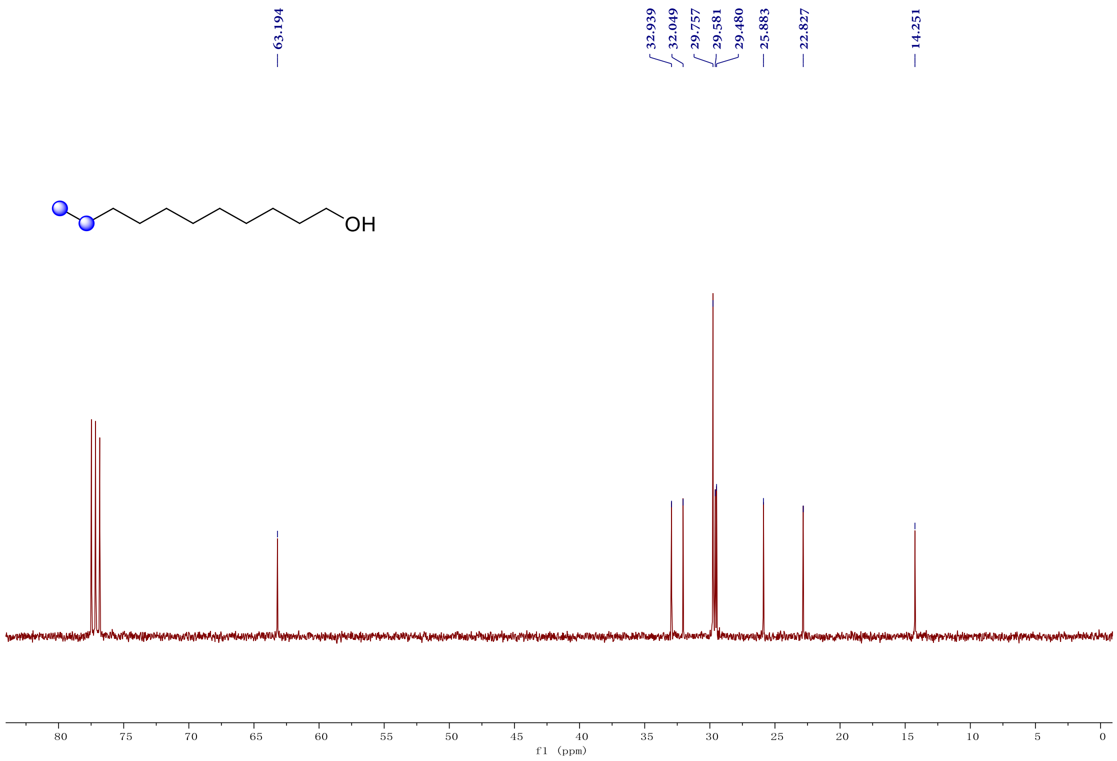

100

^{1}H NMR spectrum of **33** (**CDCl$_3$**)

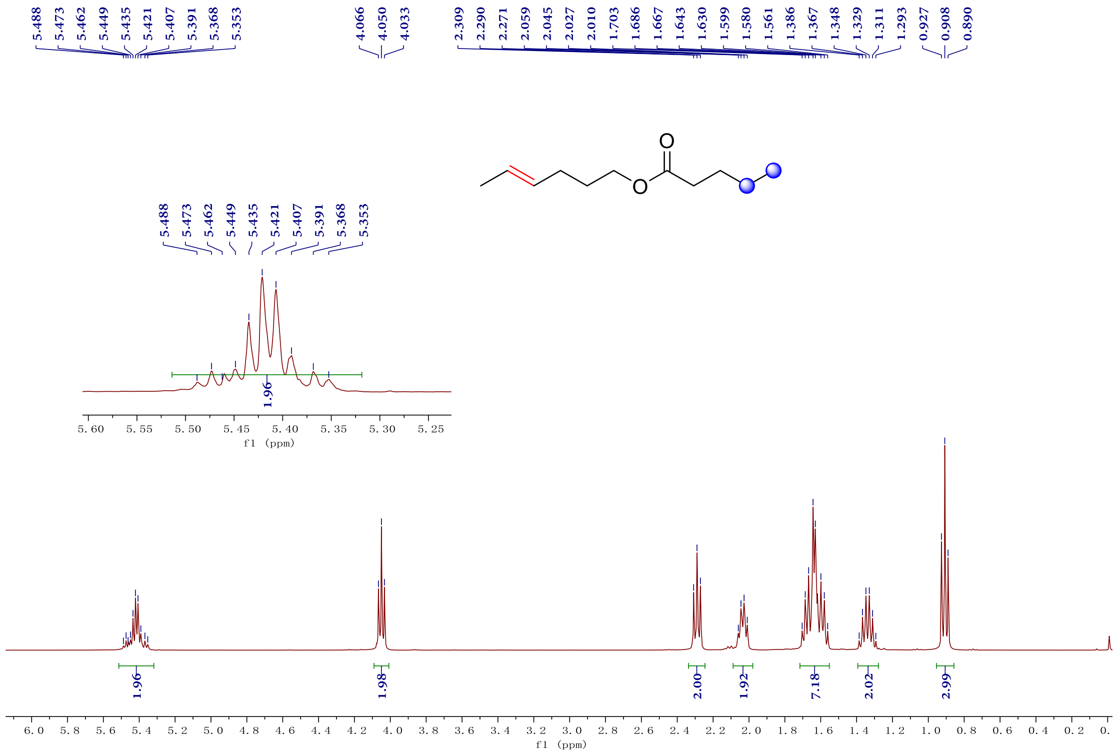

^{13}C NMR spectrum of **33** (**CDCl$_3$**)

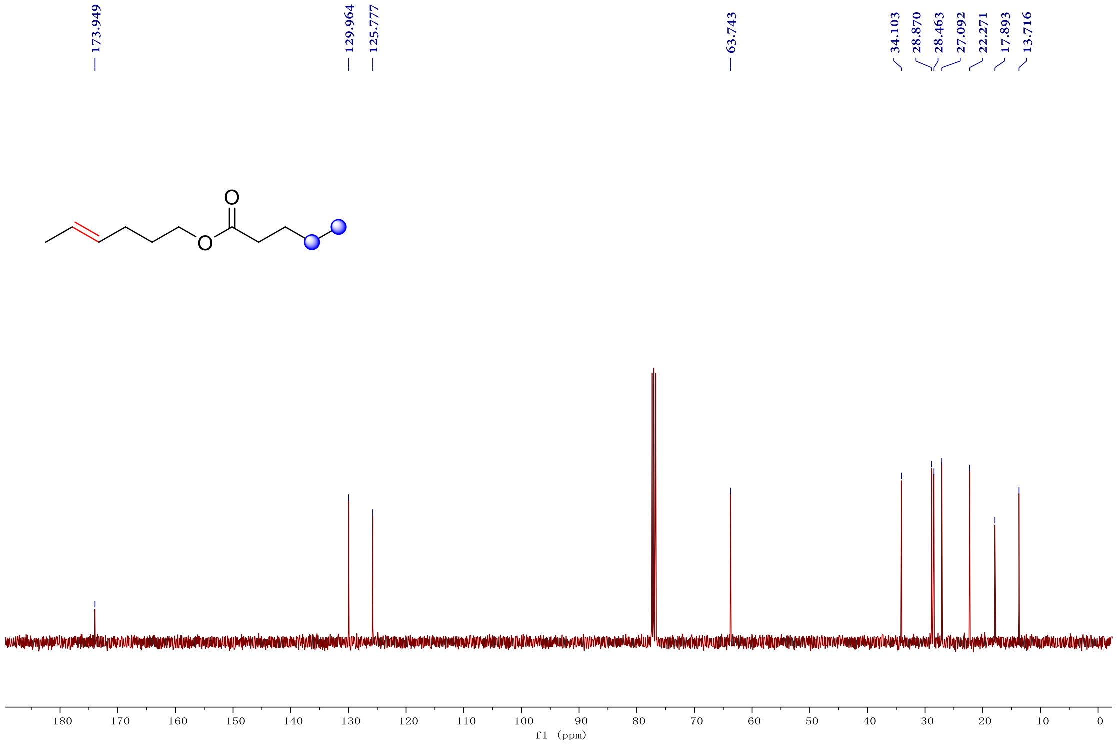

101

^{1}H NMR spectrum of **34** ($CDCl_3$)

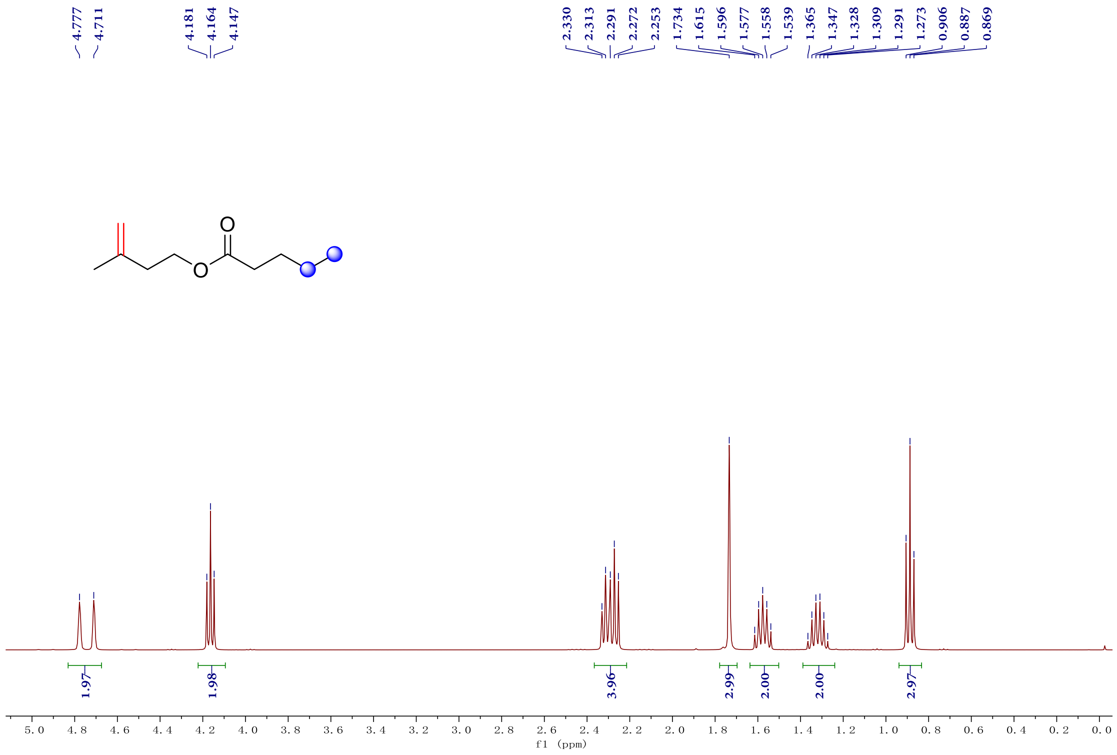

^{13}C NMR spectrum of **34** ($CDCl_3$)

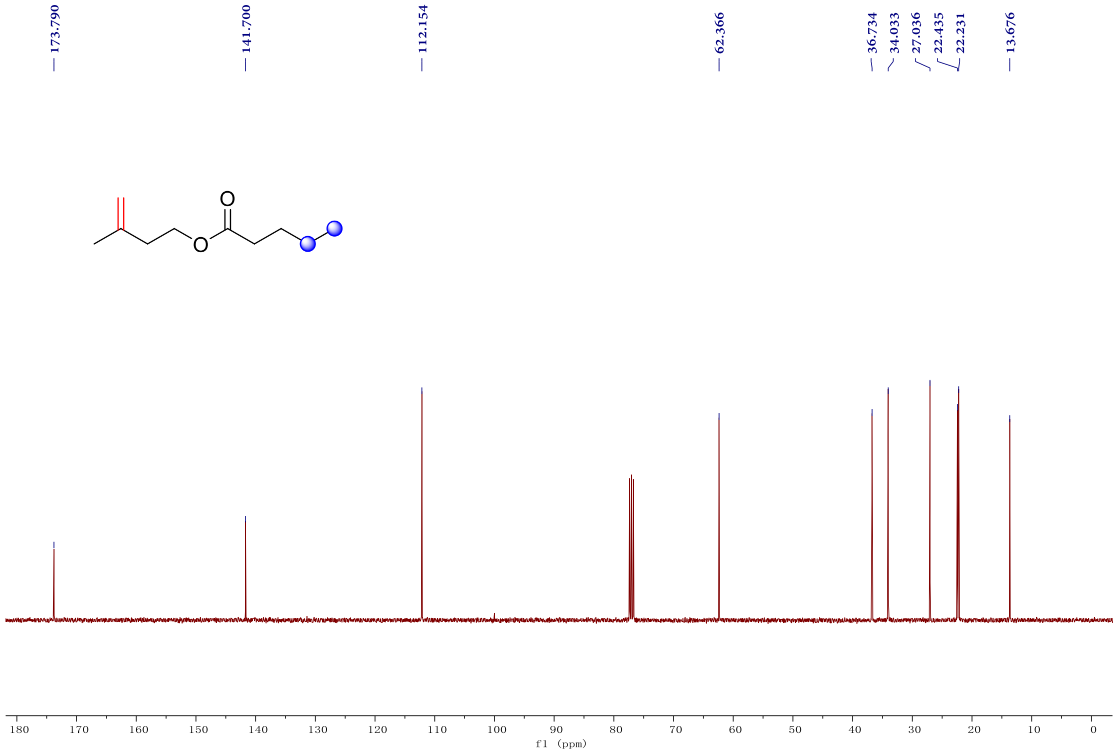

102

^{1}H NMR spectrum of **35** (**CDCl$_3$**)

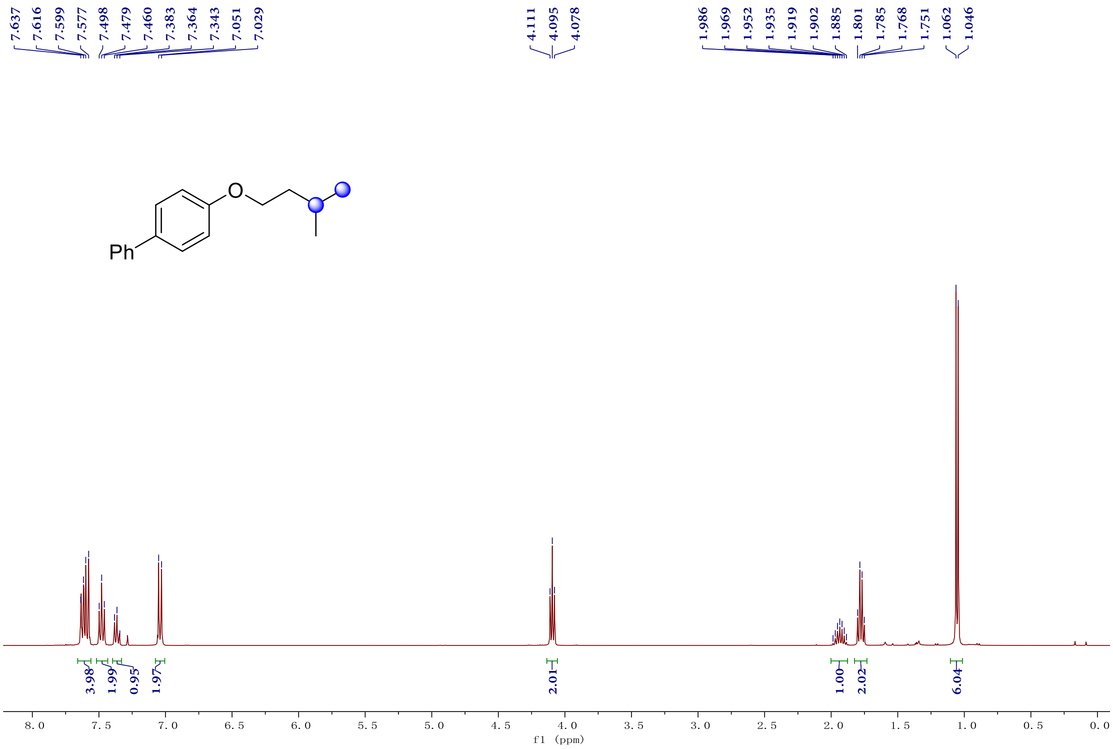

^{13}C NMR spectrum of **35** (**CDCl$_3$**)

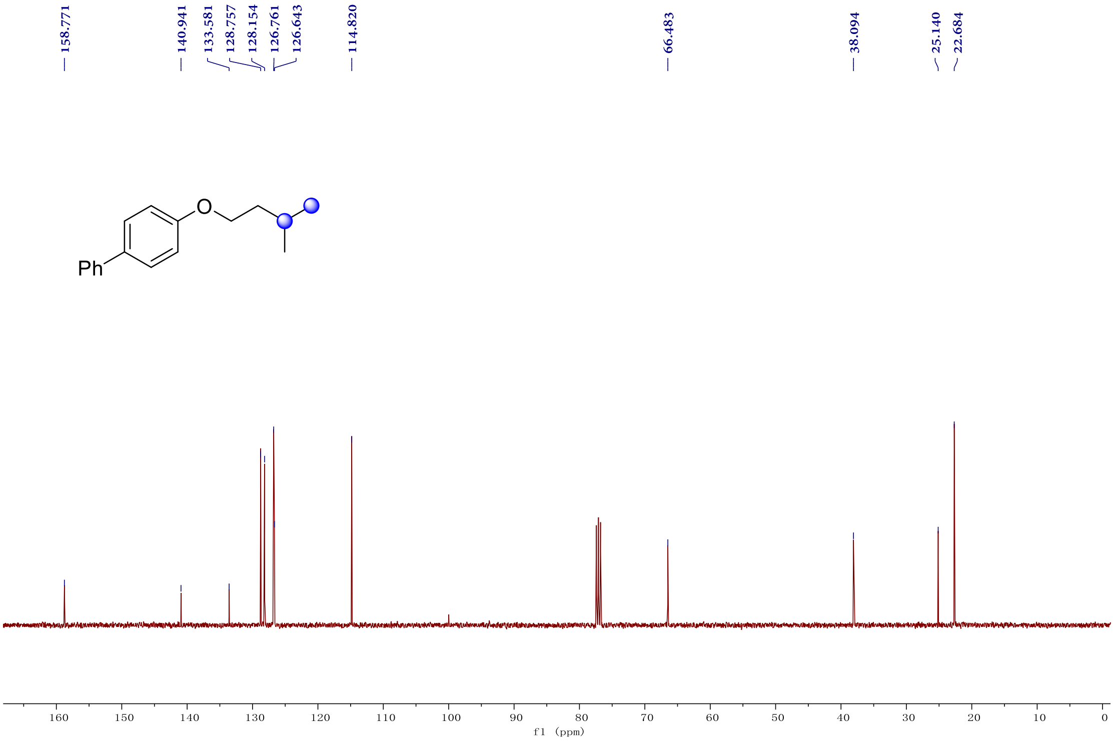

103

1417 ^{1}H NMR spectrum of **38** (**CDCl$_3$**)

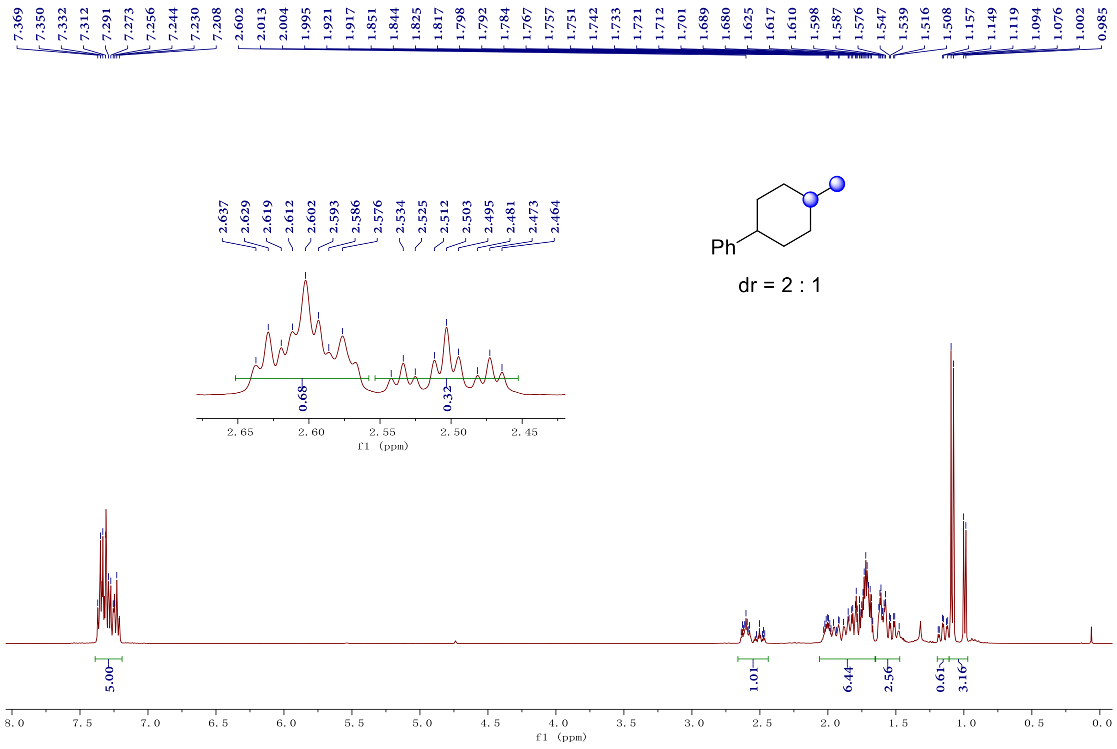

1418

1419 ^{13}C NMR spectrum of **38** (**CDCl$_3$**)

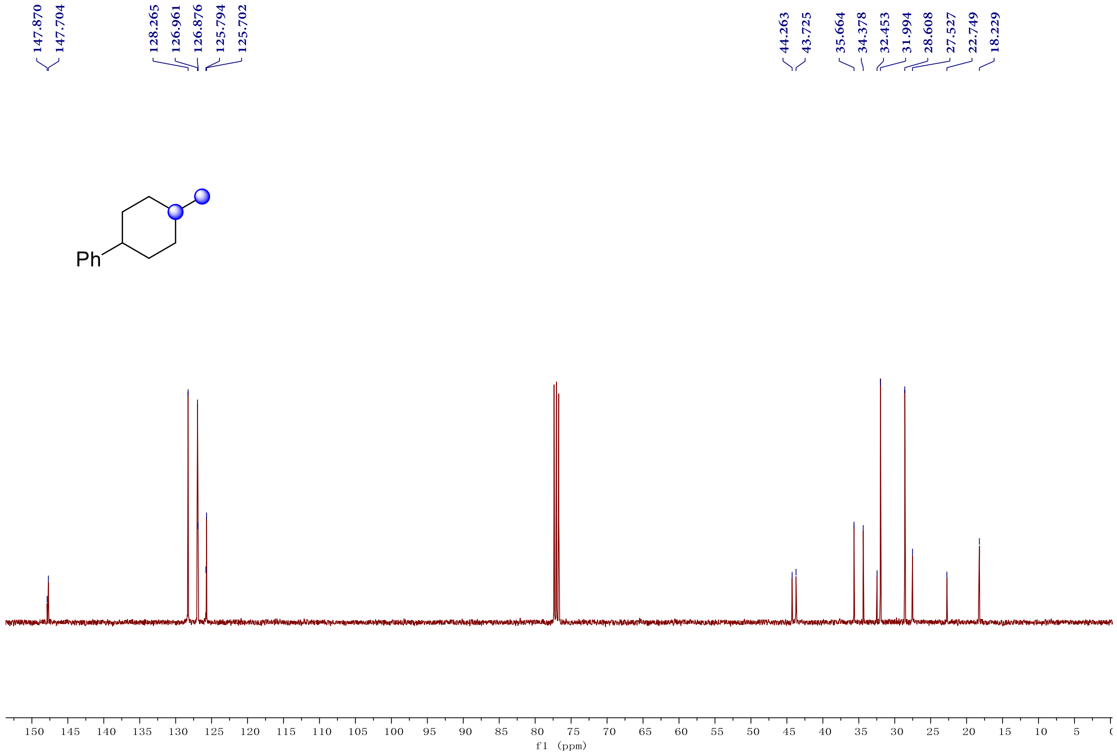

1420

1421

^{1}H NMR spectrum of **39** (**$CDCl_3$**)

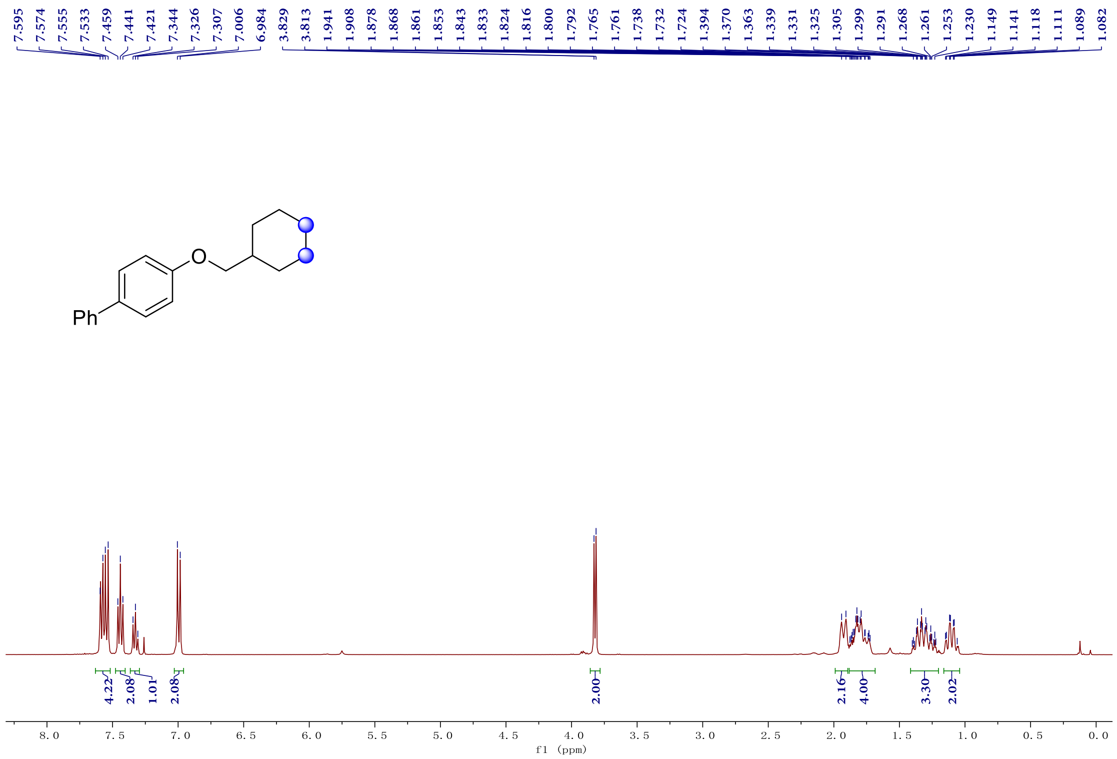

^{13}C NMR spectrum of **39** (**$CDCl_3$**)

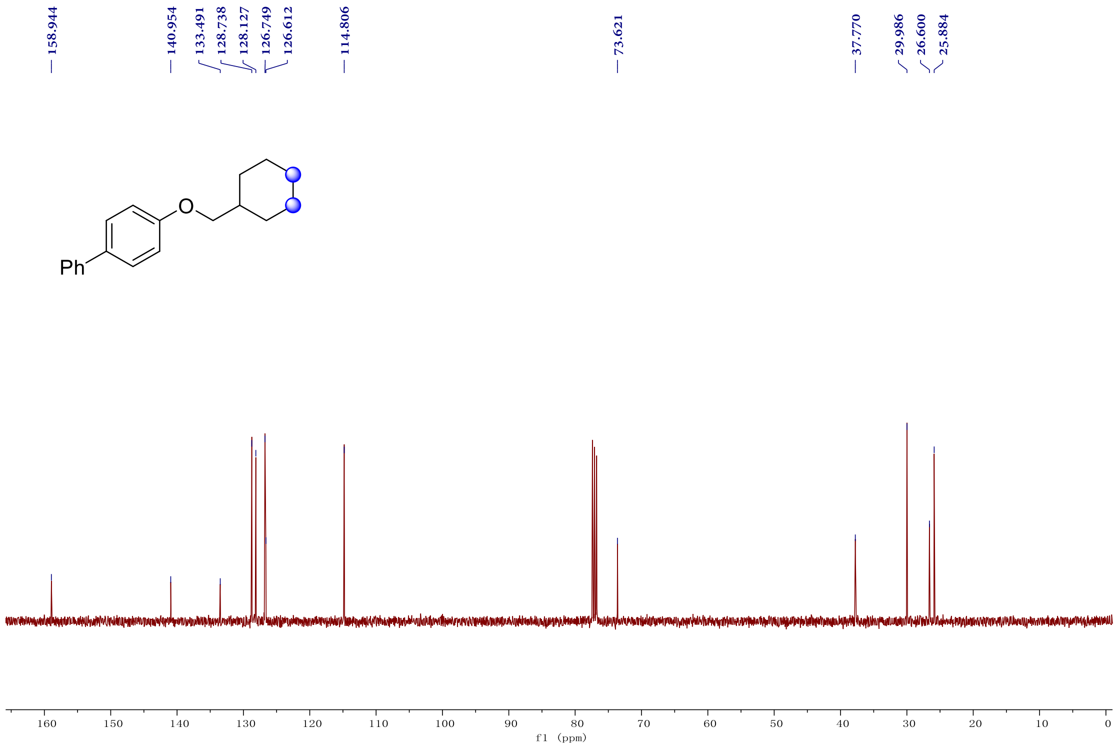

105

^{1}H NMR spectrum of **40** (**$CDCl_3$**)

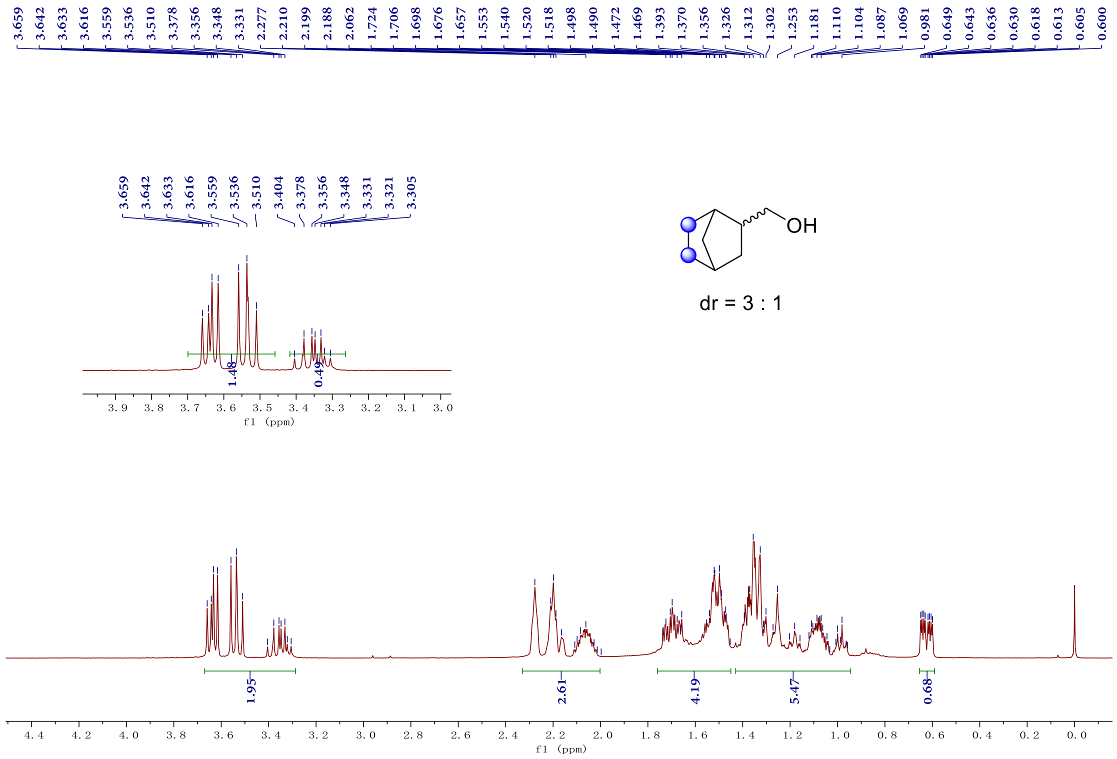

^{13}C NMR spectrum of **40** (**$CDCl_3$**)

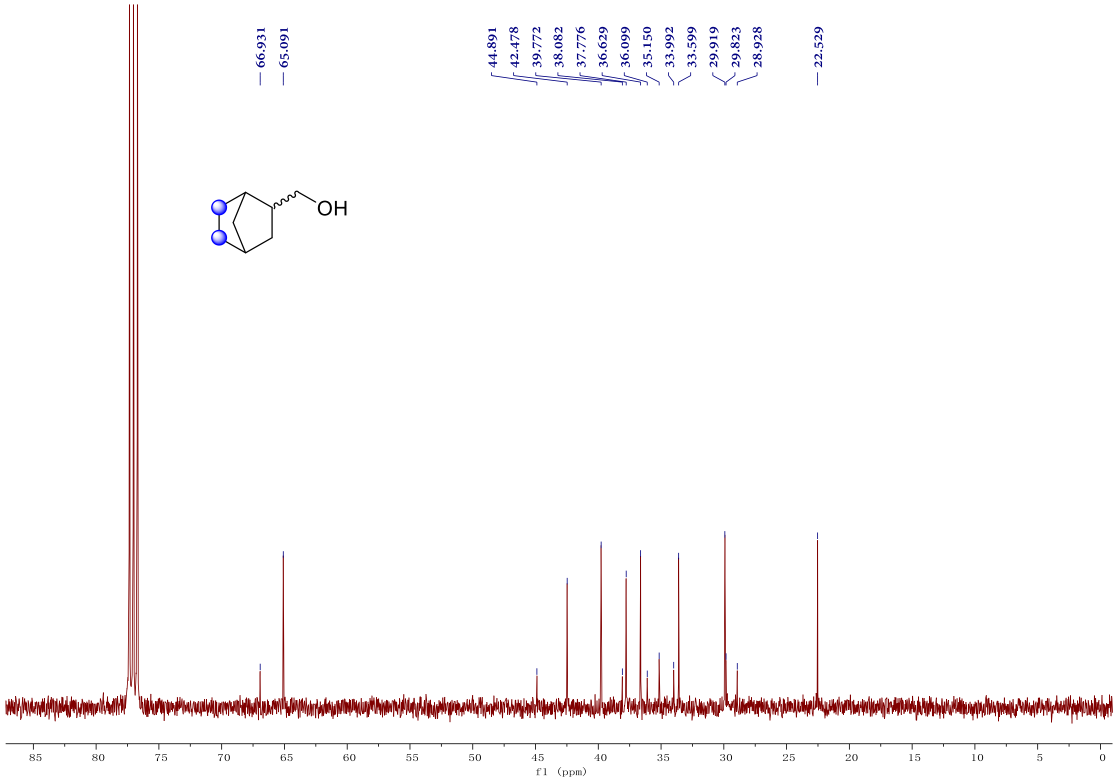

106

^{1}H NMR spectrum of **45** (**CDCl$_3$**)

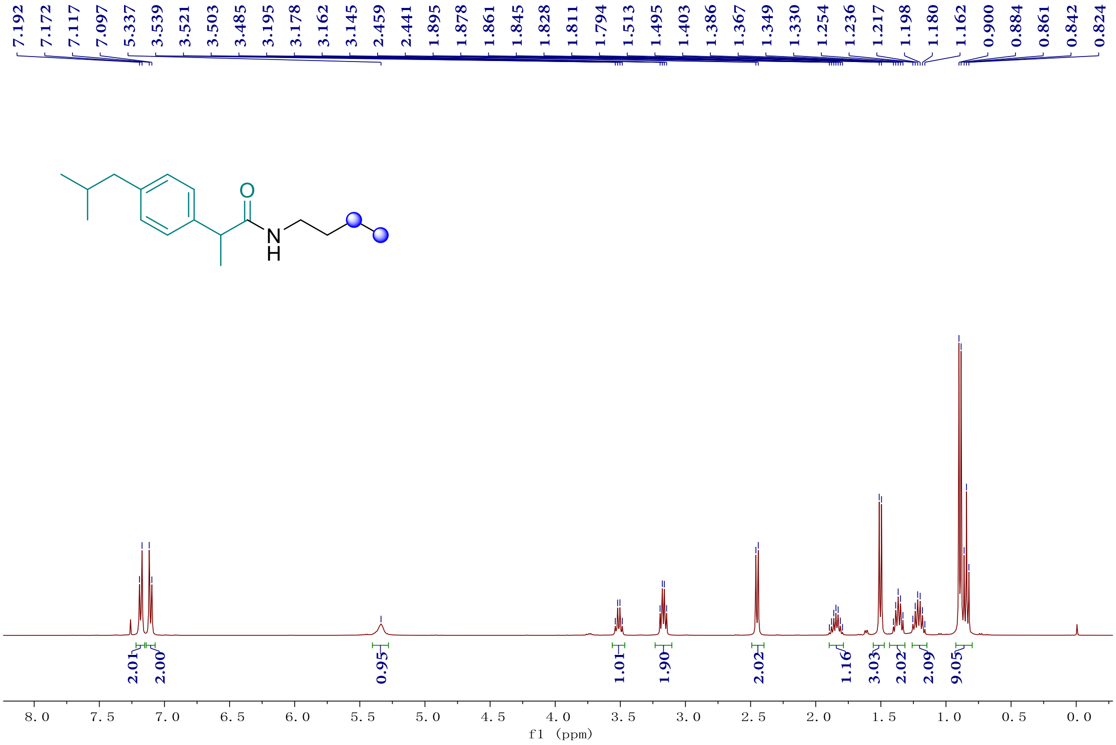

^{13}C NMR spectrum of **45** (**CDCl$_3$**)

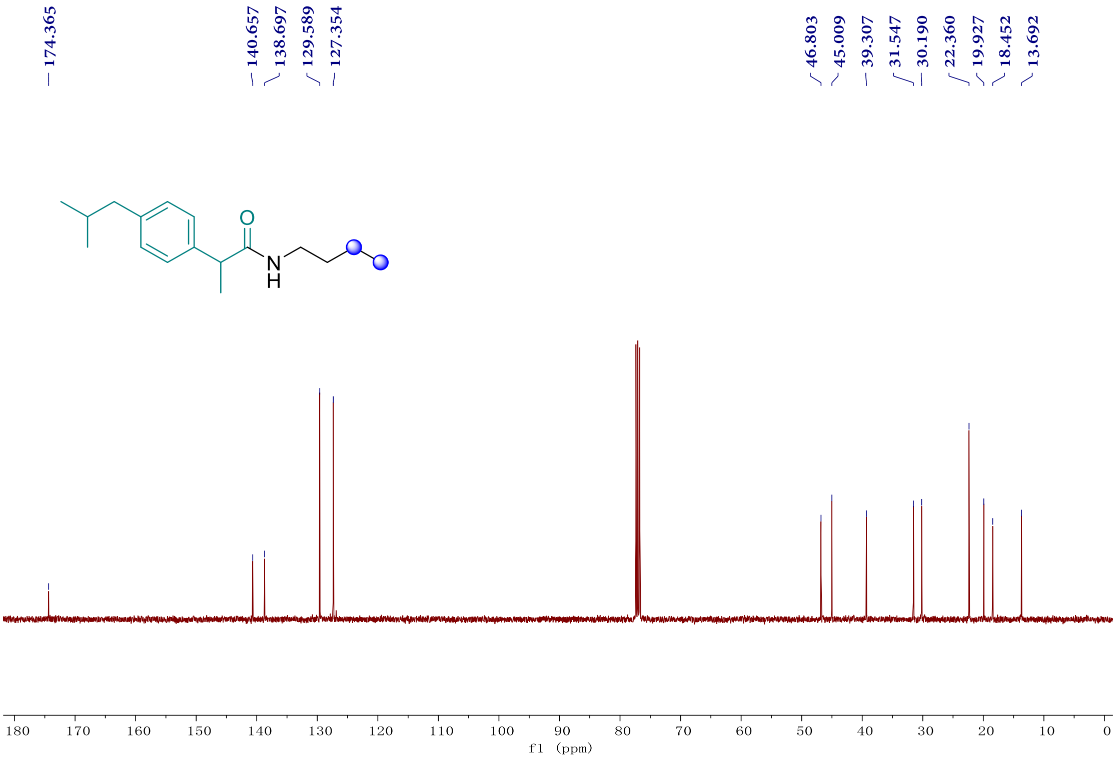

107

^{1}H NMR spectrum of **46** (**CDCl$_3$**)

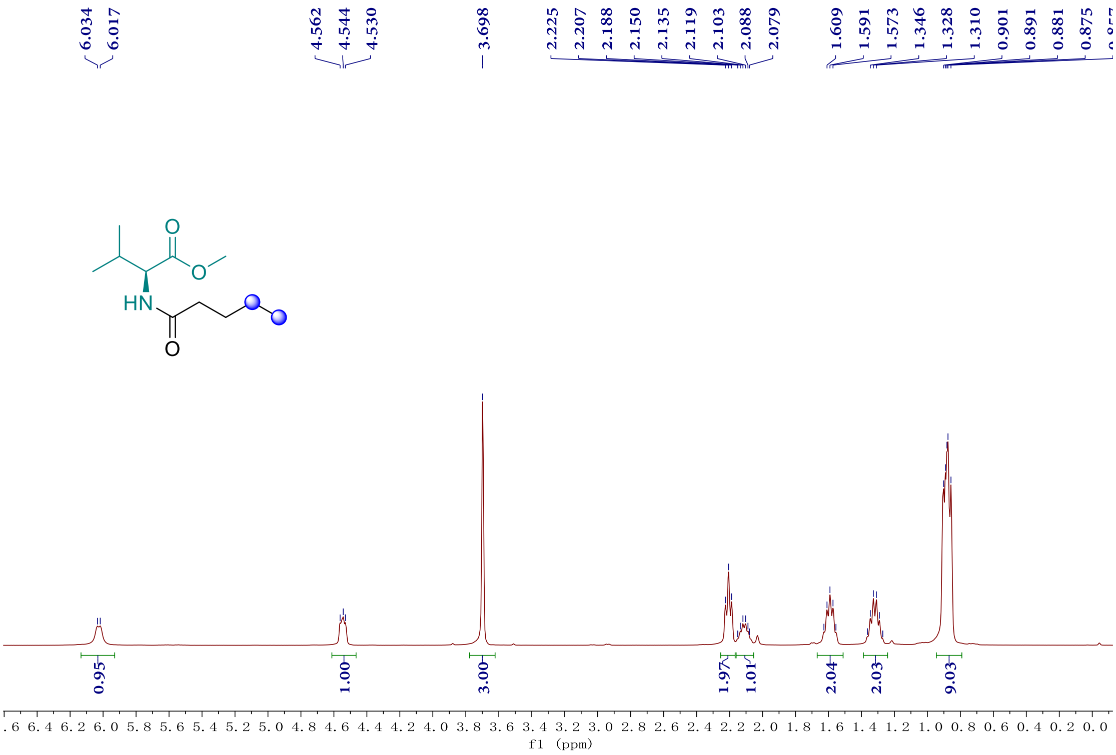

^{13}C NMR spectrum of **46** (**CDCl$_3$**)

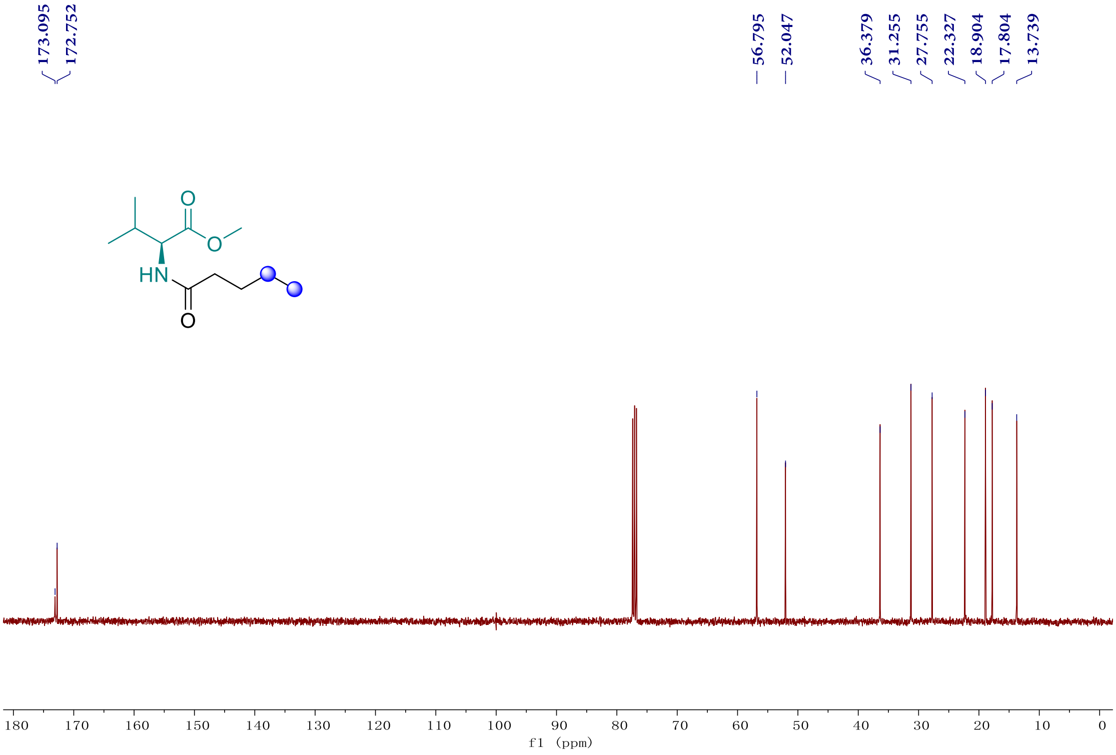

108

^{1}H NMR spectrum of **47** (**$CDCl_3$**)

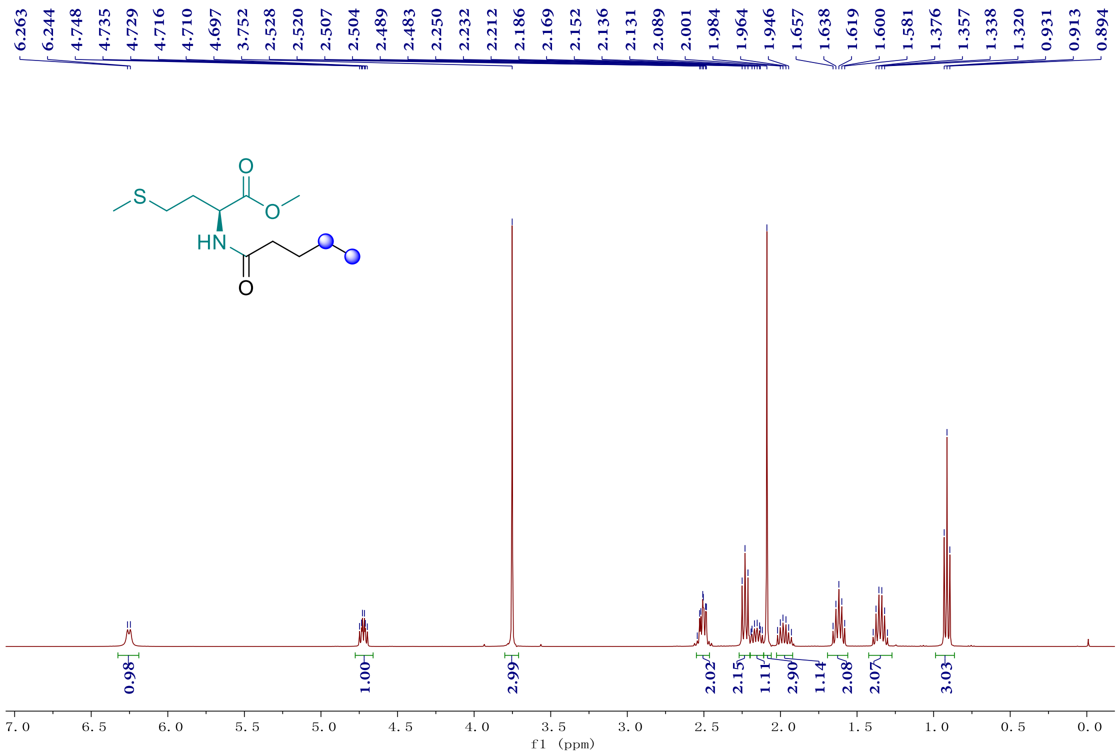

^{13}C NMR spectrum of **47** (**$CDCl_3$**)

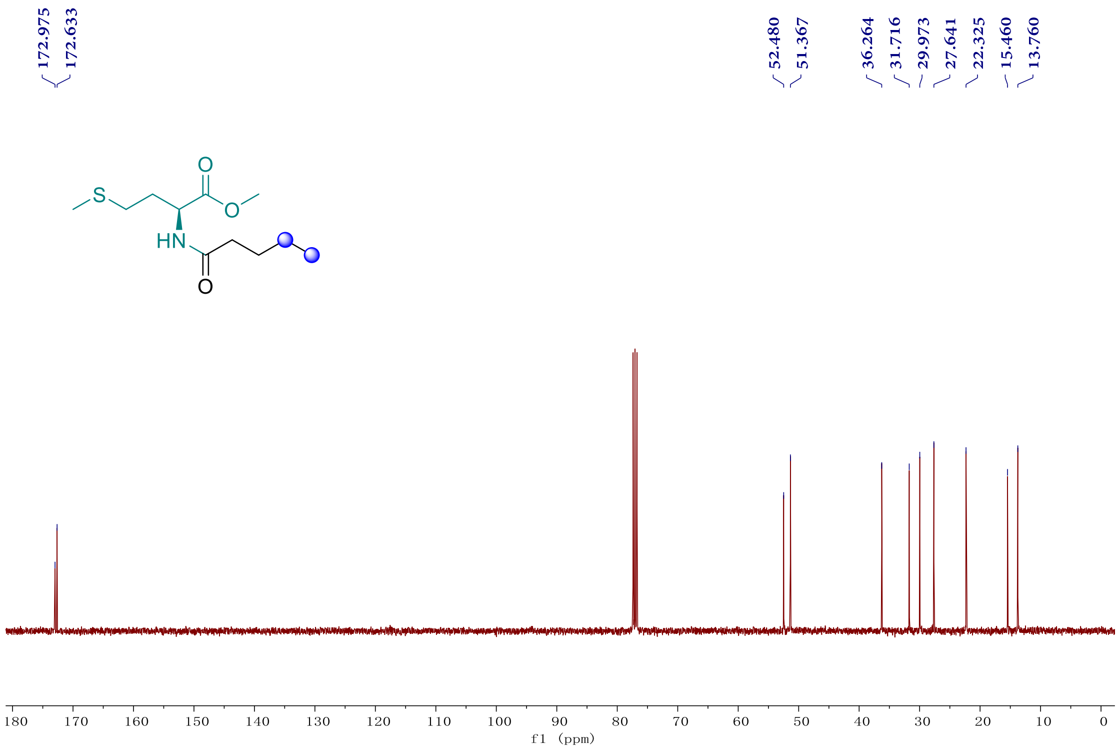

109

^{1}H NMR spectrum of **48** (**CDCl$_3$**)

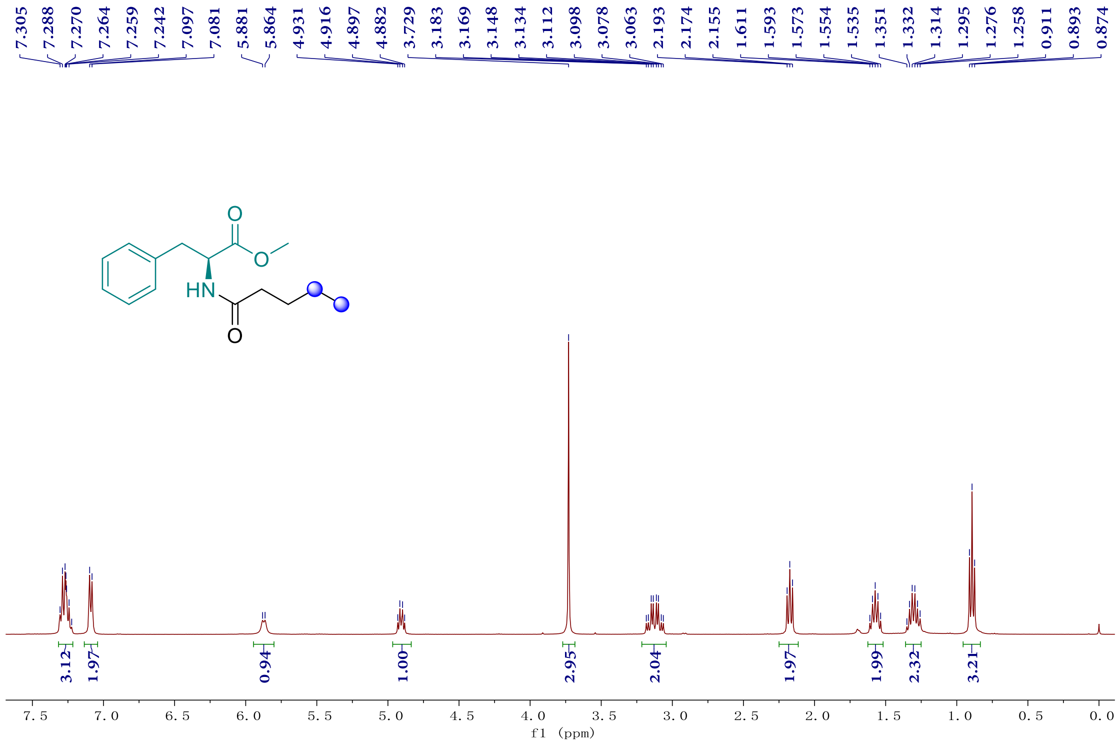

^{13}C NMR spectrum of **48** (**CDCl$_3$**)

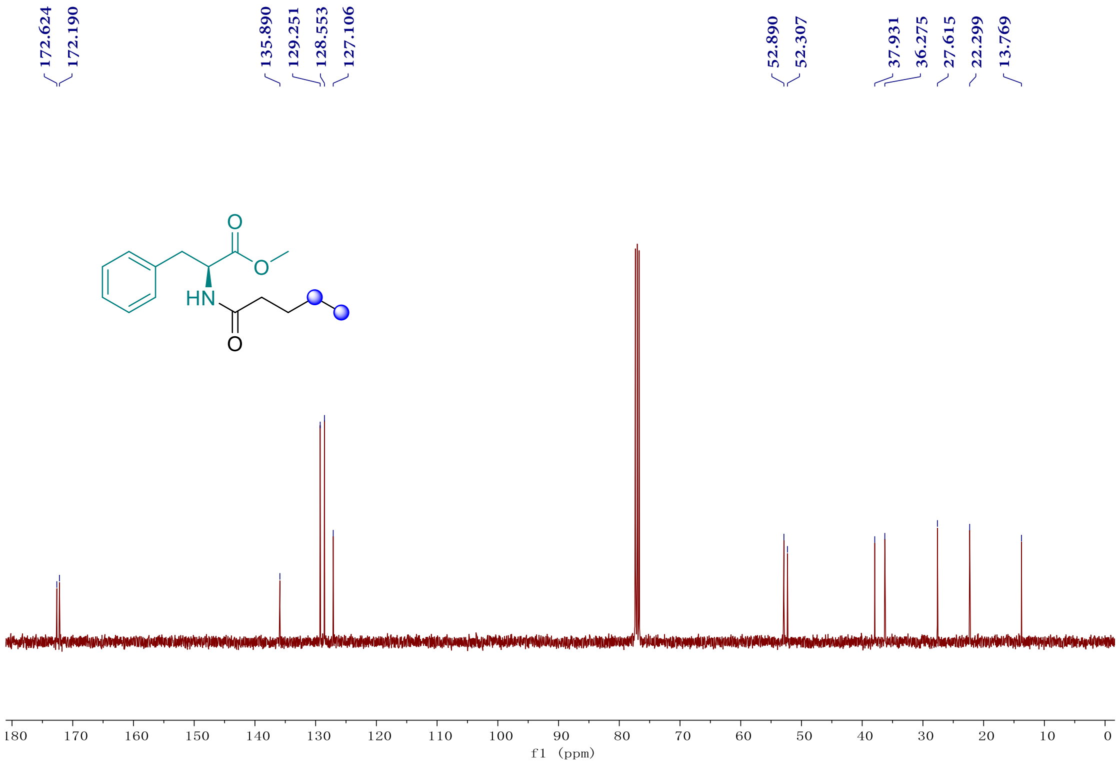

110

^{1}H NMR spectrum of **49** (**$CDCl_3$**)

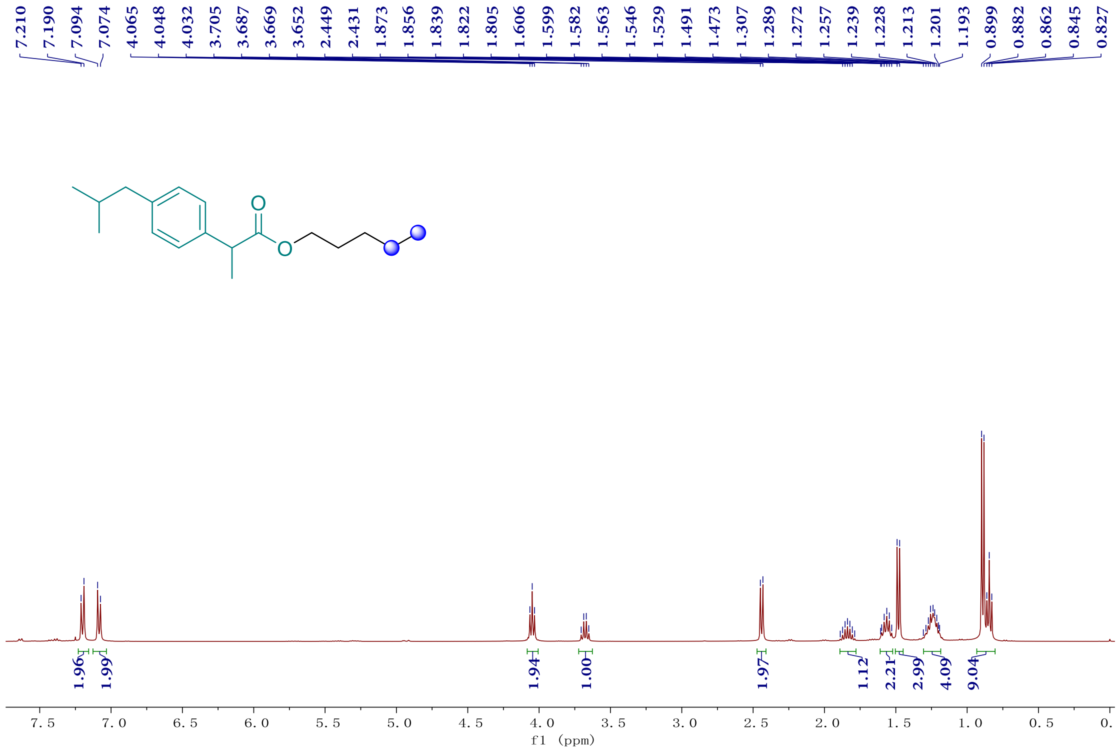

^{13}C NMR spectrum of **49** (**$CDCl_3$**)

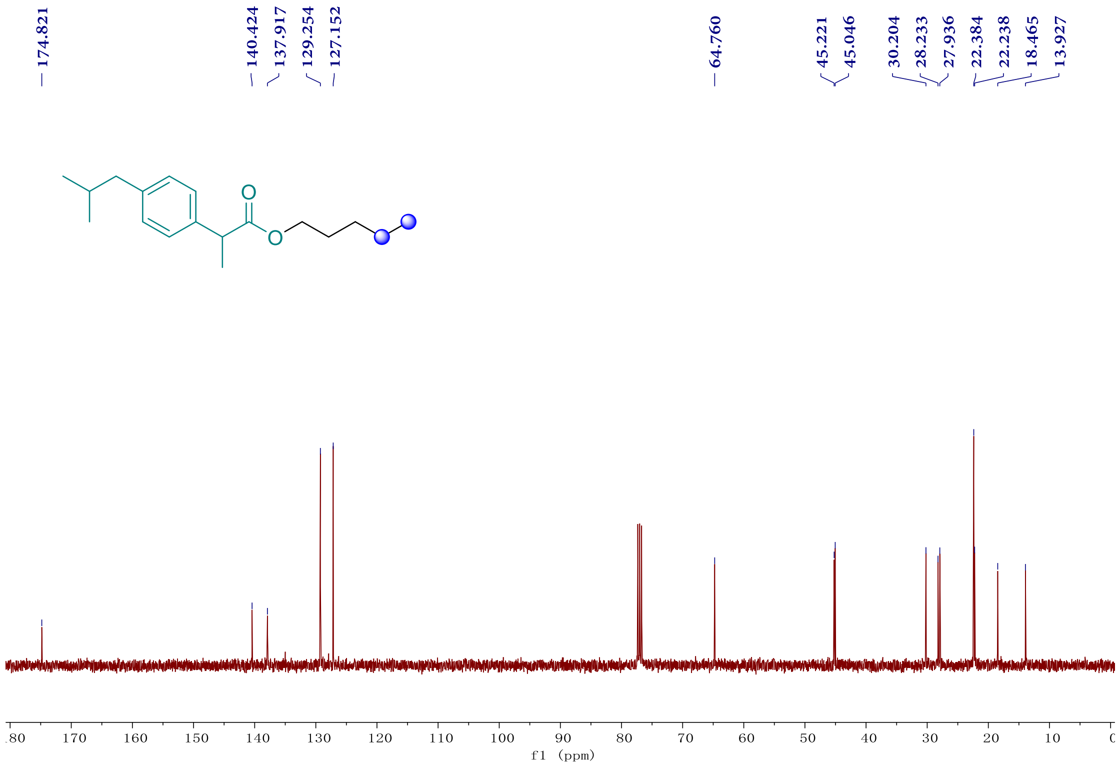

111

^{1}H NMR spectrum of **50** (**CDCl$_3$**)

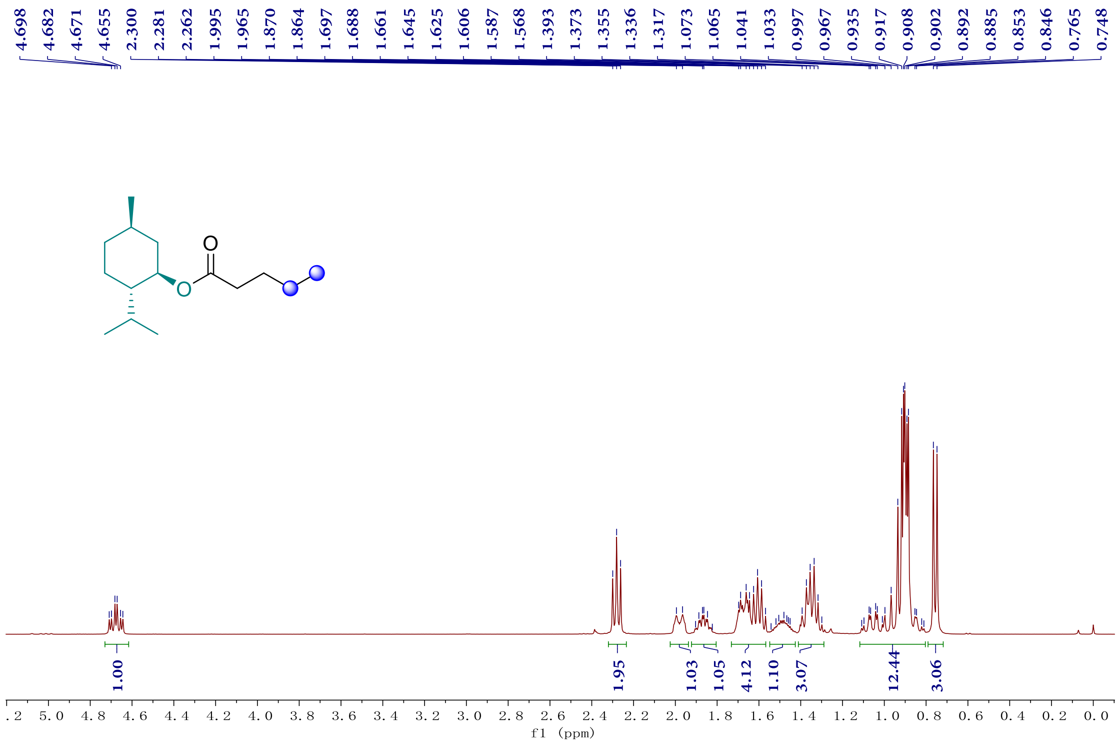

^{13}C NMR spectrum of **50** (**CDCl$_3$**)

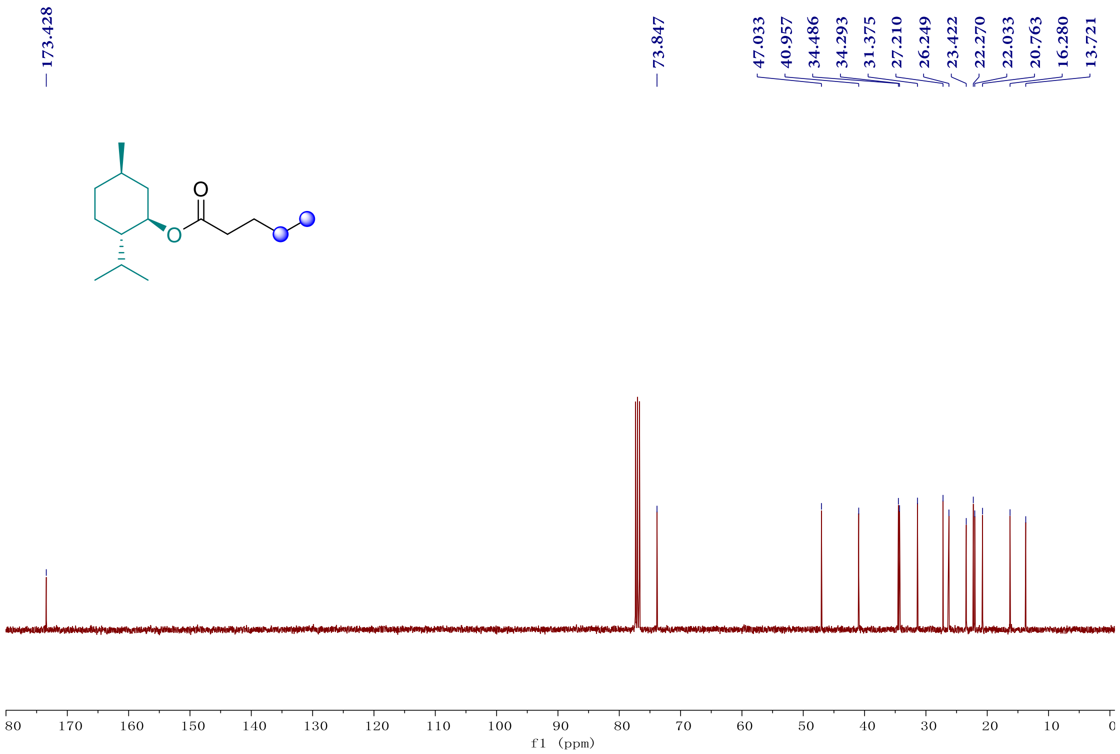

112

^{1}H NMR spectrum of **51** (**CDCl$_3$**)

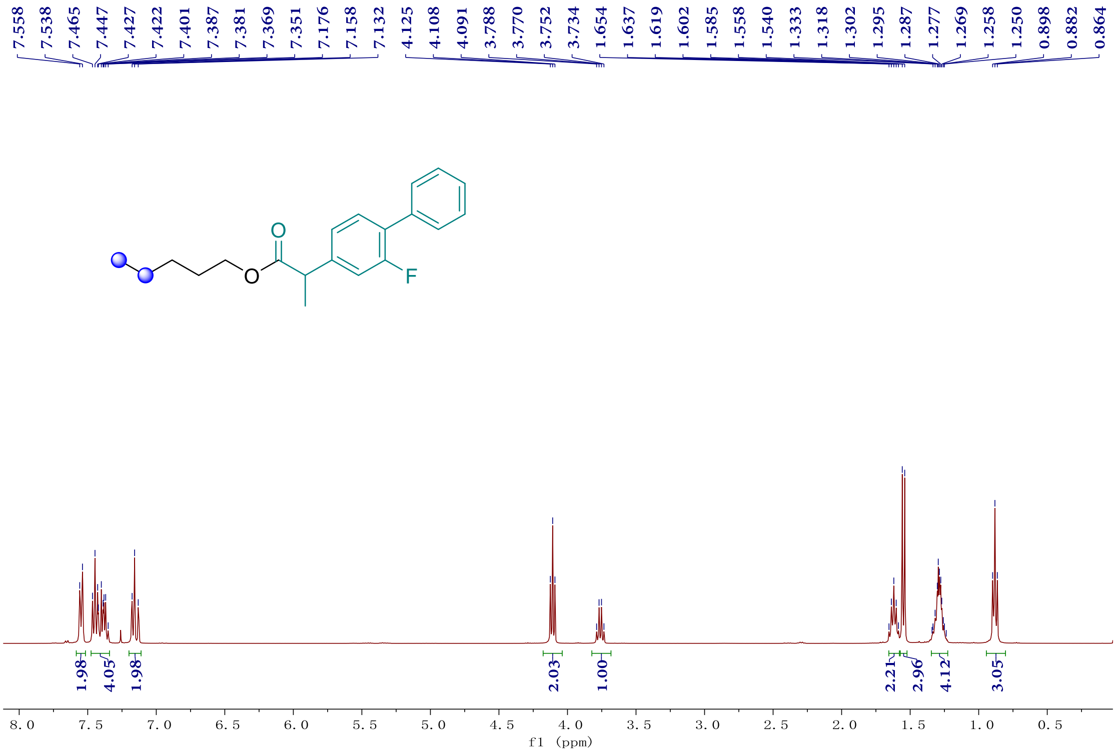

^{13}C NMR spectrum of **51** (**CDCl$_3$**)

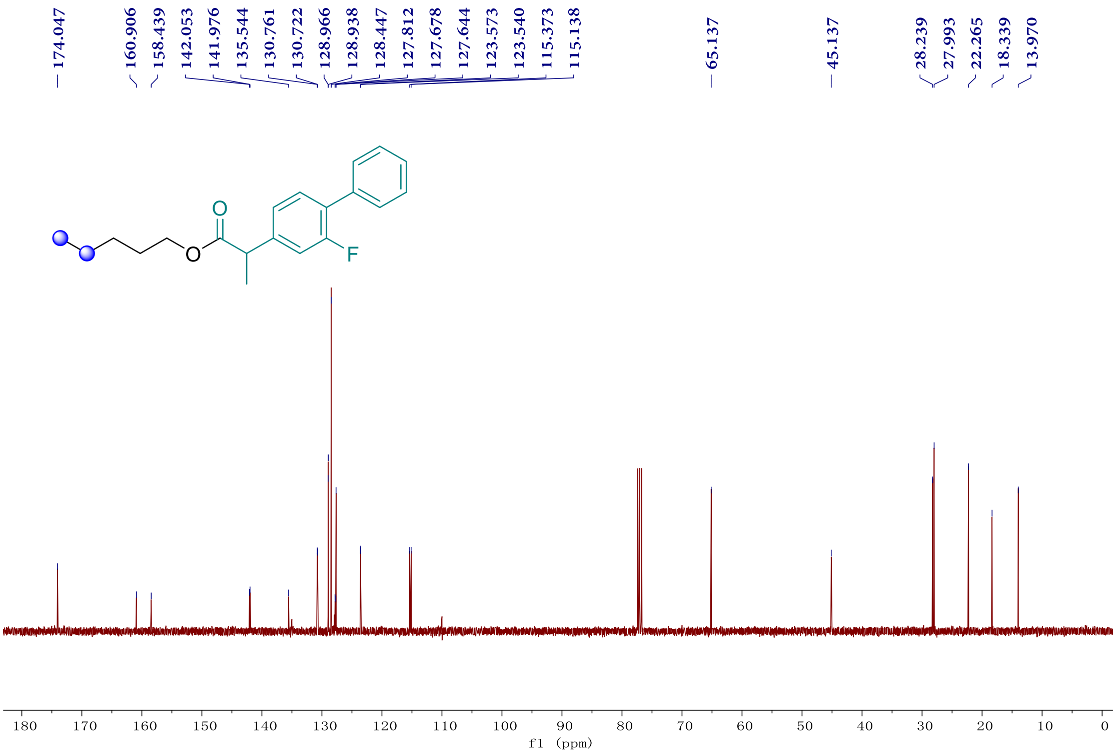

113

^{19}F NMR spectrum of **51** (**$CDCl_3$**)

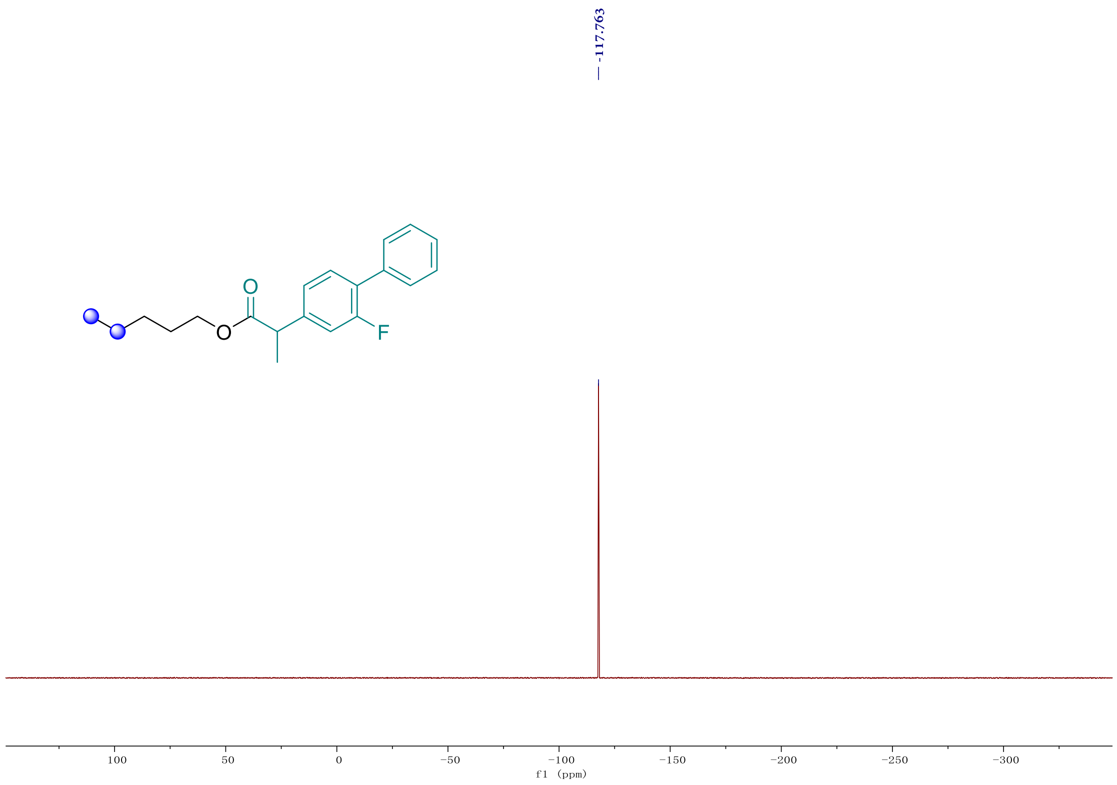

114

^{1}H NMR spectrum of **52** (**$CDCl_3$**)

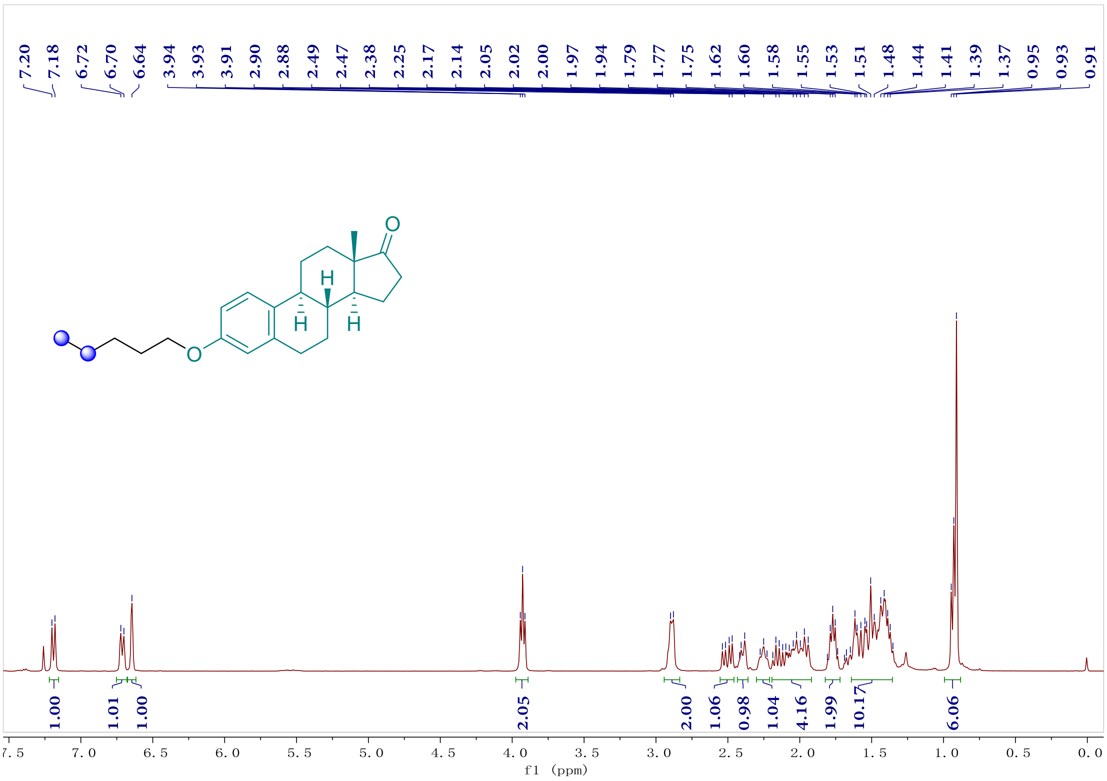

^{13}C NMR spectrum of **52** (**$CDCl_3$**)

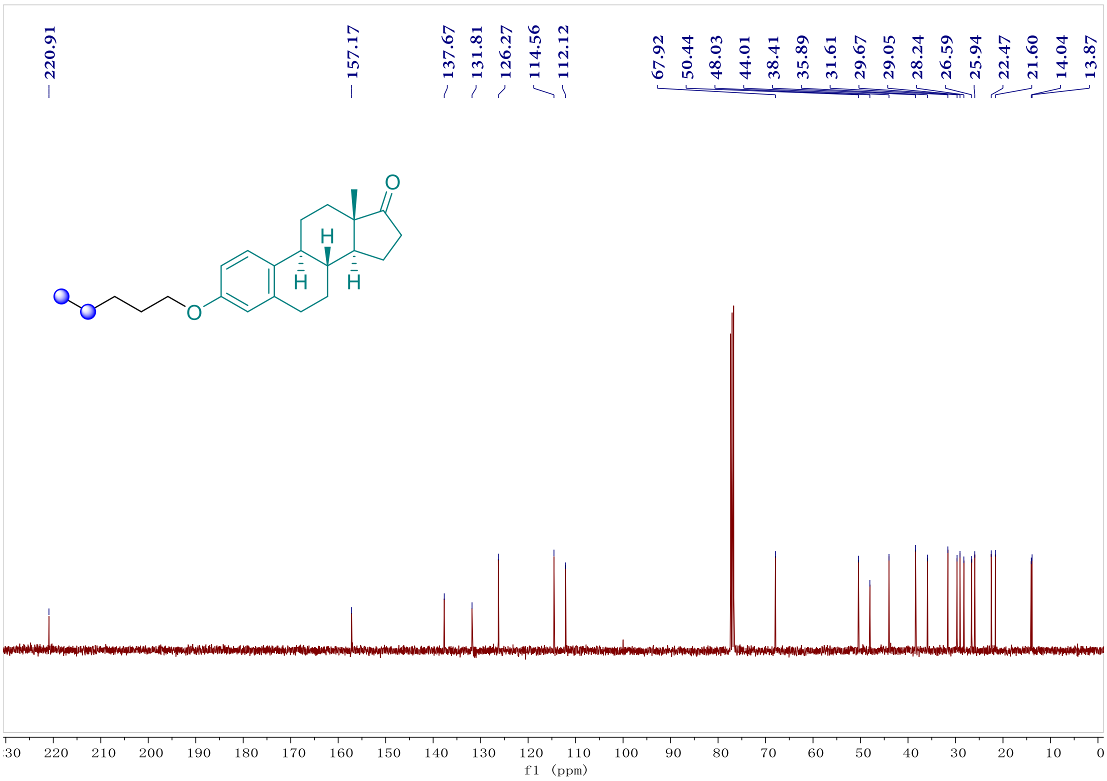

^{1}H NMR spectrum of **53** (**CDCl$_3$**)

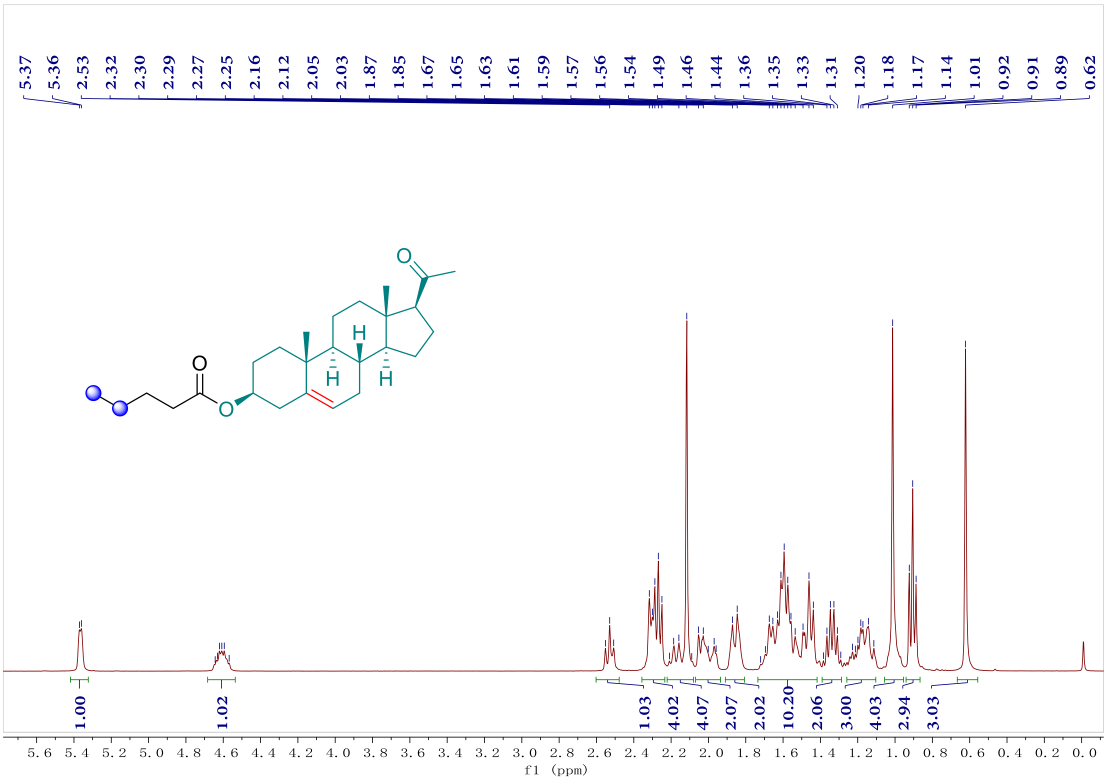

^{13}C NMR spectrum of **53** (**CDCl$_3$**)

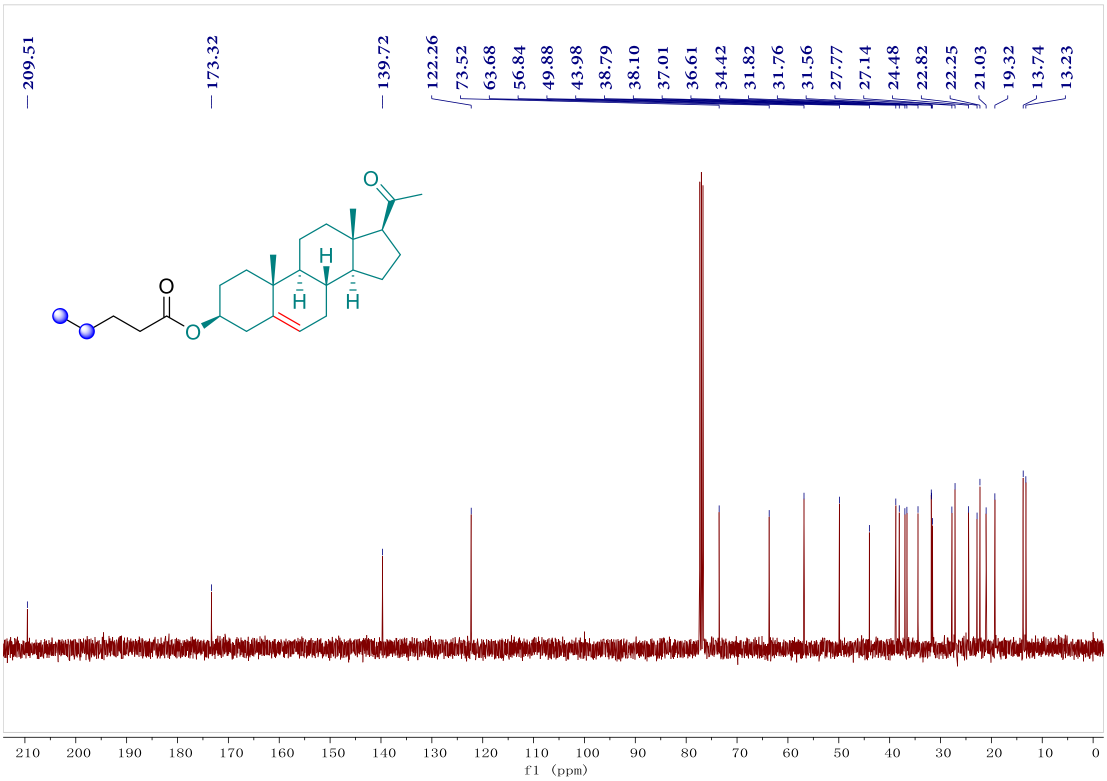

116

^{1}H NMR spectrum of **54** (**CDCl$_3$**)

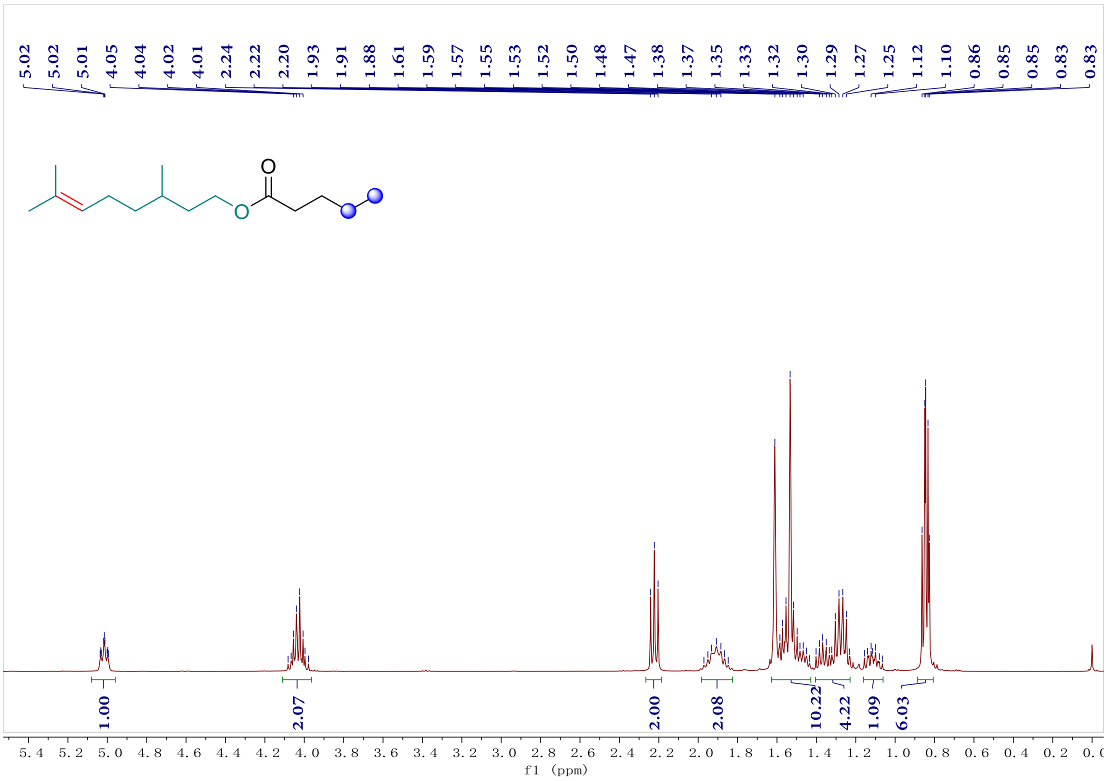

^{13}C NMR spectrum of **54** (**CDCl$_3$**)

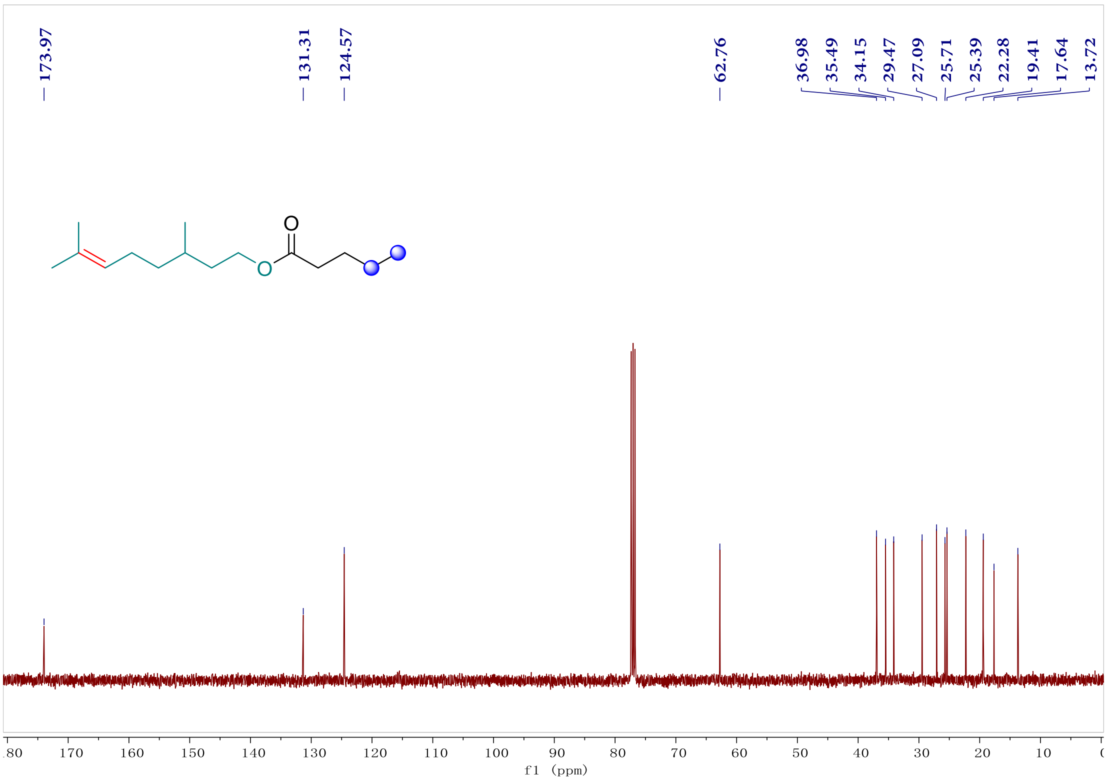

117

^{1}H NMR spectrum of **55** (**$CDCl_3$**)

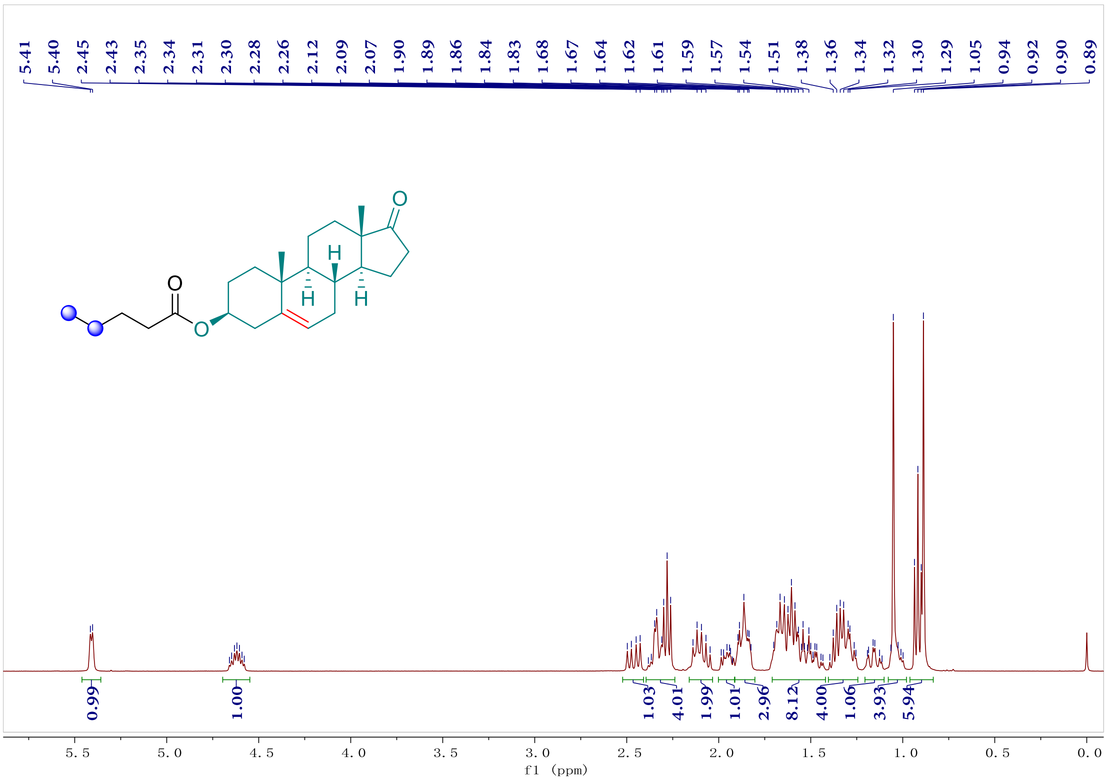

^{13}C NMR spectrum of **55** (**$CDCl_3$**)

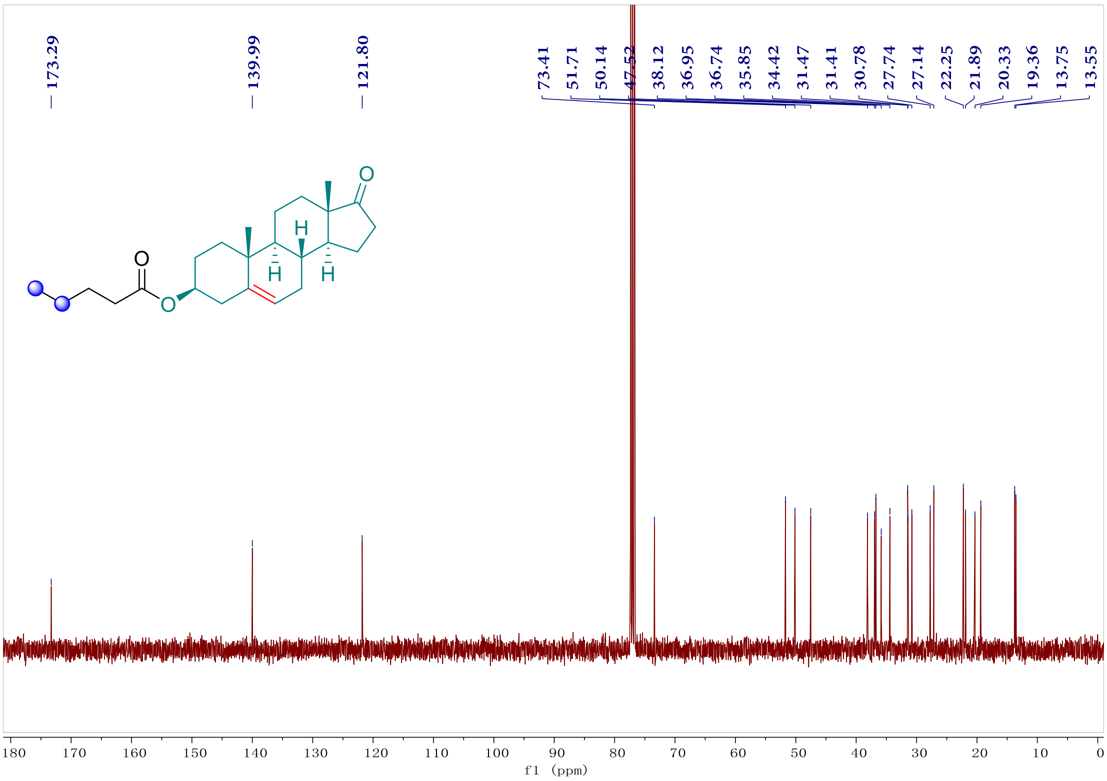

118

^{1}H NMR spectrum of **56** (**$CDCl_3$**)

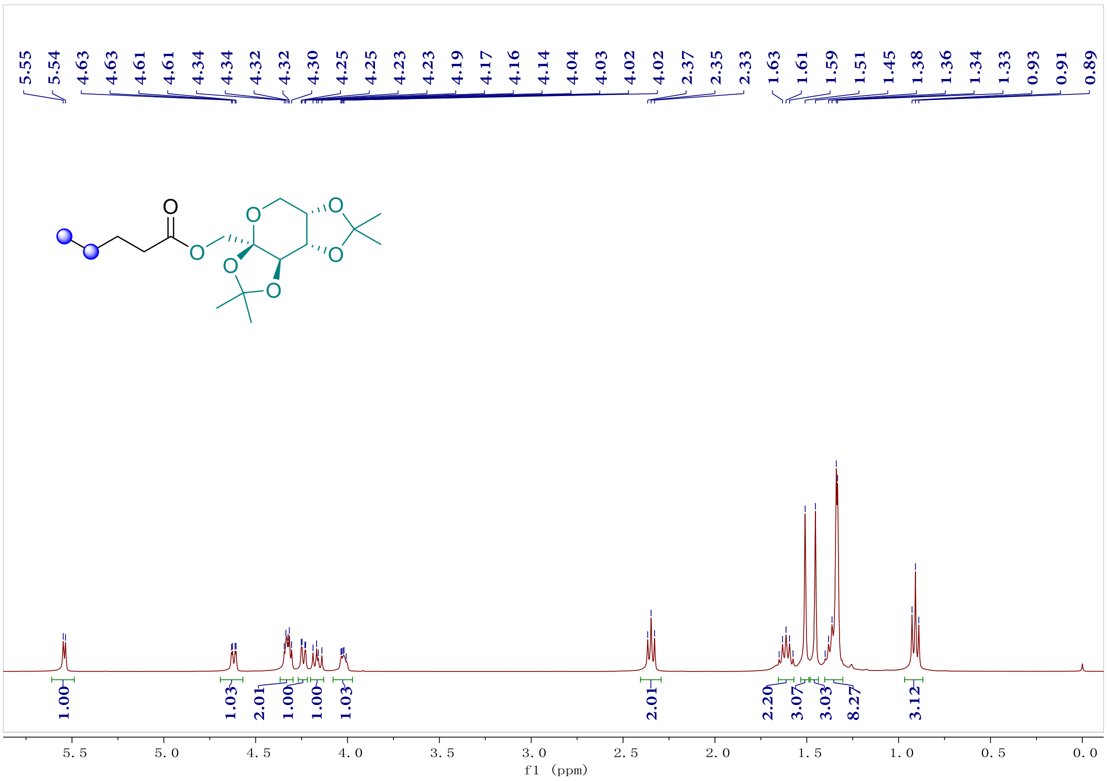

^{13}C NMR spectrum of **56** (**$CDCl_3$**)

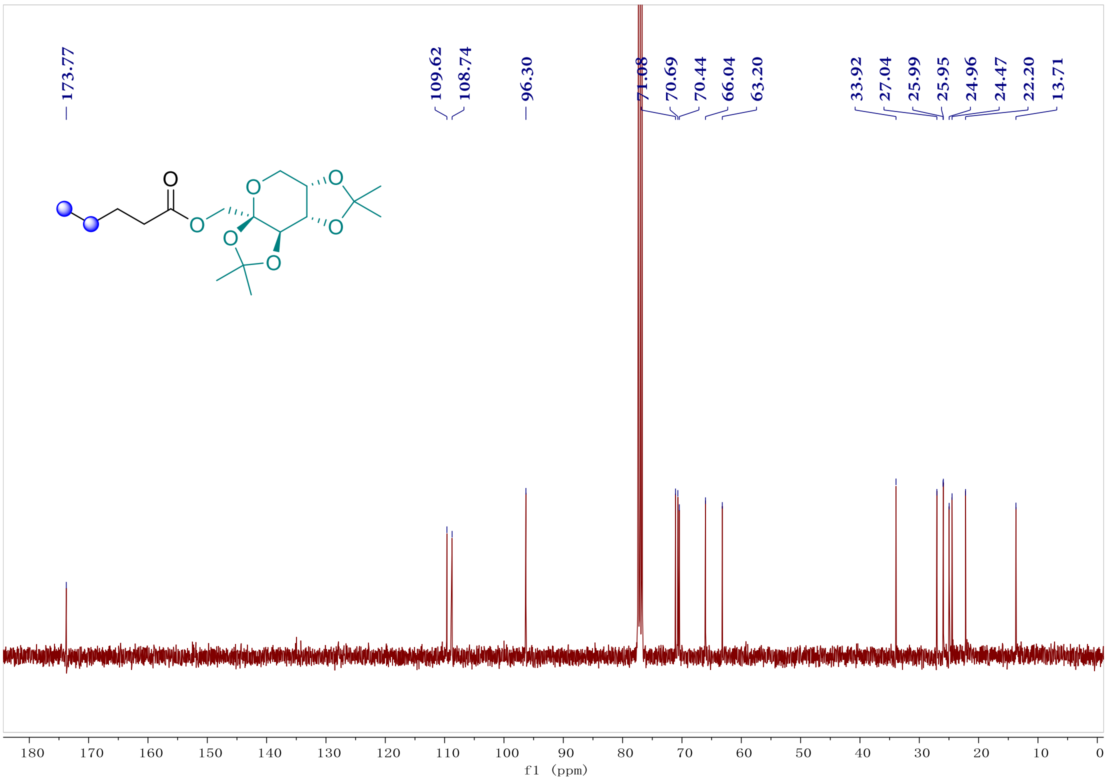

119

1496 ^{1}H NMR spectrum of **57** (**$CDCl_3$**)

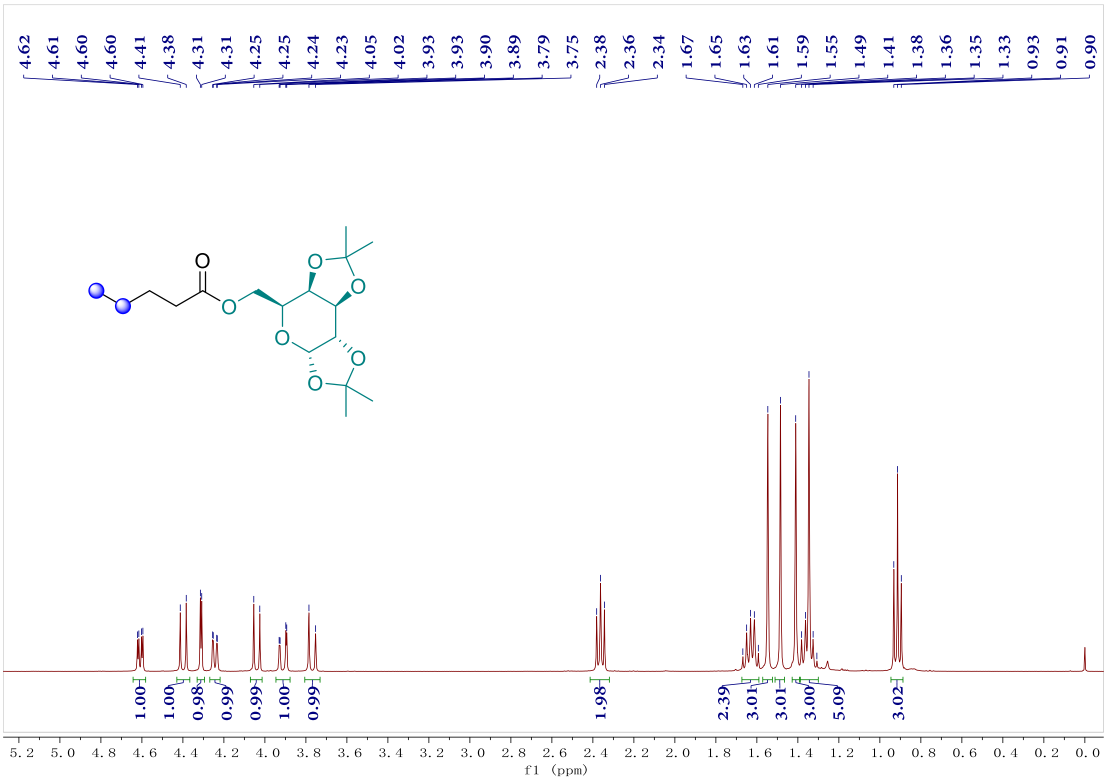

1497

1498 ^{13}C NMR spectrum of **57** (**$CDCl_3$**)

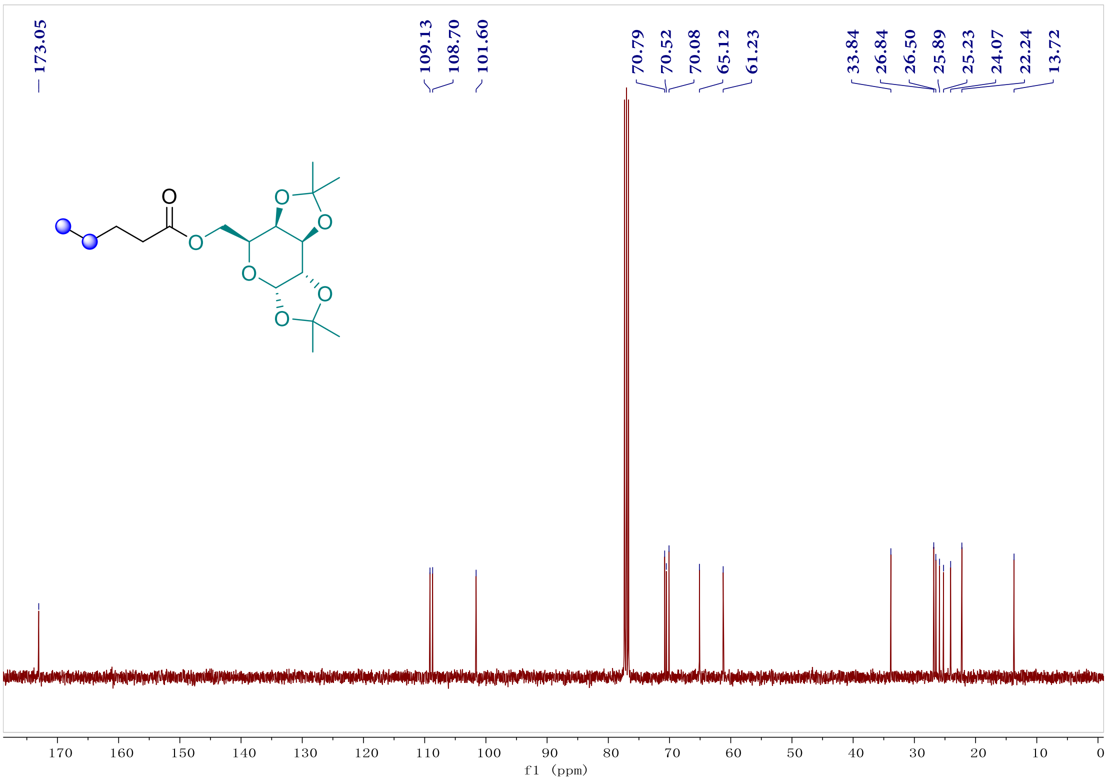

1499

1500

^{1}H NMR spectrum of **58** ($CDCl_3$)

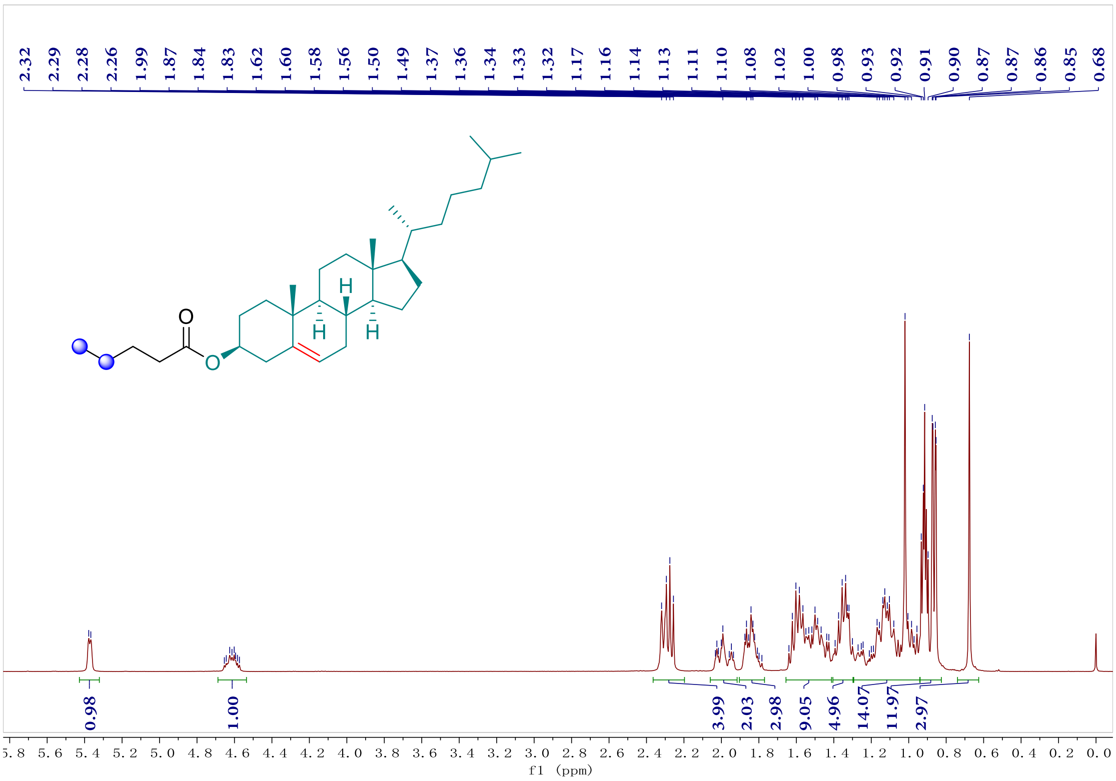

^{13}C NMR spectrum of **58** ($CDCl_3$)

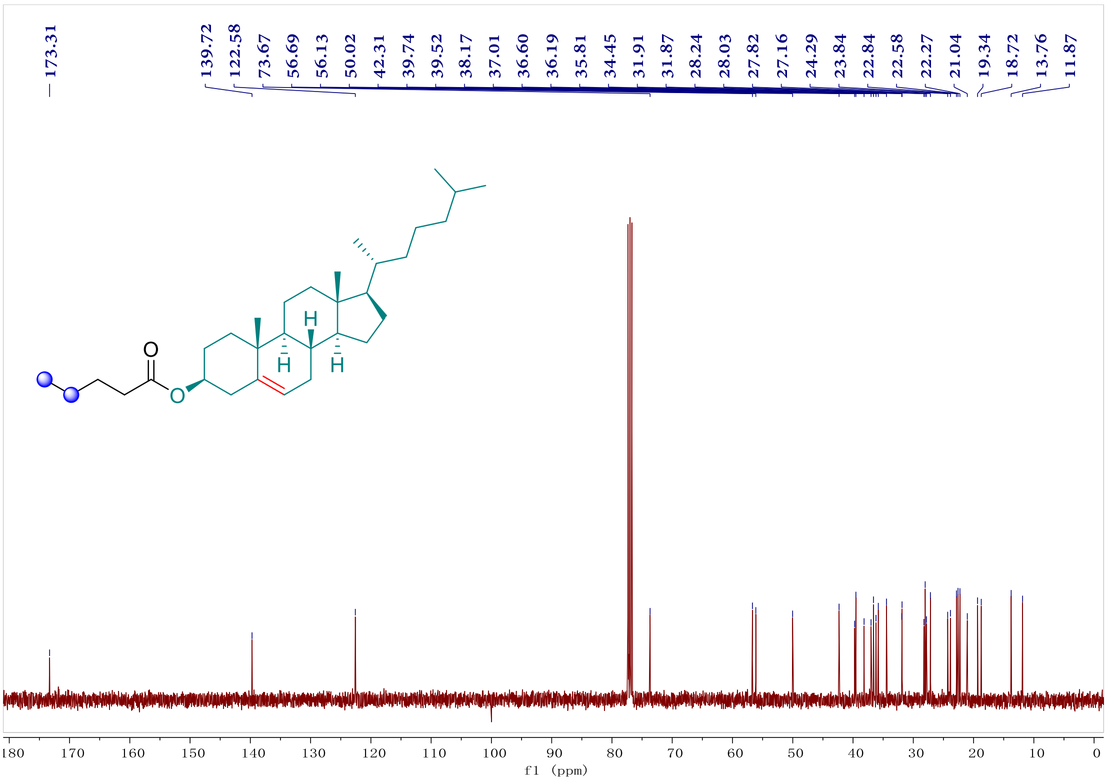

121

^{1}H NMR spectrum of **59** (**CDCl$_3$**)

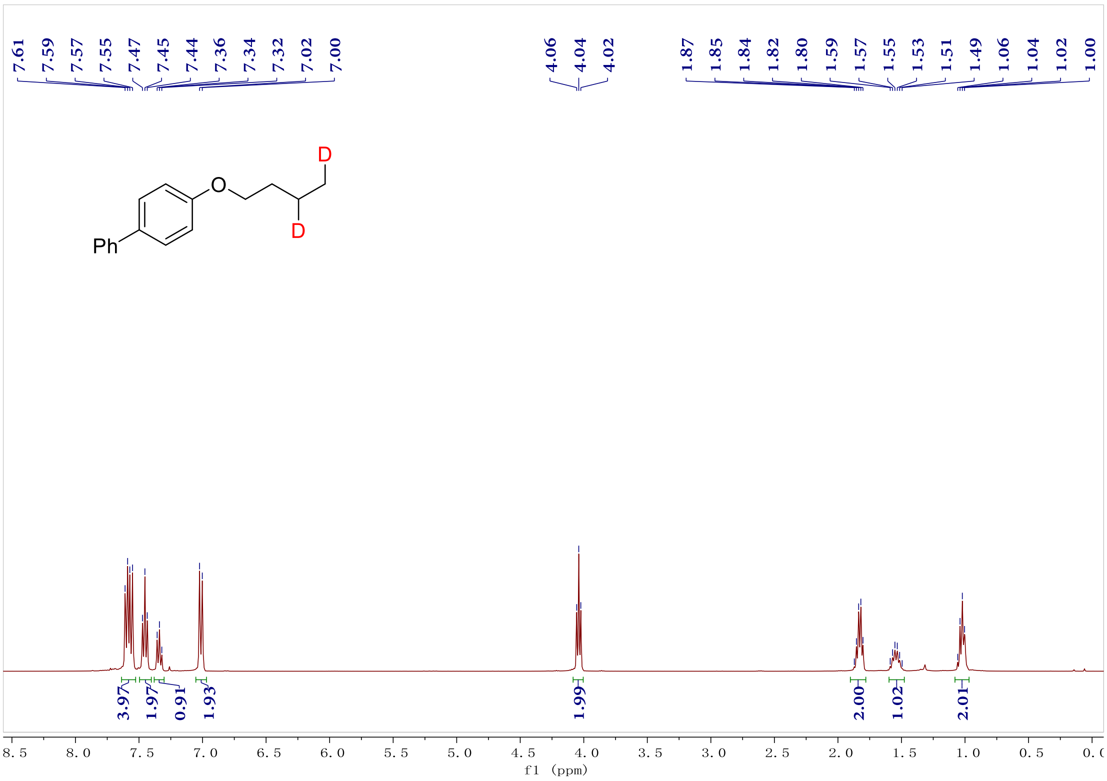

^{13}C NMR spectrum of **59** (**CDCl$_3$**)

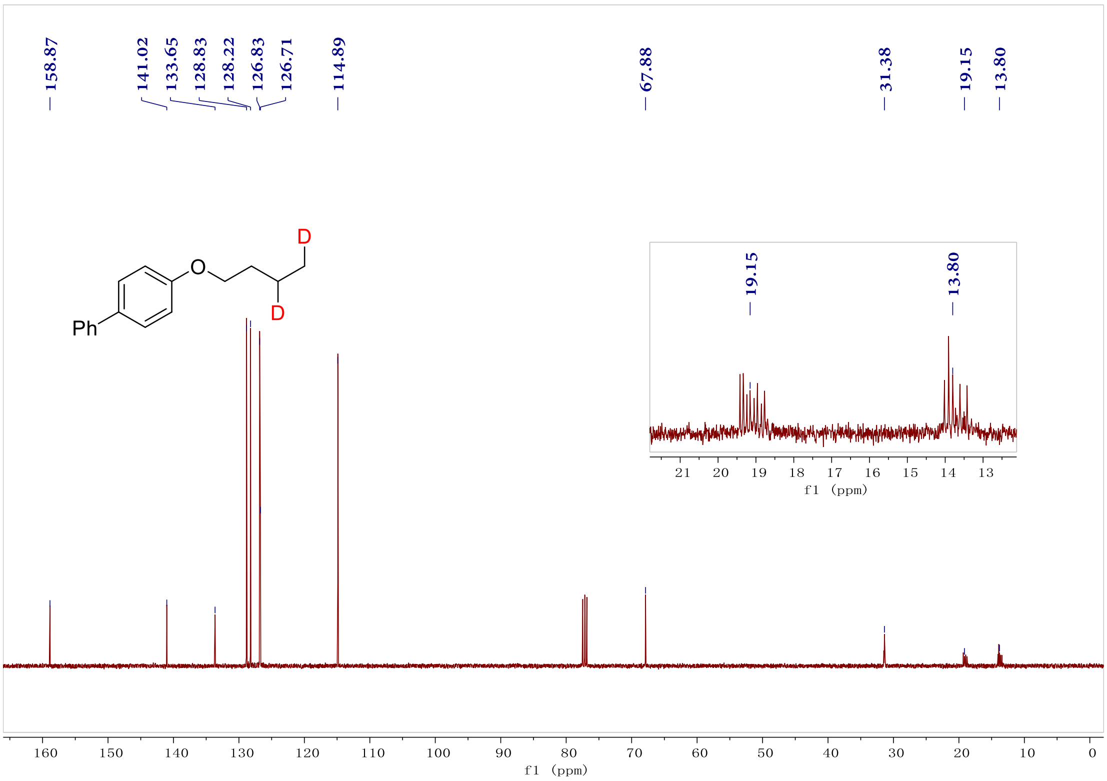

122

^{1}H NMR spectrum of **60** (**$CDCl_3$**)

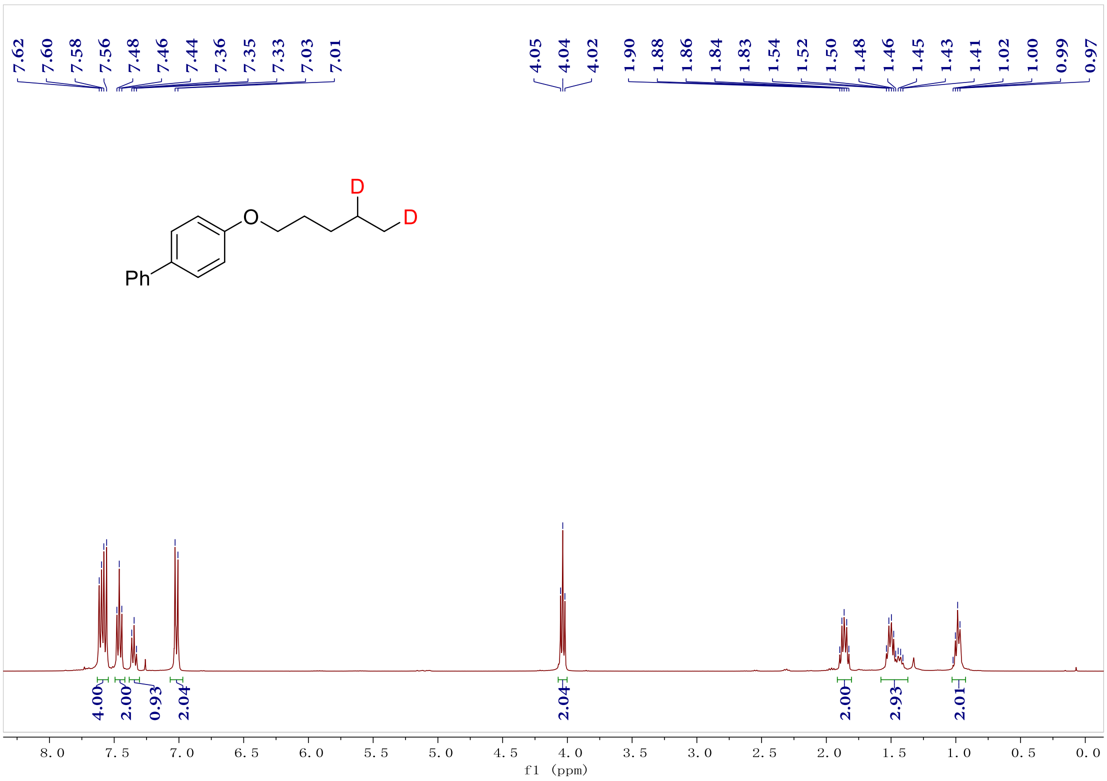

^{13}C NMR spectrum of **60** (**$CDCl_3$**)

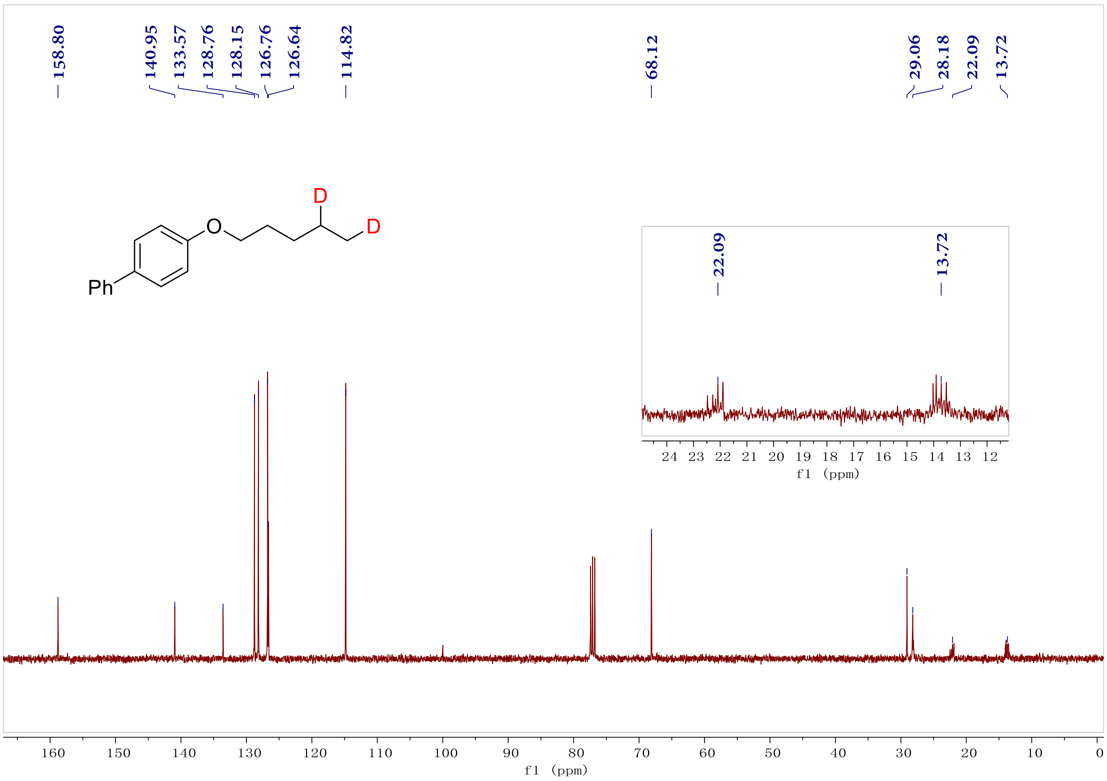

123

^{1}H NMR spectrum of **61** (**$CDCl_3$**)

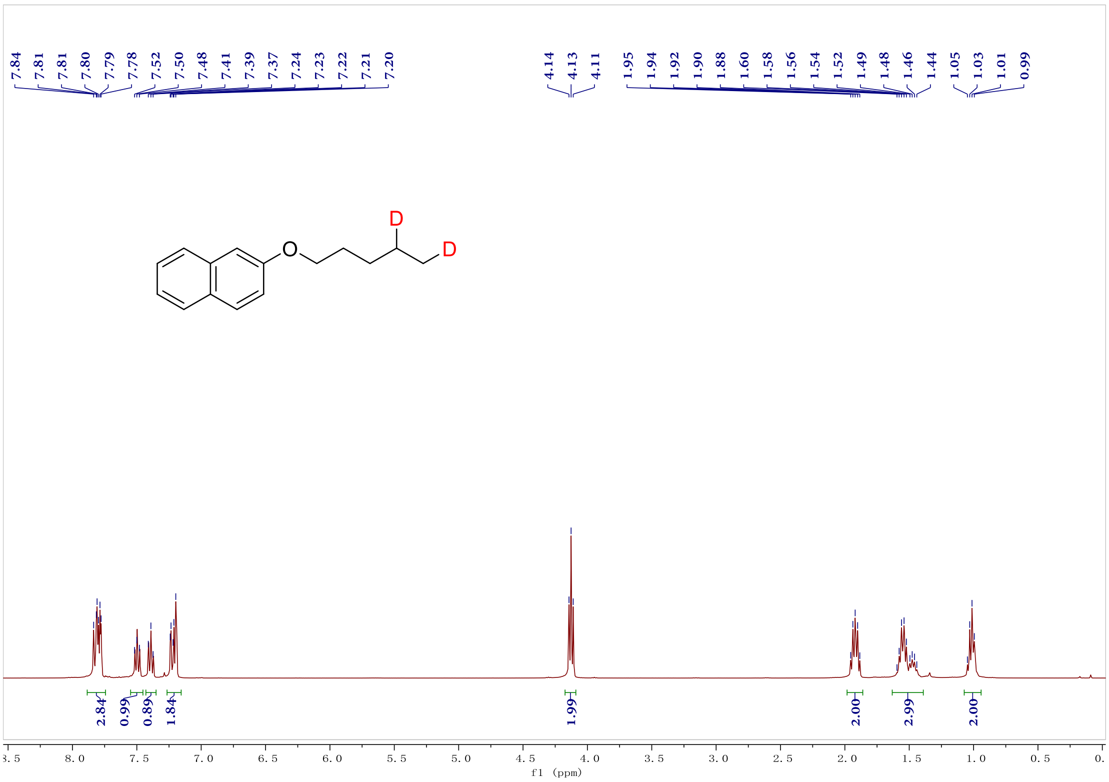

^{13}C NMR spectrum of **61** (**$CDCl_3$**)

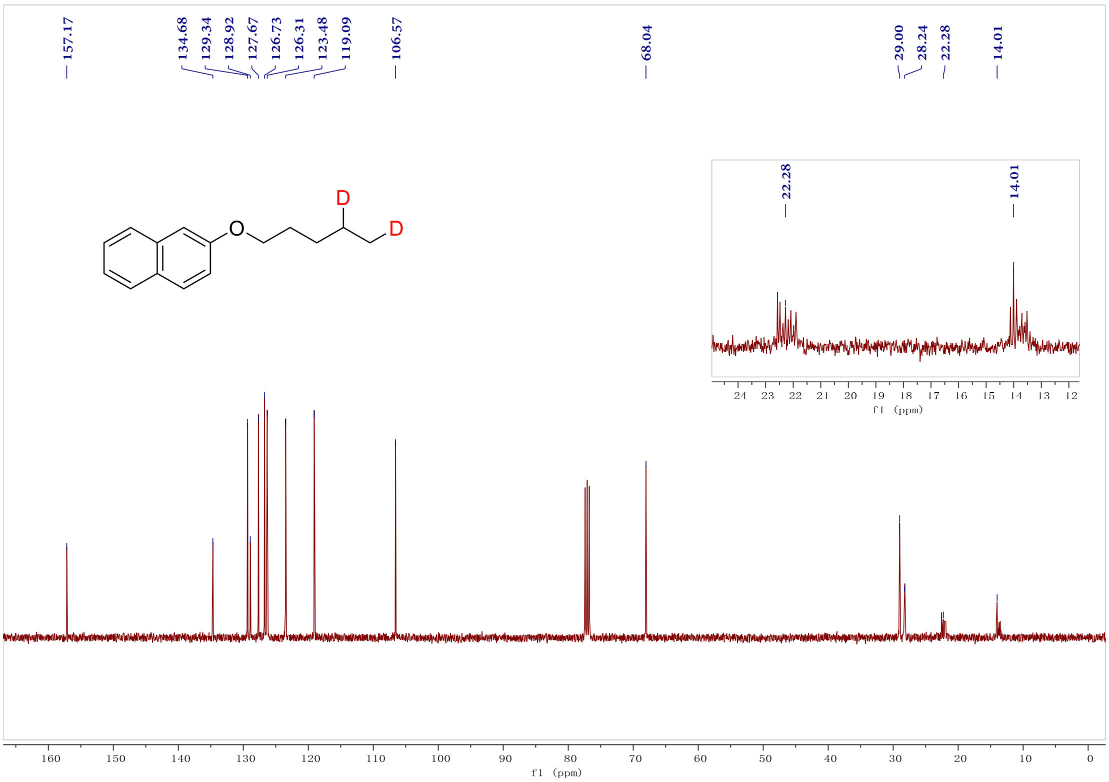

124

^{1}H NMR spectrum of **62** (**$CDCl_3$**)

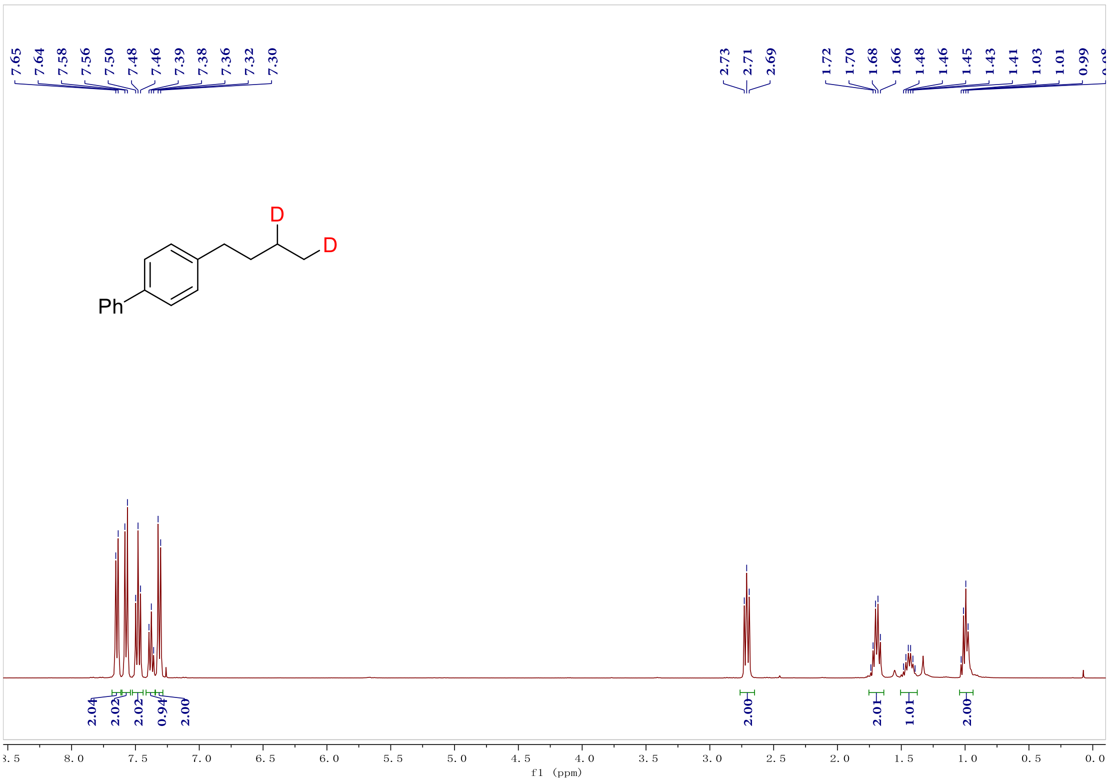

^{13}C NMR spectrum of **62** (**$CDCl_3$**)

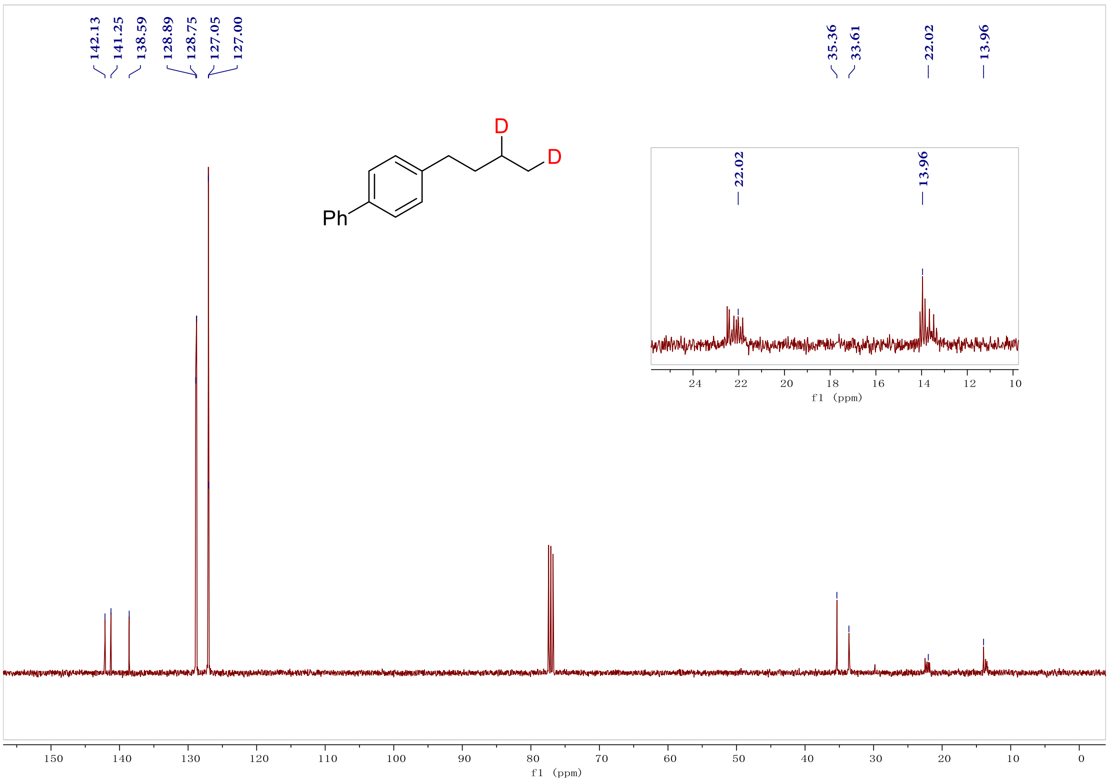

125

^{1}H NMR spectrum of **63** (**$CDCl_3$**)

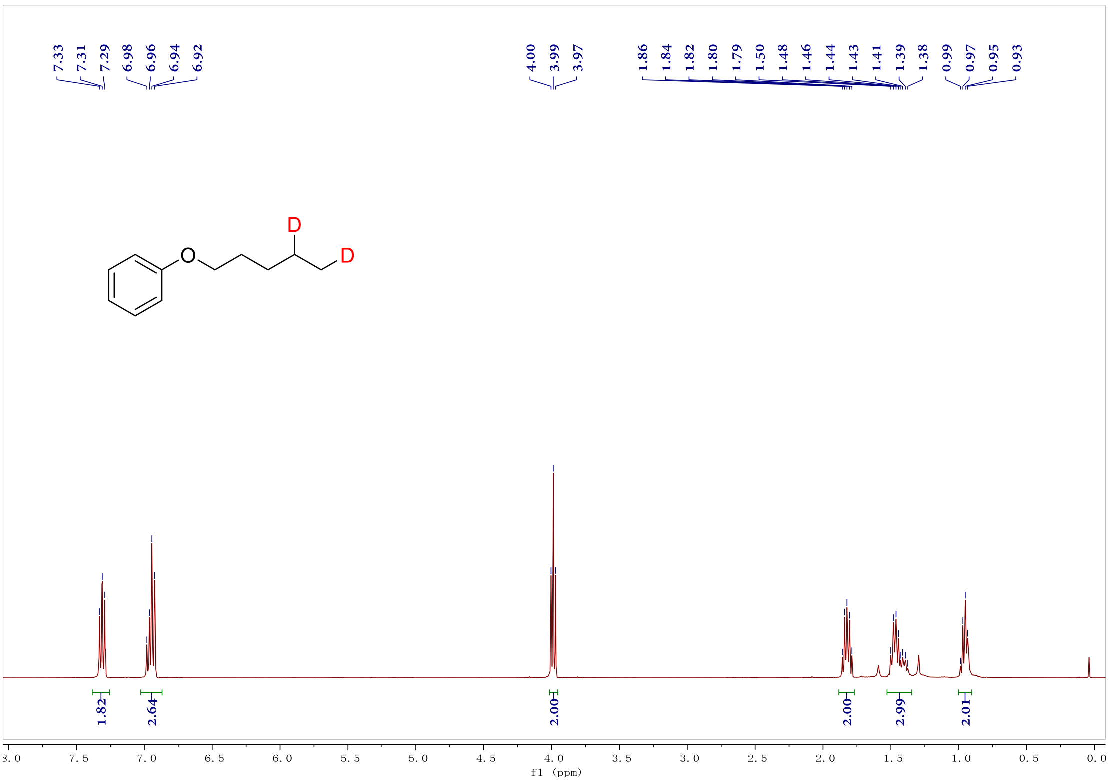

^{13}C NMR spectrum of **63** (**$CDCl_3$**)

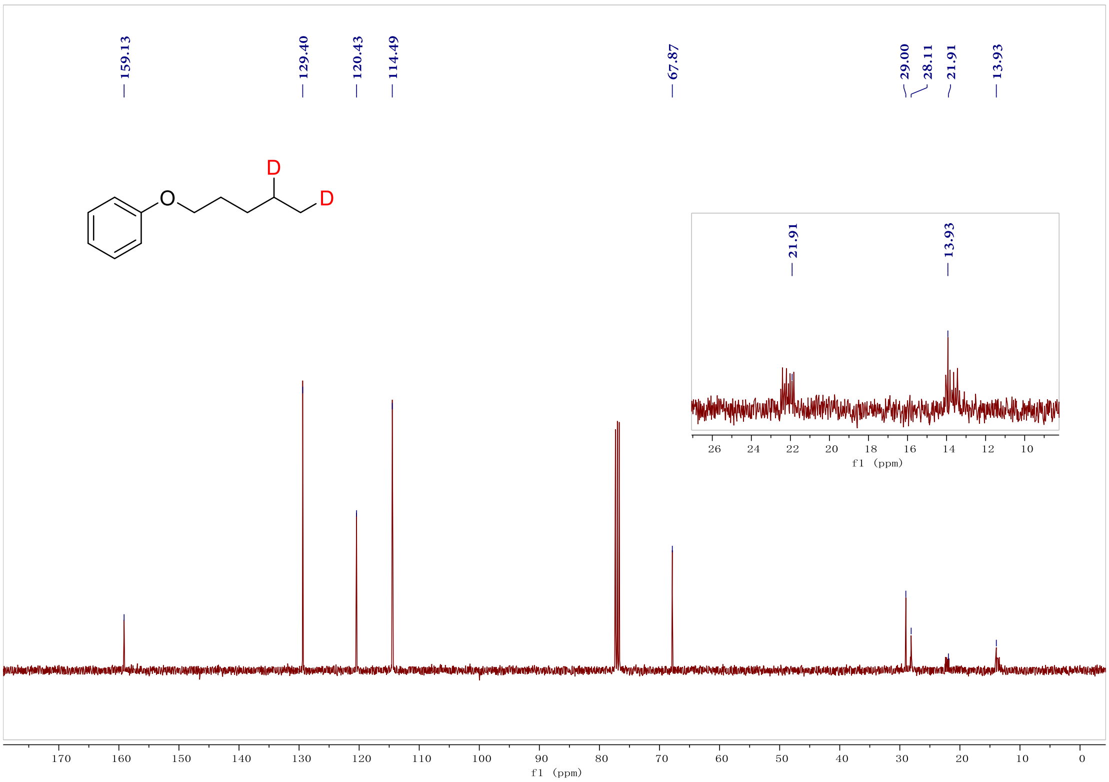

126

^{1}H NMR spectrum of **64** (**CDCl$_3$**)

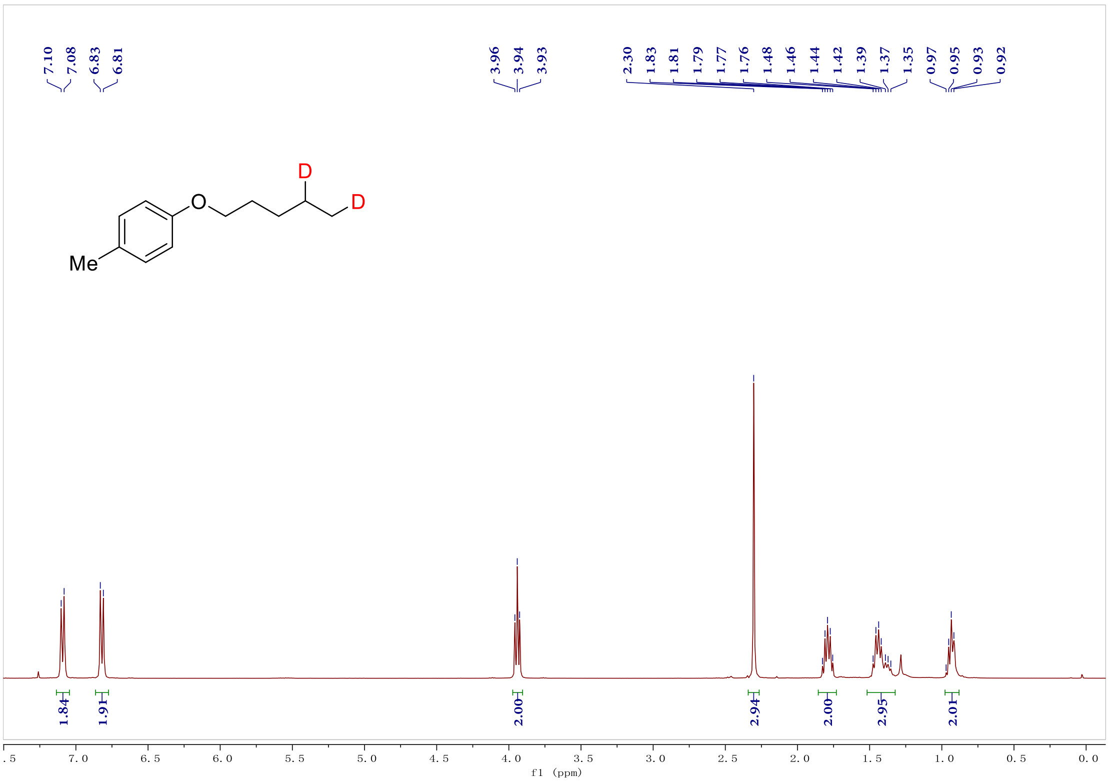

^{13}C NMR spectrum of **64** (**CDCl$_3$**)

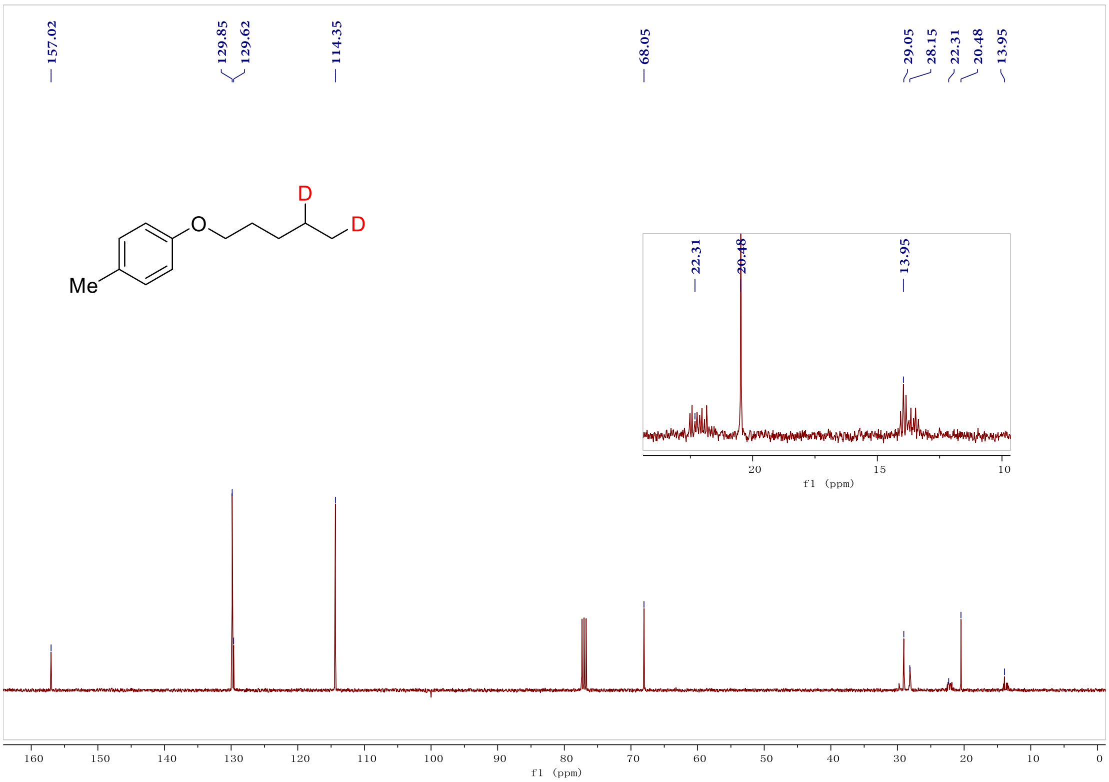

127

^{1}H NMR spectrum of **65** (**CDCl$_3$**)

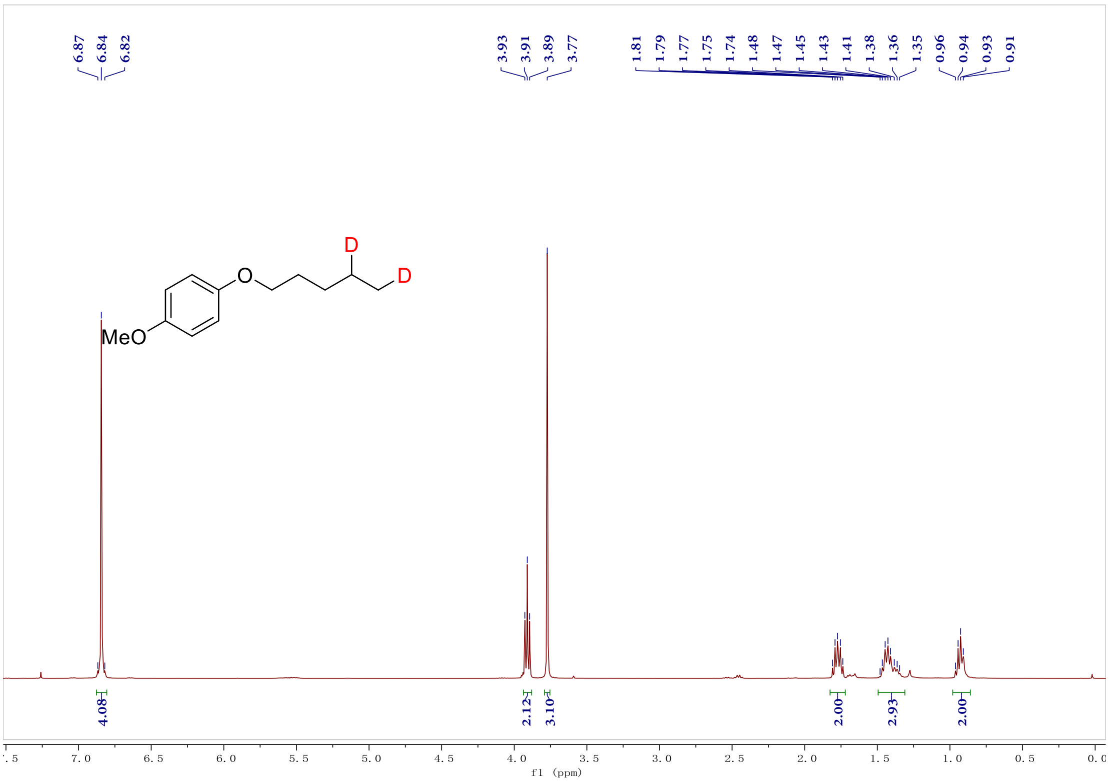

^{13}C NMR spectrum of **65** (**CDCl$_3$**)

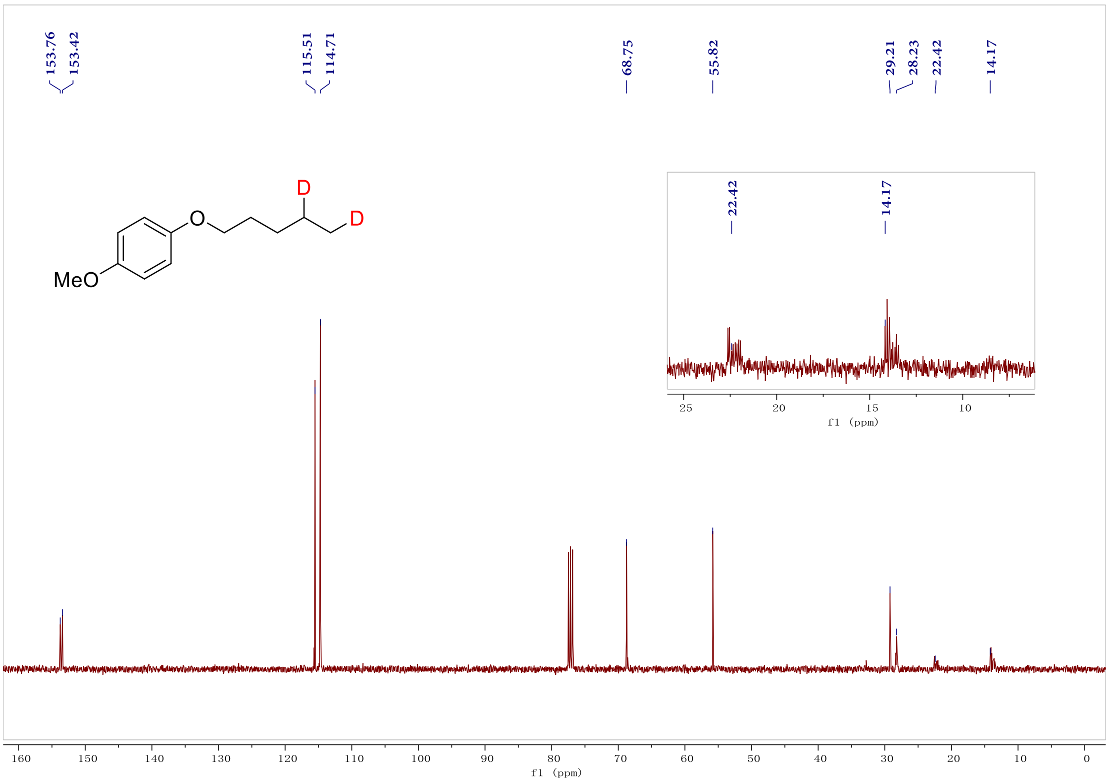

128

^{1}H NMR spectrum of **66** (**CDCl$_3$**)

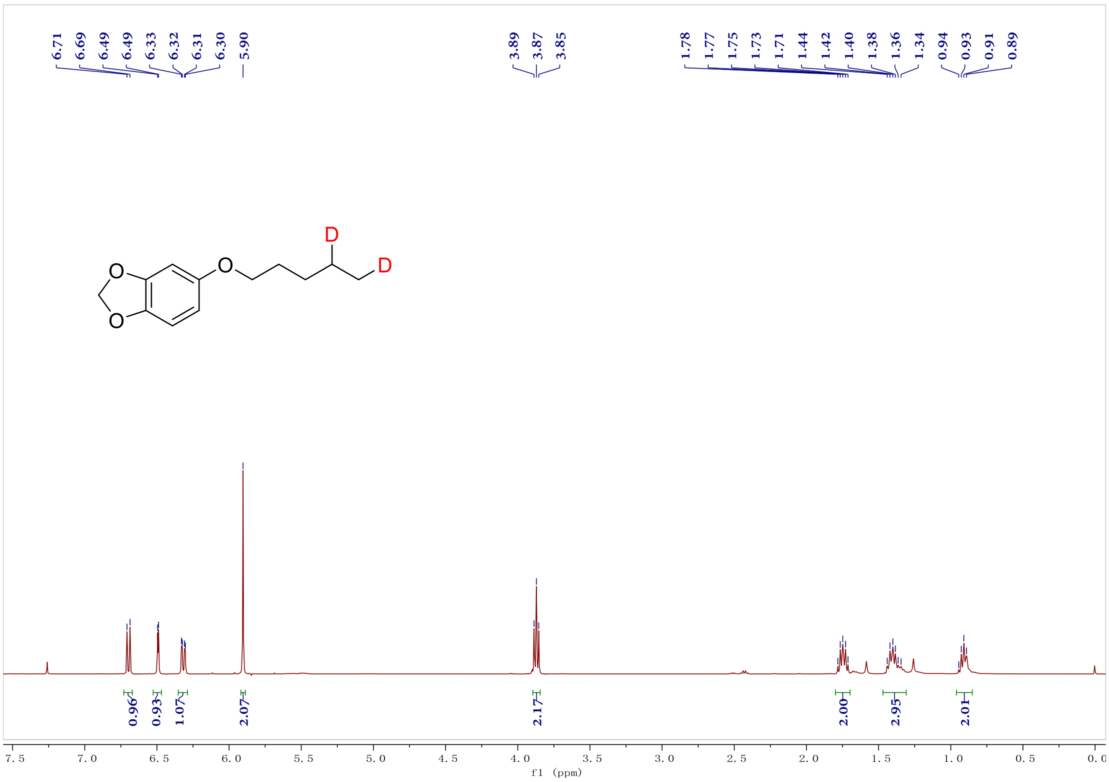

^{13}C NMR spectrum of **66** (**CDCl$_3$**)

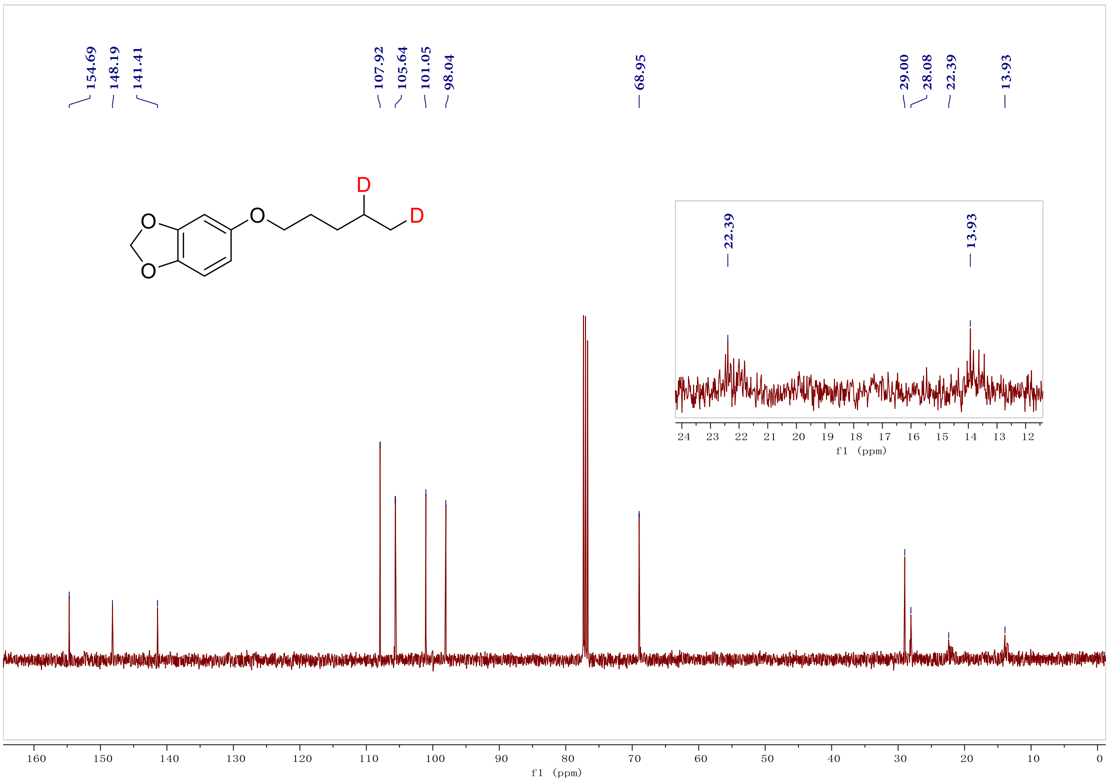

129

^{1}H NMR spectrum of **67** (**$CDCl_3$**)

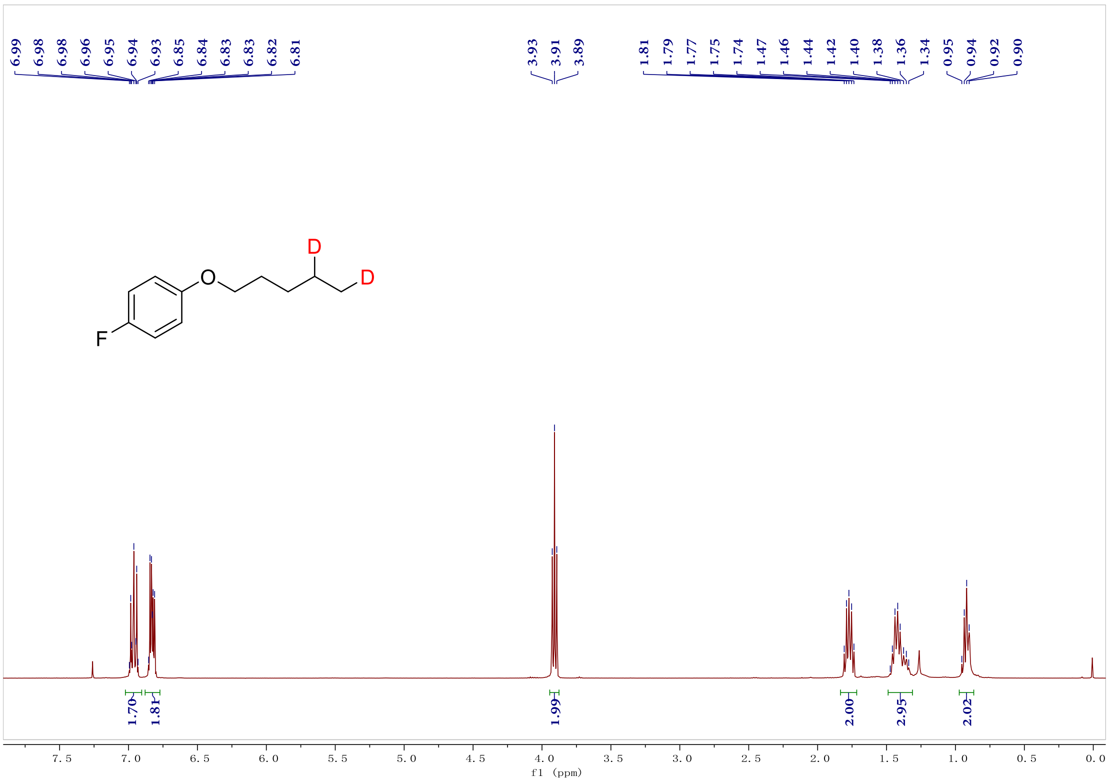

^{13}C NMR spectrum of **67** (**$CDCl_3$**)

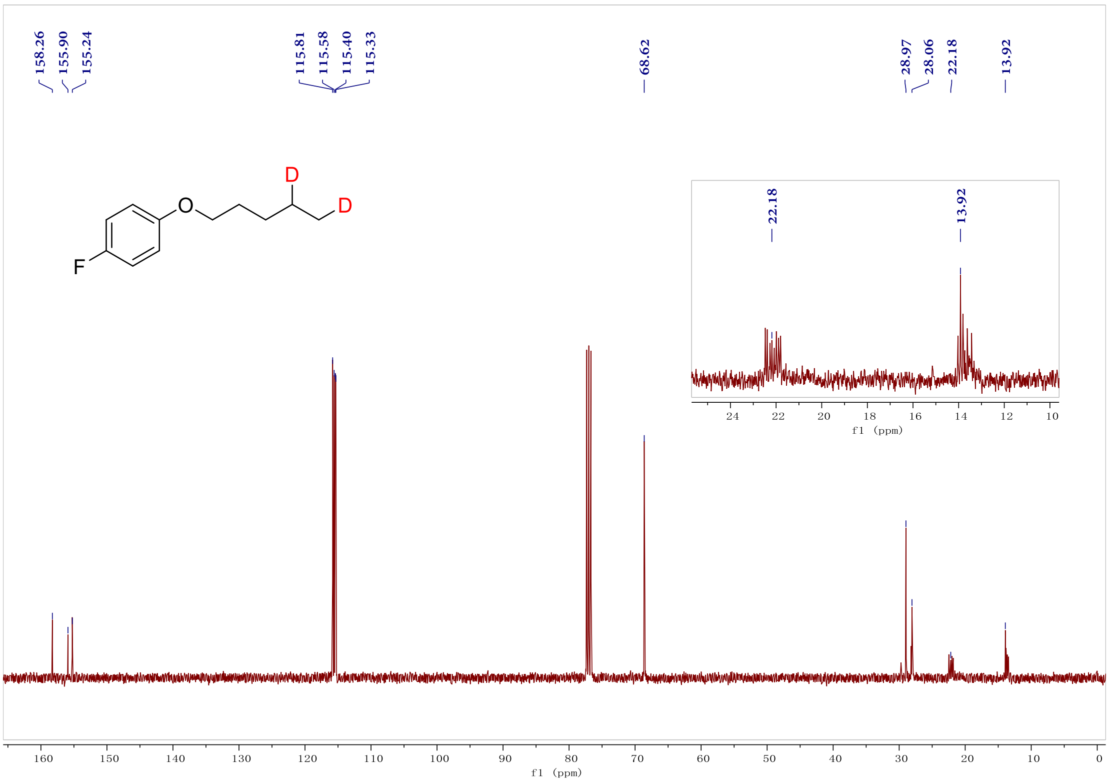

130

^{19}F NMR spectrum of **67** (**$CDCl_3$**)

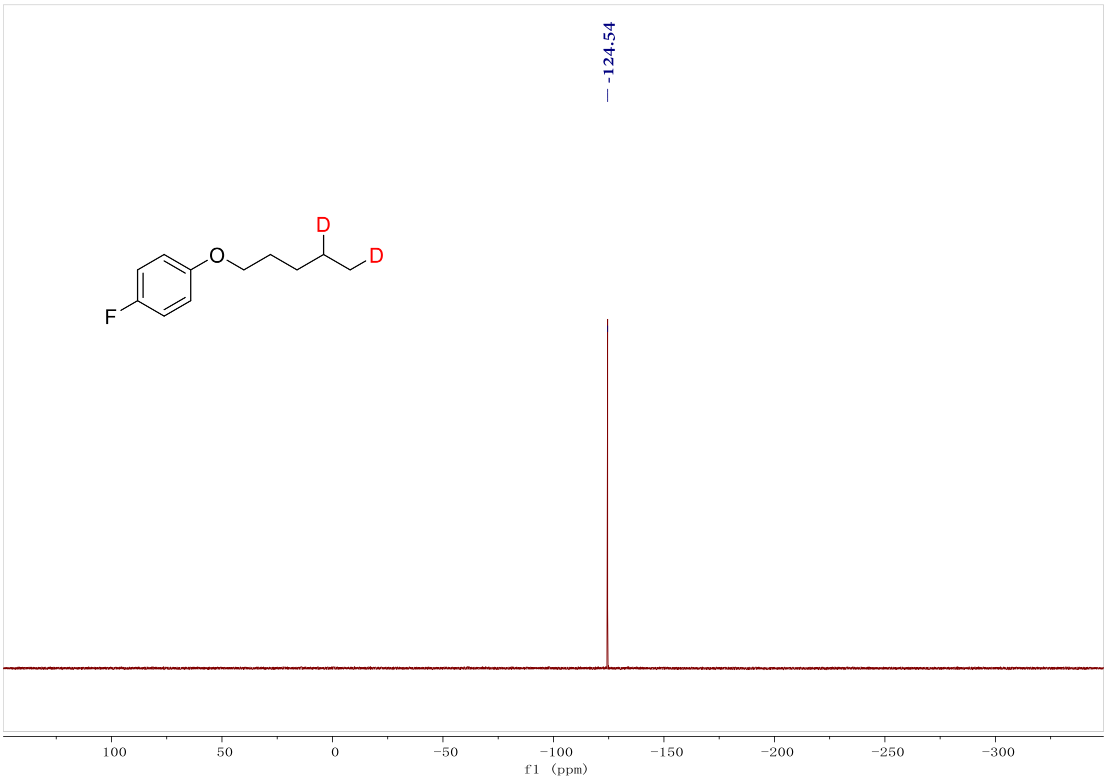

131

^{1}H NMR spectrum of **68** (**CDCl$_3$**)

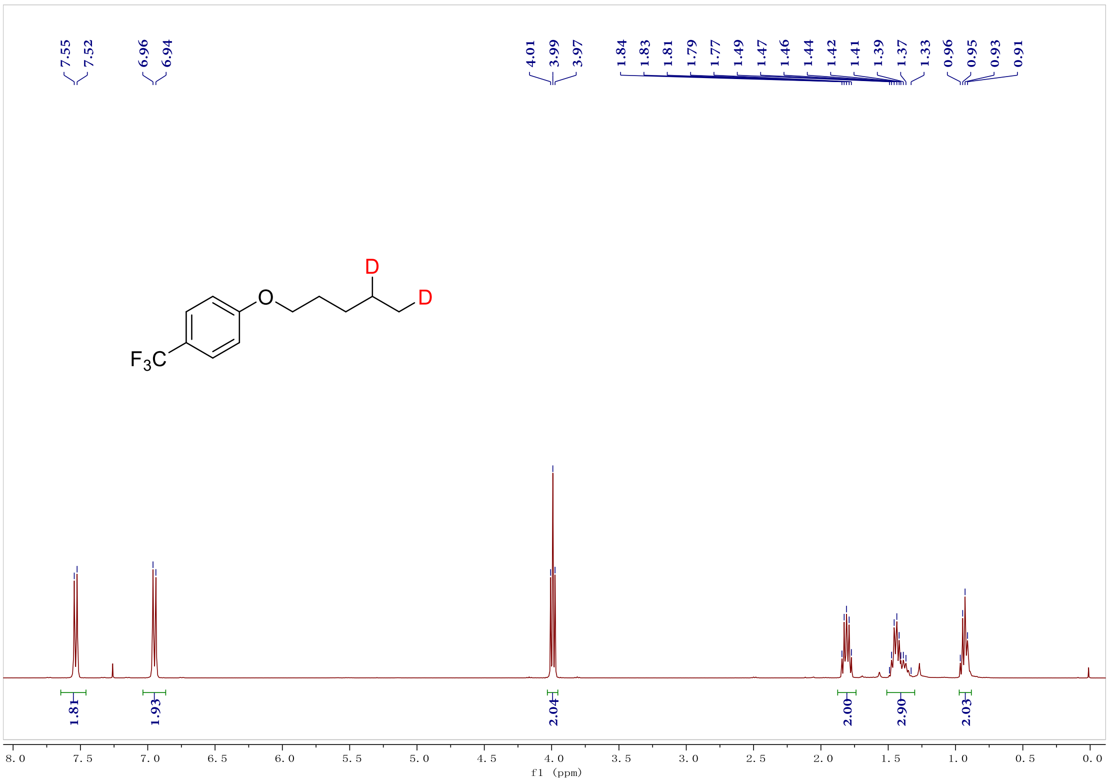

^{13}C NMR spectrum of **68** (**CDCl$_3$**)

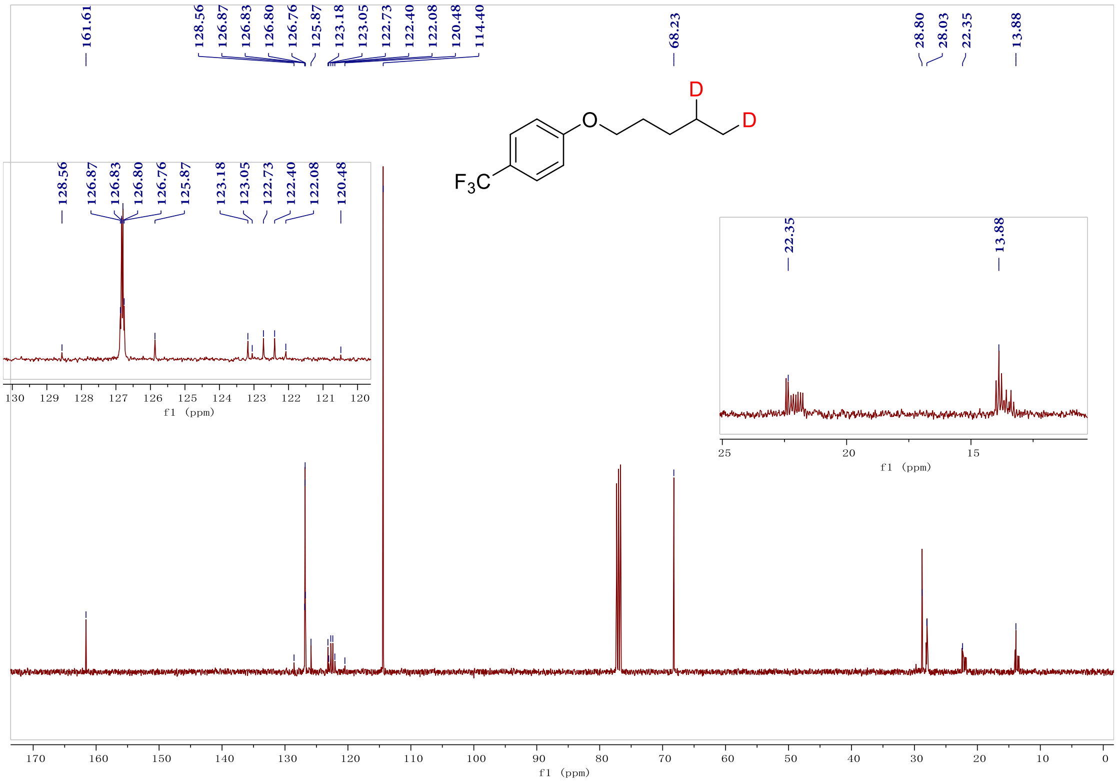

132

^{19}F NMR spectrum of **68** (**$CDCl_3$**)

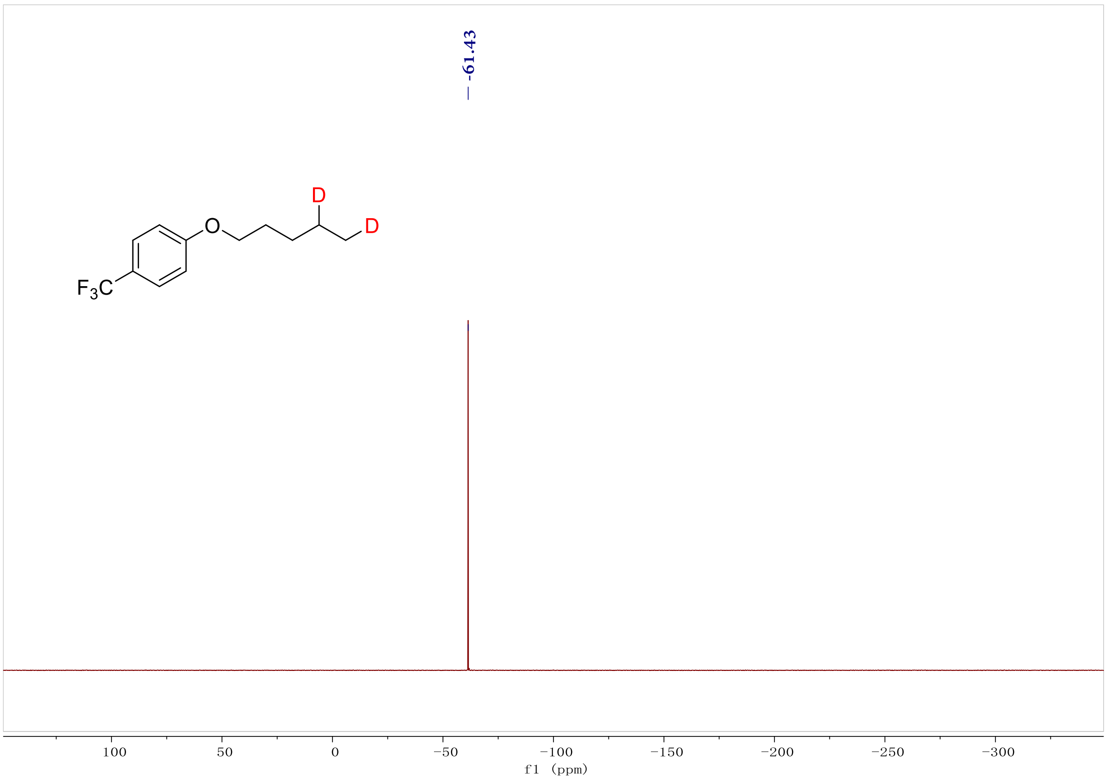

133

^{1}H NMR spectrum of **69** (**$CDCl_3$**)

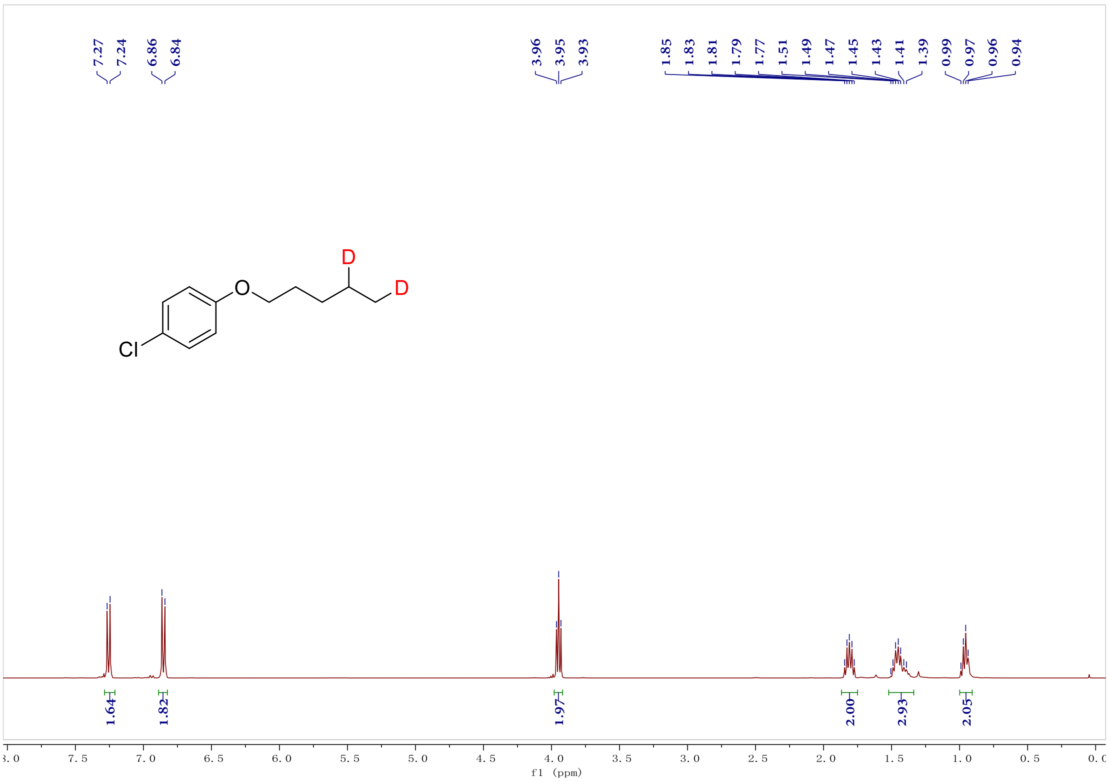

^{13}C NMR spectrum of **69** (**$CDCl_3$**)

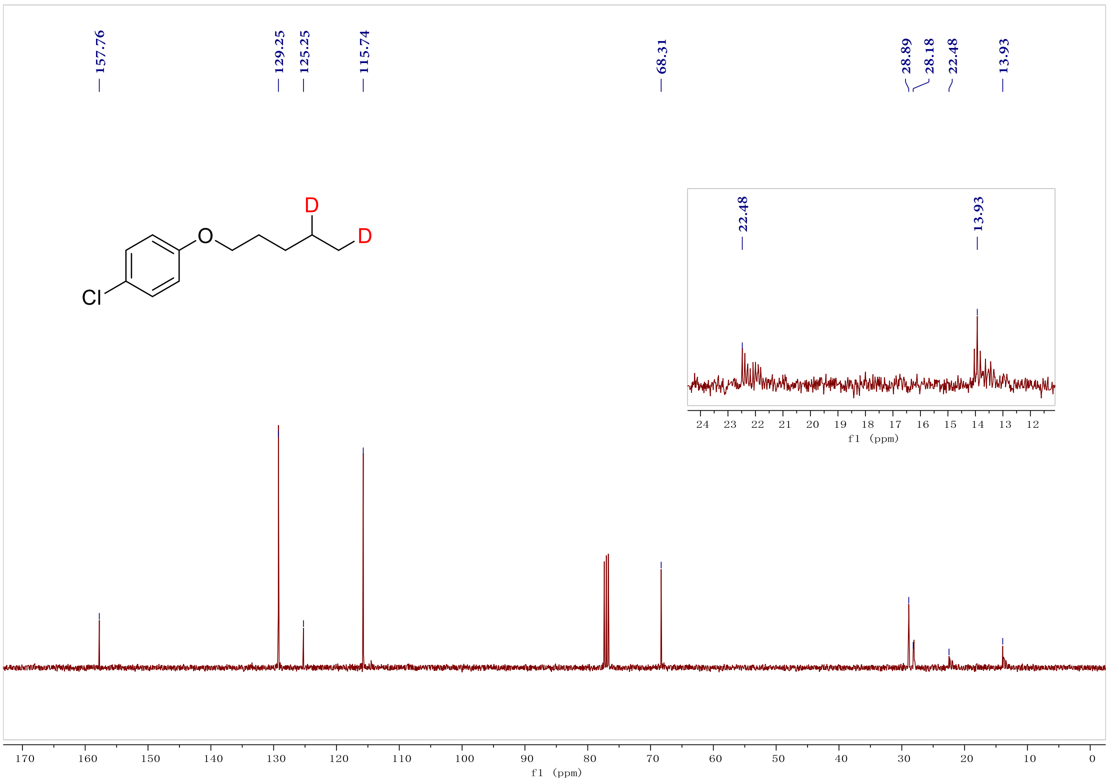

134

^{1}H NMR spectrum of **70** (**CDCl$_3$**)

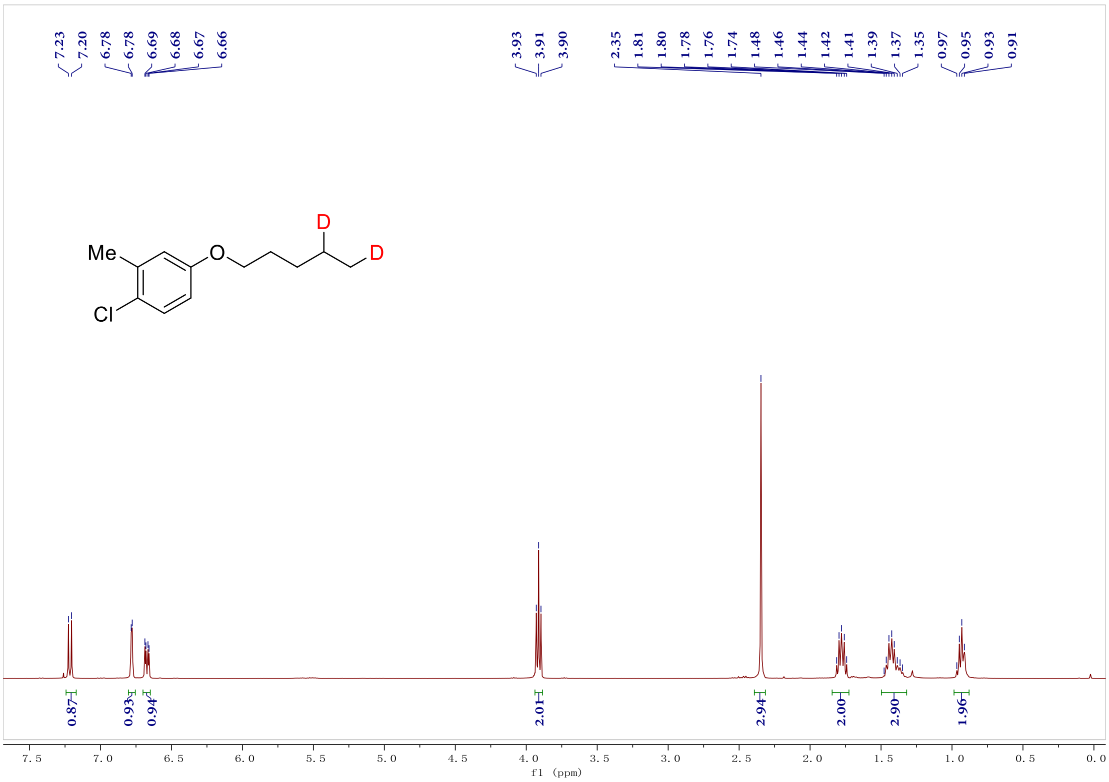

^{13}C NMR spectrum of **70** (**CDCl$_3$**)

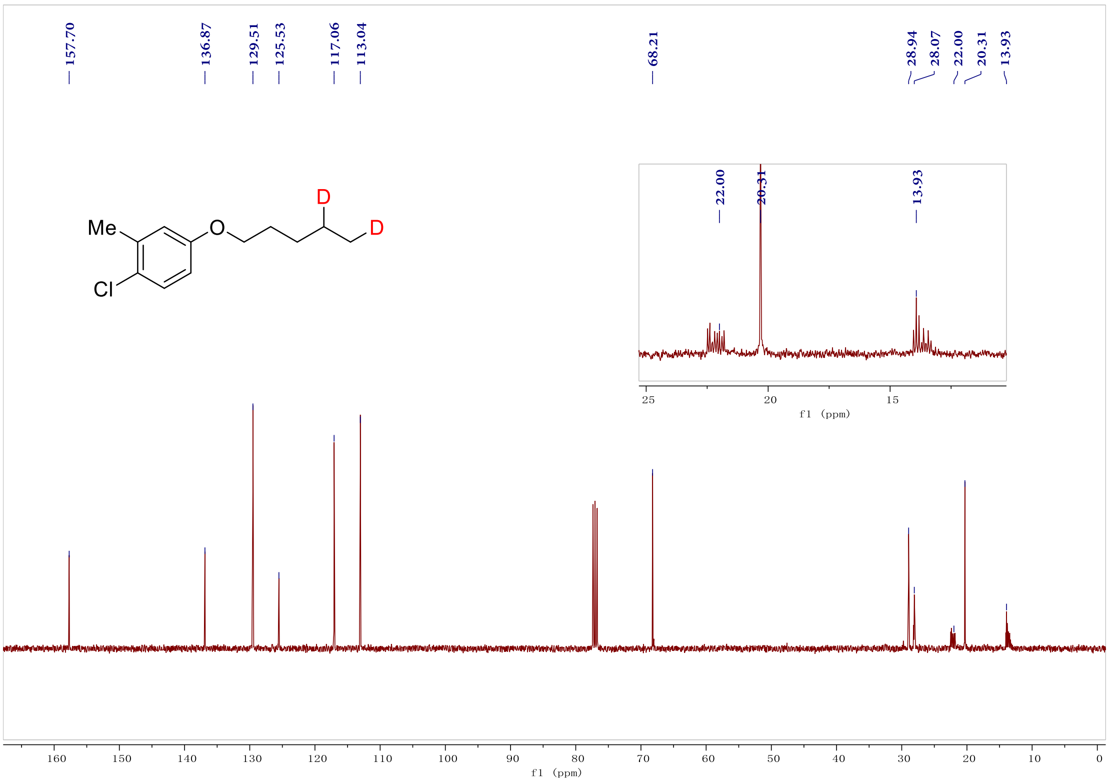

135

^{1}H NMR spectrum of **71** (**CDCl$_3$**)

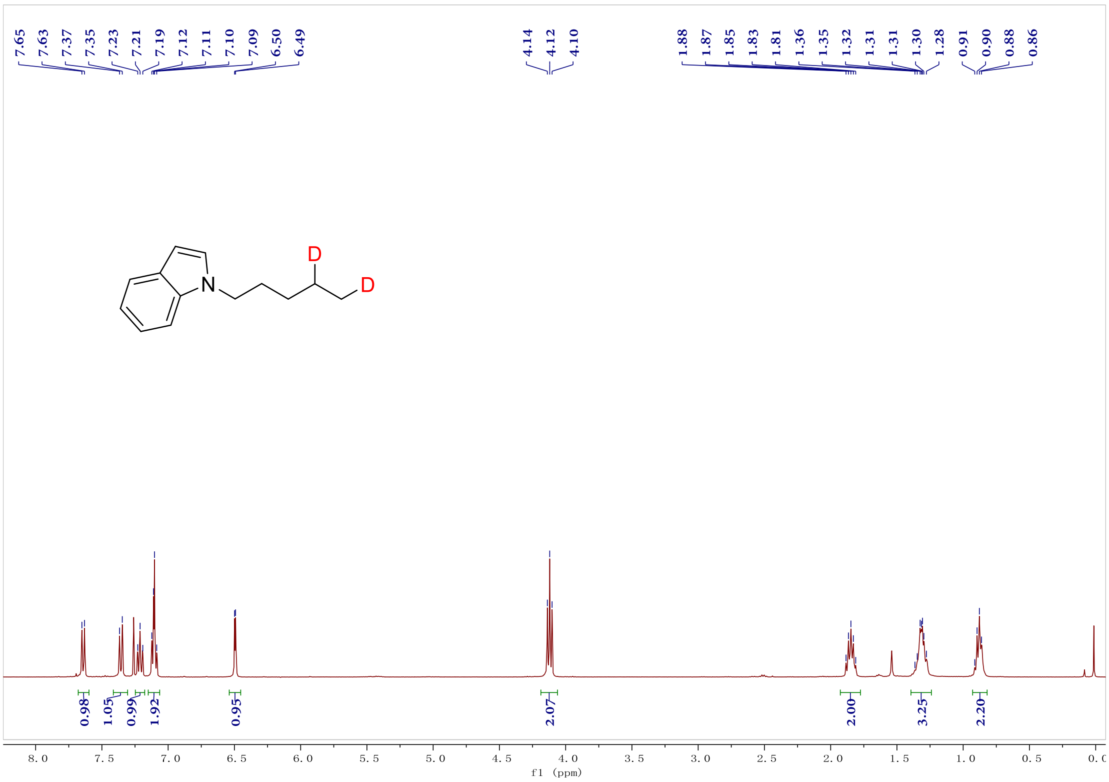

^{13}C NMR spectrum of **71** (**CDCl$_3$**)

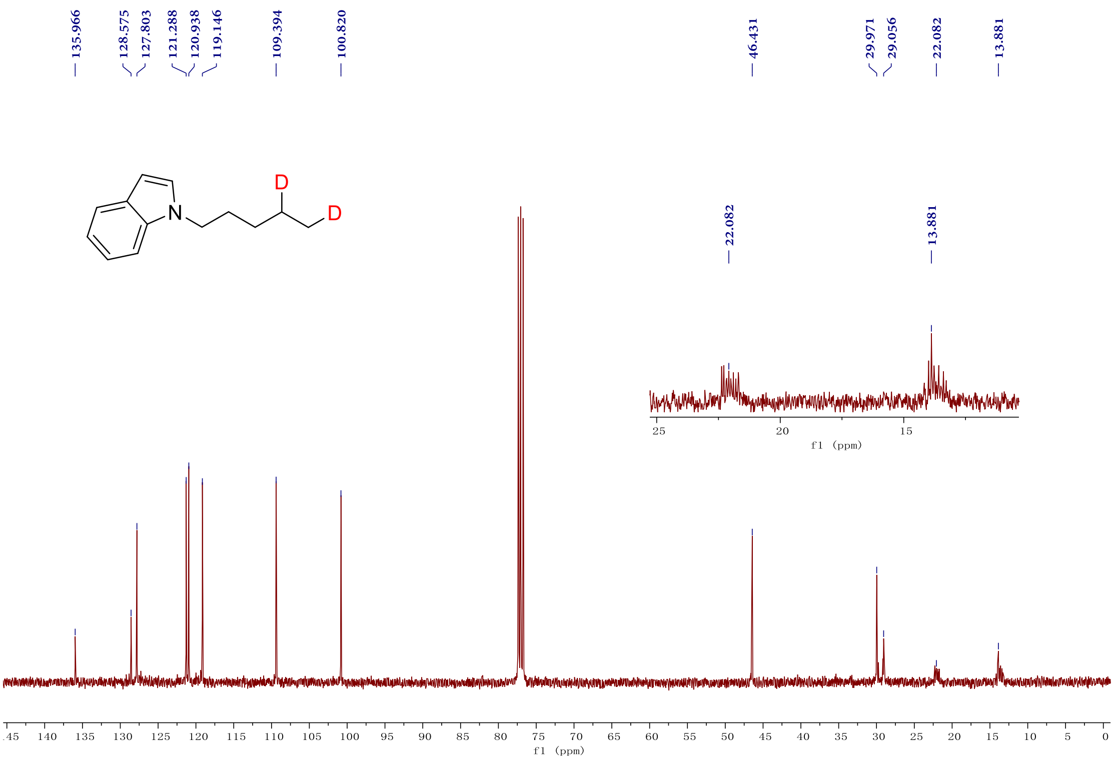

136

^{1}H NMR spectrum of **72** (**$CDCl_3$**)

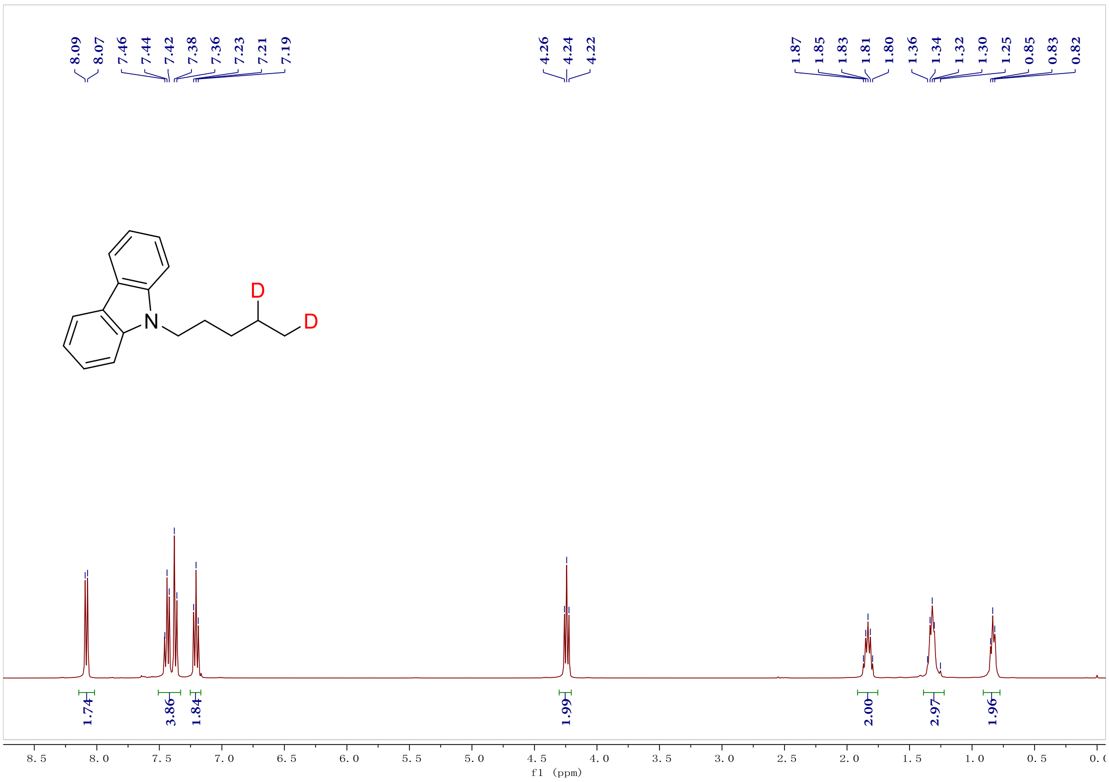

^{13}C NMR spectrum of **72** (**$CDCl_3$**)

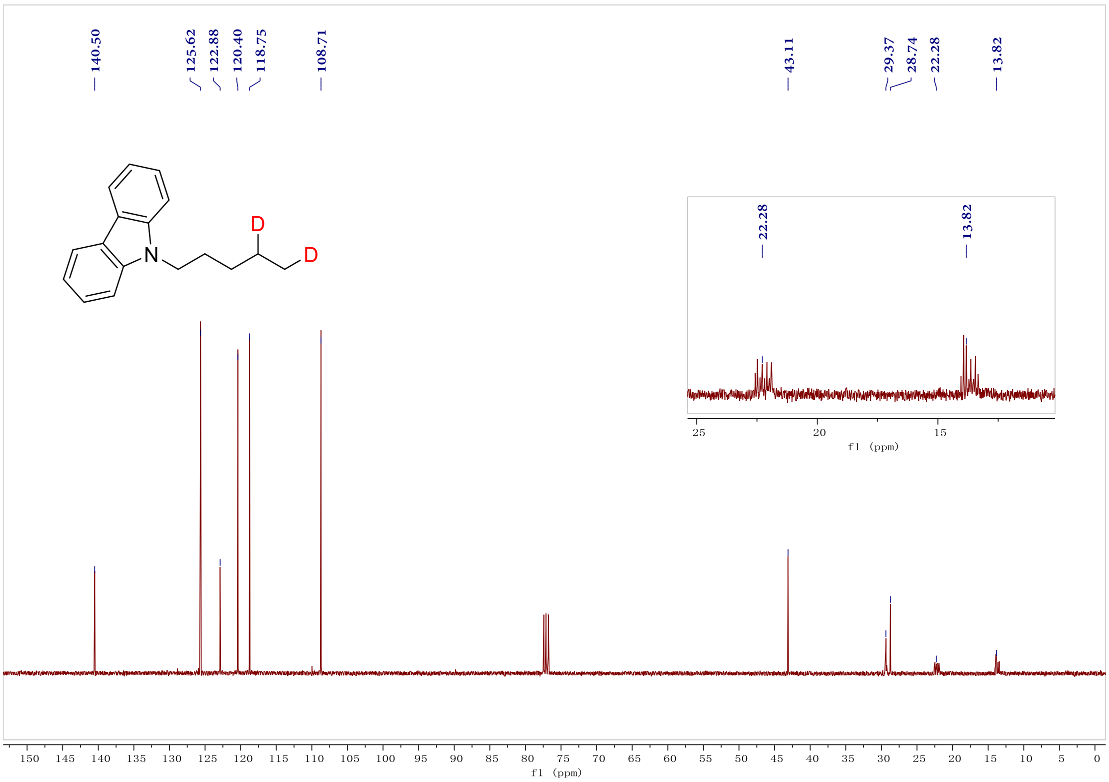

137

^{1}H NMR spectrum of **73** (**$CDCl_3$**)

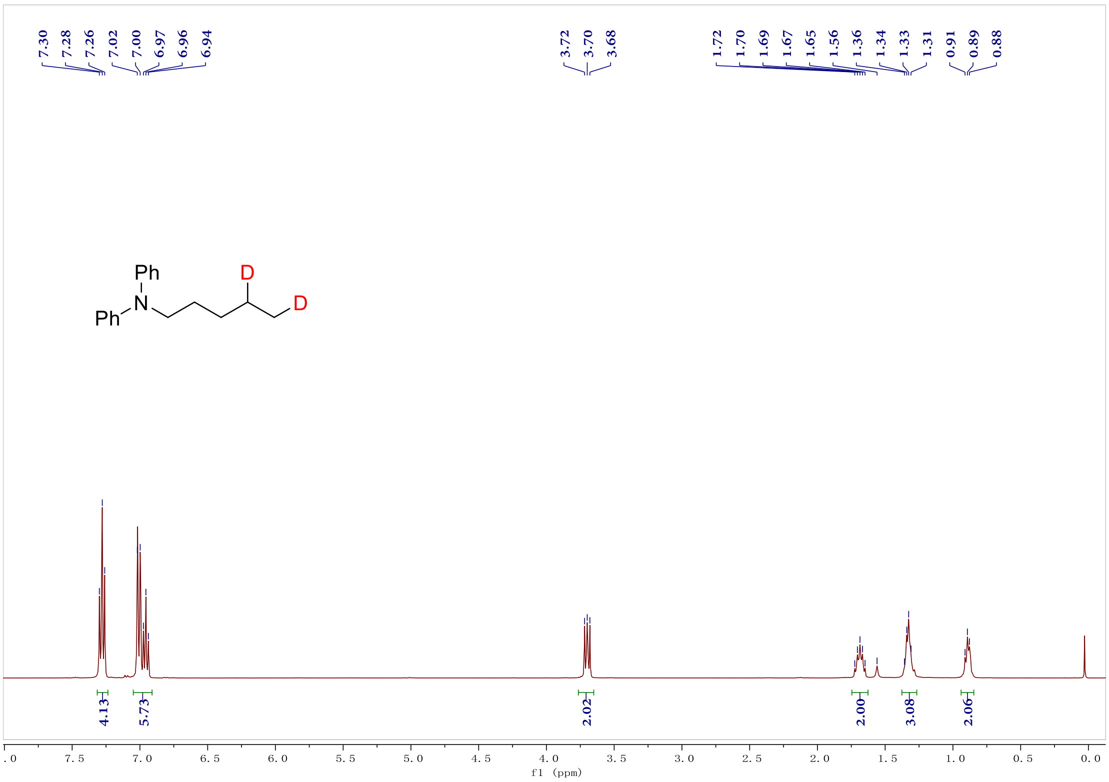

^{13}C NMR spectrum of **73** (**$CDCl_3$**)

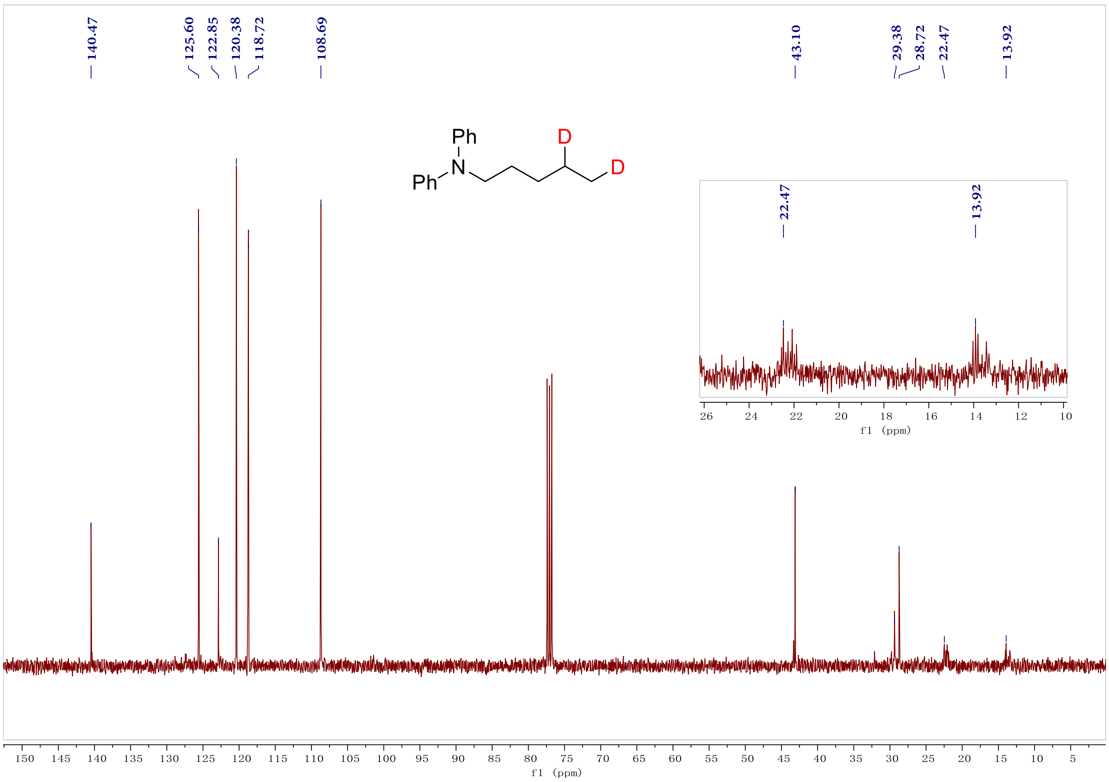

138

^1H NMR spectrum of **74** (**$CDCl_3$**)

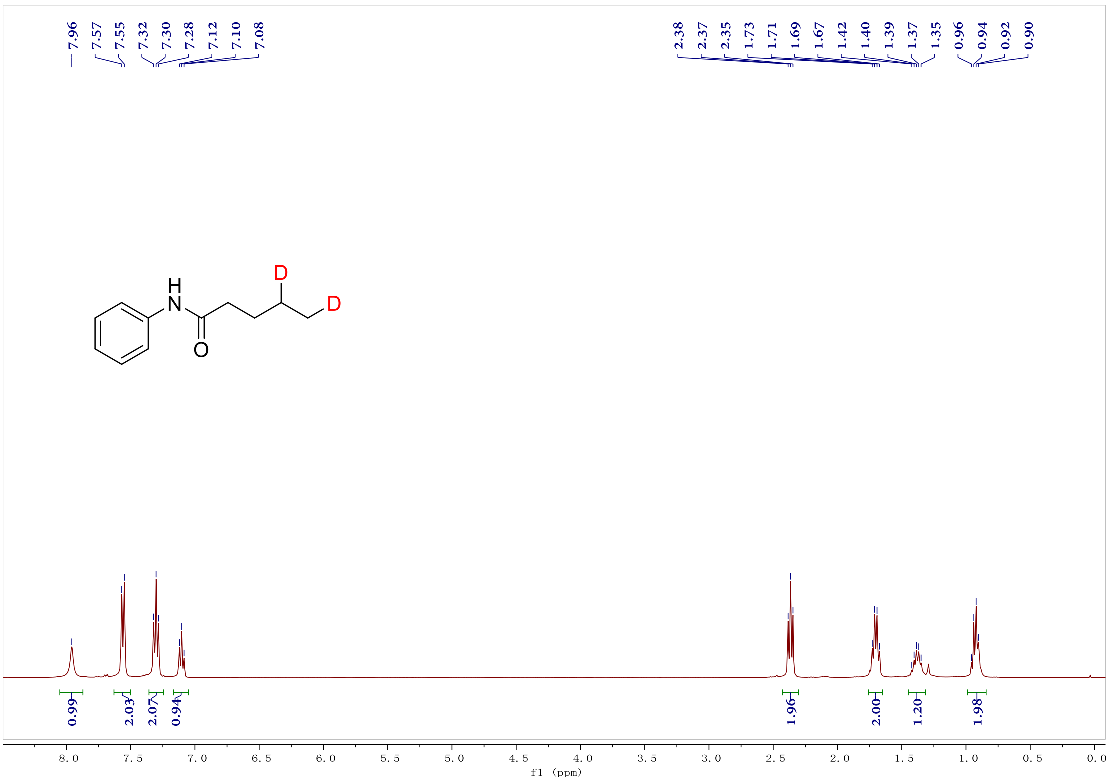

^{13}C NMR spectrum of **74** (**$CDCl_3$**)

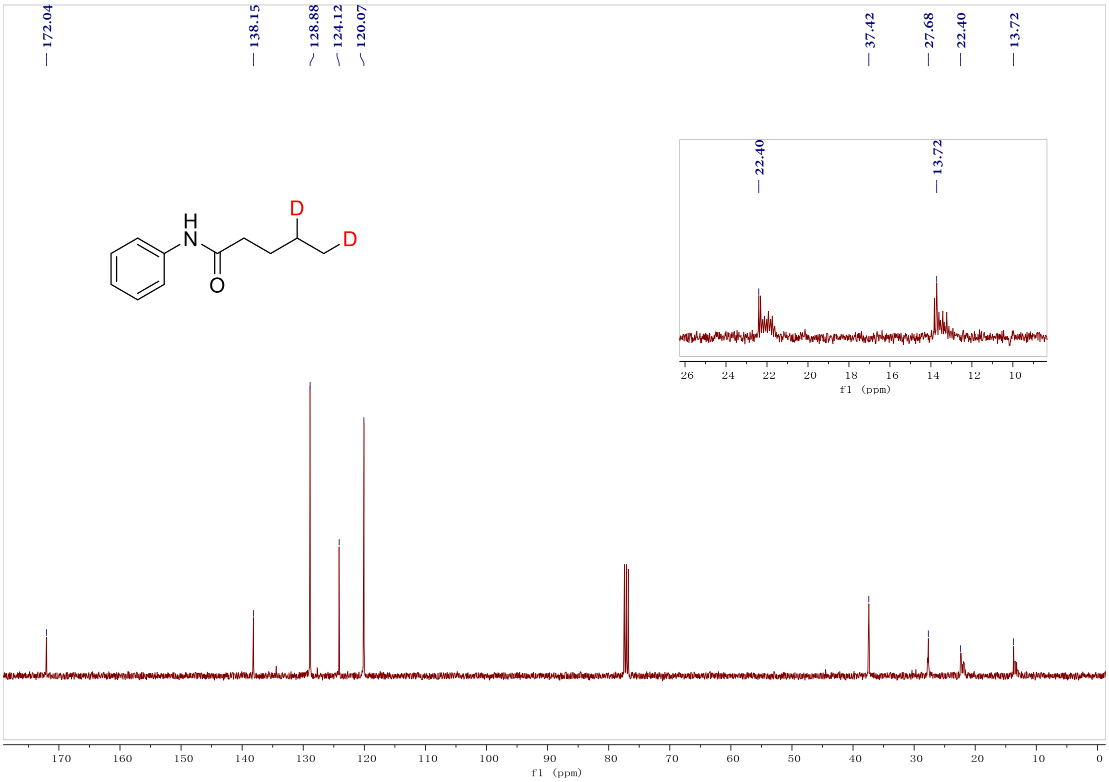

^{1}H NMR spectrum of **75** (**$CDCl_3$**)

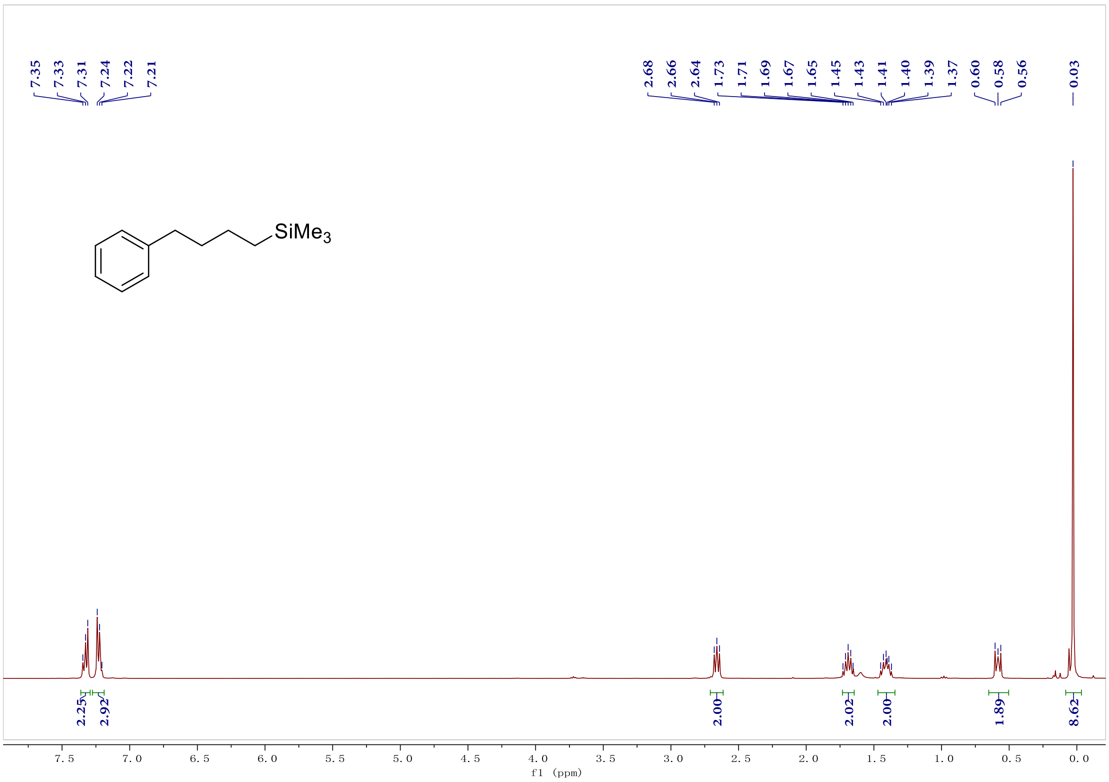

^{13}C NMR spectrum of **75** (**$CDCl_3$**)

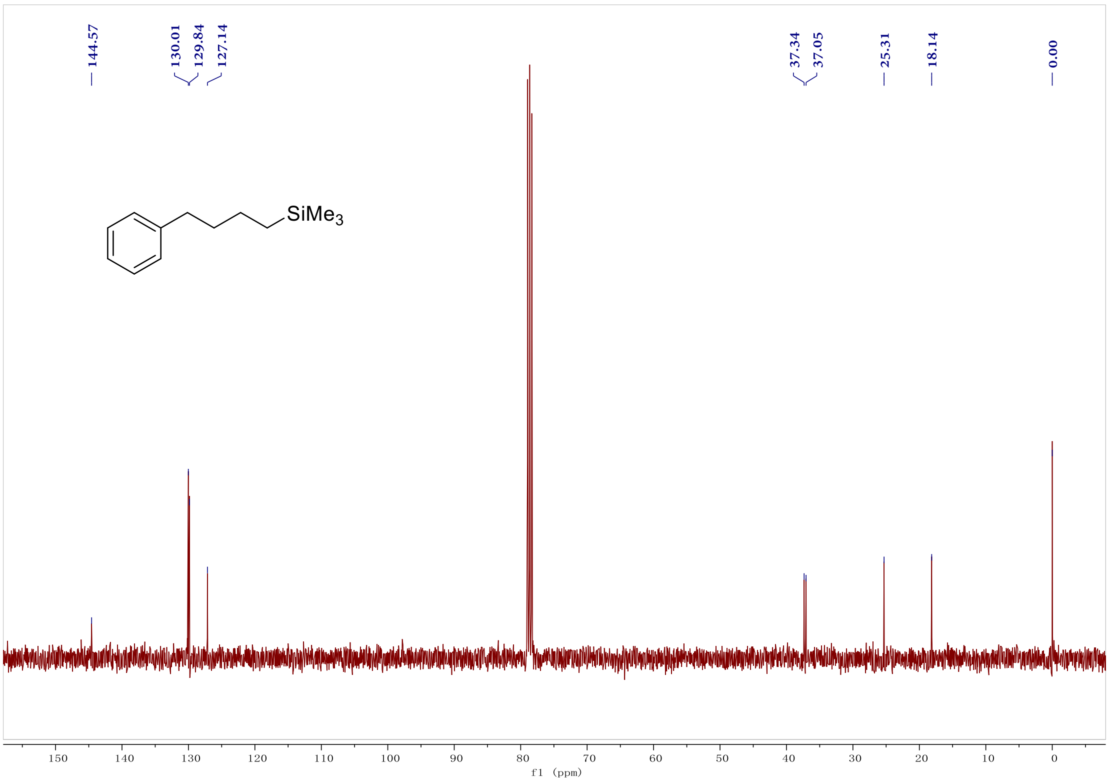

140

^{1}H NMR spectrum of **76** (**$CDCl_3$**)

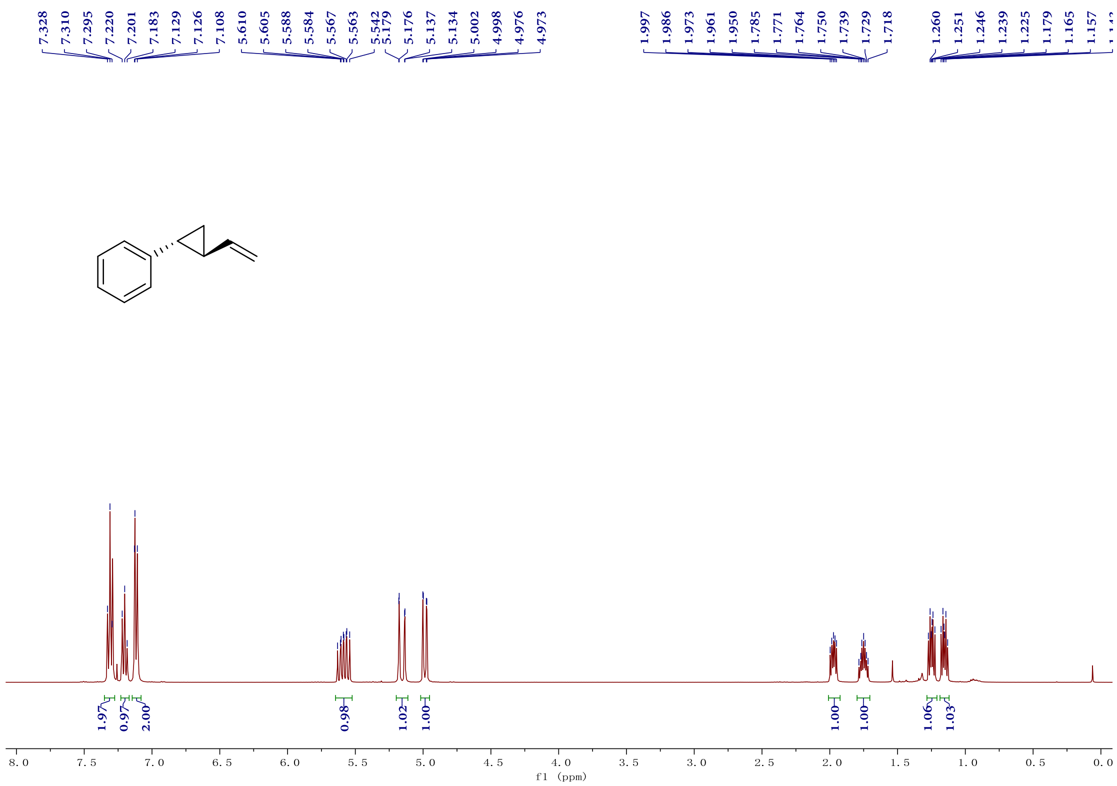

^{13}C NMR spectrum of **76** (**$CDCl_3$**)

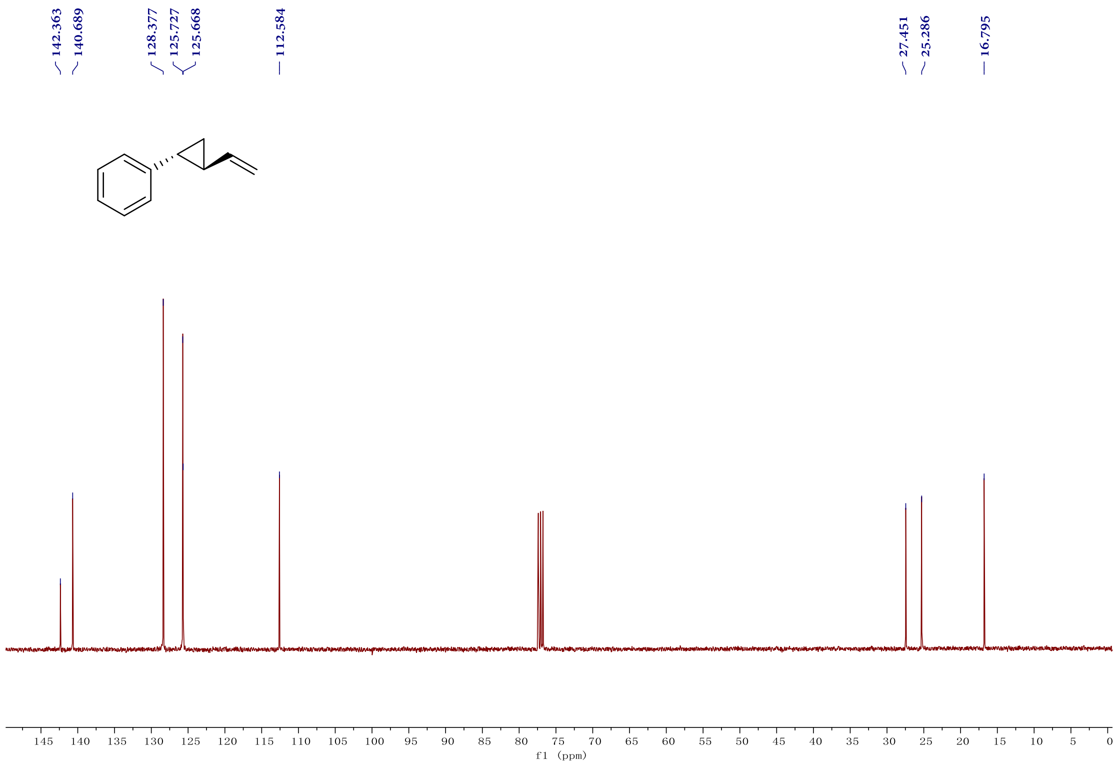

[1] H NMR spectrum of **77** (**$CDCl_3$**)

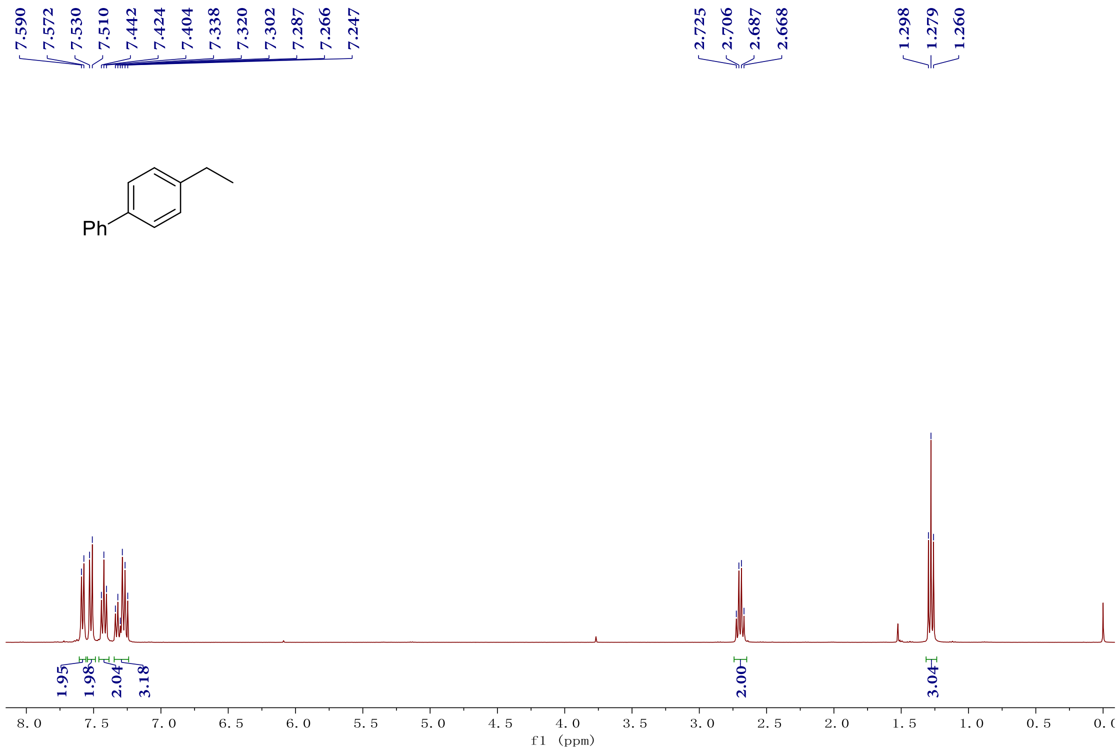

[13] C NMR spectrum of **77** (**$CDCl_3$**)

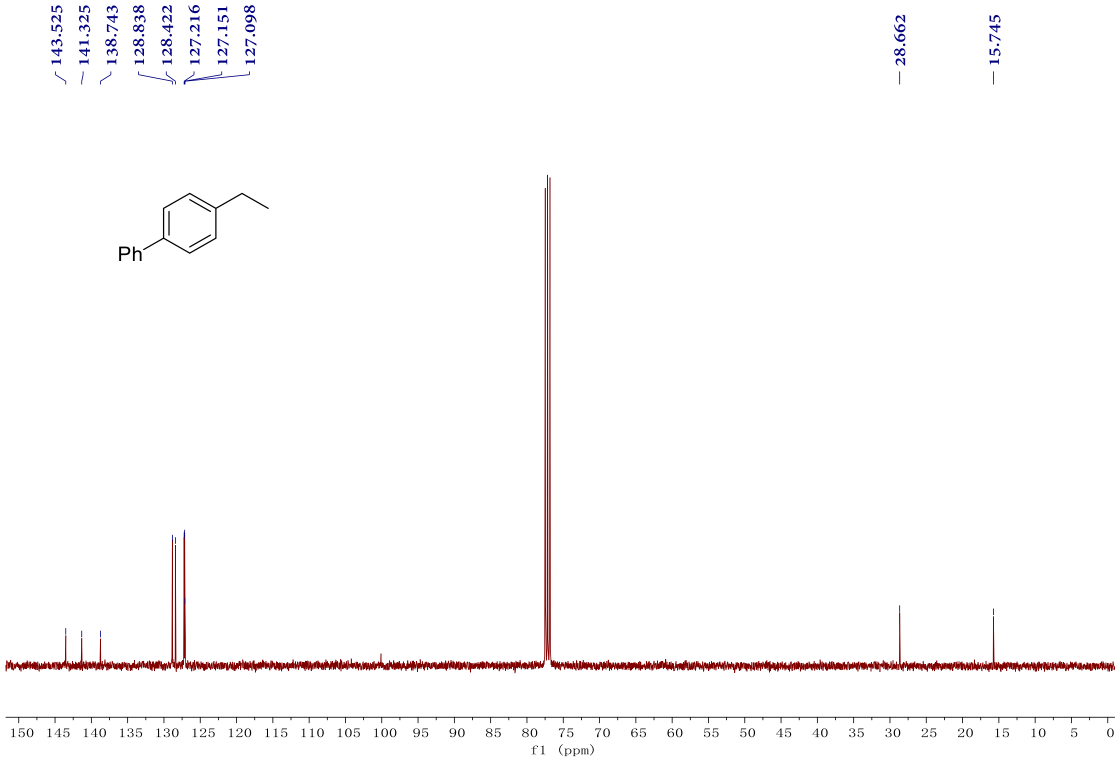

142

^{1}H NMR spectrum of **78** (**$CDCl_3$**)

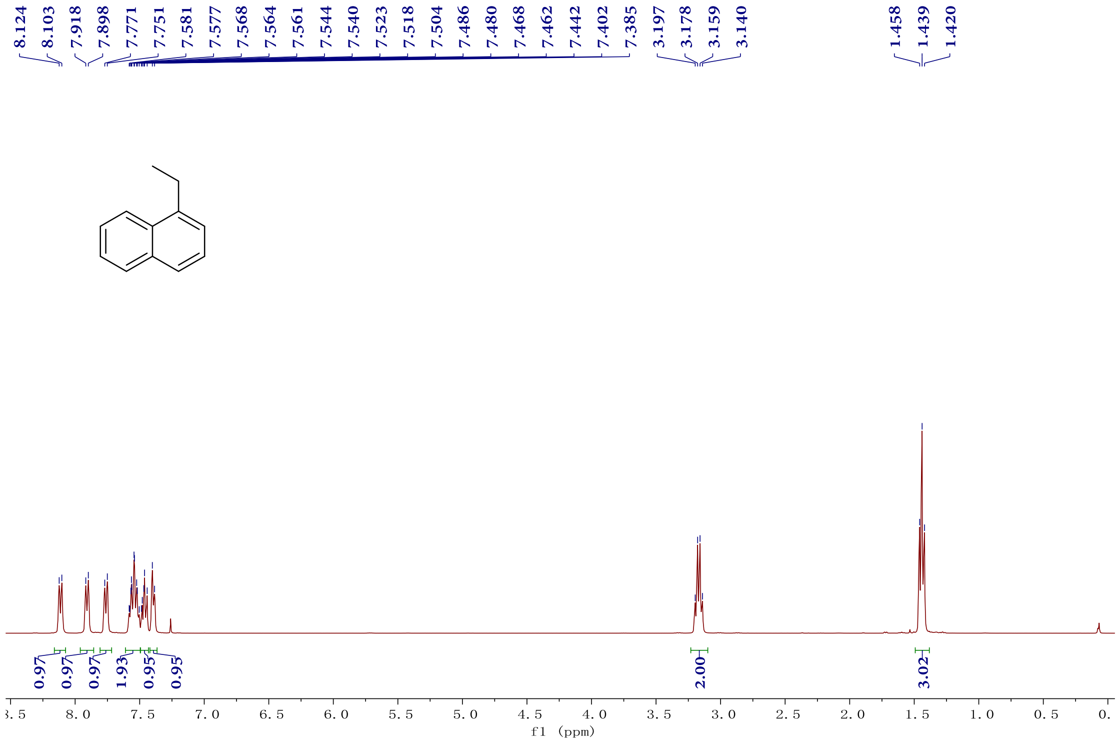

^{13}C NMR spectrum of **78** (**$CDCl_3$**)

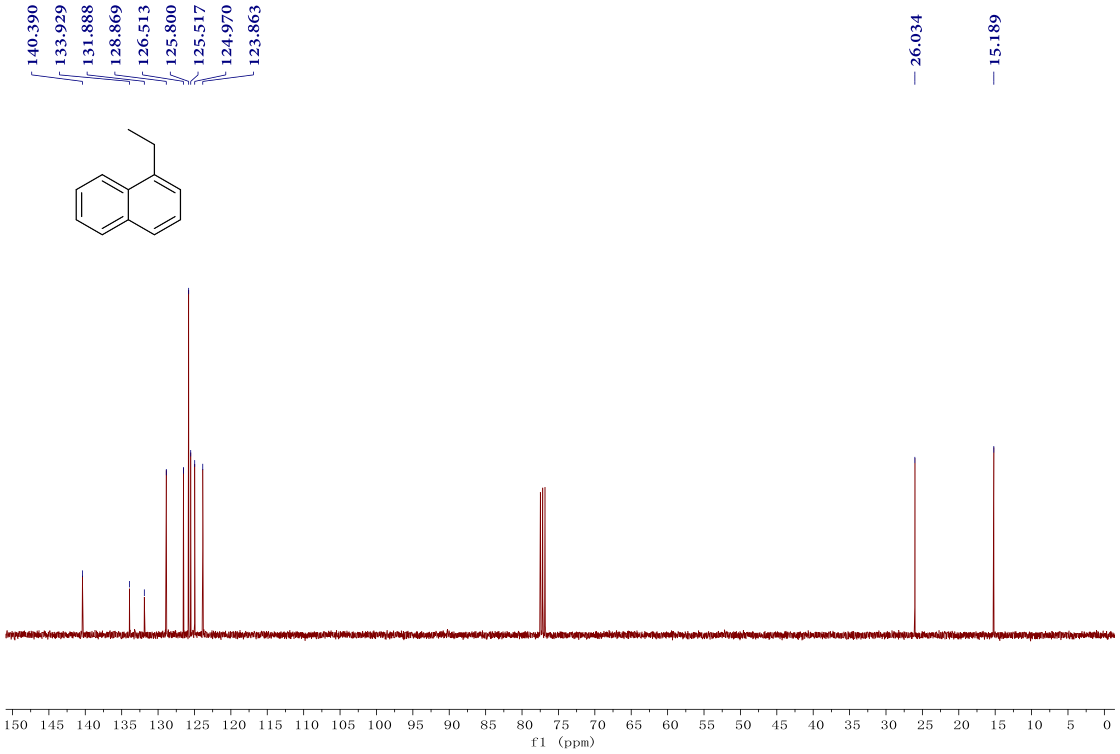

143

^{1}H NMR spectrum of **79** (**$CDCl_3$**)

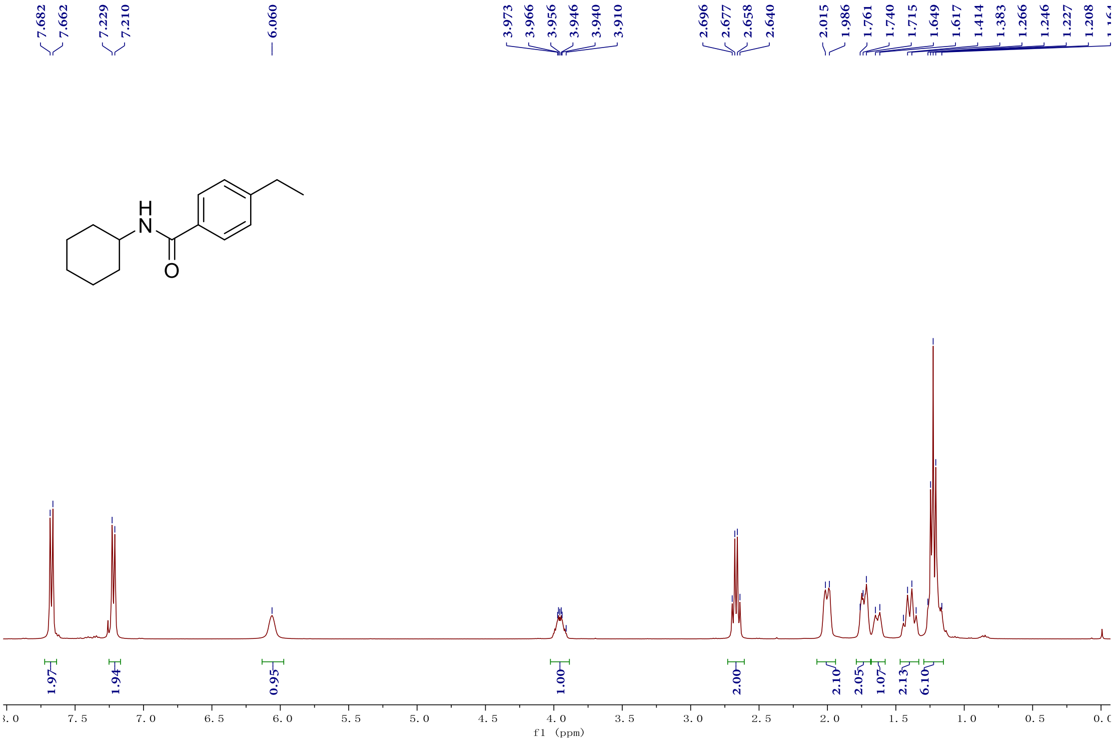

^{13}C NMR spectrum of **79** (**$CDCl_3$**)

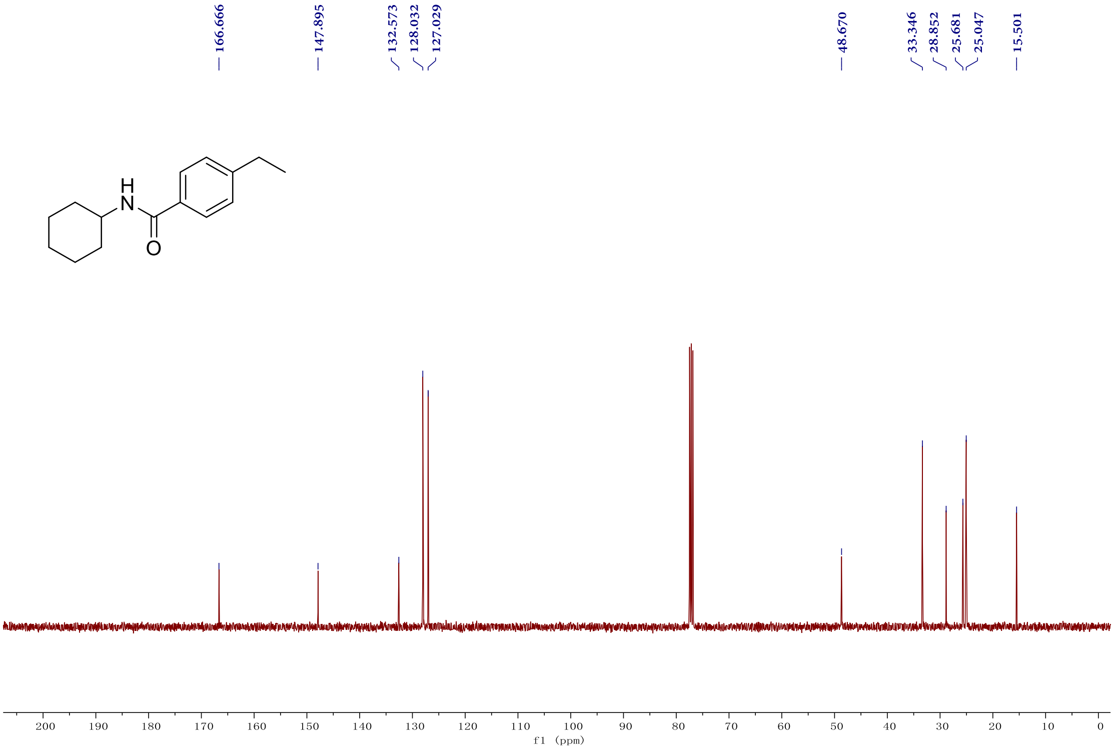

144

^{1}H NMR spectrum of **80** (**CDCl$_3$**)

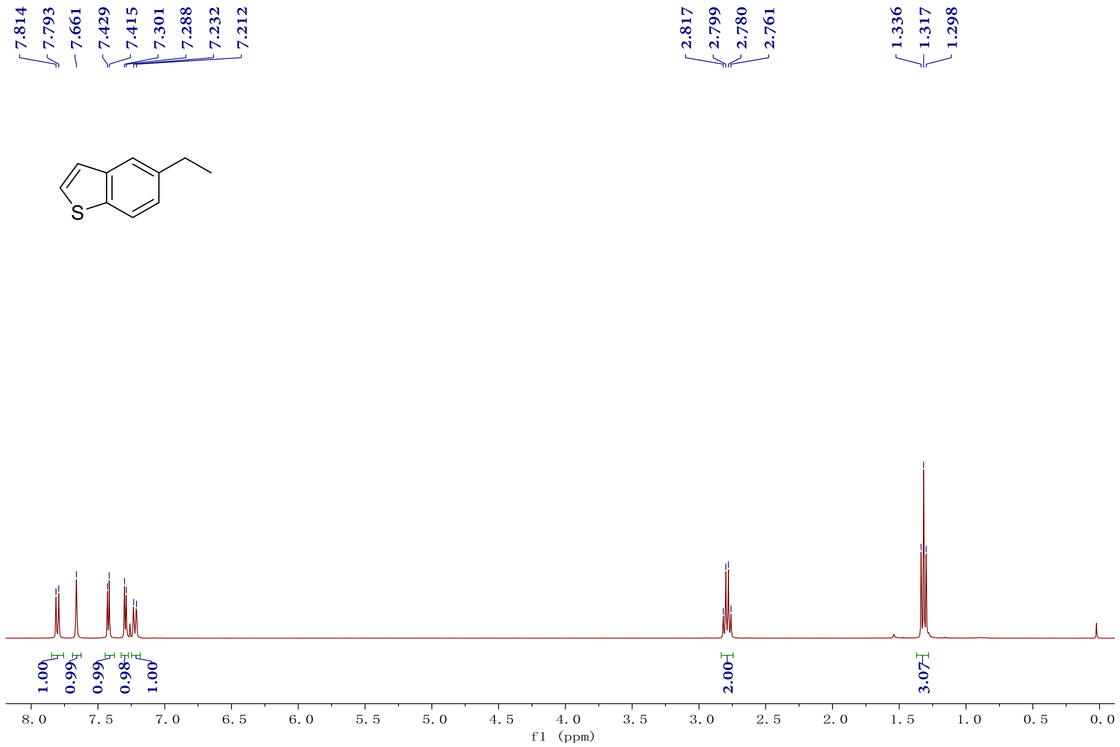

^{13}C NMR spectrum of **80** (**CDCl$_3$**)

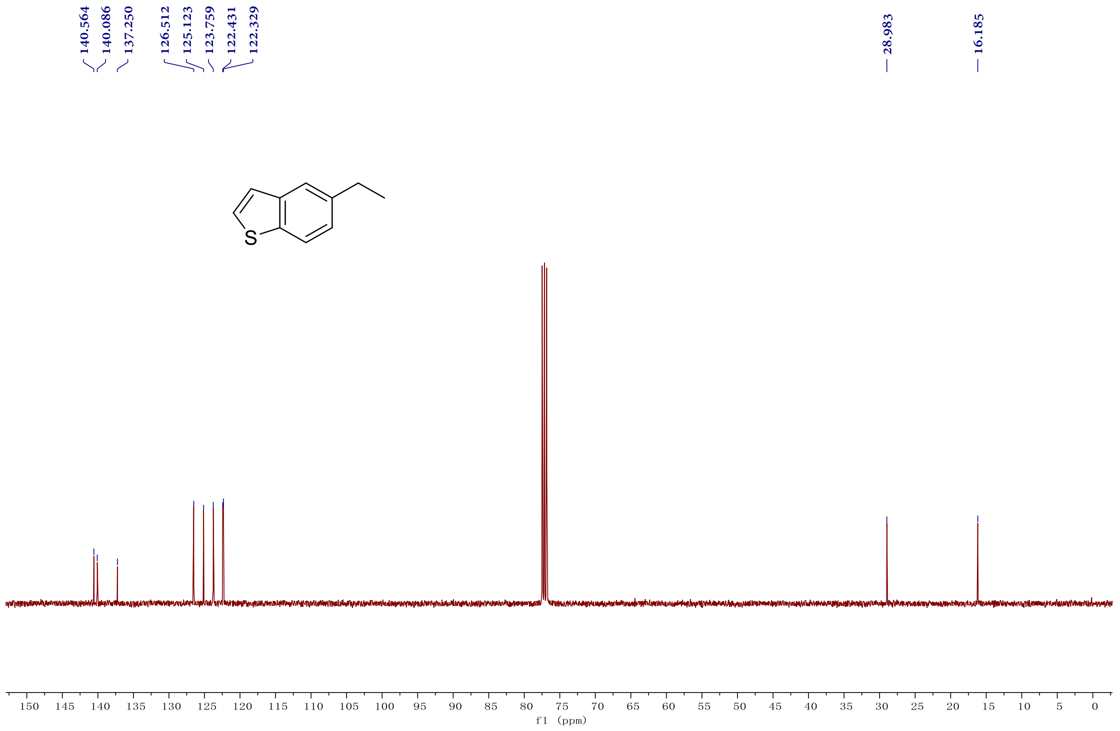

145

3. Supplementary references

1. Lipshutz, B. H., Ghorai, S. & Leong, W. W. Y. Deprotection of homoallyl (^{h}allyl) derivatives of phenols, alcohols, acids, and amines. *J. Org. Chem.* **74**, 2854–2857 (2009).

2. (a) McCourt, R. O. & Scanlan, E. M. Atmospheric oxygen mediated radical hydrothiolation of alkenes. *Chem .Eur. J.* **26**, 15804–15810 (2020); (b) Reddy, P. & Yu, B. Total synthesis of macrocyclic dysoxylactam A. *Chem Asian J.* **15**, 2467–2469 (2020).

3. Liu, R., Lu, Z.-H., Hu, X.-H., Li, H.-L. & Yang, X.-J. Monocarboxylation and intramolecular coupling of butenylated arenes via palladium-catalyzed C–H activation Process. *Org. Lett.* **17**, 1489−1492 (2015).

4. Yang, L., Lu, H-H., Lai, C-H., Li, G., Zhang, W., Cao, R., Liu, F., Wang, C., Xiao, J. & Xue, D. Light-promoted nickel catalysis: etherification of aryl electrophiles with alcohols catalyzed by a Ni^{II}-aryl complex. *Angew. Chem. Int. Ed.* **59**, 12714 –12719 (2020).

5. Ruhland, T., Andersen, K. & Pedersen, H. Selenium-linking strategy for traceless solid-phase synthesis: direct loading, aliphatic C−H bond formation upon cleavage and reaction monitoring by gradient MAS NMR spectroscopy. *J. Org. Chem*. **63**, 9204-9211 (1998).

6. Zhou, J., Jiang, B., Zhao, Z. & Shibata, N. Etherification of fluoroarenes with alkoxyboronic acid pinacol esters via C–F bond cleavage. *Org. Lett*. **24**, 5084–5089 (2022).

7. Patel, K. & Bedekar, A. Polyamine anchored palladium catalyst for suzuki–miyaura and one-pot O-alkylation-suzuki reactions. *Catal Lett*. **145**, 1710–1717 (2015).

8. Dhital, R., Sen, A., Sato, T., Hu, H., Ishii, R., Hashizume, D., Takaya, H., Uozumi, Y. & Yamada, Y. Activator-promoted aryl halide-dependent chemoselective buchwald–hartwig and Suzuki-miyaura type cross-coupling Reactions. *Org.Lett*. **22**, 4797−4805 (2020).

9. McCann, L., Hunter, H., Clyburne, J. & Organ, M. Higher-order zincates as

146

transmetalators in alkyl–alkyl negishi cross-coupling. *Angew. Chem. Int. Ed.* **51**, 7024–7027 (2012).

10. Caijo, F., Mosset, P., Grée, R., Audinot-Bouchez, V., Boutin, J., Renard, P., Caignard, D.-H., Dacquet, C. Synthesis of aromatic analogs of 8(S)-HETE and their biological evaluation as activators of the PPAR nuclear receptors. *Eur. J. Org. Chem.* 2181–2196 (2006).

11. Dang, H., Cox, N. & Lalic, G. Copper-catalyzed reduction of alkyl triflates and iodides: an efficient method for the deoxygenation of primary and secondary alcohols. *Angew. Chem. Int. Ed.* **53**, 752–756 (2014).

12. Stridfeldt, E., Lindstedt, E., Reitti, M., Blid, J., Norrby, P.-O. & Olofsson, B. Competing pathways in O-arylations with diaryliodonium salts: mechanistic insights. *Chem. Eur. J.* **23**, 13249–13258 (2017).

13. Wanga, Z.-Z., Wang, G.-Z., Zhao, B., Shang, R. & Fu, Y. Cobalt-catalyzed decarboxylative methylation and ethylation of aliphatic N-(acyloxy)phthalimides with organoaluminum reagents. *Synlett.* **31**, 1221–1225 (2020).

14. Yang, K., Li, Z., Wang, Z., Yao, Z. & Jiang, S. *Org. Lett.* **16**, 4340–4343 (2011).

15. Han, B., Ren, C. & Wu, L. Titanium-catalyzed hydrodehalogenation of alkyl halides. *Organometallics* **42**, 1248−1253 (2023).

16. Jiang, X., Tang, W., Xue, D., Xiao, J. & Wang, C. Divergent dehydrogenative coupling of indolines with alcohols. *ACS Catal.* **7**, 1831–1835 (2017).

17. Wang, Y., Zhu, D., Tang, L., Wang, D. & Wang, Z. Highly efficient amide synthesis from alcohols and amines by virtue of a water-soluble gold/DNA catalyst. *Angew. Chem. Int. Ed.* **50**, 8917–8921 (2011).

18. Zhao, F., Ai, H.-J. & Wu, X. Copper-catalyzed substrate-controlled carbonylative synthesis of α-keto amides and amides from alkyl halides. *Angew. Chem.Int. Ed.* **61**, e202200062 (2022).

19. Zhu, R., Jiang, J.-L., Li, X.-L., Deng, J. & Fu, Y. A comprehensive study on metal triflate-promoted hydrogenolysis of lactones to carboxylic acids: from synthetic and mechanistic perspectives. *ACS Catal.* **7**, 7520−7528 (2017).

20. MacNeil, C., Mendelsohn, L., Pabst, T., Hierlmeier, G. & Chirik, P. Alcohol

147

synthesis by cobalt-catalyzed visible-light-driven reductive hydroformylation. *J. Am. Chem. Soc.* **144**, 19219−19224 (2022).

21. Gevorgyan, A., Hopmann, K. & Bayer, A. Lipids as versatile solvents for chemical synthesis. *Green Chem.* **23**, 7219–7227 (2021).

22. Chen, G. & Xu, B. Hydrogen bond donor and unbalanced ion pair promoter-assisted gold-catalyzed carbon–oxygen cross-coupling of (hetero)aryl iodides with alcohols. *ACS Catal.* **13**, 1823−1829 (2023).

23. Huang, W., Tian, X., Jiao, H., Jackstell, R. & Beller, M. Iridium-catalyzed domino hydroformylation/hydrogenation of olefins to alcohols: synergy of two ligands. *Chem. Eur. J.* **28**, e202104012 (2022).

24. Sunderhaus, J. D., Lam, H. & Dudley, G. B. Oxidation of carbon−silicon bonds: the dramatic advantage of strained siletanes. *Org. Lett.* **5**, 4571–4573 (2003).

25. Zhang, L.-Y., Zhou, J.-H., Xu, Y.-H. & Loh, T.-P. Copper-catalyzed enantioselective conjugate addition of grignard reagents to methyl 4,4,4-trifluorocrotonate: synthesis of enantioenriched trifluoromethylated compounds. *Chem. Asian J.* **10**, 844–848 (2015).

26. Jia, Z., Liu, Q., Peng, X.-S. & Wong, H. N. C. Iron-catalysed cross-coupling of organolithium compounds with organic halides. *Nat. Commun.* **7**, 10614 (2016).

27. Guan, M., Miao, H., Qin, T., Zhang, G. & Zhang, Q. Retracted article: CoH-catalyzed radical hydroalkylation of alkenes with 1,3-dicarbonyls. *Chem. Commun.* **58**, 5265–5268 (2022).

28. Erchinger, J., Hoogesteger, R., Laskar, R., Dutta, S., Hümpel, C., Rana, D., Daniliuc, C. G. & Glorius, F. EnT-mediated N–S bond homolysis of a bifunctional reagent leading to aliphatic sulfonyl fluorides. *J. Am. Chem. Soc.* **145**, 2364−2374 (2023).

29. Liao, L.-L., Wang, Z.-H., Cao, K.-G., Sun, G.-Q., Zhang, W., Ran, C.-K., Li, Y., Chen, L., Cao, G.-M. & Yu, D.-G. Electrochemical ring-opening dicarboxylation of strained carbon–carbon single bonds with CO_2: facile synthesis of diacids and derivatization into polyesters. *J. Am. Chem. Soc.* **144**, 2062−2068 (2022).

30. Gaussian 09, Revision E.01, Frisch, M. J., Trucks, G. W., Schlegel, H. B., Scuseria, G. E., Robb, M. A., Cheeseman, J. R., Scalmani, G., Barone, V., Mennucci, B., Petersson, G. A., Nakatsuji, H., Caricato, M., Li, X., Hratchian, H. P., Izmaylov, A. F.,

148

Bloino, J., Zheng, G., Sonnenberg, J. L., Hada, M., Ehara, M., Toyota, K., Fukuda, R., Hasegawa, J., Ishida, M., Nakajima, T., Honda, Y., Kitao, O., Nakai, H., Vreven, T., Montgomery, J. A., Peralta, Jr., J. E., Ogliaro, F., Bearpark, M., Heyd, J. J., Brothers, E., Kudin, K. N., Staroverov, V. N., Keith, T., Kobayashi, R., Normand, J., Raghavachari, K., Rendell, A., Burant, J. C., Iyengar, S. S., Tomasi, J., Cossi, M., Rega, N., Millam, J. M., Klene, M., Knox, J. E., Cross, J. B., Bakken, V., Adamo, C., Jaramillo, J., Gomperts, R., Stratmann, R. E., Yazyev, O., Austin, A. J., Cammi, R., Pomelli, C., Ochterski, J. W., Martin, R. L., Morokuma, K., Zakrzewski, V. G., Voth, G. A., Salvador, P., Dannenberg, J. J., Dapprich, S., Daniels, A. D., Farkas, O., Foresman, J. B., Ortiz, J. V., Cioslowski, J. & Fox, D. J. Gaussian, Inc., Wallingford CT, 2013.

31. Becke, A. D. Density‐functional thermochemistry. III. The role of exact exchange. *J. Chem. Phys.* **98**, 5648−5652 (1993).

32. Lee, C., Yang, W. & Parr, R. G. Development of the Colle-Salvetti correlation-energy formula into a functional of the electron density. *Phys. Rev. B*, **37**, 785–789 (1988).

33. Grimme, S., Ehrlich, S. & Goerigk, L. Effect of the damping function in dispersion corrected density functional theory. *J. Comput. Chem.* **32**, 1456–1465 (2011).

34. Weigend, F. & Ahlrichs, R. Balanced basis sets of split valence, triple zeta valence and quadruple zeta valence quality for H to Rn: Design and assessment of accuracy. *Phys. Chem. Chem. Phys.* **7**, 3297–3305 (2005).

35. Deubel, D. V. & Lau, J. K. C. In silico evolution of substrate selectivity: comparison of organometallic ruthenium complexes with the anticancer drug cisplatin. *Chem. Commun.* 2451–2453 (2006).

36. Deubel, D. V. Mechanism and control of rare tautomer trapping at a metal−metal bond: adenine binding to dirhodium antitumor agents. *J. Am. Chem. Soc.* **130**, 665–675 (2008).

37. Lau, J. K. C. & Deubel, D. V. Hydrolysis of the anticancer drug cisplatin: pitfalls in the interpretation of quantum chemical calculations. *J. Chem. Theory Comput.* **2**, 103–106 (2006).

38. Plata, R. E. & Singleton, D. A. A case study of the mechanism of alcohol-mediated

149

morita baylis–hillman reactions. The importance of experimental observations. *J. Am. Chem. Soc.* **137**, 3811–3826 (2015).

39. Kua, J., Krizner, H. E. & De Haan, D. O. Thermodynamics and kinetics of imidazole formation from glyoxal, methylamine, and formaldehyde: a computational study. *J. Phys. Chem. A* **115**, 1667–1675 (2011).

40. Zhao, Y. & Truhlar, D. G. The M06 suite of density functionals for main group thermochemistry, thermochemical kinetics, noncovalent interactions, excited states, and transition elements: two new functionals and systematic testing of four M06-class functionals and 12 other functionals. *Theor. Chem. Acc.* **120**, 215–241 (2008).

41. Marenich, A. V., Cramer, C. J. & Truhlar, D. G. Universal solvation model based on solute electron density and on a continuum model of the solvent defined by the bulk dielectric constant and atomic surface tensions. *J. Phys. Chem. B.* **113**, 6378–6396 (2009).

42. Legault, C. Y. CYL View, version 1.0 b; Universite de Sherbrooke, Sherbrooke, Quebec, Canada, 2009; http://www.cylview.org.

43. Velian, A., Lin, S., Miller, A. J. M., Day, M. W. & Agapie, T. Synthesis and C−C coupling reactivity of a dinuclear NiI−NiI complex supported by a terphenyl diphosphine. *J. Am. Chem. Soc.* **132**, 6296–6297 (2010).

44. Jones, G. D., Martin, J. L., McFarland, C., Allen, O. R., Hall, R. E., Haley, A. D., Brandon, R. J., Konovalova, T., Desrochers, P. J., Pulay, P. & Vicic, D. A. Ligand redox effects in the synthesis, electronic structure, and reactivity of an alkyl−alkyl cross-coupling catalyst. *J. Am. Chem. Soc.* **128**, 13175–13183 (2006).

45. Iluc, V. M. & Hillhouse, G. L. Arrested 1,2-hydrogen migration from silicon to nickel upon oxidation of a three-coordinate Ni(I) silyl complex. *J. Am. Chem. Soc.* **132**, 11890–11892 (2010).

150
